# Peniexpansones A–F: polyketides from *Penicillium expansum* DWS880 capable of enhancing the activity of fluconazole against *Candida albicans*

**DOI:** 10.1007/s13659-026-00592-5

**Published:** 2026-03-03

**Authors:** Wen-Yu Lu, Qing-Hui Xiao, Ai-Lin Liang, Peng-Ju Xu, Jing Li, Wen-Xuan Wang

**Affiliations:** 1 Xiangya School of Pharmaceutical Sciences, Central South University, Changsha, 410008 Hunan People’s Republic of China; 2 Department of Pharmacy, National Clinical Research Center for Geriatric Disorder, Xiangya Hospital, Central South University, Changsha, 410008 Hunan People’s Republic of China; 3 Hunan Prima Drug Research Center Co., Ltd, Hunan Research Center for Drug Safety Evaluation, Hunan Key Laboratory of Pharmacodynamics and Safety Evaluation of New Drugs, Changsha, 410331 Hunan People’s Republic of China

**Keywords:** *Penicillium expansum*, Polyketides, ECD calculation, Anti-fungal activity enhancement

## Abstract

Six new polyketides, peniexpansones A–F (**1**–**4**,** 6**,** 7**), along with an additional novel compound, (2*E*,4*E*,6*E*)-8-methyldeca-2,4,6-trienoic acid (**5**), and a known naphthopyrone, were isolated from the ethyl acetate extract of rice fermented with the soil fungus *Penicillium expansum* DWS880. Structurally, peniexpansones A–D feature a highly oxygenated tetrahydronaphthalene moiety linked to an acyl chain. The structures of the new compounds were elucidated by extensive spectroscopic analysis (1D/2D NMR and HRESIMS) and further supported by quantum chemical calculations. Peniexpansone C showed weak cytotoxicity against HCT116 cells (IC_50_ 22.81 ± 0.42 μM) and moderate antimicrobial activity against pathogens including *Staphylococcus aureus* and *Candida albicans*. Moreover, peniexpansones A and B, can significantly improve the anti-fungal activity of fluconazole against resistant *Candida albicans*. Our results provided new structures for the future development of efficiency enhancement agents for anti-fungal drugs.

**Graphical Abstract:**

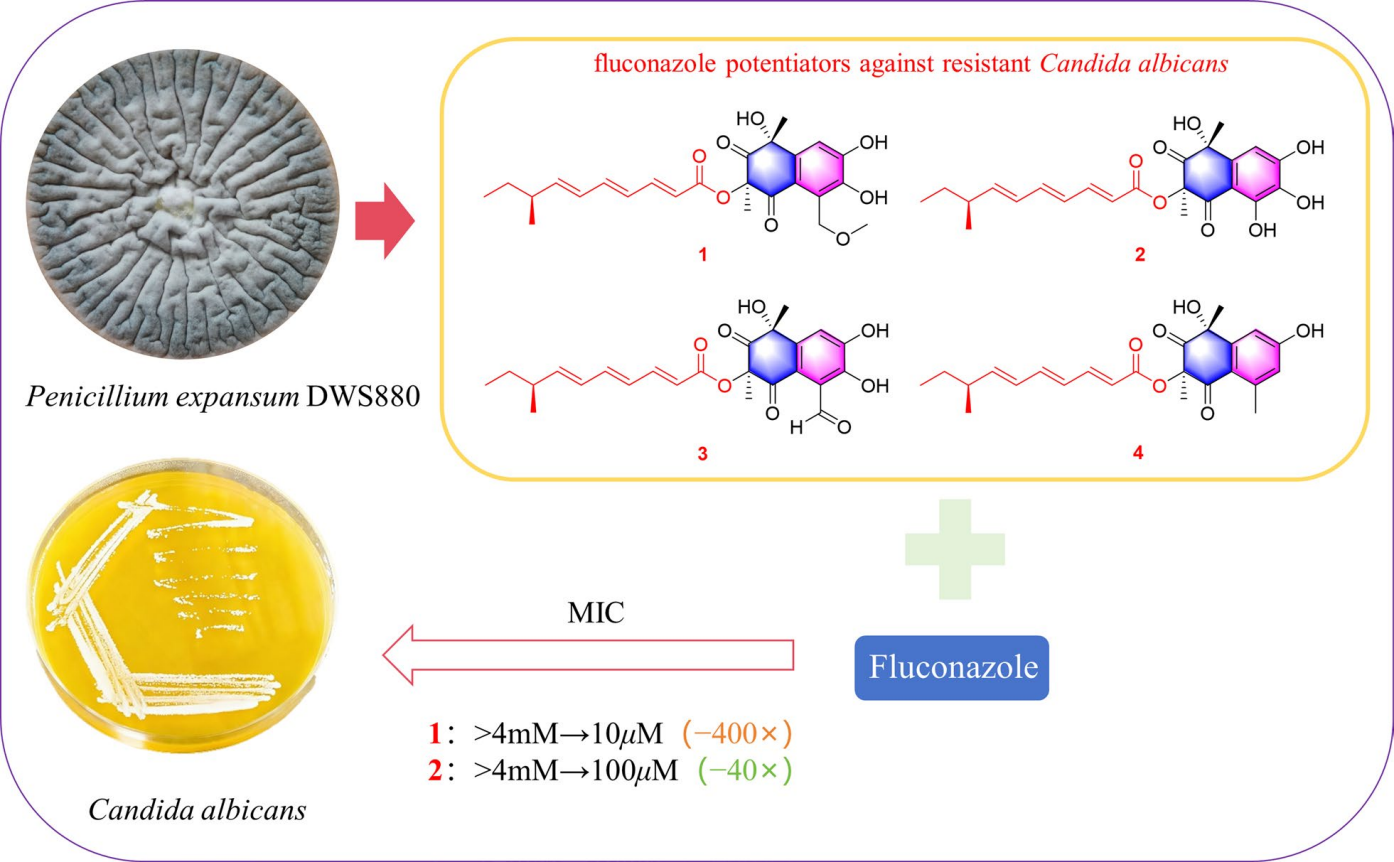

**Supplementary Information:**

The online version contains supplementary material available at 10.1007/s13659-026-00592-5.

## Introduction

Fungi are well-known producers of diverse small-molecule natural products, such as terpenes, polyketides, alkaloids, and peptides [1, 2]. Among these, polyketides have attracted significant interest owing to their structural complexity and broad biological activities, establishing them as privileged scaffolds in drug discovery [3–5]. To date, over 10,000 natural polyketides have been identified, many of which possess substantial commercial and therapeutic value [6]. Prominent examples include lovastatin [7]—a leading therapeutic that comprises over 80% of the global lipid-lowering market—as well as the antibiotic erythromycin [8], the antifungal griseofulvin [9], and the anti-angiogenic compound fumagillin [10].

Since the initial report in 2009 of the antioxidative polyketide JBIR-12 [11]—characterized by a highly oxygenated tetrahydronaphthalene core linked to an acyl chain—fewer than ten natural analogues of this distinctive scaffold have been documented. In 2023, the absolute configuration of the tetrahydronaphthalene core was tentatively proposed via ECD comparison [12]; nevertheless, a more rigorous and experimentally supported stereochemical determination remained to be established.

**Fig. 1:**
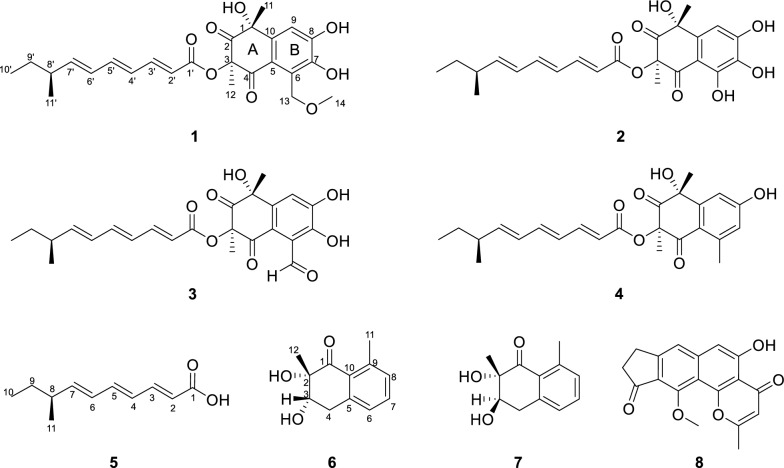
The structures of compounds **1**–**8**

In our ongoing investigation of bioactive metabolites from soil-derived fungi, *Penicillium expansum* DWS880 was isolated from a pristine humic layer collected in Daweishan National Forest Park. Chemical investigation of this strain led to the identification of six new polyketides, designated peniexpansones A–F. Structurally, peniexpansones A–D share the same rare architectural motif as JBIR-12. The complete and unequivocal absolute configuration for this structural family was established via 1D/2D NOE analysis and TDDFT-ECD calculations. All compounds were evaluated for cytotoxic and antimicrobial activities, providing a foundation for further exploration of their biological potential (Fig. 1).

## Results and discussion

### Structural analysis

Six new polyketides, peniexpansones A–F (**1**–**4**,** 6**,** 7**), were isolated from the ethyl acetate extract of the rice fermentation of the fungal strain. Structurally, compounds **1**–**4** each feature a 3-*O*-(2′*E*,4′*E*,6′*E*)-8′-methyldeca-2′,4′,6′-trienoate side chain with a chiral center at C-8′. Analysis of the NMR data indicated that the signals for this acyl side chain are highly consistent across compounds **1–4**. Furthermore, a new (2*E*,4*E*,6*E*)-8-methyldeca-2,4,6-trienoic acid (**5**) was isolated as a yellow oil. The absolute configuration of **5** was established as 8*S* by comparative analysis of its chiroptical data with those of the (8*R*)-enantiomer [12]. The experimental ECD spectrum exhibited a negative Cotton effect at *λ*_*max*_ 272 nm (Δ*ε* –5.7) and end absorption at 200 nm (Δ*ε* –7.8) (Table S81, Supporting Information), and a positive specific rotation was recorded ([α]_D_ + 3.8)—both opposite in sign to the reported *R* enantiomer ([α]_D_ –60, Cotton effects at 280 nm (Δ*ε* + 15) and 200 nm (Δ*ε* + 30) were both positive in the ECD spectrum). DFT calculations of specific rotation at the PBE1PBE/aug-cc-pvdz (IEFPCM, methanol) level further supported this assignment, yielding values of [α]_D_ –124.3 for 8*R* and + 124.3 for 8*S*, which align with the respective literature and experimental signs. Accordingly, the side chain in **1**–**4** was determined to be 3-*O*-(2′*E*,4′*E*,6′*E*)-8′*S*-methyldeca-2′,4′,6′-trienoate. The structural differences among **1**–**4** arise solely from the substitution patterns on their tetrahydronaphthalene moieties.

**Fig. 2:**
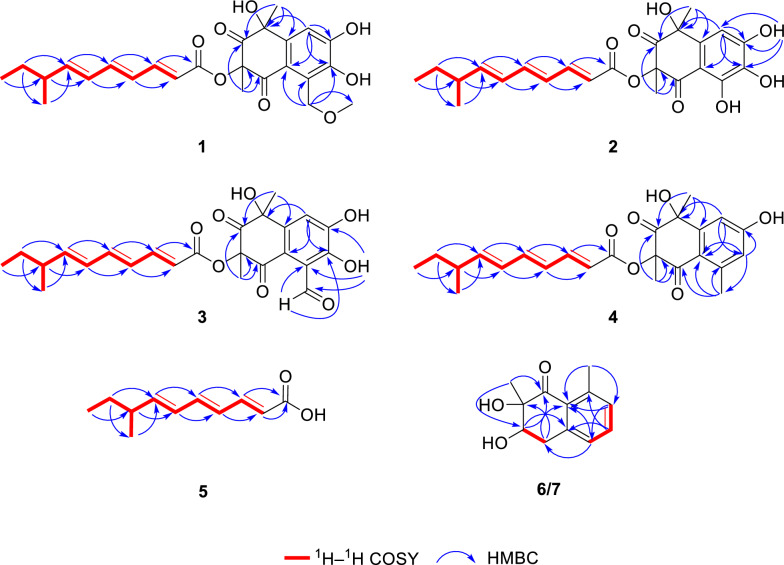
Key HMBC (H → C) and COSY (bold lines) correlations of compounds **1**–**7**

Compound **1** was obtained as a yellow oil, and the molecular formula was established as C_25_H_30_O_8_ by HR-ESI–MS analysis based on the sodium adduct ion at *m/z* 481.1856 [M + Na]⁺ (calcd for C_25_H_30_O_8_Na^+^, 481.1833), indicating 11 degrees of unsaturation. The ^1^H NMR spectrum (Table 1) exhibited signals for six olefinic protons (*δ*_H_ 7.39, 6.55, 6.27, 6.12, 5.99, 5.86), one aromatic proton (*δ*_H_ 7.33), one methine proton (*δ*_H_ 2.15), two methylenes (*δ*_H_ 5.28, 4.98, 1.36), four methyl groups (*δ*_H_ 1.71, 1.57, 1.02, 0.87), and one methoxy group (*δ*_H_ 3.52). The ^13^C NMR and DEPT spectra revealed 25 carbon resonances, including two ketone carbonyls (*δ*_C_ 206.1, 191.8), one ester carbonyl (*δ*_C_ 165.4), six olefinic carbons (*δ*_C_ 147.6, 147.2, 142.9, 128.2, 127.6, 117.3), six aromatic carbons (*δ*_C_ 150.7, 144.2, 138.1, 122.1, 119.1, 111.5), two oxygenated non-protonated carbons (*δ*_C_ 83.3, 75.7), one methine (*δ*_C_ 38.8), two methylene groups (one oxygenated at *δ*_C_ 73.0), and five methyl groups (one oxygenated at *δ*_C_ 59.2). Analysis of COSY and HMBC correlations of **1** indicated two distinct substructures (Fig. 2): a highly oxygenated tetrahydronaphthalene moiety and a branched alkyl chain. The tetrahydronaphthalene core was confirmed by key HMBC correlations from H-9 to C-1/C-5/C-7/C-8, from H_2_-13 to C-5/C-6/C-7, from H_3_-11 to C-1/C-2/C-10, and from H_3_-12 to C-2/C-3/C-4. The 1D NMR data of **1** (Table 1) were highly similar to those of JBIR-12 [11], differing only by the replacement of a hydroxy group at C-13 in JBIR-12 with a methoxy group in **1**, a modification supported by the observed HMBC correlation from H_2_-13 to C-14. The branched alkyl chain moiety was identical to that of JBIR-12, as evidenced by COSY correlations (H-10′/H-9′/H-8′/H-11′ and H-8′/H-7′/H-6′/H-5′/H-4′/H-3′/H-2′) and supporting HMBC data. The large coupling constants observed for H-2′/H-3′ (*J* = 15.3 Hz), H-4′/H-5′ (*J* = 14.8 Hz), and H-6′/H-7′ (*J* = 15.2 Hz) are consistent with *trans*-configured double bonds. This, combined with a key HMBC correlation from H-3′ to the ester carbonyl (C-1′), established the side chain as a (2*E*,4*E*,6*E*)-8-methyldeca-2,4,6-trienoate substituent.

**Table 1:** The ^1^H NMR data (600 MHz) and ^13^C NMR data (150 MHz) of compounds **1**–**4** in CDCl_3_ (*δ* in ppm)

No	1		2		3		4	
	*δ*_H_	*δ*_C,_ type	*δ*_H_	*δ*_C,_ type	*δ*_H_	*δ*_C,_ type	*δ*_H_	*δ*_C,_ type
1		75.7, C		74.4, C		75.4, C		76.3, C
2		206.1, C		205.0, C		205.2, C		206.5, C
3		83.3, C		81.4, C		83.1, C		83.3, C
4		191.8, C		196.5, C		191.2, C		191.4, C
5		119.1, C		107.0, C		121.6, C		120.6, C
6		122.1, C		151.4, C		116.9, C		143.8, C
7		144.2, C		130.8, C		151.2, C	6.70, s	118.8, CH
8		150.7, C		149.7, C		150.7, C		160.2, C
9	7.33, s	111.5, CH	6.93, s	106.7, CH	7.59, s	117.2, CH	7.14, s	110.5, CH
10		138.1, C		136.9, C		138.2, C		146.7, C
11	1.71, s	34.8, CH_3_	1.71, s	31.9, CH_3_	1.75, s	34.1, CH_3_	1.74, s	35.2, CH_3_
12	1.57, s	23.5, CH_3_	1.69, s	23.9, CH_3_	1.67, s	23.2, CH_3_	1.53, s	23.5, CH_3_
13	5.28, d (15.0) 4.98, d (15.0)	73, CH_2_			10.49, s	197.8, CH	2.52, s	21.8, CH_3_
14	3.52, s	59.2, CH_3_						
1'		165.4, C		165.9, C		165.4, C		165.6, C
2'	5.99, d (15.3)	117.3, CH	5.96, d (15.3)	116.9, CH	5.97, d (15.3)	117.0, CH	5.99, d (15.3)	117.4, CH
3'	7.39, dd (15.3,11.3)	147.6, CH	7.38, dd (15.3,11.3)	148.0, CH	7.40, dd (15.3,11.3)	147.9, CH	7.40, dd (15.3,11.3)	147.6, CH
4'	6.27, dd (14.8,11.3)	127.6, CH	6.26, dd (14.8,11.3)	127.5, CH	6.27, dd (14.8,11.3)	127.5, CH	6.26, dd (14.8,11.3)	127.7, CH
5'	6.55, dd (14.8,10.8)	142.9, CH	6.56, dd (14.8,10.8)	143.2, CH	6.57, dd (14.8,10.8)	143.2, CH	6.55, dd (14.8,10.8)	142.9, CH
6'	6.12, dd (15.2,10.8)	128.2, CH	6.12, dd (15.2,10.8)	128.2, CH	6.13, dd (15.2,10.8)	128.1, CH	6.12, dd (15.2,10.8)	128.2, CH
7'	5.86, dd (15.2,8.0)	147.2, CH	5.86, dd (15.2,8.0)	147.5, CH	5.87, dd (15.2,8.0)	147.5, CH	5.85, dd (15.2,8.0)	147.2, CH
8'	2.15, m	38.8, CH	2.15, m	38.8, CH	2.15, m	38.8, CH	2.15, m	38.8, CH
9'	1.36, m	29.5, CH_2_	1.36, m	29.4, CH_2_	1.36, m	29.4, CH_2_	1.36, m	29.5, CH_2_
10'	0.87, t (7.3)	11.7, CH_3_	0.87, t (7.3)	11.7, CH_3_	0.87, t (7.3)	11.7, CH_3_	0.87, t (7.3)	11.7, CH_3_
11'	1.02, d (6.8)	19.6, CH_3_	1.02, d (6.8)	19.6, CH_3_	1.03, d (6.8)	19.6, CH_3_	1.02, d (6.8)	19.6, CH_3_
7-OH					12.76, s			
8-OH			11.82, s

**Fig. 3:**
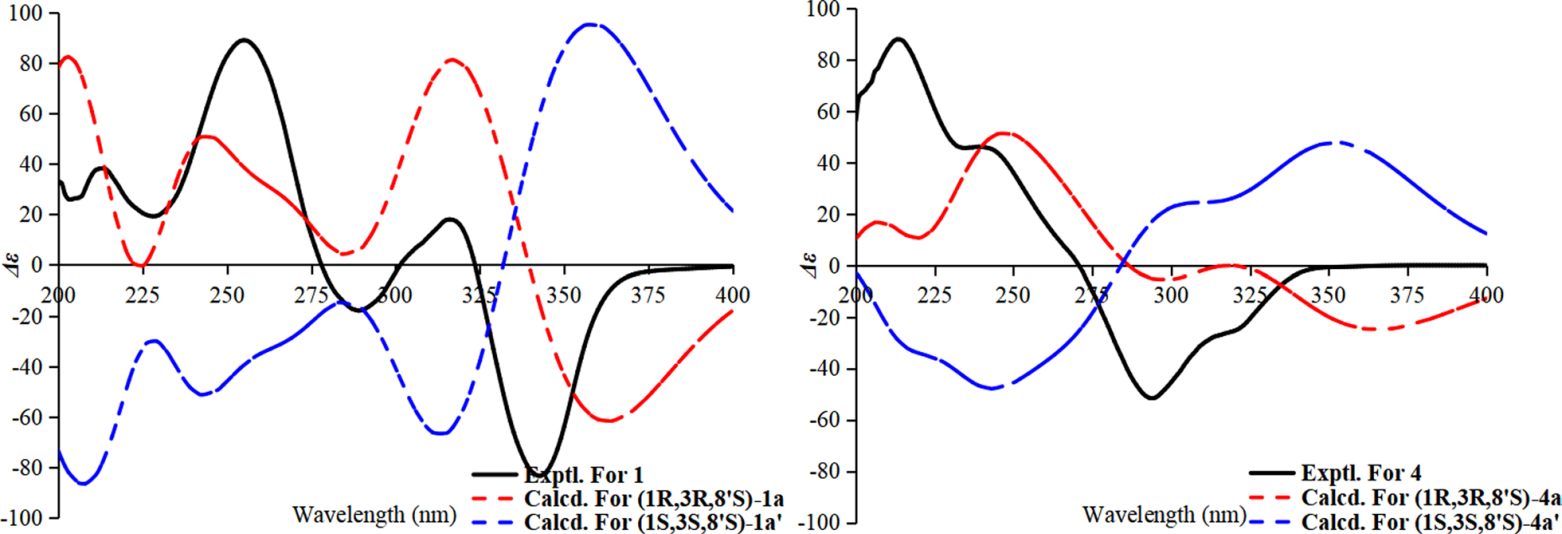
The experimental and calculated ECD curves of **1** and **4**

The relative configuration of **1** was established through analysis of 2D NOESY and selective 1D NOE experiments. In alignment with the structural assignment of penijanthinone A [13], 2D NOESY correlations between H_3_-11 (*δ*_H_ 1.71) and H_3_-12 (*δ*_H_ 1.57) were absent. Furthermore, selective irradiation of H_3_-11 or H_3_-12 yielded no observable NOE enhancement of the other methyl group (Fig. S7, Supporting Information), indicating that H_3_-11 and H_3_-12 are oriented on opposite faces of ring A in **1**. To determine the absolute configuration, the ECD spectra of 1*R*,3*R*,8′S-**1a** and 1*S*,3*S*,8′*S*-**1a′** were calculated using the B3LYP/TZVP//B3LYP-D3(BJ)/TZVP level with the IEFPCM solvation model in methanol. As shown in Fig. 3, the calculated ECD curve for 1*R*,3*R*,8′*S*-**1** closely matched the experimental spectrum. Thus, the absolute configuration of **1** was assigned as 1*R*,3*R*,8′*S*, and the compound was designated peniexpansone A.

**Fig. 4:**
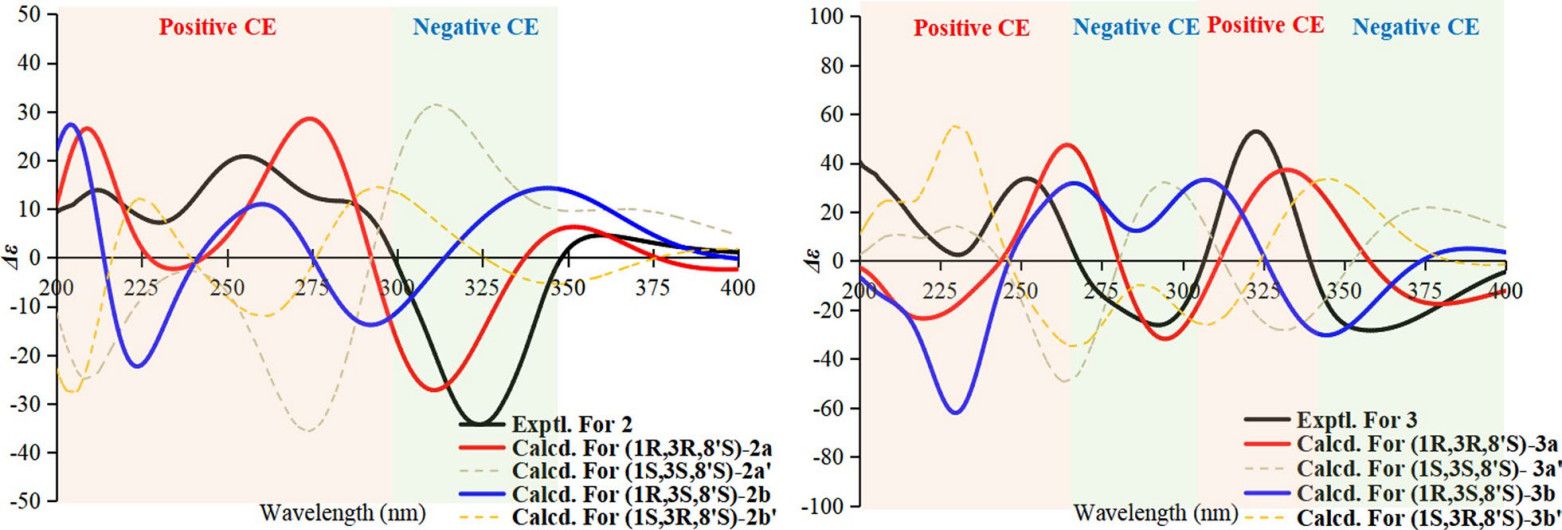
The experimental and calculated ECD curves of **2** and **3**

**Fig. 5:**
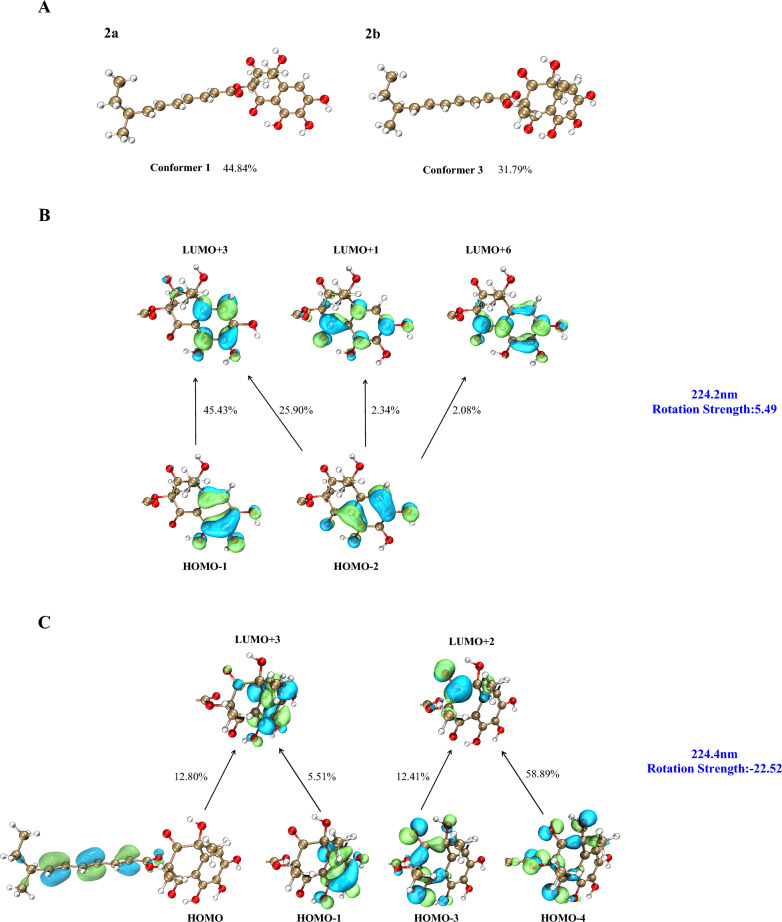
Orbital analysis of the characteristic CEs for structures **2a** (3*R*) and **2b** (3*S*). **A** The lowest-energy conformers of **2a** and **2b**, and their population. **B** Representative electronic transition orbitals in **2a** contributing to the positive CE at 224.2 nm, and their percentage contributions. **C** Representative electronic transition orbitals in **2b** contributing to the negative CE at 224.4 nm, and their percentage contributions

Compound **2** was obtained as a yellow oil, and the molecular formula was established as C_23_H_26_O_8_ by HR-ESI–MS at *m/z* 431.1721 [M + H]⁺ (calcd for C_23_H_27_O_8_^+^, 431.1700), indicating 11 degrees of unsaturation. Comparative analysis of the NMR data revealed that **1** and **2** share an identical core scaffold, differing structurally only at the C-6 position. The distinctive structural motif of three contiguous phenolic hydroxy groups at C-6, C-7, and C-8 in compound **2** was established by a definitive downfield shift of the C-6 resonance (*δ*_C_ 151.4) relative to that in **1** (*δ*_C_ 122.1), together with the key HMBC correlation from OH‑8 to C‑7/C‑8/C‑9. Due to signal overlap between H_3_-11 (*δ*_H_ 1.71) and H_3_-12 (*δ*_H_ 1.69), definitive NOE correlations for **2** could not be obtained. Attempts to determine the relative configuration via GIAO ^13^C NMR chemical shift calculations (using the STS protocol) [14] for the four possible diastereomers were unsuccessful; the computed chemical shifts showed insufficient dispersion and yielded *P*_rel_ values below the 95% confidence threshold required for a reliable stereochemical assignment (Table S67). Consequently, TDDFT-ECD calculations were performed for the four diastereomeric candidates, **2a** (1*R*,3*R*,8′*S*), **2a′** (1*S*,3*S*,8′*S*), **2b** (1*R*,3*S*,8′*S*), and **2b′** (1*S*,3*R*,8′*S*) at the B3LYP/TZVP//B3LYP-D3(BJ)/TZVP level. The experimental ECD spectrum of **2** exhibited a positive Cotton effect (CE) at 200–300 nm and a negative Cotton effect at 300–350 nm. Among the computed spectra, only that of **2a** reproduced both of these features, showing close agreement with the experimental data (Fig. 4). The calculated ECD curves for **2a** and **2a′** (as well as for **2b** and **2b′**) displayed a nearly enantiomeric relationship, indicating that the configuration at C-8′ has a negligible influence on the overall ECD profile. Moreover, the calculated ECD curve of **2b** showed negative CE at the region around 224 nm, which is the most significant discrepancy with the experimental curve. We further analyzed its dominant molecular orbitals contributing to the 224 nm band, and found that its negative CE is mainly caused by the transition from the *n* orbitals of oxygen atoms close to position 3 to the π* orbital of the 2-CO group (Fig. 5). While this transition is absent in **2a** at the same band. Consequently, the configuration at C-3 should be the same as that in **2a**, namely *R*. Thus, the absolute configuration of **2** was assigned as 1*R*,3*R*,8′*S*, and the compound was designated peniexpansone B.

**Fig. 6:**
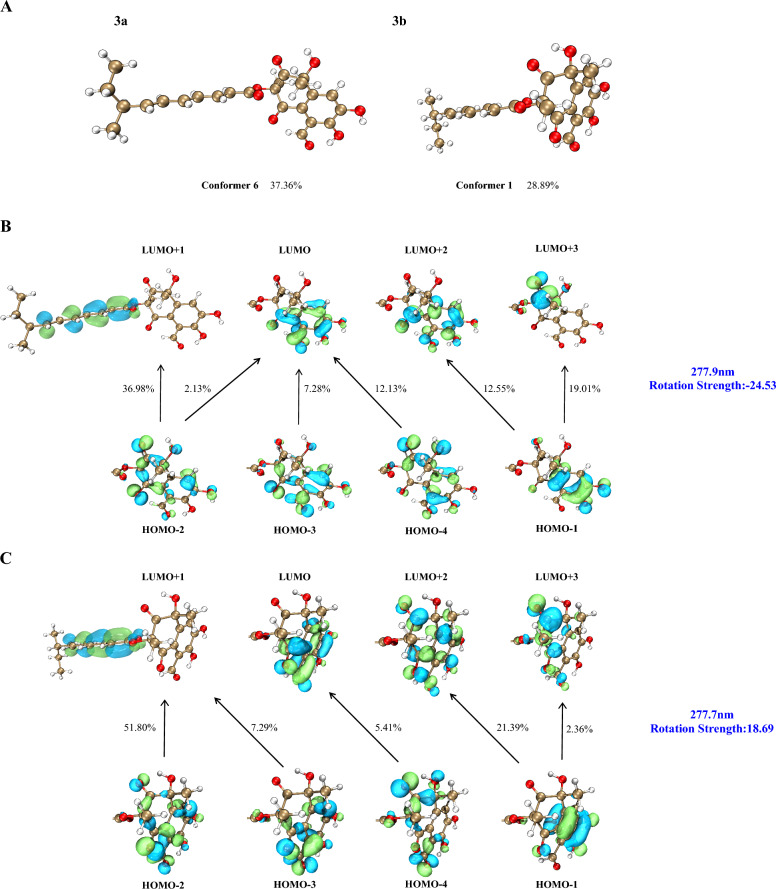
Orbital analysis of the characteristic CEs for structures **3a** (3*R*) and **3b** (3*S*). **A** The lowest-energy conformers of **3a** and **3b**, and their population. **B** Representative electronic transition orbitals in **3a** contributing to the negative CE at 277.9 nm, and their percentage contributions. **C** Representative electronic transition orbitals in **3b** contributing to the positive CE at 277.7 nm, and their percentage contributions

Compound **3** was obtained as a yellow oil. HR-ESI–MS analysis established its molecular formula as C_24_H_26_O_8_, based on the observed [M + H]⁺ ion at *m/z* 443.1713 (calcd for C_24_H_27_O_8_^+^, 443.1700), indicating 12 degrees of unsaturation. Comparative analysis of the ^1^H and ^13^C NMR data of **3** with those of **2** (Table 1) revealed an identical core scaffold. The key structural difference was the conversion of the C-6 substituent from a hydroxy group in **2** to an aldehyde group in **3**, a change unequivocally supported by HMBC correlations from the aldehyde proton (CHO-13) to C-6 and C-7. Determination of the relative configuration of **3** was impeded by challenges similar to those with **2**: signal overlap between H_3_-11 (*δ*_H_ 1.75) and H_3_-12 (*δ*_H_ 1.67) precluded definitive NOE correlations, and GIAO ^13^C NMR chemical shift calculations (STS protocol) [14] for the four possible diastereomers again exhibited insufficient dispersion (*P*_rel_ < 95%; Table S68). Consequently, the absolute configuration was assigned by comparing the experimental ECD spectrum of **3** with TDDFT-ECD calculated curves. Geometry optimizations and subsequent ECD calculations were conducted at the B3LYP/TZVP//B3LYP-D3(BJ)/TZVP level in methanol for all candidate stereoisomers: **3a** (1*R*,3*R*,8′*S*), **3a′** (1*S*,3*S*,8′*S*), **3b** (1*R*,3*S*,8′*S*), and **3b′** (1*S*,3*R*,8′*S*). The experimental ECD spectrum of **3** displayed four major Cotton effects—positive, negative, positive, and negative—between 200 and 400 nm. Among all computed spectra, only that of diastereomer **3a** (1*R*,3*R*,8′*S*) closely reproduced both the sign and magnitude of these effects (Fig. 4). Structures **3a** (3*R*) and **3b** (3*S*) are epimers at postion 3, and show opposite CE at the band around 278 nm, indicating that the chiral center at position 3 plays major role in their different optical features. We further analyzed the orbital contributions within this absorption band, and found that the transitions are dominated by excitation from the *n* orbitals of the oxygen atoms on the core skeleton to the π* orbital of the side chain, with the chiral center at position 3 serving as a pivotal hub (Fig. 6). The negative CE of the structure **3a** in this band matches the experimental value, confirming that the configuration at position **3** is *R*. Consequently, the absolute configuration of **3** was assigned as 1*R*,3*R*,8′*S*, and the compound was designated peniexpansone C.

Compound **4** was obtained as a yellow oil, and the molecular formula was established as C_24_H_28_O_6_ by HR-ESI–MS analysis based on the detection of an [M + H]⁺ ion at *m/z* 413.1976 (calcd for C_24_H_29_O_6_^+^, 413.1959), indicating 11 degrees of unsaturation. The NMR spectra of **4** closely resembled those of **2**, indicating an identical core structure with two key modifications: the hydroxy group at C-6 was supplanted with a methyl group (H_3_-13, *δ*_H_ 2.52), and the hydroxy substituent at C-7 was replaced by an aromatic proton (H-7, *δ*_H_ 6.70). The former modification was confirmed by an HMBC correlation from H_3_-13 to C-4/C-5/C-6, and the latter by correlations from H-7 to C-5/C-8/C-13 (Fig. 2). The relative configuration of **4** was determined through selective 1D NOE experiments, applying the strategy previously used for **1**. Irradiation of H_3_-11 did not yield an observable enhancement of H_3_-12 (Fig. S35), indicating that these methyl groups are oriented on opposite faces of ring A. The absolute configuration of compound **4** was assigned as 1*R*,3*R*,8′*S* by comparing the experimental ECD spectrum with the TDDFT-calculated ECD curves of the two candidate diastereomers, **4a** (1*R*,3*R*,8′*S*) and **4a′** (1*S*,3*S*,8′*S*) (Fig. 3). Thus, the complete structure of **4** was elucidated and assigned the name peniexpansone D. The consistent assignment of the 1*R*,3*R*,8′*S* configuration across these compounds — based on ECD analysis —suggests a highly conserved biosynthetic pathway, indicative of a common genetic origin within this fungal source.

Compounds **6** and **7** were identified as a pair of epimers, both exhibiting the molecular formula C_12_H_14_O_3_ as established by HR-ESI–MS analysis, with sodium adduct ions observed at *m/z* 229.0835 [M + Na]⁺ for compound **6** and *m/z* 229.0809 [M + Na]⁺ for compound **7** (calcd for C_12_H_14_O_3_Na^+^, 229.0835), consistent with 6 degrees of unsaturation. The similarity in their ^1^H and ^13^C NMR spectra (Table 2) suggested that both compounds share the same carbon skeleton. The key feature in the ^1^H NMR spectra of both compounds was a set of aromatic protons indicative of a 1,2,3-trisubstituted benzene ring. Additional signals were consistent with an oxygenated methine, a methylene group, and two methyl groups. The ^13^C NMR data confirmed the presence of 12 carbon resonances, which were classified as one ketone carbonyl, one oxygenated non-protonated carbon, one oxygenated methine, one methylene group, two methyl groups, and six aromatic carbons. COSY correlations revealed two spin systems: H_2_-4/H-3 and H-6/H-7/H-8. The planar structures were further elucidated by key HMBC correlations, observed from H-8 to C-6 and C-10; from H-7 to C-5 and C-9; from H-6 to C-4 and C-10; from H_3_-11 to C-8 and C-10; from H_3_-12 to C-1 and C-3; from H-4 to C-2 and C-10; and from H-3 to C-1 and C-5 (Fig. 2). On the basis of these results, compounds **6** and **7** were deduced to be tetralone derivatives [15].

**Table 2:** The ^1^H NMR data (600 MHz) and ^13^C NMR data (150 MHz) of compounds **6** and **7** in CDCl_3_ (*δ* in ppm)

No	6		7	
	*δ*_H_	*δ*_C,_ type	*δ*_H_	*δ*_C,_ type
1		202.2, C		202.4, C
2		77.2, C		78.6, C
3	4.28, dd (2.3,3.7)	74.0, CH	4.08, dd (6.0,11.3)	72.7, CH
4	3.34, dd (3.7,18.1) 3.27, dd (2.3,18.1)	33.4, CH_2_	3.29, dd (6.0,17.0) 3.02, dd (11.3,17.0)	34.9, CH_2_
5		140.6, C		140.9, C
6	7.11, d (7.6)	130.4, CH	7.16, overlapped	130.7, CH
7	7.40, dd (7.5,7.6)	133.7, CH	7.40, dd (7.5,7.6)	133.4, CH
8	7.14, d (7.5)	127.7, CH	7.15, overlapped	127.5, CH
9		141.8, C		142.2, C
10		127.7, C		127.9, C
11	2.63, s	22.6, CH_3_	2.63, s	22.4, CH_3_
12	1.36, s	22.4, CH_3_	1.29, s	17.1, CH_3_
2-OH	4.41, s			
3-OH	2.79, s			

**Fig. 7:**
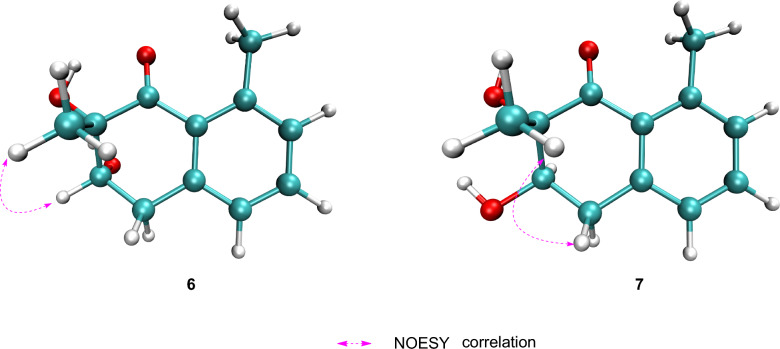
Key NOESY correlations of compounds **6** and **7**

**Fig. 8:**
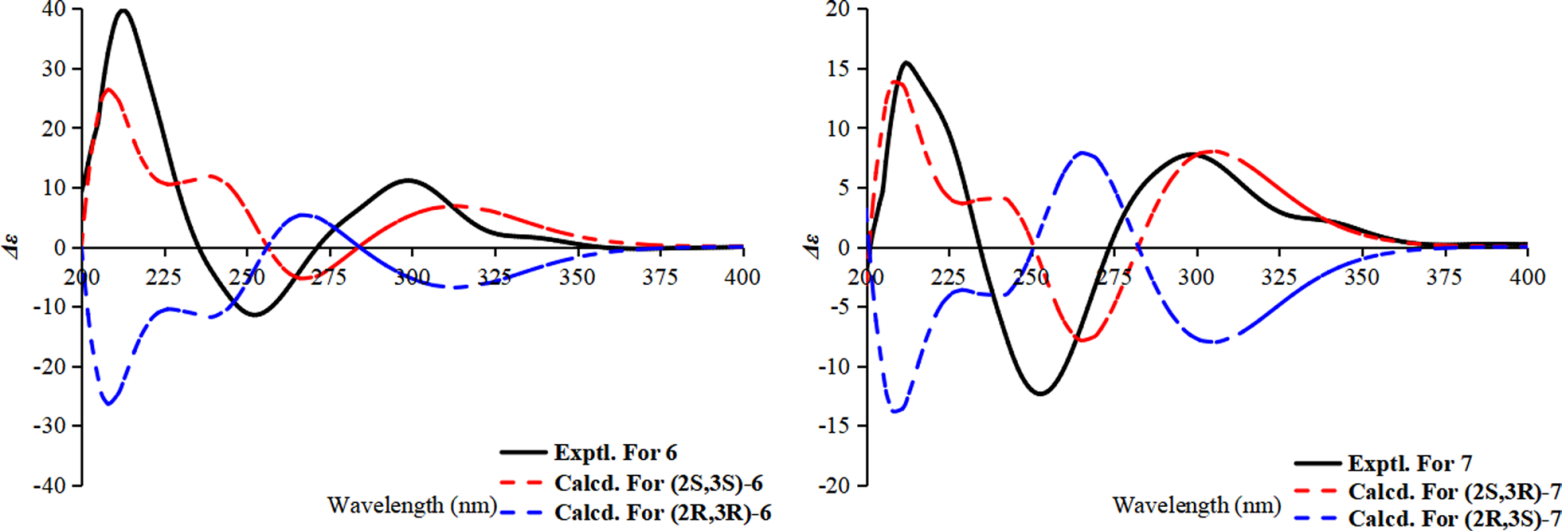
The experimental and calculated ECD curves of **6** and **7**

The relative configuration of **6** was established by a key NOESY correlation between H-3 and H_3_-12, indicating their co-facial orientation on the cyclohexanone ring (Fig. 7). For **7**, the large coupling constant *J*_3,4_ (11.3 Hz) between H-4b (*δ*_H_ 3.02) and H-3 indicated a trans-diaxial relationship, while a NOESY correlation between H-4b and H_3_-12 placed H-3 and the methyl group on opposite faces. The absolute configurations of the cyclohexanone moiety in **6** and **7** were assigned as 2*S*,3*S* and 2*S*,3*R* by comparison of experimental and calculated ECD spectra (Fig. 8); thus, the compounds were designated peniexpansones E and F, respectively.

By comparing spectroscopic properties with the reported data [16], the structure of **8** was elucidated as ligustrone B, first isolated from *Cercospora ligustrina* in 1975 [17].

### Cytotoxicity and anti-microbial assays

Among the tested compounds, only **3** exhibited notable antimicrobial activity. It showed potent effects against *Staphylococcus aureus* (MIC = 25.0 μM), comparable to streptomycin (MIC = 22.0 μM), and inhibited *Acinetobacter baumannii*, *Saccharomyces cerevisiae*, and *Candida albicans* (MIC = 50.0 μM each). Notably, **3** was significantly more active than terbinafine (MIC = 439.2 μM) and fluconazole (MIC > 4 mM) against resistant *C. albicans*. None of the other compounds (**1**, **2**, **4**–**8**) were active (MIC > 50 μM) against the microbial strains tested.

Strikingly, despite their lack of intrinsic antifungal activity, compounds **1** and **2** demonstrated a profound ability to potentiate fluconazole against the resistant *C. albicans* isolate. The potentiation effect was concentration-dependent and remarkably strong for compound **1**, which at 40 and 20 μM reduced the fluconazole MIC from > 4 mM to 10 μM and 25 μM, respectively. Similarly, compound **2** at 40 μM lowered the fluconazole MIC to 100 μM. In contrast, compounds **3** and **4** showed only weak potentiation, reducing the fluconazole MIC to 500 μM and 800 μM at 40 μM, respectively (Fig. S85-90, Supporting Information).

In cytotoxicity evaluations against HCT116, 4T1, and MHCC97H cancer cell lines, only compound **3** exhibited weak activity against HCT116 cells, with an IC_50_ value of 22.81 ± 0.42 μM. The other compounds showed no significant cytotoxicity across all tested cancer lines.

The selectivity and safety profiles of these compounds were further assessed using the human gastric epithelial cell line GES-1. Notably, compounds **1** and **2** displayed no cytotoxicity toward GES-1 cells at a concentration of 50 μM, underscoring their favorable safety profile as potential fluconazole potentiators. In contrast, compound **3** demonstrated moderate cytotoxicity against GES-1 cells.

## Experimental

### General experiment procedures

UV and CD spectra were acquired on a Chirascan™-plus circular dichroism spectrometer. Optical rotations were measured using a Rudolph Research Analytical Autopol IV automatic polarimeter. IR spectra were recorded on a Shimadzu Fourier transform infrared spectrometer with KBr pellets. High-resolution electrospray ionization mass spectrometry (HR-ESI–MS) spectra were acquired with an Agilent 6500 series Q-TOF mass spectrometer (Agilent Technologies, Singapore). NMR spectra were recorded at 600 MHz for ^1^H and 150 MHz for ^13^C on Bruker spectrometers (Bruker BioSpin, Rheinstetten, Germany). Column chromatography was performed using macroporous adsorbent resin D101 (Tianjin Haoju Resin Technology Co., LTD., Tianjin, China), silica gel (200 − 300 mesh, Qingdao Marine Chemical, Qingdao, China), and Sephadex LH-20 (GE Healthcare, Uppsala, Sweden). HPLC separations were conducted using an Agilent 1260 system, which included an automatic sampler, a pump, and a wavelength absorbance detector, fitted with an Agilent semipreparative column (9.4 mm × 150 mm, C18, 5 μm) (Agilent Technologies). Additionally, an EClassical P3500 prep-HPLC system equipped with a Sinochrom ODS-BP column (Elite, 5 μm, 10 mm × 250 mm), Phenyl-Hexyl (Shimadzu, 5 μm, 10 mm × 250 mm) and a DAD detector (EClassical P3500, Dalian Elite Analytical Instruments Co., Ltd., Dalian, China) was used. Thin-layer chromatography (TLC) separations were performed on precoated silica gel GF254 plates (Qingdao Marine Chemical), with spots visualized under UV light (254 or 365 nm) or by spraying with 10% H_2_SO_4_ in ethanol followed by heating.

### Fungal material

The fungal strain DWS880 was isolated from a soil sample collected from Daweishan Forest Park, Hunan Province, China, in 2022. This strain was identified as *P. expansum* based on ITS sequencing, with a percentage identity of 99.50% to be *P. expansum* for ITS (Table S84). The fungal specimen is deposited at the Xiangya School of Pharmaceutical Sciences, Central South University, Changsha, China.

### Extraction and isolation

The fungal strain was initially cultivated on potato dextrose agar for 8 days at 25 °C. The mycelia were then cut into pieces and cultured on a cooked rice medium, prepared by mixing 100 g of rice with 80 mL of water in 500 mL Erlenmeyer flasks. The total weight of the rice used was 5 kg. The rice cultures were maintained at 25 °C for 71 d. After incubation at 25 °C for 71 d, the rice was chopped and exhaustively extracted with EtOAc (15 L, five cycles, room temperature). The solvent was then evaporated under vacuum to yield 90 g of crude extract. The crude extract was adsorbed onto D101 macroporous resin and eluted stepwise with water, 20% methanol, 80% methanol, and 100% methanol. The 80% aquatic MeOH portion was collected and condensed to yield 60 g residue. The 80% MeOH portion was subjected to silica gel column flushing with gradient petroleum ether and ethyl acetate (50:1 → 1:1, v/v) to afford fractions A–G. Fraction D was separated by Sephadex LH-20 (MeOH) to afford three sub-fractions (Fr. D1–Fr. D3). Fr. D2 was purified by semipreparative HPLC (MeOH–H_2_O, 60:40 → 82:18, v/v, 27 min, 3.0 mL/min, Phenyl-Hexyl column, 10 × 250 mm, 5 μm) to yield six subfractions (Fr. D21–Fr. D26). Fr. D21 was further purified by semipreparative HPLC ((MeOH–H_2_O, 43:57, v/v, 30 min, 3.0 mL/min, Phenyl-Hexyl, 10 mm × 250 mm, 5 μm) to yield compound** 6** (1.2 mg, t_R_ = 21.89 min) and **7** (2.0 mg, t_R_ = 24.84 min). Fr. F was separated by Sephadex LH-20 (MeOH) to afford four sub-fractions (Fr. F1 − Fr. F4). Fr. F2 was purified by semipreparative HPLC (MeCN–H_2_O, 35:65 → 75:25, v/v, 35 min, 3.0 mL/min, ZORBAX-C18, 9.4 mm × 150 mm, 5 μm) to yield compound **5** (7.0 mg, t_R_ = 20.55 min), **2** (6.0 mg, t_R_ = 23.13 min), **3** (7.0 mg, t_R_ = 25.54 min), and **4** (3.0 mg, t_R_ = 28.61 min). Fr. F3 was purified by semipreparative HPLC (MeCN–H_2_O, 50:50 → 75:25, v/v, 25 min, 3.0 mL/min, Sinochrom ODS-BP, 10 mm × 250 mm, 5 μm) to yield compound **8** (10.0 mg, t_R_ = 13.46 min). Fr. G was separated by Sephadex LH-20 (MeOH) to afford four sub-fractions (Fr. G1 − Fr. G4). Fr. G2 was further purified by semipreparative HPLC (MeCN–H_2_O, 50:50 → 71:29, v/v, 25 min, 3.0 mL/min, Sinochrom ODS-BP, 10 mm × 250 mm, 5 μm) to yield compound **1** (6.0 mg, t_R_ = 23.75 min).

### Compound characterization

#### *Peniexpansones A* (1)

Yellow oil. $[\alpha]_\mathrm{D}^{25}$ −71.2 (*c* 0.13, MeOH). UV (MeOH) λ_max_ (log ε) 300 (3.17) nm. IR (KBr): *υ*_max_ 3484, 2959, 1741, 1684, 1596, 1449, 1371, 1266, 1084, 739 cm^−1^. ^1^H NMR (600 MHz in CDCl_3_) and ^13^C NMR (150 MHz in CDCl_3_) data, see Table 1. HRESIMS *m/z* 481.1856 [M + Na]⁺ (calcd for C_25_H_30_O_8_Na^+^, 481.1833).

#### *Peniexpansones B* (2)

Yellow oil. $[\alpha]_\mathrm{D}^{25}$ −10.47 (*c* 0.09, MeOH). UV (MeOH) λ_max_ (log ε) 310 (3.51) nm. IR (KBr): *υ*_max_ 3434, 1704, 1614, 1302, 1094, 894 cm^−1^. ^1^H NMR (600 MHz in CDCl_3_) and ^13^C NMR (150 MHz in CDCl_3_) data, see Table 1. HRESIMS *m/z* 431.1721 [M + H]⁺ (calcd for C_23_H_27_O_8_^+^, 431.1700).

#### *Peniexpansones C* (3)

Yellow oil. $[\alpha]_\mathrm{D}^{25}$ −57.61 (*c* 0.09, MeOH). UV (MeOH) λ_max_ (log ε) 250 (3.05) nm, 315 (3.08) nm. IR (KBr): *υ*_max_ 3436, 2964, 1697, 1461, 1291, 1082, 1008, 890, 740 cm^−1^. ^1^H NMR (600 MHz in CDCl_3_) and ^13^C NMR (150 MHz in CDCl_3_) data, see Table 1. HRESIMS *m/z* 443.1713 [M + H]⁺ (calcd for C_24_H_27_O_8_^+^, 443.1700).

#### *Peniexpansones D* (4)

Yellow oil. $[\alpha]_\mathrm{D}^{25}$ −79.97 (*c* 0.57, MeOH). UV (MeOH) λ_max_ (log ε) 288 (3.34) nm. IR (KBr): *υ*_max_ 3428, 2966, 1692, 1604, 1464, 1320, 1096, 867 cm^−1^. ^1^H NMR (600 MHz in CDCl_3_) and ^13^C NMR (150 MHz in CDCl_3_) data, see Table 1. HRESIMS *m/z* 413.1976 [M + H]⁺ (calcd for C_24_H_29_O_6_^+^, 413.1959).

#### (2*E*,4*E*,6*E*)-8-methyldeca-2,4,6-trienoic acid (5)

Yellow oil. $[\alpha]_\mathrm{D}^{25}$ +3.77 (*c* 0.35, MeOH). UV (MeOH) λ_max_ (log ε) 258 (2.72) nm. IR (KBr): *υ*_max_ 3434, 2960, 1636, 1384, 498 cm^−1^. ^1^H NMR (600 MHz in CDCl_3_) and ^13^C NMR (150 MHz in CDCl_3_) data. HRESIMS *m/z* 181.1223 [M + H]⁺ (calcd for C_11_H_17_O_2_^+^, 181.1223). ^1^H NMR (600 MHz, CDCl_3_) δ 7.39 (dd, 1H, H-3), 6.58 (dd, 1H, H-5), 6.26 (dd, 1H, H-4), 6.12 (dd, 1H, H-6) 5.87, (ov, 1H, H-7), 5.85 (ov, 1H, H-2), 2.14 (m, 1H, H-8), 1.36 (m, 2H, H-9), 1.02 (d, 3H, Me-11) 0.87 (t, 3H, Me-10); ^13^C NMR (150 MHz, CDCl_3_) δ 172.4 (C-1), 147.4 (C-3), 147.2 (C-7), 142.7 (C-5), 128.1 (C-6), 127.6 (C-4), 118.8 (C-2), 38.8 (C-8), 29.5 (C-9), 19.7 (C-11), 11.7 (C-10).

#### *Peniexpansones E* (6)

Yellow oil. $[\alpha]_\mathrm{D}^{25}$ +93.33 (c 0.06, MeOH). UV (MeOH) λ_max_ (log ε) 210 (3.44) nm. ^1^H NMR (600 MHz in CDCl_3_) and ^13^C NMR (150 MHz in CDCl_3_) data, see Table 2. HRESIMS *m/z* 229.0835 [M + Na]⁺ (calcd for C_12_H_14_O_3_Na^+^, 229.0835).

#### *Peniexpansones F* (7)

Yellow oil. $[\alpha]_\mathrm{D}^{25}$ +25.37 (*c* 0.07, MeOH). UV (MeOH) λ_max_ (log ε) 210 (3.17) nm. ^1^H NMR (600 MHz in CDCl_3_) and ^13^C NMR (150 MHz in CDCl_3_) data, see Table 2. HRESIMS *m/z* 229.0809 [M + Na]⁺ (calcd for C_12_H_14_O_3_Na^+^, 229.0835).

### Quantum chemical calculation

Crest [19] was used to search the conformational space of candidate structures at the GFNFF [20] level of theory, followed by optimization at the GFN2-xTB [21] level with a 4 kcal mol⁻1 energy window to discard high-energy conformers. Each conformer was then optimized and subjected to frequency analysis at the B3LYP-D3(BJ)/TZVP(IEFPCM) level of theory. ECD calculations for compounds **1**–**4**, **6** and **7** were carried out at the B3LYP/TZVP (IEFPCM, methanol) level. DFT-GIAO ^13^C NMR shielding tensors were computed at the ωB97X-D/6-31G*(IEFPCM, chloroform) level, consistent with the reported STS protocol [14]. The calculated shielding tensors and ECD curves of all conformers were Boltzmann-averaged based on Gibbs free energies. The ECD spectra of compounds **1**, **2**, **6**, and **7** were simulated using SpecDis v1.71 [22] with a sigma/gamma value of 0.35 eV, while values of 0.28 eV and 0.45 eV were applied for compounds **3** and **4**, respectively. All DFT calculations were performed using the Gaussian 16 [23] software package. The DFT optimized geometry data, relative energies, and conformational populations of all optimized structures are provided in the Supporting Information. The 3D structures for compounds **6** and **7** were rendered with VMD 1.9.3 [24]. Molecular orbitals were computed with Multiwfn 3.8 (dev) [25] and subsequently visualized using VMD 1.9.3 [24].

### Assay of antimicrobial activity

The antimicrobial activities of all compounds were evaluated against a panel of bacterial and fungal strains, including *Bacillus subtilis* ATCC 6051, *Escherichia coli* DH5α, *Staphylococcus aureus* ATCC 25923, *Acinetobacter baumannii* ATCC 19606, *Pseudomonas aeruginosa* ATCC 27853, *Saccharomyces cerevisiae* ATCC 9763, and *Candida albicans* ATCC 10231. Each sample was dissolved in 80% aqueous ethanol to achieve an initial concentration of 2 mM, followed by dilution to a final concentration of 7.5 × 10^5^ CFU/mL. Bacterial suspensions were dispensed into individual wells of a 96-well culture plate. The plate was incubated at 37 °C for 48 h, after which microbial growth inhibition was assessed visually based on the absence of turbidity. The experimental design included medium blanks, bacterial controls, streptomycin as a positive control for bacteria, and terbinafine and fluconazole as positive controls for fungi.

The ability of the compounds to potentiate fluconazole against the resistant *C. albicans* was assessed using a broth microdilution method. Fixed concentrations of fluconazole were combined with serial dilutions of the test compounds in a 96-well plate. The fungal suspension was prepared and added as described previously. After incubation at 37 °C for 48 h, the minimum inhibitory concentration (MIC) of the combinations was determined visually by the absence of turbidity. The effective combinations are reported in Table 3.

**Table 3:** Compound-mediated potentiation of fluconazole against resistant *C. albicans*

Compound	Fluconazole MIC (*μ*M) in combination with compound at (*μ*M)			
	0	10	20	40
**1**	> 4000	> 4000	25	10
**2**		> 4000	> 4000	100
**3**		> 4000	> 2000	500
**4**		> 4000	> 4000	800

*MIC* minimum inhibitory concentration.

The intrinsic antifungal activity of fluconazole against this strain was > 4 mM, confirming its resistant phenotype [18]. The activities of the compounds alone were as follows: Compounds **1**, **2**, and **4** were inactive (MIC > 50 μM); Compound **3** showed moderate activity (MIC = 50 μM).

### Cytotoxicity assay

The cytotoxicity was evaluated against the human gastric epithelial cell line GES-1, human colorectal cancer cell line HCT116, mouse breast cancer cell line 4T1, and highly metastatic liver cancer cell line MHCC97H. The assay was performed using the CCK-8 method, with doxorubicin serving as a positive control. Cells were cultured in DMEM supplemented with 10% fetal bovine serum and 1% penicillin–streptomycin, and maintained in a humidified atmosphere at 37 °C with 5% CO_2_. For the assay, cells were seeded into 96-well plates at a density of 10,000 cells per well and incubated for 24 h. Subsequently, the cells were exposed to the test compounds at varying concentrations ≤ 50 μM, prepared in culture medium, for a duration of 48 h. Alongside the experimental groups, blank controls (wells with CCK-8 solution but no cells) and negative controls (wells with vehicle) were also included. After the treatment, 10 μL of CCK-8 reagent was added to each well and incubated for 1 h. The optical density was then measured at 450 nm using a microplate reader (Feyond-A300, allsheng, Hangzhou, China). The IC_50_ values, indicative of the concentration of the compounds required to inhibit cell growth by 50%, were calculated using the GraphPad Prism 10.6.0 software.

## Conclusions

In conclusion, the chemical investigation of *P. expansum* DWS880 led to the identification of six new polyketides, designated peniexpansones A–F. Among them, peniexpansones A–D (**1**–**4**) possess a highly oxygenated tetrahydronaphthalene unit connected to an acyl chain bearing a stereogenic carbon at C-8′, representing a unique substitution pattern. In biological evaluations, compound **3** exhibited weak cytotoxicity against the HCT116 cell line. Notably, it also demonstrated antimicrobial activity against *Staphylococcus aureus*, *Acinetobacter baumannii*, *Saccharomyces cerevisiae*, and *Candida albicans*. More importantly, compounds **1** and **2** showed no intrinsic antifungal activity but functioned as strong and selective potentiators of fluconazole against resistant *C. albicans*, while exhibiting minimal cytotoxicity against human normal cell lines, marking them as exceptionally promising candidates for combination therapy. These findings enhance the structural and bioactive diversity known among fungal polyketides.

## Supplementary Information

Supplementary material 1.

## Data Availability

All data generated or analyzed during this study are included in this published article and its supplementary information files.
