# Supplementary material for: Peniexpansones A–F: polyketides from Penicillium expansum DWS880 capable of enhancing the activity of fluconazole against Candida albicans
Source: Nat Prod Bioprospect. 2026 Mar 3;16(1):40. doi: 10.1007/s13659-026-00592-5 (PMC12953831; doi:10.1007/s13659-026-00592-5)
Supplement: Supplementary file 1 — Supplementary material 1. [file 13659_2026_592_MOESM1_ESM.docx]

# Supplementary Material

**Peniexpansones A–F: Polyketides from *Penicillium expansum* DWS880 capable of enhancing the activity of fluconazole against *Candida albicans***

Wen-Yu Lu ^1^, Qing-Hui Xiao ^2^, Ai-Lin Liang ^1^, Peng-Ju Xu ^1^, Jing Li^1,2,3^* and Wen-Xuan Wang^1,3^*

^1^ Xiangya School of Pharmaceutical Sciences, Central South University, Changsha, Hunan 410008, PR China.

^2^ Department of Pharmacy, National Clinical Research Center for Geriatric Disorder, Xiangya Hospital, Central South University, Changsha, Hunan 410008, PR China.

^3^ Hunan Prima Drug Research Center Co., Ltd, Hunan Research Center for Drug Safety Evaluation, Hunan Key Laboratory of Pharmacodynamics and Safety Evaluation of New Drugs, Changsha, Hunan 410331, PR China

Contents

[Fig. S1.](#_Toc13435) ^[1](#_Toc13435)^[H NMR spectrum of](#_Toc13435) **[1](#_Toc13435)** [(600 MHz, CDCl](#_Toc13435)_[3](#_Toc13435)_[) 5](#_Toc13435)

[Fig. S2.](#_Toc27758) ^[13](#_Toc27758)^[C NMR and DEPT spectrum of](#_Toc27758) **[1](#_Toc27758)** [(150 MHz, CDCl](#_Toc27758)_[3](#_Toc27758)_[) 5](#_Toc27758)

[Fig. S3.](#_Toc32013)^[1](#_Toc32013)^[H-](#_Toc32013)^[1](#_Toc32013)^[H COSY spectrum of](#_Toc32013) **[1](#_Toc32013)** [6](#_Toc32013)

[Fig. S4. HSQC spectrum of](#_Toc18491) **[1](#_Toc18491)** [6](#_Toc18491)

[Fig. S5. HMBC spectrum of](#_Toc15422) **[1](#_Toc15422)** [6](#_Toc15422)

[Fig. S6. NOESY spectrum of](#_Toc14716) **[1](#_Toc14716)** [7](#_Toc14716)

[Fig. S7. 1D NOE (CDCl](#_Toc26655)_[3](#_Toc26655)_[) spectrum of compound](#_Toc26655) **[1](#_Toc26655)** [8](#_Toc26655)

[Fig. S8. HR-ESI-MS spectrum of](#_Toc4753) **[1](#_Toc4753)** [8](#_Toc4753)

[Fig. S9. IR spectra of](#_Toc13268) **[1](#_Toc13268)** [8](#_Toc13268)

[Fig. S10. UV spectrum (methanol) of](#_Toc25395) **[1](#_Toc25395)** [9](#_Toc25395)

[Fig. S11.](#_Toc31955) ^[1](#_Toc31955)^[H NMR spectrum of](#_Toc31955) **[2](#_Toc31955)** [(600 MHz, CDCl](#_Toc31955)_[3](#_Toc31955)_[) 9](#_Toc31955)

[Fig. S12.](#_Toc21805) ^[13](#_Toc21805)^[C NMR and DEPT spectrum of](#_Toc21805) **[2](#_Toc21805)** [(150 MHz, CDCl](#_Toc21805)_[3](#_Toc21805)_[) 9](#_Toc21805)

[Fig. S13.](#_Toc18207)^[1](#_Toc18207)^[H-](#_Toc18207)^[1](#_Toc18207)^[H COSY spectrum of](#_Toc18207) **[2](#_Toc18207)** [10](#_Toc18207)

[Fig. S14. HSQC spectrum of](#_Toc60) **[2](#_Toc60)** [11](#_Toc60)

[Fig. S15. HMBC spectrum of](#_Toc26685) **[2](#_Toc26685)** [11](#_Toc26685)

[Fig. S16. NOESY spectrum of](#_Toc30111) **[2](#_Toc30111)** [12](#_Toc30111)

[Fig. S17. HR-ESI-MS spectrum of](#_Toc16123) **[2](#_Toc16123)** [12](#_Toc16123)

[Fig. S18. IR spectra of](#_Toc2386) **[2](#_Toc2386)** [12](#_Toc2386)

[Fig. S19. UV spectrum (methanol) of](#_Toc1598) **[2](#_Toc1598)** [13](#_Toc1598)

[Fig. S20.](#_Toc19988) ^[1](#_Toc19988)^[H NMR spectrum of](#_Toc19988) **[3](#_Toc19988)** [(600 MHz, CDCl](#_Toc19988)_[3](#_Toc19988)_[) 13](#_Toc19988)

[Fig. S21.](#_Toc883) ^[13](#_Toc883)^[C NMR and DEPT spectrum of](#_Toc883) **[3](#_Toc883)** [(150 MHz, CDCl](#_Toc883)_[3](#_Toc883)_[) 14](#_Toc883)

[Fig. S22.](#_Toc8868)^[1](#_Toc8868)^[H-](#_Toc8868)^[1](#_Toc8868)^[H COSY spectrum of](#_Toc8868) **[3](#_Toc8868)** [14](#_Toc8868)

[Fig. S23. HSQC spectrum of](#_Toc5464) **[3](#_Toc5464)** [15](#_Toc5464)

[Fig. S24. HMBC spectrum of](#_Toc25047) **[3](#_Toc25047)** [15](#_Toc25047)

[Fig. S25. NOESY spectrum of](#_Toc3637) **[3](#_Toc3637)** [16](#_Toc3637)

[Fig. S26. HR-ESI-MS spectrum of](#_Toc5242) **[3](#_Toc5242)** [16](#_Toc5242)

[Fig. S27. IR spectra of](#_Toc23744) **[3](#_Toc23744)** [16](#_Toc23744)

[Fig. S28. UV spectrum (methanol) of](#_Toc15128) **[3](#_Toc15128)** [17](#_Toc15128)

[Fig. S29.](#_Toc24899) ^[1](#_Toc24899)^[H NMR spectrum of](#_Toc24899) **[4](#_Toc24899)** [(600 MHz, CDCl](#_Toc24899)_[3](#_Toc24899)_[) 17](#_Toc24899)

[Fig. S30.](#_Toc14108) ^[13](#_Toc14108)^[C NMR and DEPT spectrum of](#_Toc14108) **[4](#_Toc14108)** [(150 MHz, CDCl](#_Toc14108)_[3](#_Toc14108)_[) 18](#_Toc14108)

[Fig. S31.](#_Toc30901)^[1](#_Toc30901)^[H-](#_Toc30901)^[1](#_Toc30901)^[H COSY spectrum of](#_Toc30901) **[4](#_Toc30901)** [18](#_Toc30901)

[Fig. S32. HSQC spectrum of](#_Toc21997) **[4](#_Toc21997)** [19](#_Toc21997)

[Fig. S33. HMBC spectrum of](#_Toc21996) **[4](#_Toc21996)** [19](#_Toc21996)

[Fig. S34. NOESY spectrum of](#_Toc22000) **[4](#_Toc22000)** [20](#_Toc22000)

[Fig. S35. 1D NOE (CDCl](#_Toc16657)_[3](#_Toc16657)_[) spectrum of compound](#_Toc16657) **[4](#_Toc16657)** [20](#_Toc16657)

[Fig. S36. HR-ESI-MS spectrum of](#_Toc15510) **[4](#_Toc15510)** [21](#_Toc15510)

[Fig. S37. IR spectra of](#_Toc22523) **[4](#_Toc22523)** [21](#_Toc22523)

[Fig. S38. UV spectrum (methanol) of](#_Toc19938) **[4](#_Toc19938)** [21](#_Toc19938)

[Fig. S39.](#_Toc32286) ^[1](#_Toc32286)^[H NMR spectrum of](#_Toc32286) **[5](#_Toc32286)** [(600 MHz, CDCl](#_Toc32286)_[3](#_Toc32286)_[) 22](#_Toc32286)

[Fig. S40.](#_Toc14597) ^[13](#_Toc14597)^[C NMR and DEPT spectrum of](#_Toc14597) **[5](#_Toc14597)** [(150 MHz, CDCl](#_Toc14597)_[3](#_Toc14597)_[) 22](#_Toc14597)

[Fig. S41.](#_Toc24878)^[1](#_Toc24878)^[H-](#_Toc24878)^[1](#_Toc24878)^[H COSY spectrum of](#_Toc24878) **[5](#_Toc24878)** [23](#_Toc24878)

[Fig. S42. HSQC spectrum of](#_Toc27400) **[5](#_Toc27400)** [23](#_Toc27400)

[Fig. S43. HMBC spectrum of](#_Toc24490) **[5](#_Toc24490)** [24](#_Toc24490)

[Fig. S44. HR-ESI-MS spectrum of](#_Toc16286) **[5](#_Toc16286)** [24](#_Toc16286)

[Fig. S45. IR spectra of](#_Toc18665) **[5](#_Toc18665)** [24](#_Toc18665)

[Fig. S46. UV spectrum (methanol) of](#_Toc18822) **[5](#_Toc18822)** [25](#_Toc18822)

[Fig. S47.](#_Toc27230) ^[1](#_Toc27230)^[H NMR spectrum of](#_Toc27230) **[6](#_Toc27230)** [(600 MHz, CDCl](#_Toc27230)_[3](#_Toc27230)_[) 25](#_Toc27230)

[Fig. S48.](#_Toc30523) ^[13](#_Toc30523)^[C NMR and DEPT spectrum of](#_Toc30523) **[6](#_Toc30523)** [(150 MHz, CDCl](#_Toc30523)_[3](#_Toc30523)_[) 26](#_Toc30523)

[Fig. S49.](#_Toc15404) ^[1](#_Toc15404)^[H-](#_Toc15404)^[1](#_Toc15404)^[H COSY spectrum of](#_Toc15404) **[6](#_Toc15404)** [26](#_Toc15404)

[Fig. S50. HSQC spectrum of](#_Toc15792) **[6](#_Toc15792)** [27](#_Toc15792)

[Fig. S51. HMBC spectrum of](#_Toc20299) **[6](#_Toc20299)** [27](#_Toc20299)

[Fig. S52. NOESY spectrum of](#_Toc627) **[6](#_Toc627)** [28](#_Toc627)

[Fig. S53. HR-ESI-MS spectrum of](#_Toc29123) **[6](#_Toc29123)** [28](#_Toc29123)

[Fig. S54. UV spectrum (methanol) of](#_Toc1903) **[6](#_Toc1903)** [28](#_Toc1903)

[Fig. S55.](#_Toc25030) ^[1](#_Toc25030)^[H NMR spectrum of](#_Toc25030) **[7](#_Toc25030)** [(600 MHz, CDCl](#_Toc25030)_[3](#_Toc25030)_[) 29](#_Toc25030)

[Fig. S56.](#_Toc28188) ^[13](#_Toc28188)^[C NMR and DEPT spectrum of](#_Toc28188) **[7](#_Toc28188)** [(150 MHz, CDCl](#_Toc28188)_[3](#_Toc28188)_[) 29](#_Toc28188)

[Fig. S57.](#_Toc28348) ^[1](#_Toc28348)^[H-](#_Toc28348)^[1](#_Toc28348)^[H COSY spectrum of](#_Toc28348) **[7](#_Toc28348)** [30](#_Toc28348)

[Fig. S58. HSQC spectrum of](#_Toc21734) **[7](#_Toc21734)** [30](#_Toc21734)

[Fig. S59. HMBC spectrum of](#_Toc8054) **[7](#_Toc8054)** [31](#_Toc8054)

[Fig. S60. NOESY spectrum of](#_Toc26894) **[7](#_Toc26894)** [31](#_Toc26894)

[Fig. S61. HR-ESI-MS spectrum of](#_Toc28446) **[7](#_Toc28446)** [32](#_Toc28446)

[Fig. S62. UV spectrum (methanol) of](#_Toc22613) **[7](#_Toc22613)** [32](#_Toc22613)

[Fig. S63.](#_Toc32427) ^[1](#_Toc32427)^[H NMR spectrum of](#_Toc32427) **[8](#_Toc32427)** [(600 MHz, CDCl](#_Toc32427)_[3](#_Toc32427)_[) 32](#_Toc32427)

[Fig. S64.](#_Toc10538) ^[13](#_Toc10538)^[C NMR and DEPT spectrum of](#_Toc10538) **[8](#_Toc10538)** [(150 MHz, CDCl](#_Toc10538)_[3](#_Toc10538)_[) 33](#_Toc10538)

[Fig. S65. HMBC spectrum of](#_Toc18136) **[8](#_Toc18136)** [33](#_Toc18136)

[Fig. S66. HR-ESI-MS spectrum of](#_Toc30013) **[8](#_Toc30013)** [34](#_Toc30013)

[Table S67. Experimental ¹³C NMR chemical shifts (CDCl₃) of](#_Toc30449) **[2](#_Toc30449)** [and the calculated values for diastereoisomers](#_Toc30449) **[2a](#_Toc30449)**[,](#_Toc30449) **[2a'](#_Toc30449)**[,](#_Toc30449) **[2b](#_Toc30449)** [and](#_Toc30449) **[2b'](#_Toc30449)** [obtained by GIAO-STS ¹³C NMR calculations. 34](#_Toc30449)

[Table S68. Experimental ¹³C NMR chemical shifts (CDCl₃) of](#_Toc8603) **[3](#_Toc8603)** [and the calculated values for diastereoisomers](#_Toc8603) **[3a](#_Toc8603)**[,](#_Toc8603) **[3a'](#_Toc8603)**[,](#_Toc8603) **[3b](#_Toc8603)** [and](#_Toc8603) **[3b'](#_Toc8603)** [obtained by GIAO-STS ¹³C NMR calculations. 35](#_Toc8603)

[Table S69. Geometry data of conformers of structure](#_Toc20216) **[1a](#_Toc20216)** [35](#_Toc20216)

[Table S70. Geometry data of conformers of structure](#_Toc18629) **[1a'](#_Toc18629)** [53](#_Toc18629)

[Table S71. Geometry data of conformers of structure](#_Toc30694) **[2a](#_Toc30694)** [61](#_Toc30694)

[Table S72. Geometry data of conformers of structure](#_Toc28252) **[2a'](#_Toc28252)** [70](#_Toc28252)

[Table S73. Geometry data of conformers of structure](#_Toc13085) **[2b](#_Toc13085)** [83](#_Toc13085)

[Table S74. Geometry data of conformers of structure](#_Toc4444) **[2b'](#_Toc4444)** [95](#_Toc4444)

[Table S75. Geometry data of conformers of structure](#_Toc16968) **[3a](#_Toc16968)** [108](#_Toc16968)

[Table S76. Geometry data of conformers of structure](#_Toc6394) **[3a'](#_Toc6394)** [120](#_Toc6394)

[Table S77. Geometry data of conformers of structure](#_Toc13103) **[3b](#_Toc13103)** [136](#_Toc13103)

[Table S78. Geometry data of conformers of structure](#_Toc14752) **[3b'](#_Toc14752)** [155](#_Toc14752)

[Table S79. Geometry data of conformers of structure](#_Toc22354) **[4a](#_Toc22354)** [173](#_Toc22354)

[Table S80. Geometry data of conformers of structure](#_Toc6812) **[4a'](#_Toc6812)** [181](#_Toc6812)

[Table S81. The experimental ECD curve of](#_Toc11597) **[5](#_Toc11597)** [194](#_Toc11597)

[Table S82. Geometry data of conformers of compound](#_Toc2686) **[6](#_Toc2686)** [194](#_Toc2686)

[Table S83. Geometry data of conformers of compound](#_Toc29257) **[7](#_Toc29257)** [195](#_Toc29257)

[Table S84. The ITS sequences of](#_Toc16192) *[Penicillium expansum](#_Toc16192)* [DWS880 195](#_Toc16192)

[Fig. S85. Initial evaluation of fluconazole resistance in](#_Toc1031) *[Candida albicans.](#_Toc1031)*  [197](#_Toc1031)

[Fig. S86. Screening for fluconazole potentiators against resistant](#_Toc18587) *[Candida albicans](#_Toc18587)*[. 198](#_Toc18587)

Fig. S87. Synergy screening using fixed-ratio combinations................................................... 199

[Fig. S88. Detailed characterization of potentiation efficacy for Compound](#_Toc16466) **[1](#_Toc16466)**[. 200](#_Toc16466)

[Fig. S89. Detailed characterization of potentiation efficacy for Compound](#_Toc20039) **[2](#_Toc20039)** [201](#_Toc20039)

[Fig. S90. Detailed characterization of potentiation efficacy for Compound](#_Toc13518) **[3](#_Toc13518)** [202](#_Toc13518)

# Fig. S1. ^1^H NMR spectrum of 1 (600 MHz, CDCl_3_)


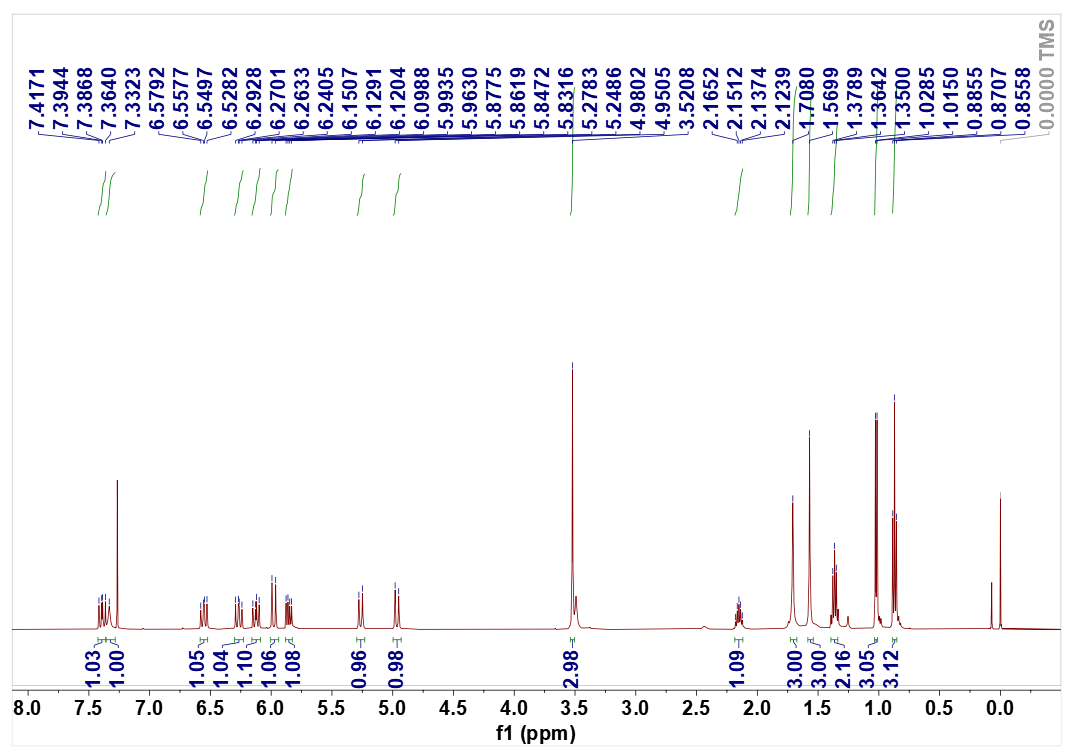


# Fig. S2. ^13^C NMR and DEPT spectrum of 1 (150 MHz, CDCl_3_)


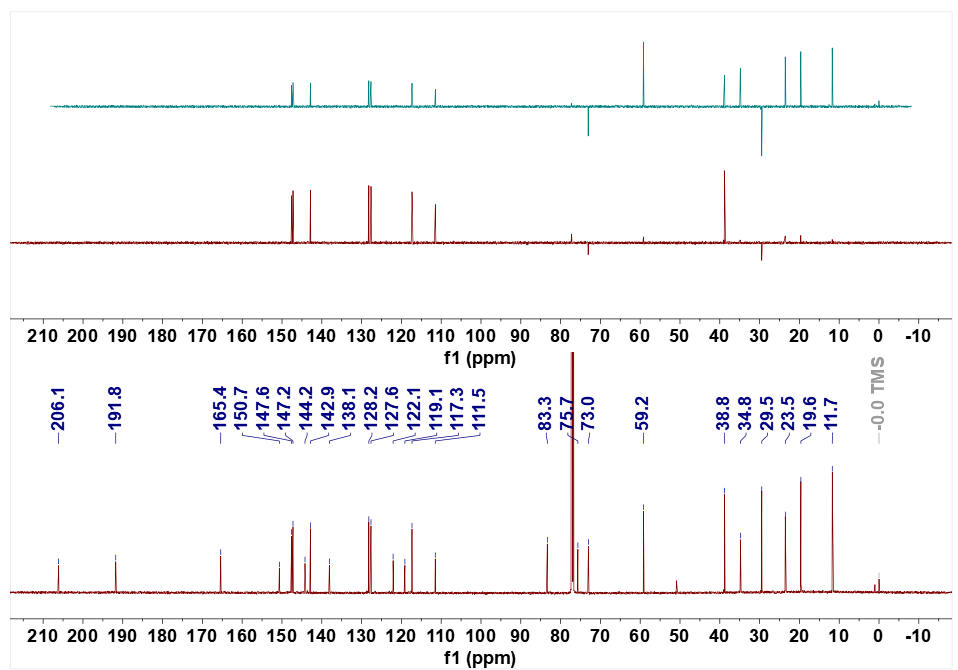


# Fig. S3.^1^H-^1^H COSY spectrum of 1


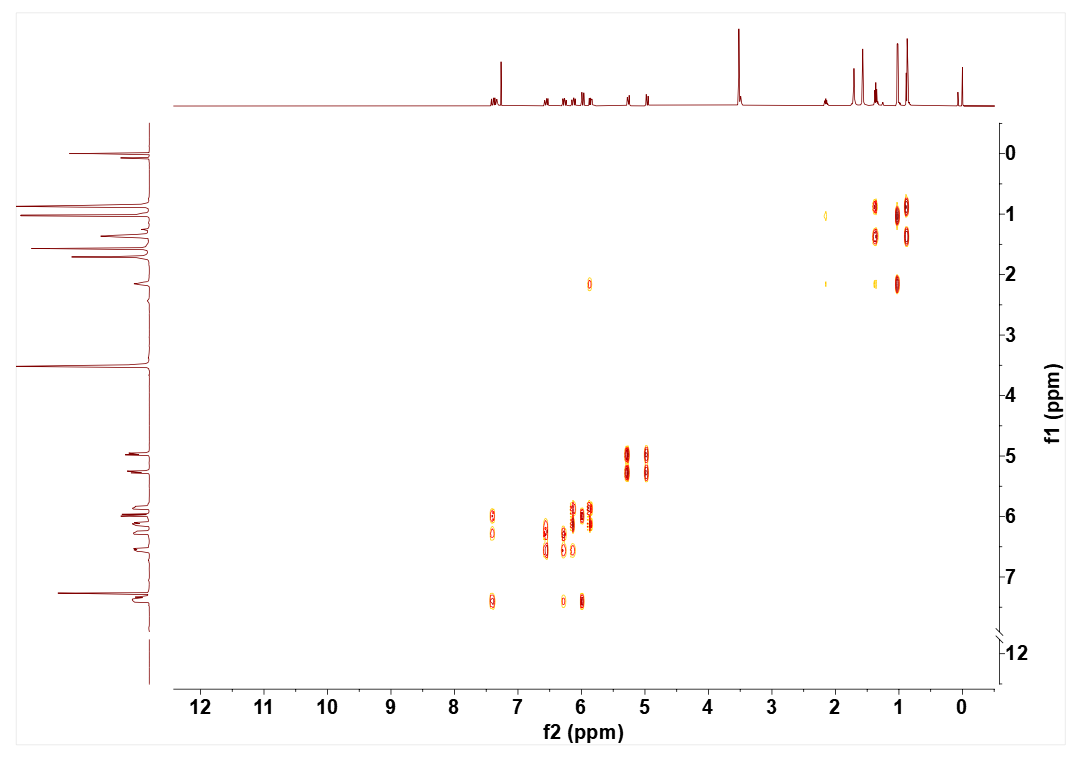


Fig. S4. HSQC spectrum of 1


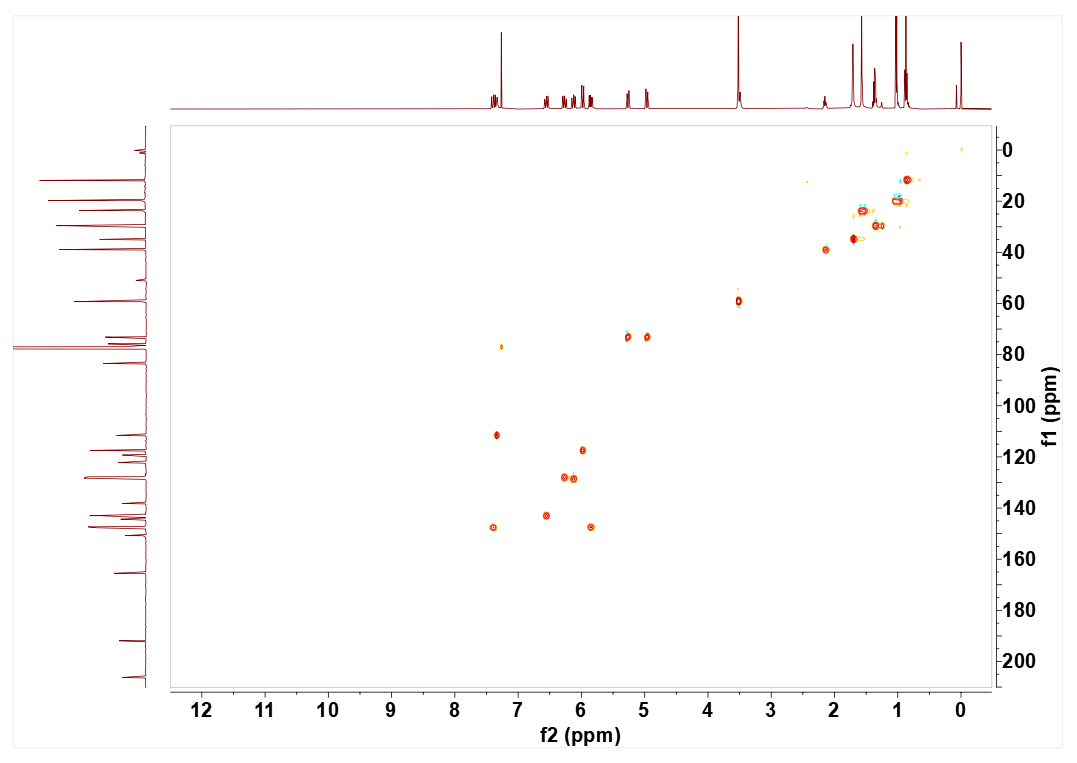


Fig. S5. HMBC spectrum of 1


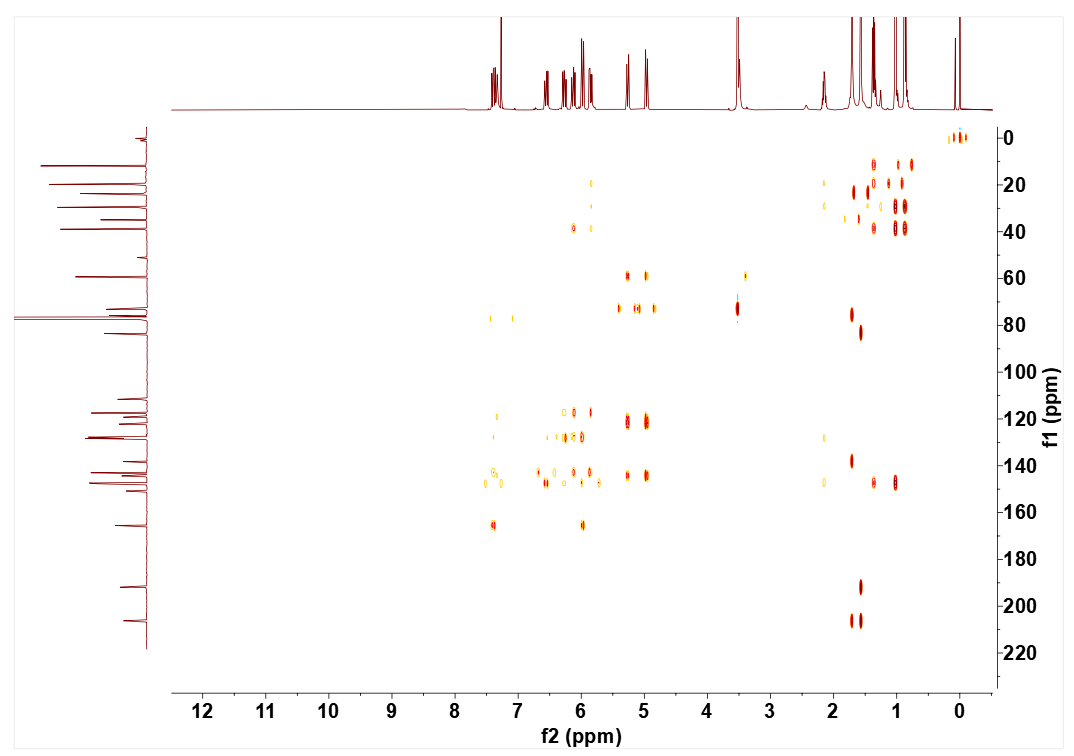


# Fig. S6. NOESY spectrum of 1


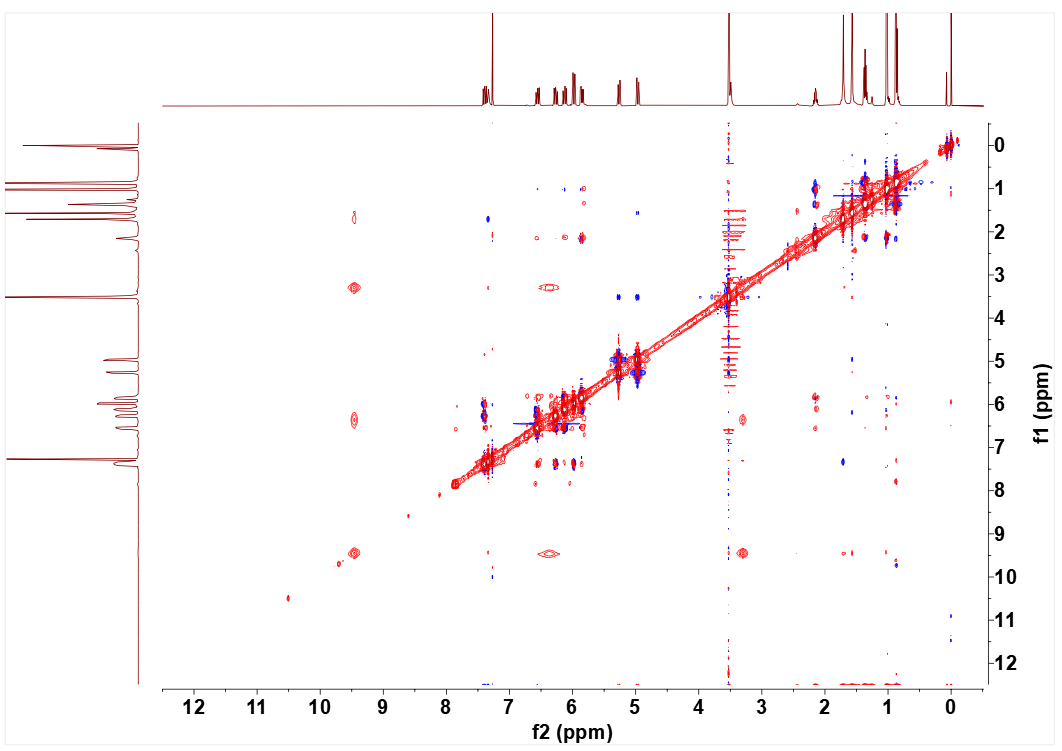


# Fig. S7. 1D NOE (CDCl_3_) spectrum of compound 1


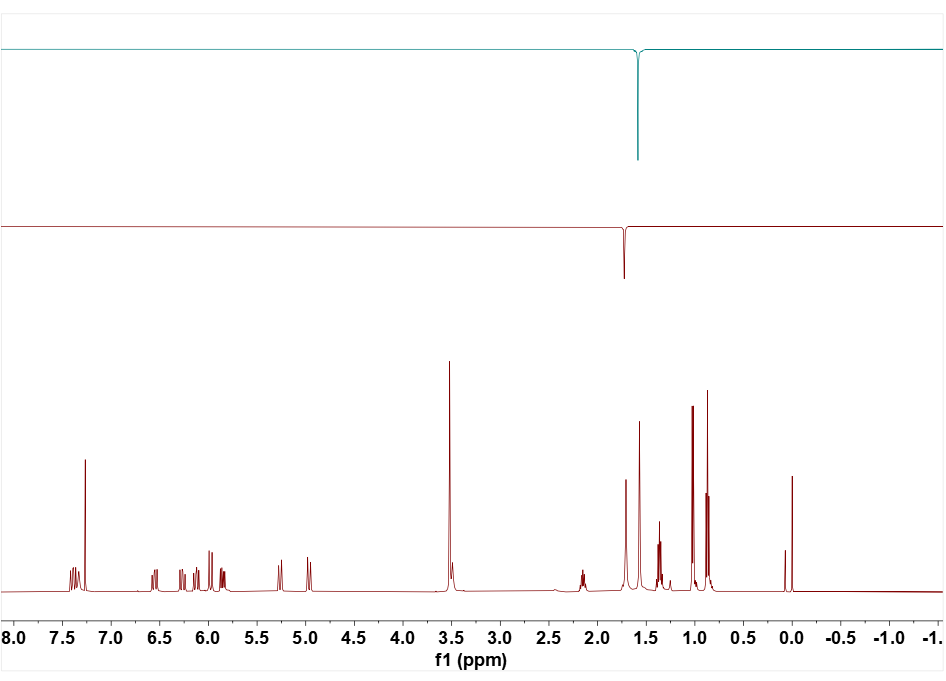


# Fig. S8. HR-ESI-MS spectrum of 1


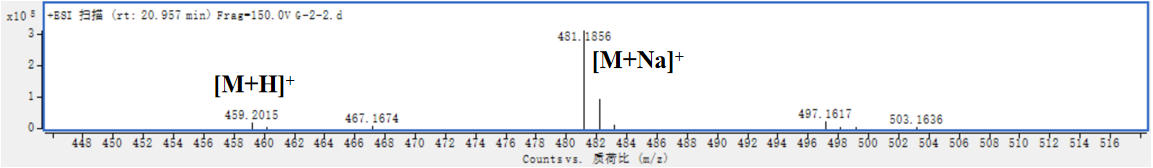


# Fig. S9. IR spectra of 1

# Fig. S10. UV spectrum (methanol) of 1


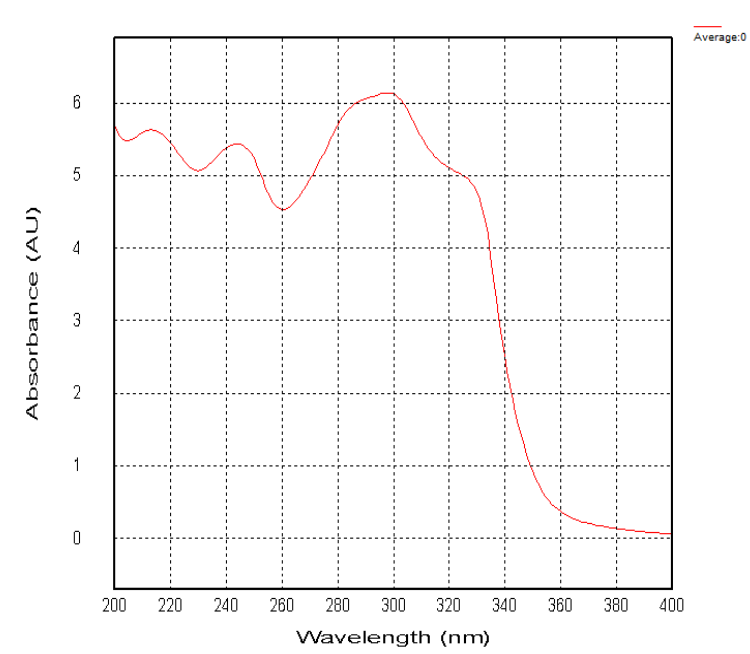


# Fig. S11. ^1^H NMR spectrum of 2 (600 MHz, CDCl_3_)


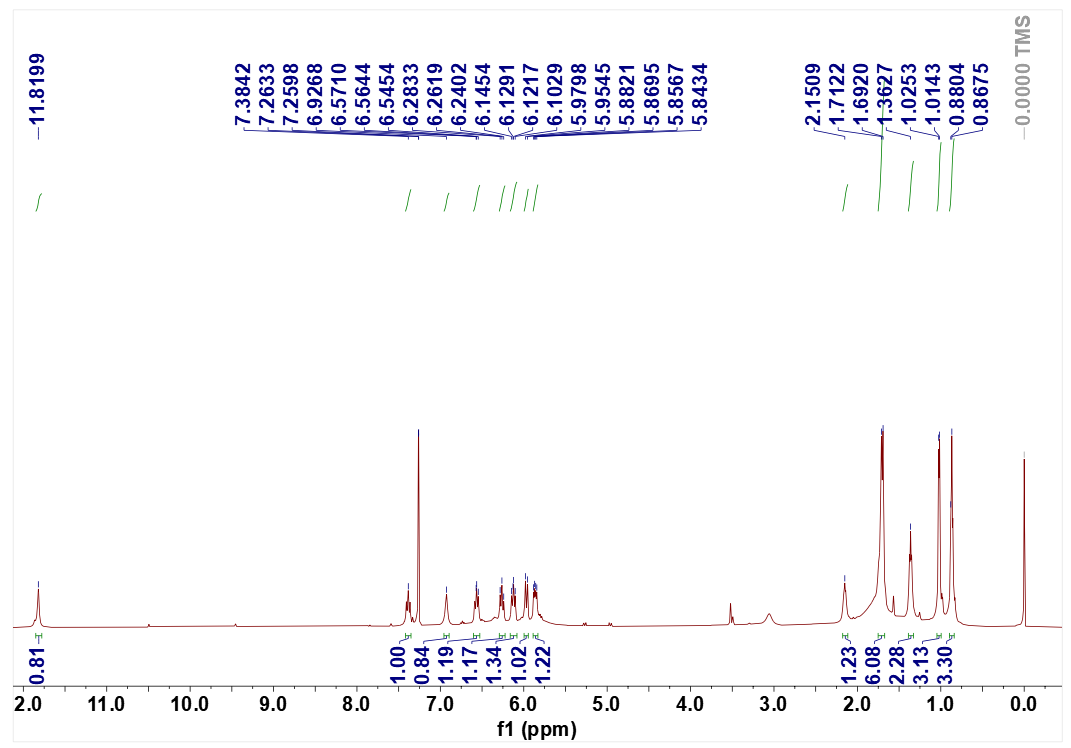


# Fig. S12. ^13^C NMR and DEPT spectrum of 2 (150 MHz, CDCl_3_)


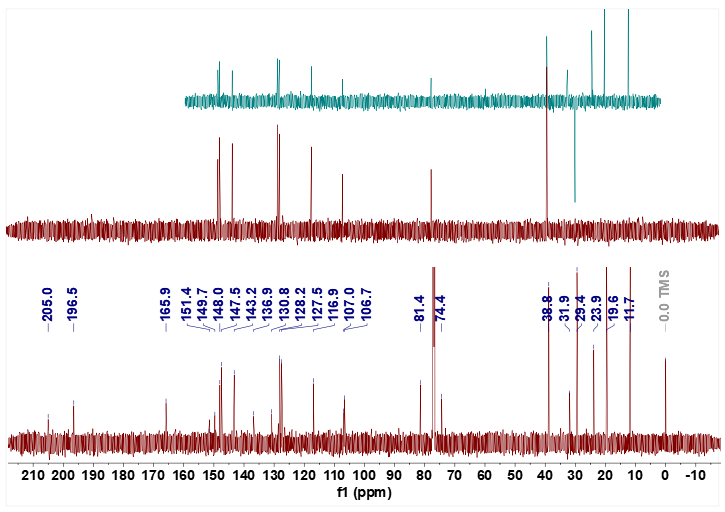


# Fig. S13.^1^H-^1^H COSY spectrum of 2


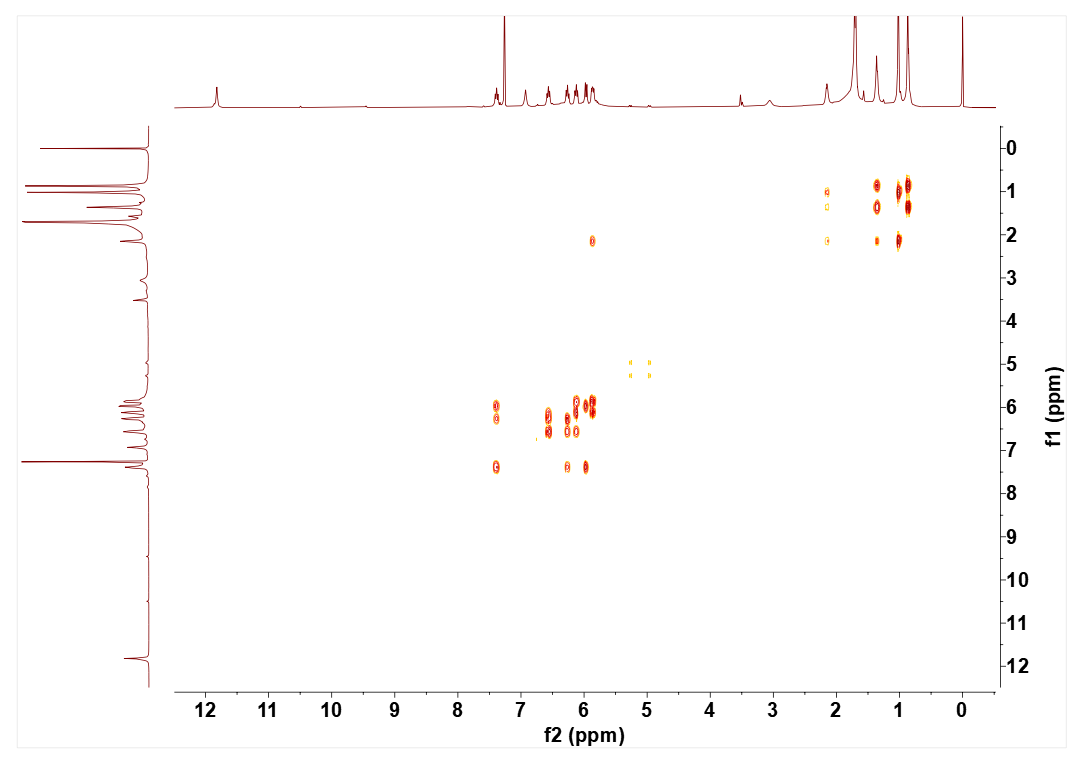


# **Fig. S14.** HSQC spectrum of **2**


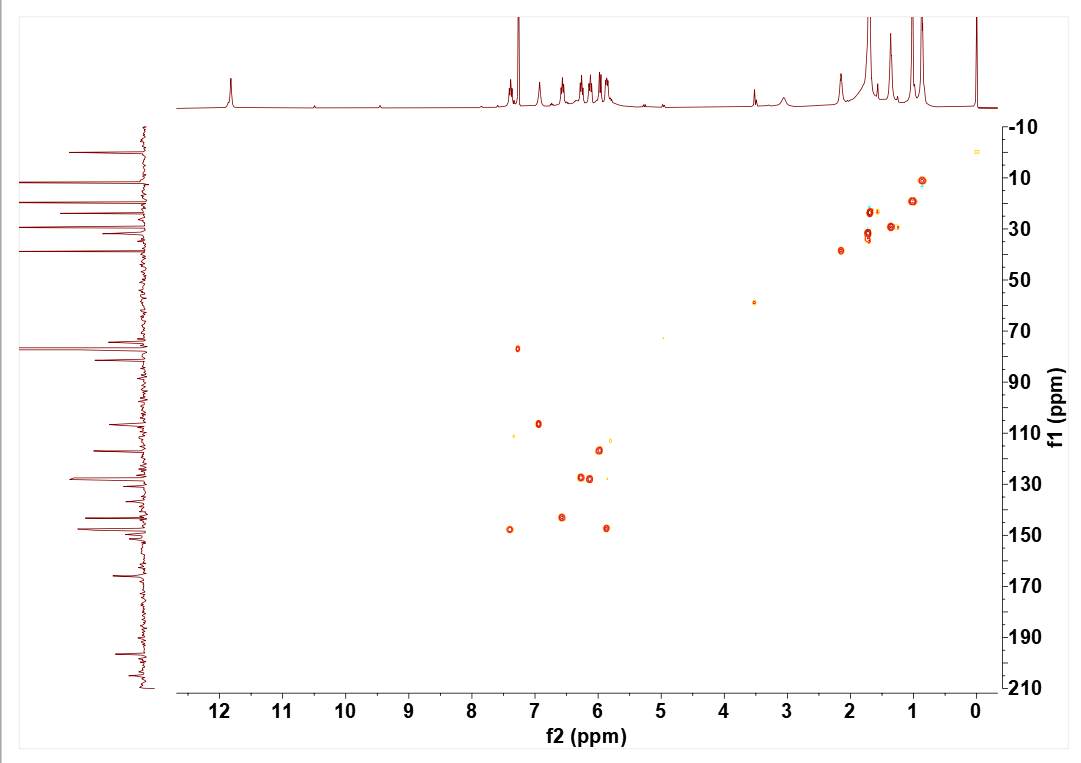


# **Fig. S15.** HMBC spectrum of **2**


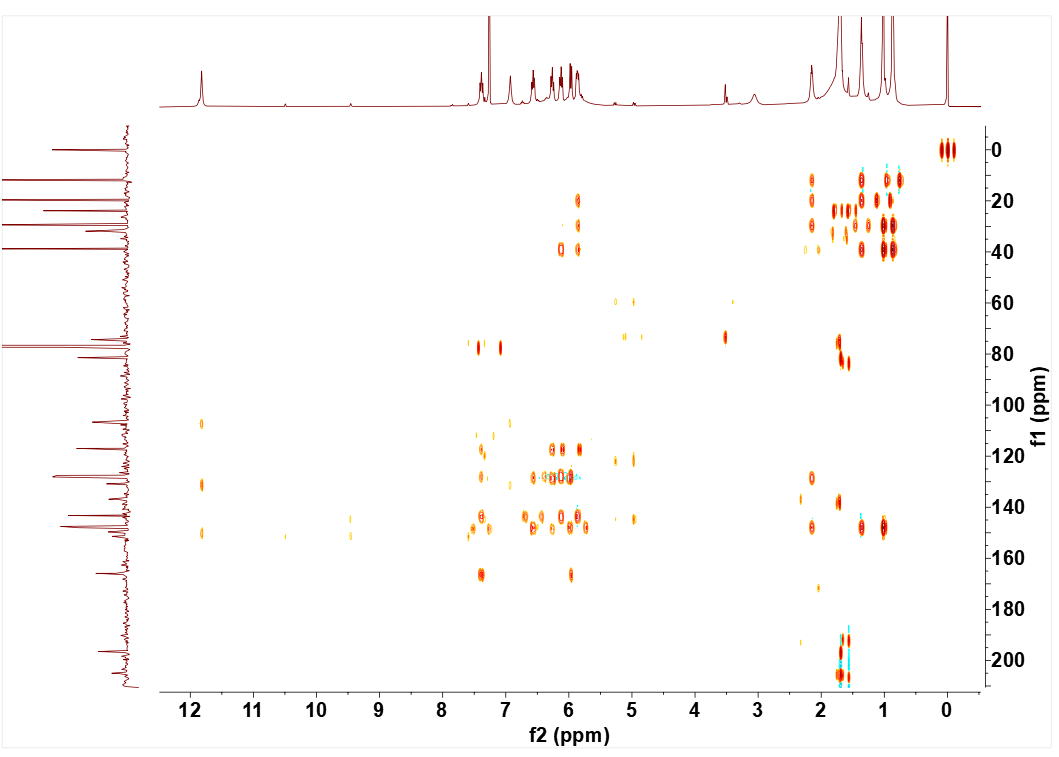


# Fig. S16. NOESY spectrum of 2


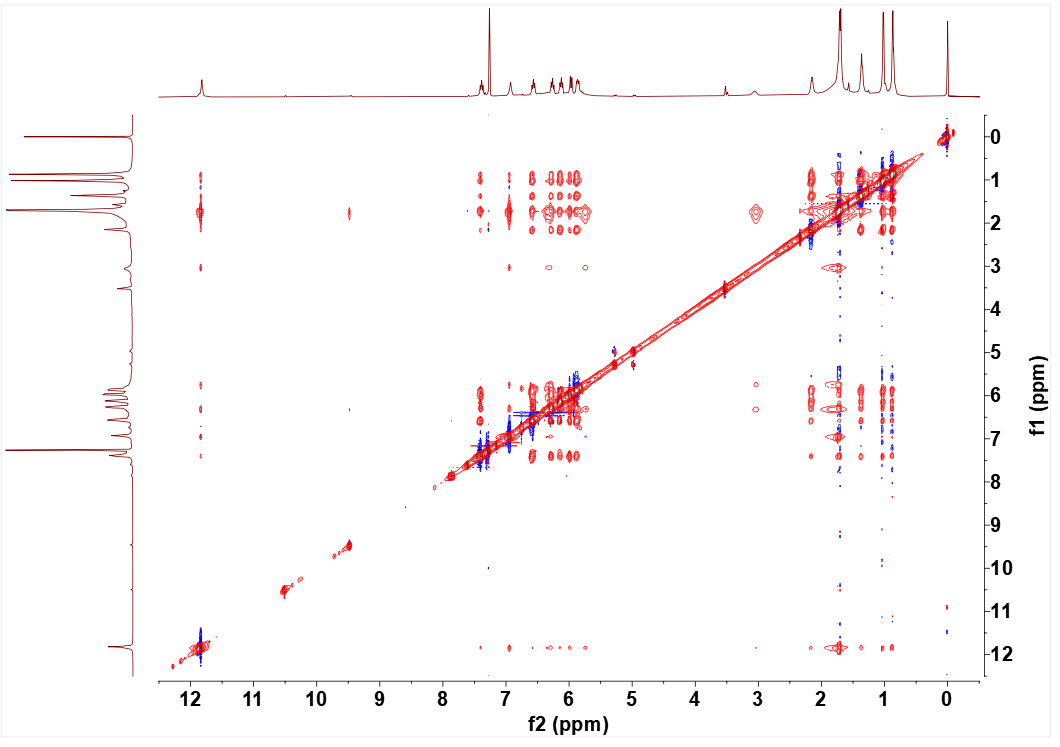


# Fig. S17. HR-ESI-MS spectrum of 2


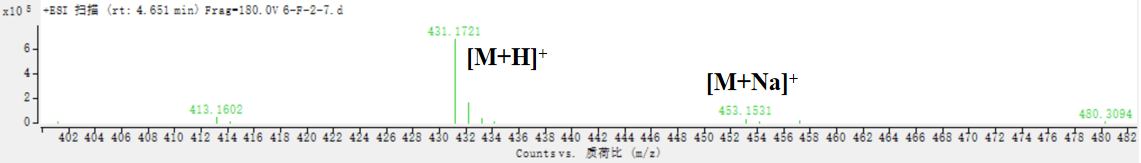


# Fig. S18. IR spectra of 2

# Fig. S19. UV spectrum (methanol) of 2


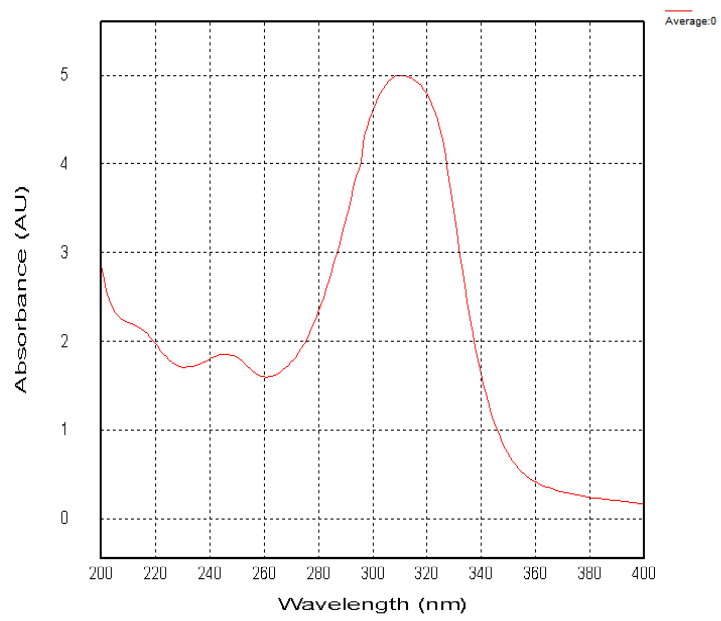


# Fig. S20. ^1^H NMR spectrum of 3 (600 MHz, CDCl_3_)


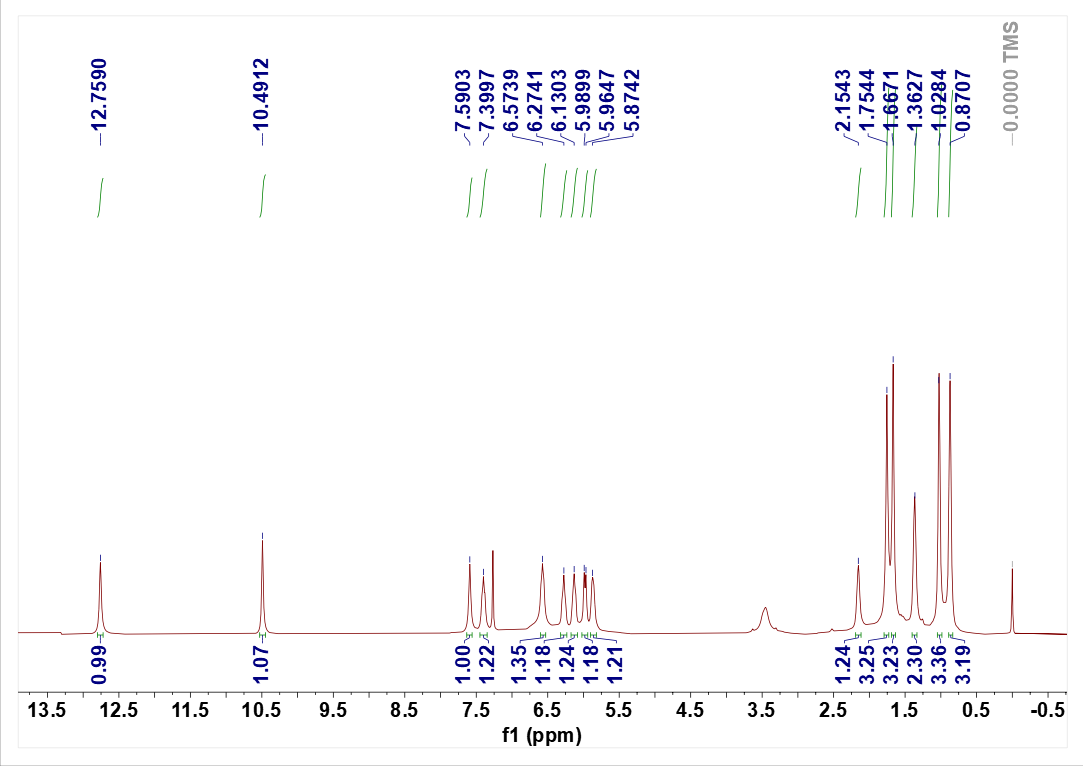


# Fig. S21. ^13^C NMR and DEPT spectrum of 3 (150 MHz, CDCl_3_)


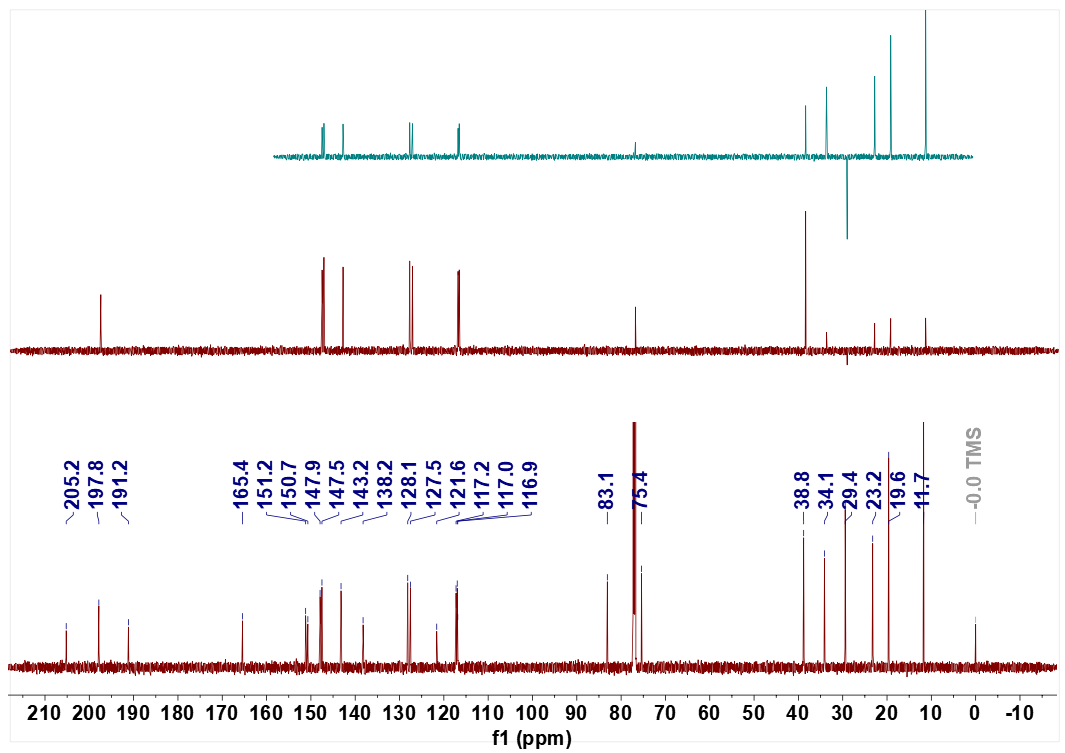


# Fig. S22.^1^H-^1^H COSY spectrum of 3


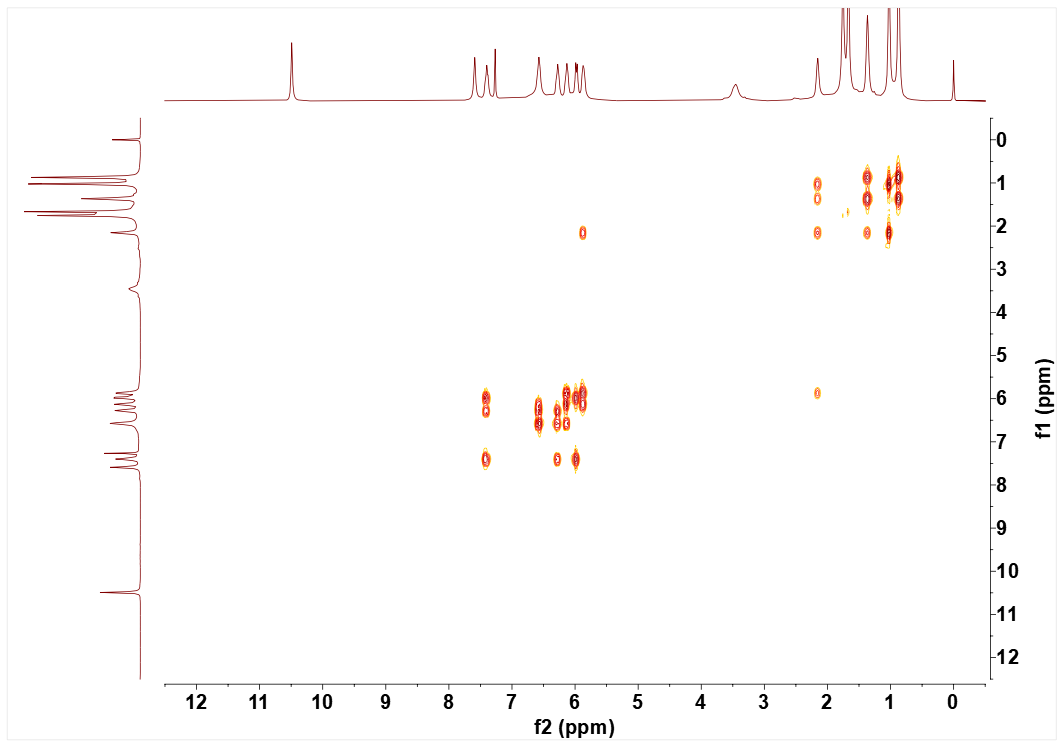


# **Fig. S23.** HSQC spectrum of **3**


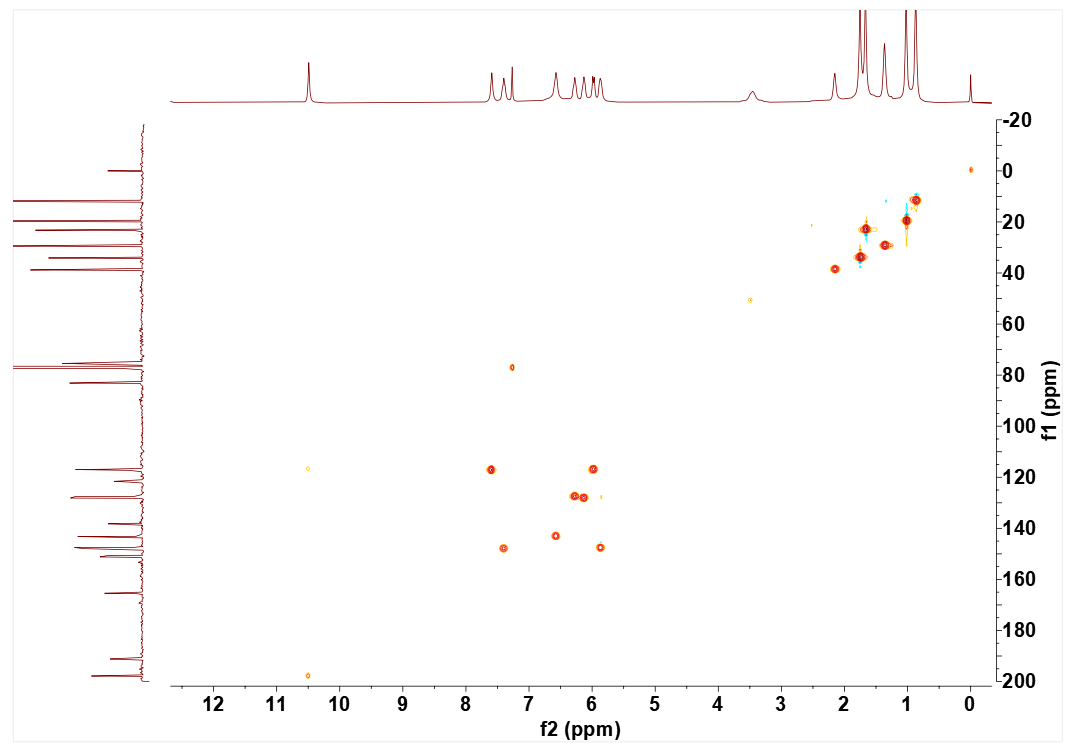


# **Fig. S24.** HMBC spectrum of **3**


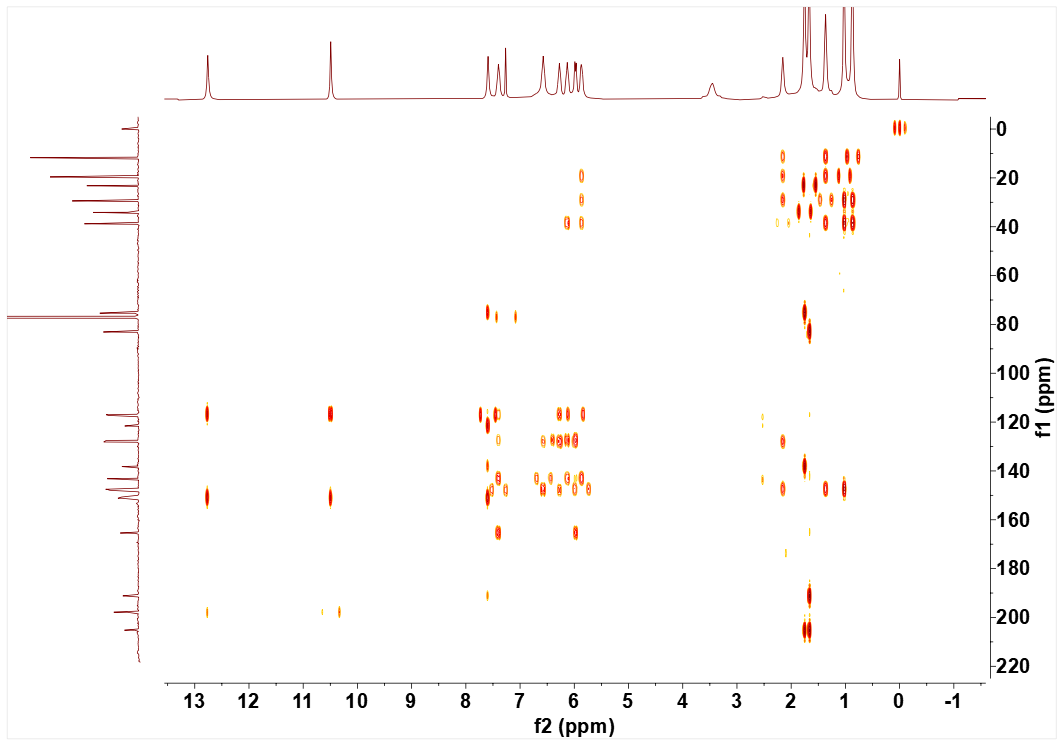


# Fig. S25. NOESY spectrum of 3


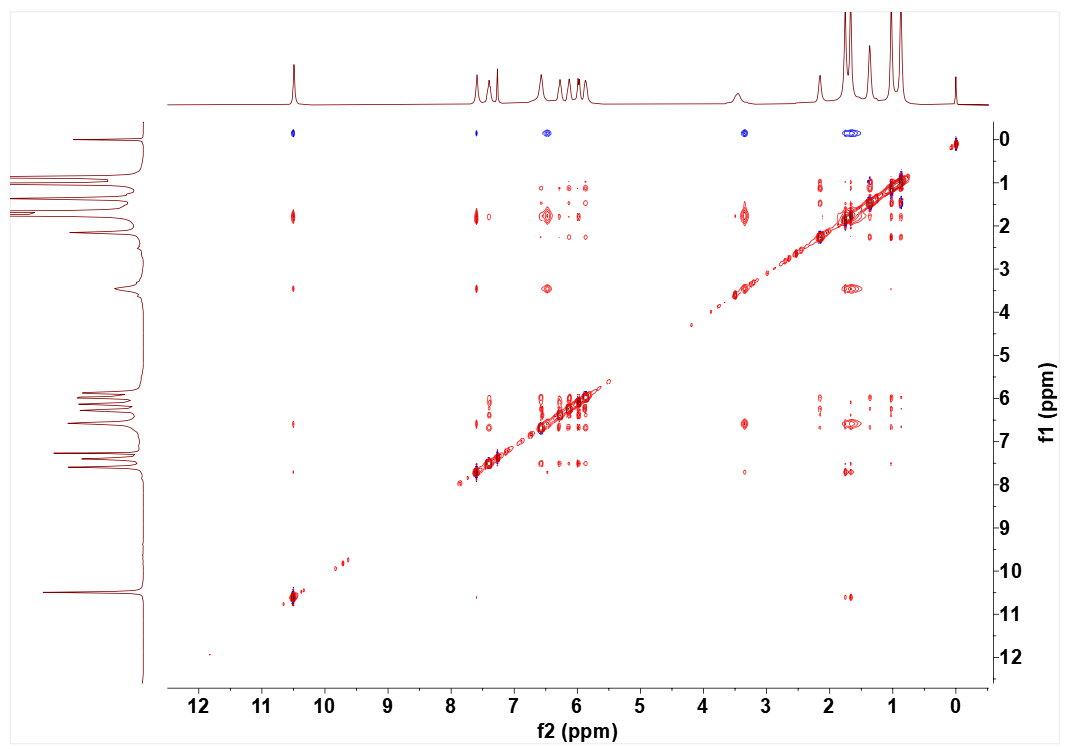


# Fig. S26. HR-ESI-MS spectrum of 3


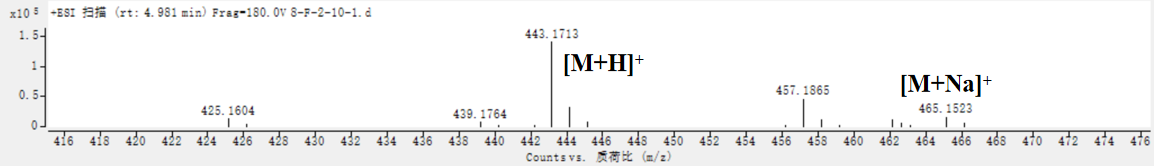


# Fig. S27. IR spectra of 3

# Fig. S28. UV spectrum (methanol) of 3


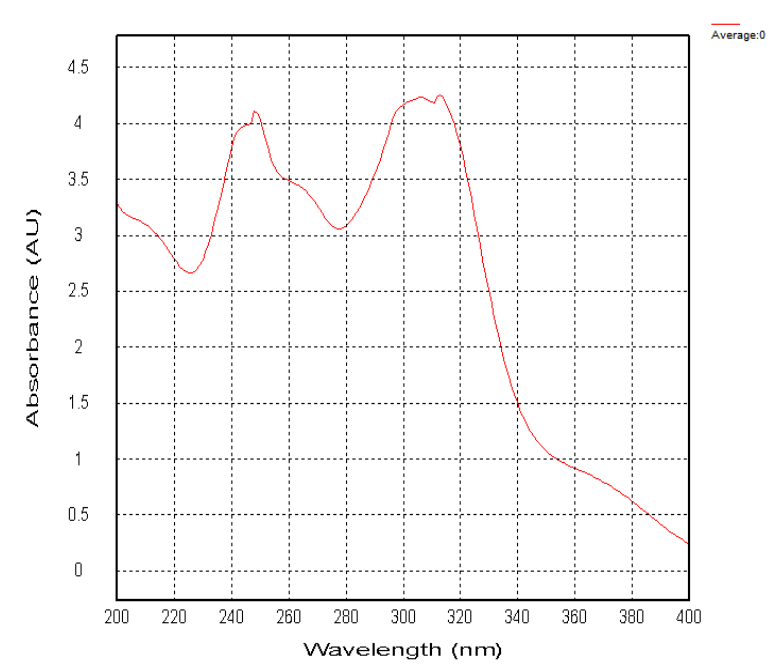


# Fig. S29. ^1^H NMR spectrum of 4 (600 MHz, CDCl_3_)


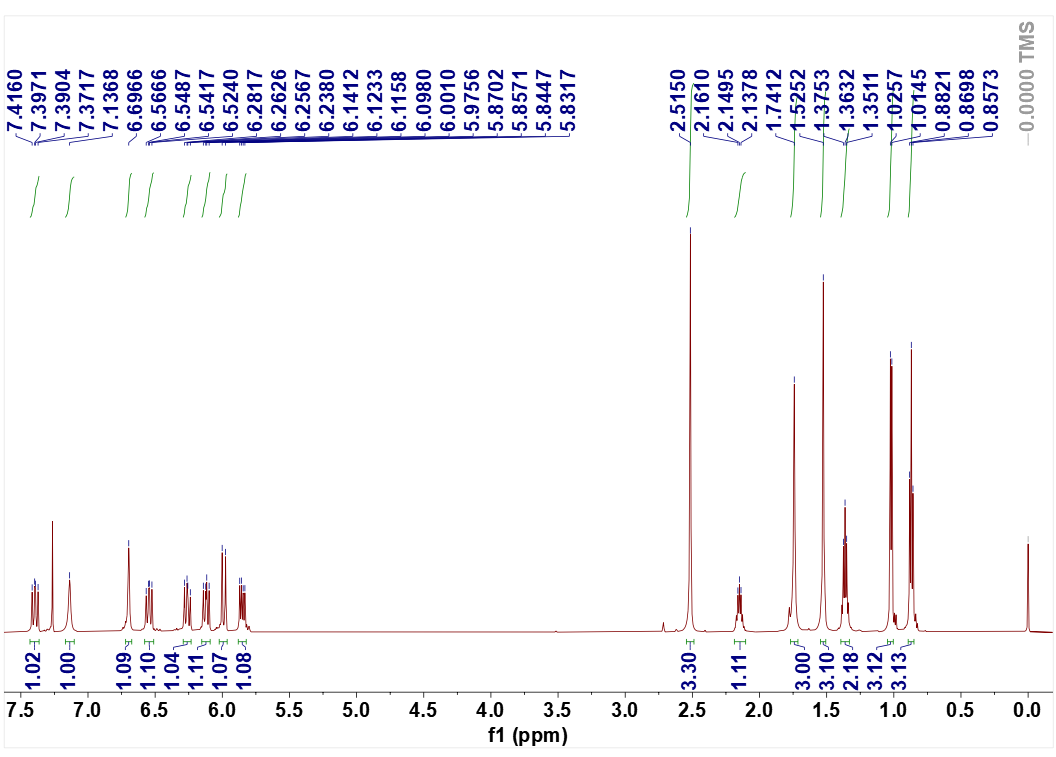


# Fig. S30. ^13^C NMR and DEPT spectrum of 4 (150 MHz, CDCl_3_)


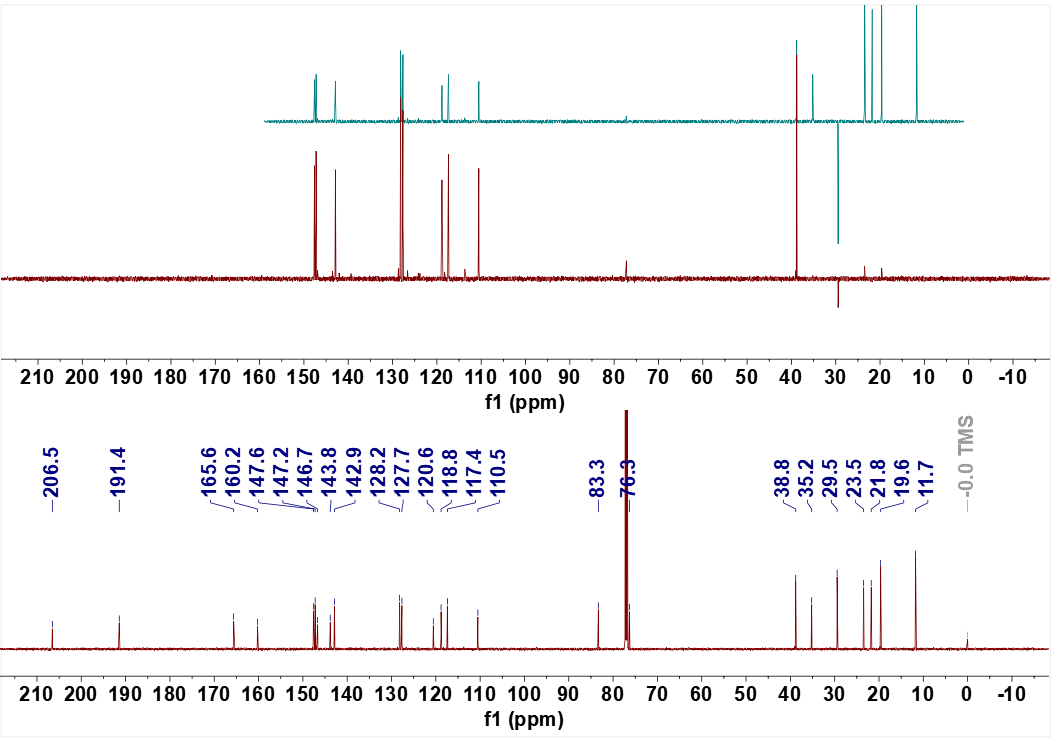


# Fig. S31.^1^H-^1^H COSY spectrum of 4


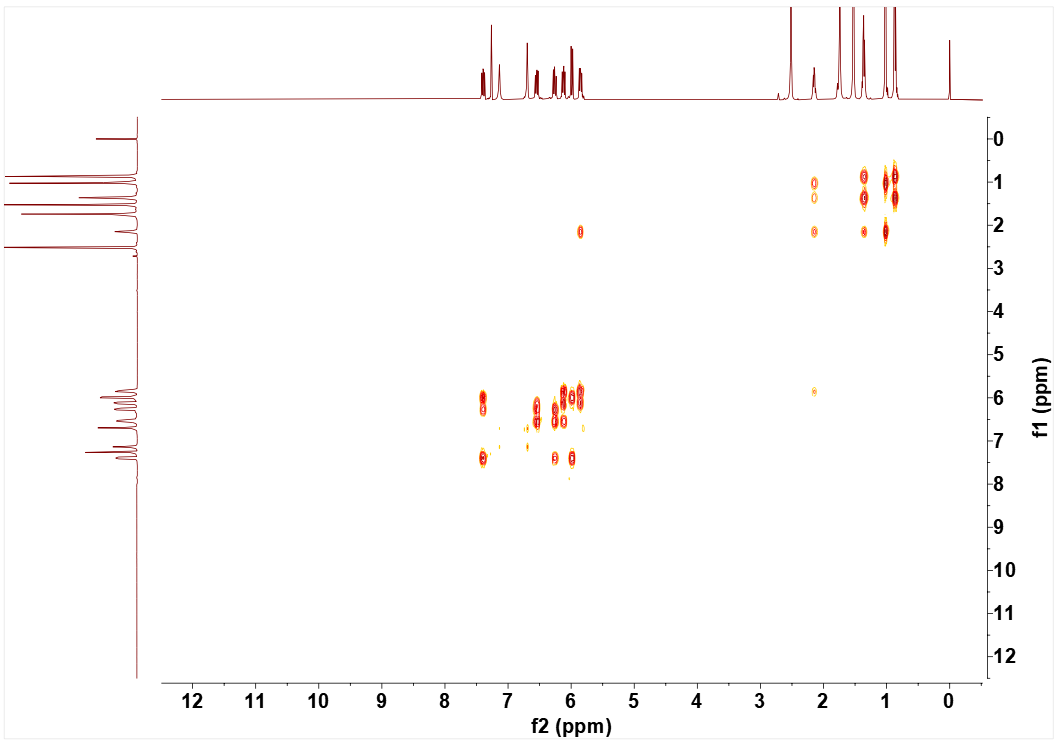


# **Fig. S32.** HSQC spectrum of **4**


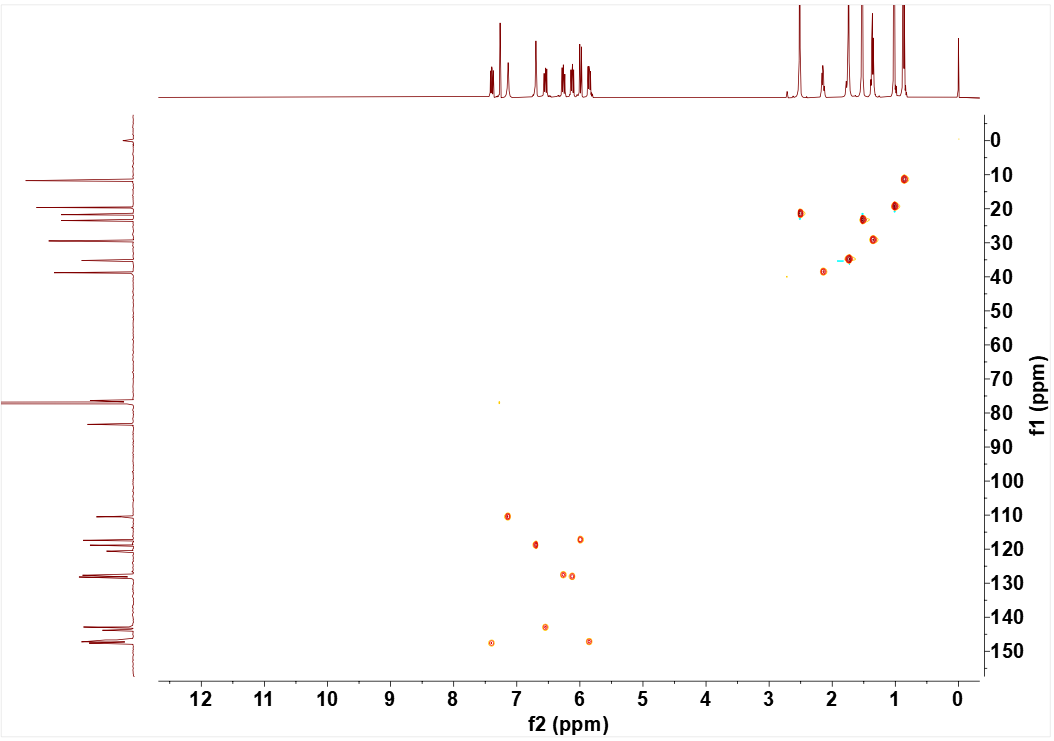


# **Fig. S33.** HMBC spectrum of **4**


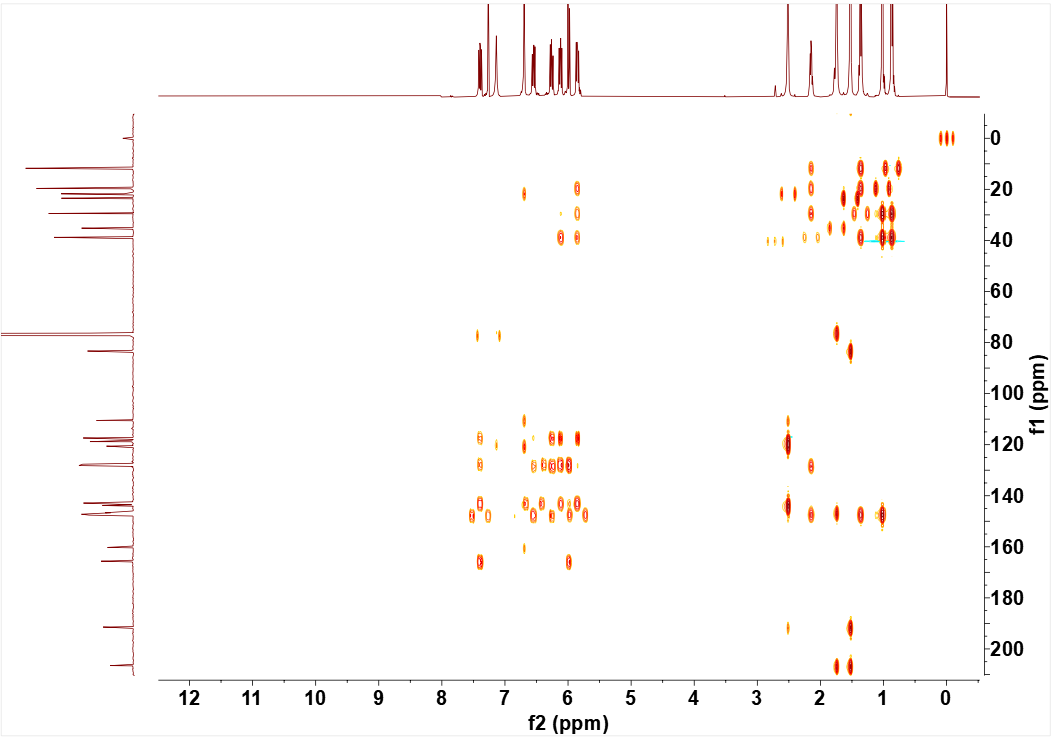


# Fig. S34. NOESY spectrum of 4


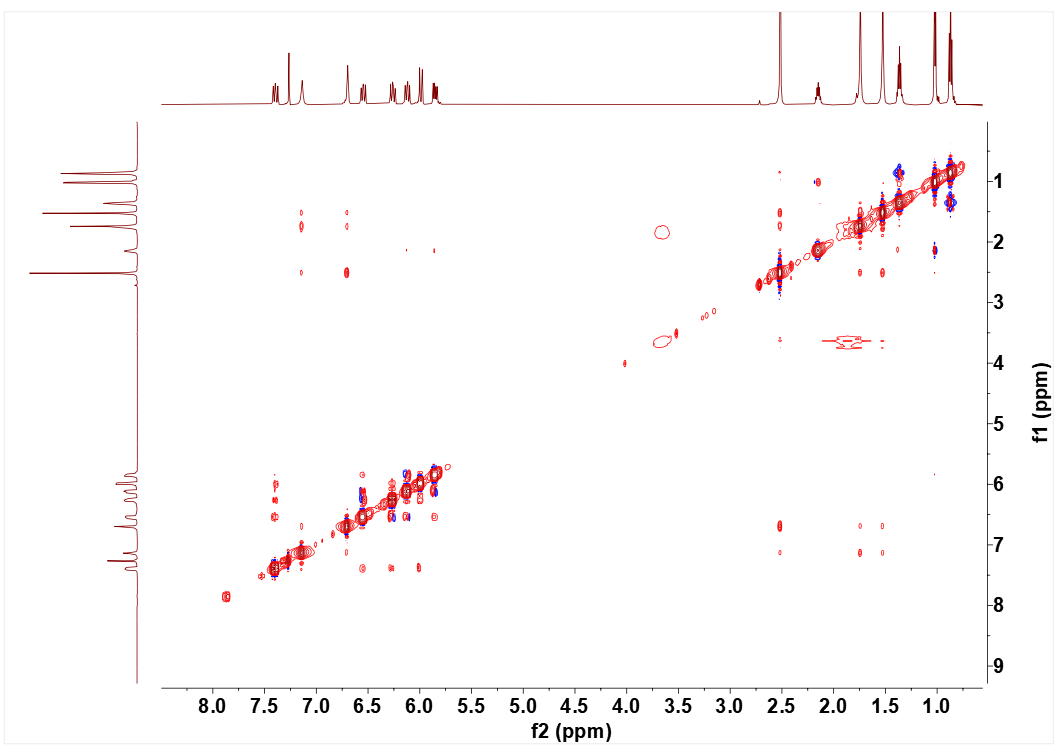


# Fig. S35. 1D NOE (CDCl_3_) spectrum of compound 4


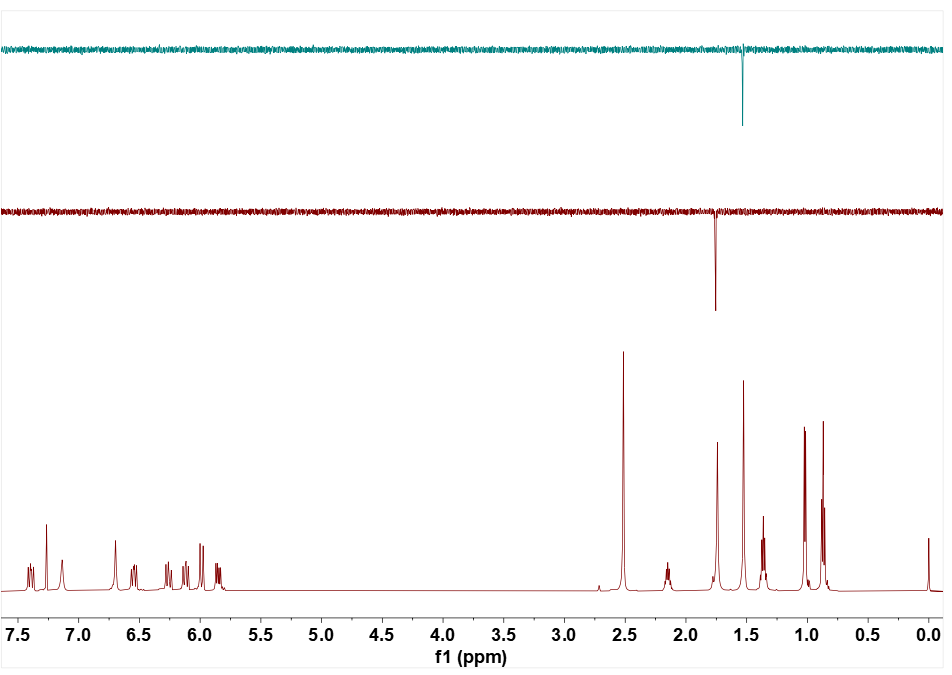


# Fig. S36. HR-ESI-MS spectrum of 4


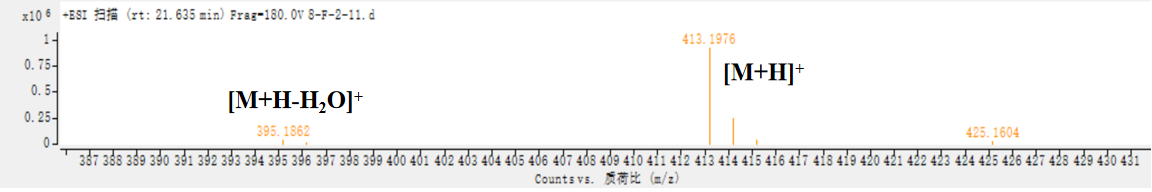


# Fig. S37. IR spectra of 4

# Fig. S38. UV spectrum (methanol) of 4


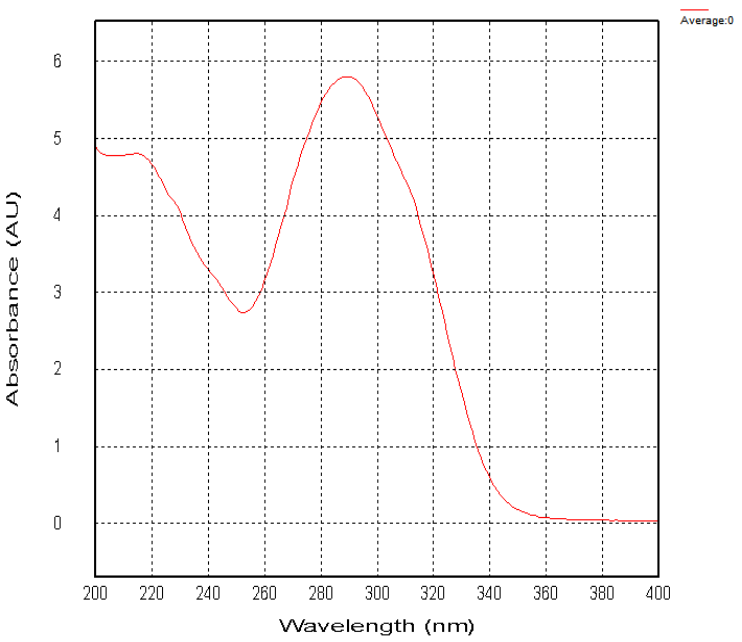


# Fig. S39. ^1^H NMR spectrum of 5 (600 MHz, CDCl_3_)


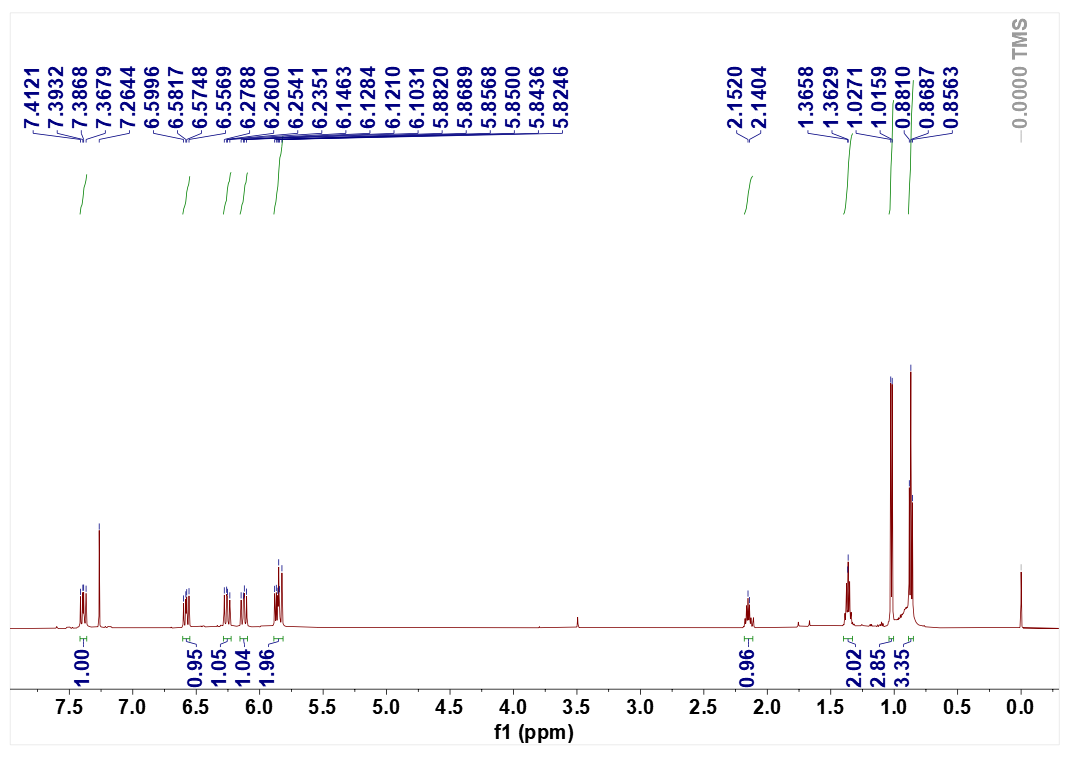


# Fig. S40. ^13^C NMR and DEPT spectrum of 5 (150 MHz, CDCl_3_)


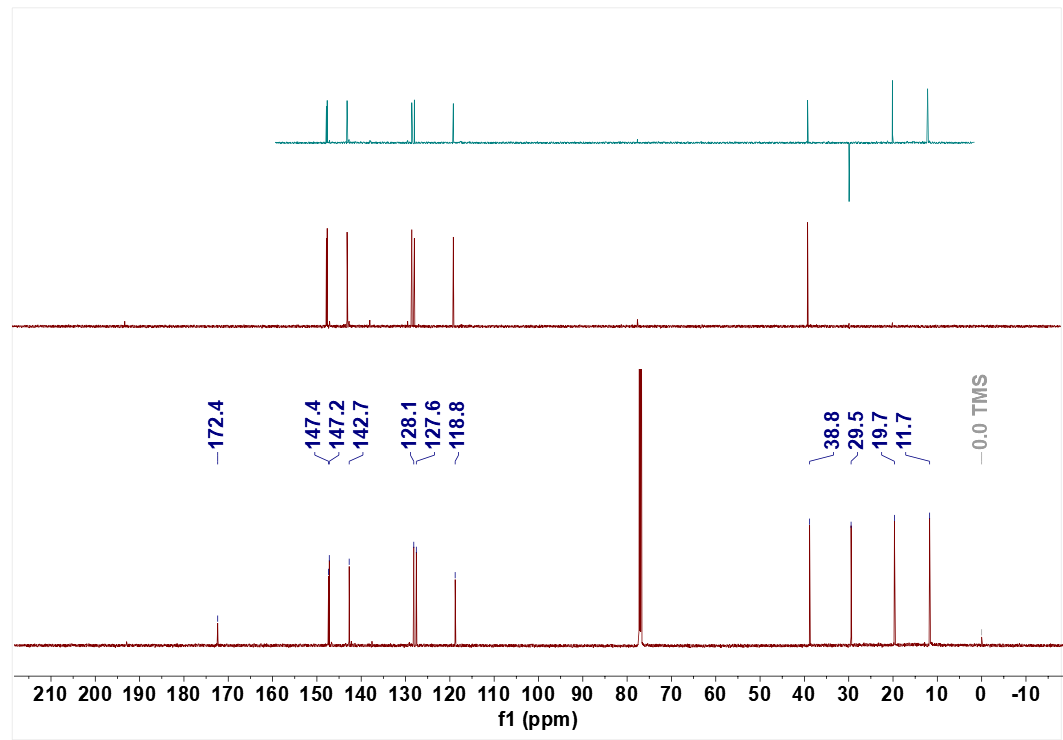


# Fig. S41.^1^H-^1^H COSY spectrum of 5


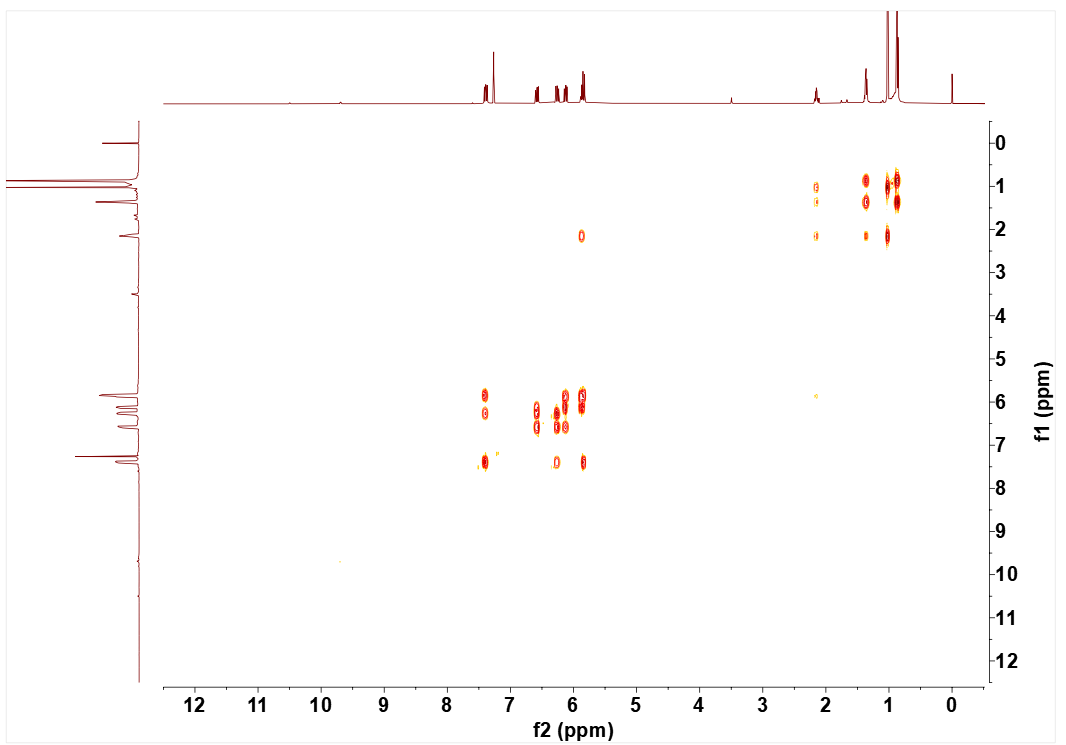


# **Fig. S42.** HSQC spectrum of **5**


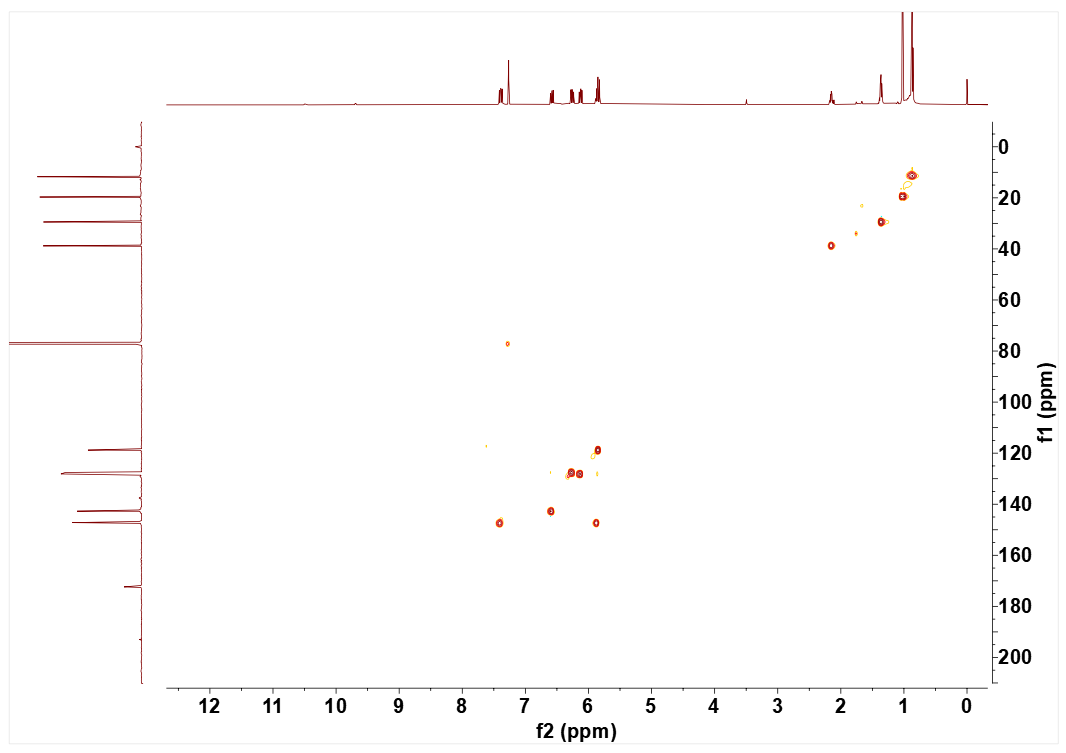


# **Fig. S43.** HMBC spectrum of **5**


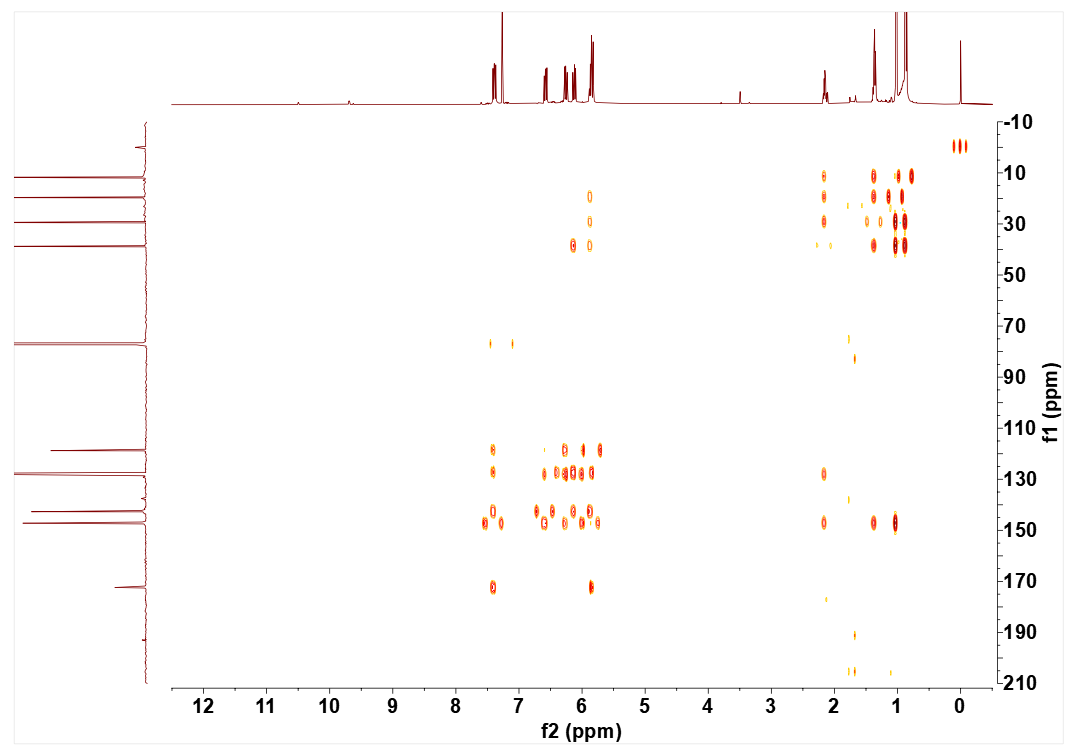


# Fig. S44. HR-ESI-MS spectrum of 5


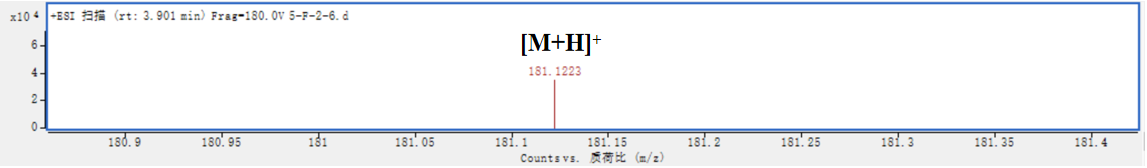


# Fig. S45. IR spectra of 5

# Fig. S46. UV spectrum (methanol) of 5


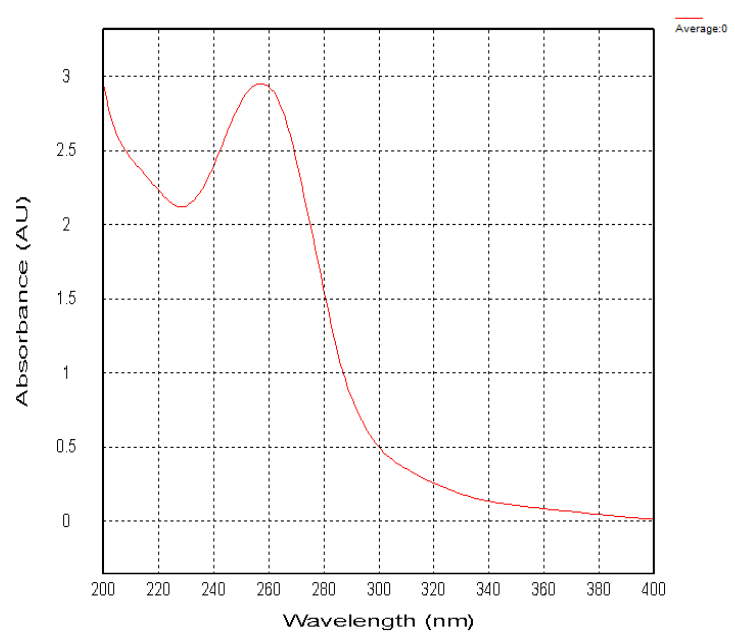


# Fig. S47. ^1^H NMR spectrum of 6 (600 MHz, CDCl_3_)


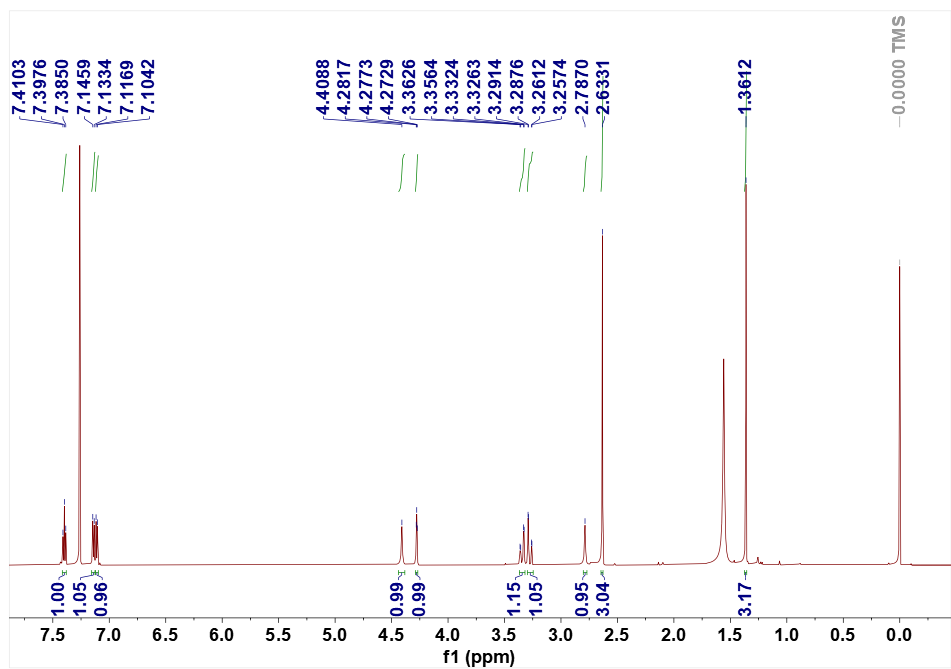


# Fig. S48. ^13^C NMR and DEPT spectrum of 6 (150 MHz, CDCl_3_)


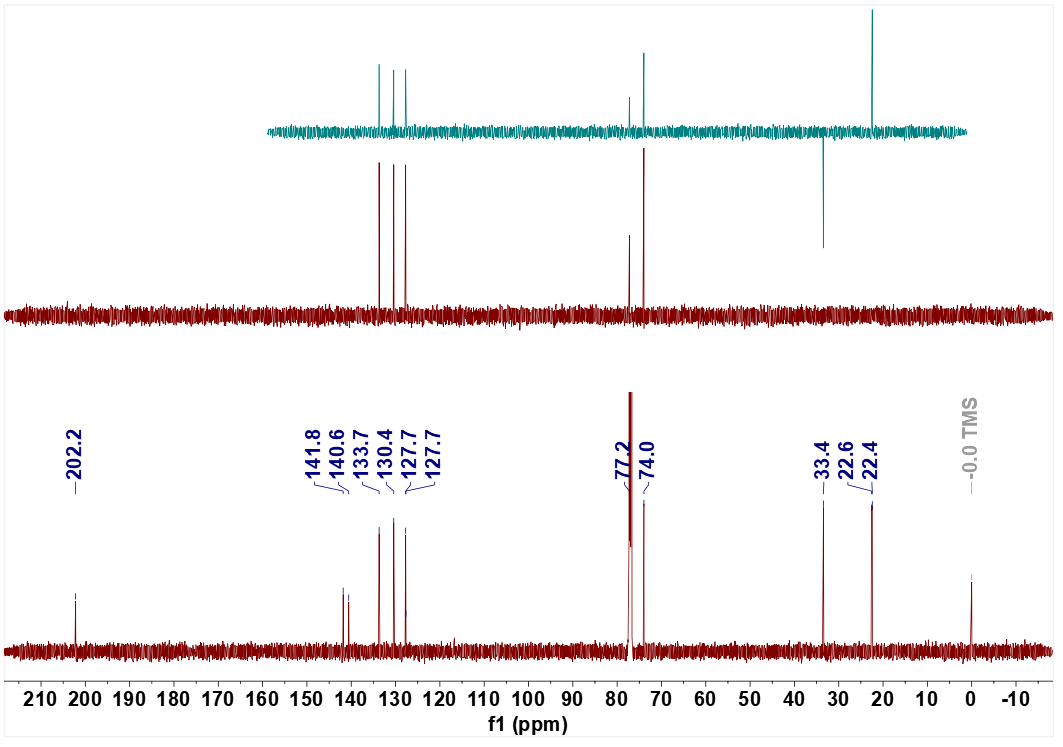


Fig. S49. ^1^H-^1^H COSY spectrum of 6


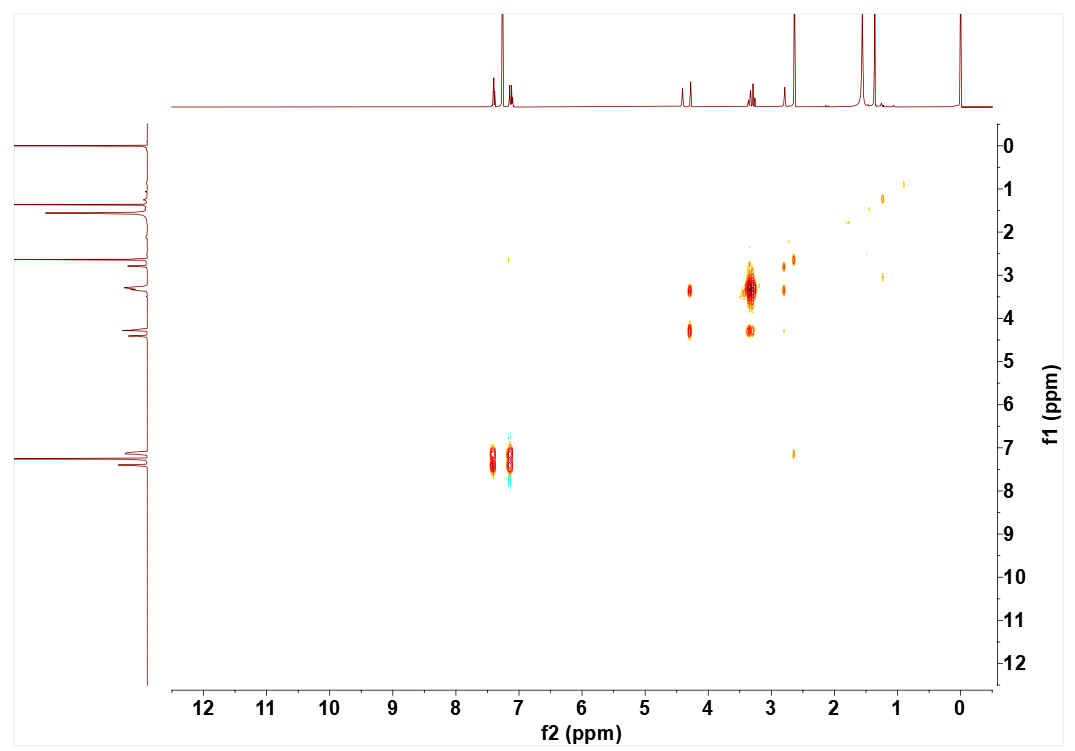


Fig. S50. HSQC spectrum of 6


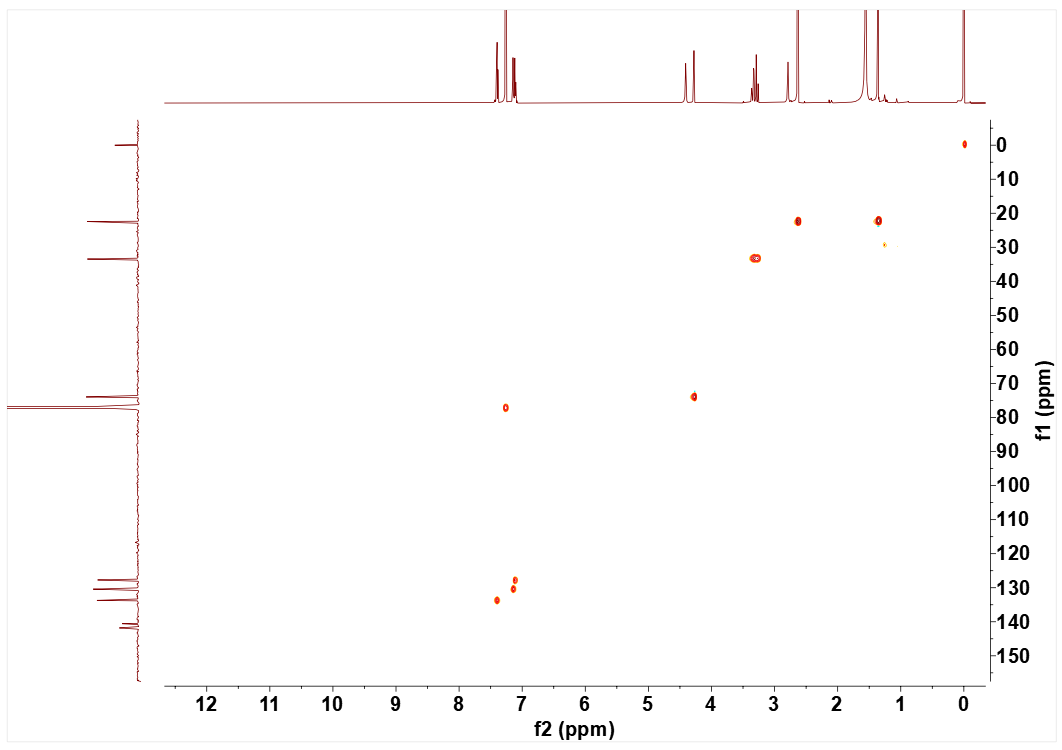


Fig. S51. HMBC spectrum of 6


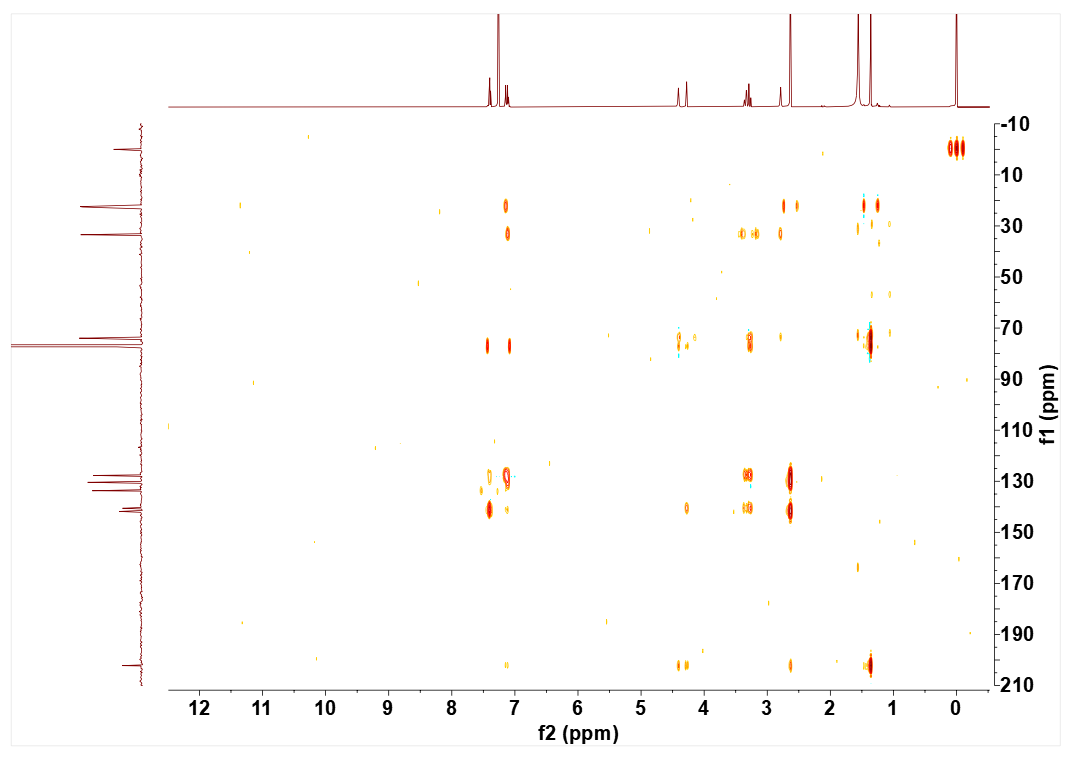


# Fig. S52. NOESY spectrum of 6


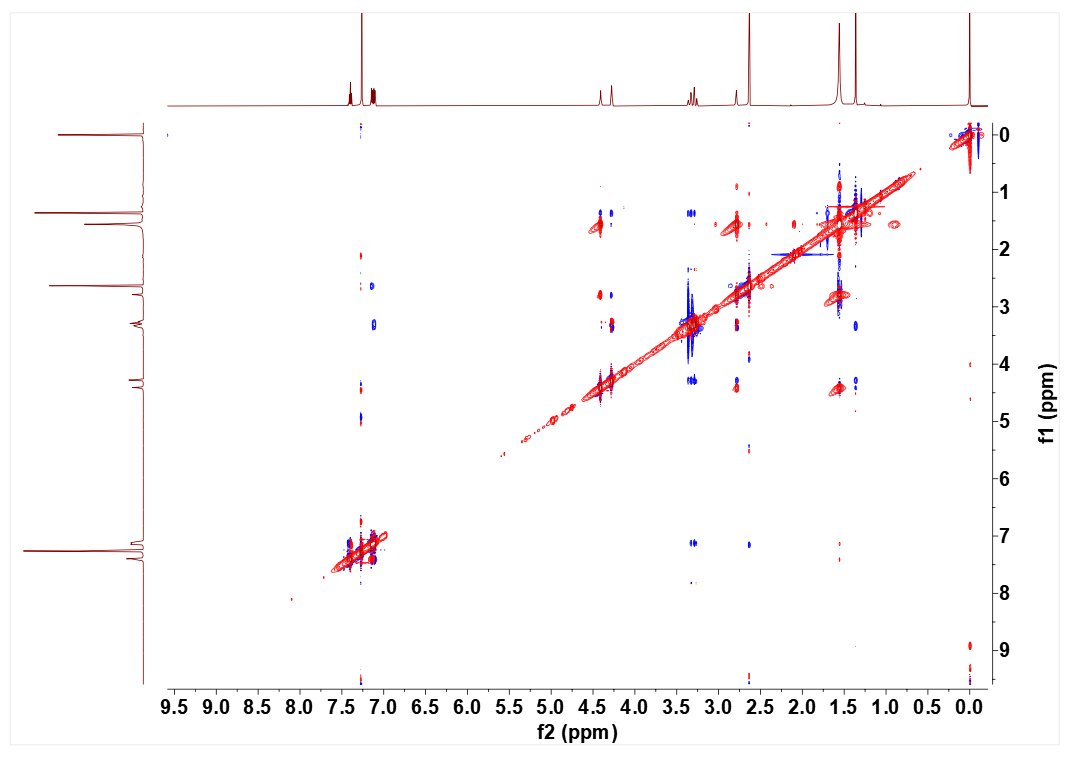


# Fig. S53. HR-ESI-MS spectrum of 6


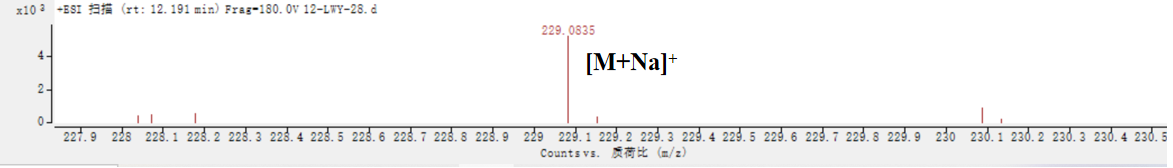


# Fig. S54. UV spectrum (methanol) of 6


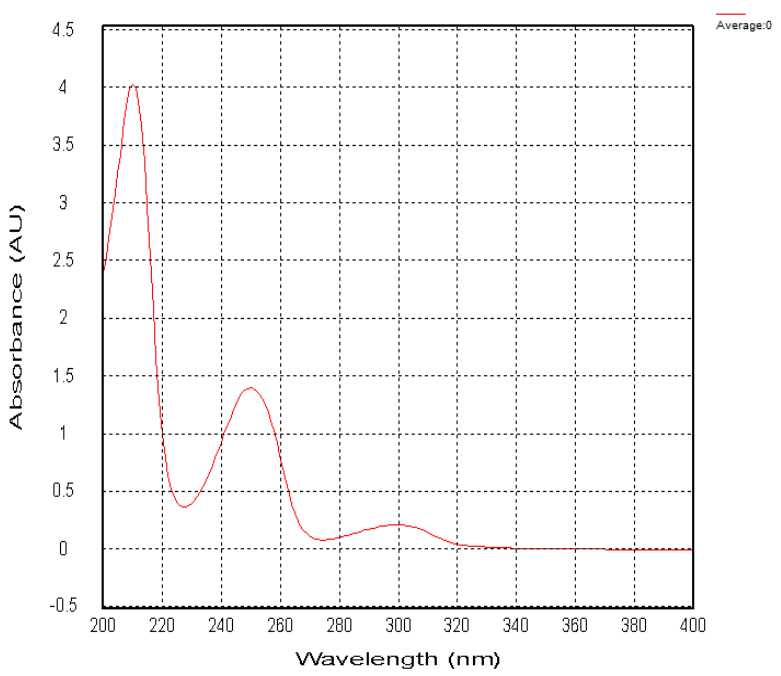


# Fig. S55. ^1^H NMR spectrum of 7 (600 MHz, CDCl_3_)


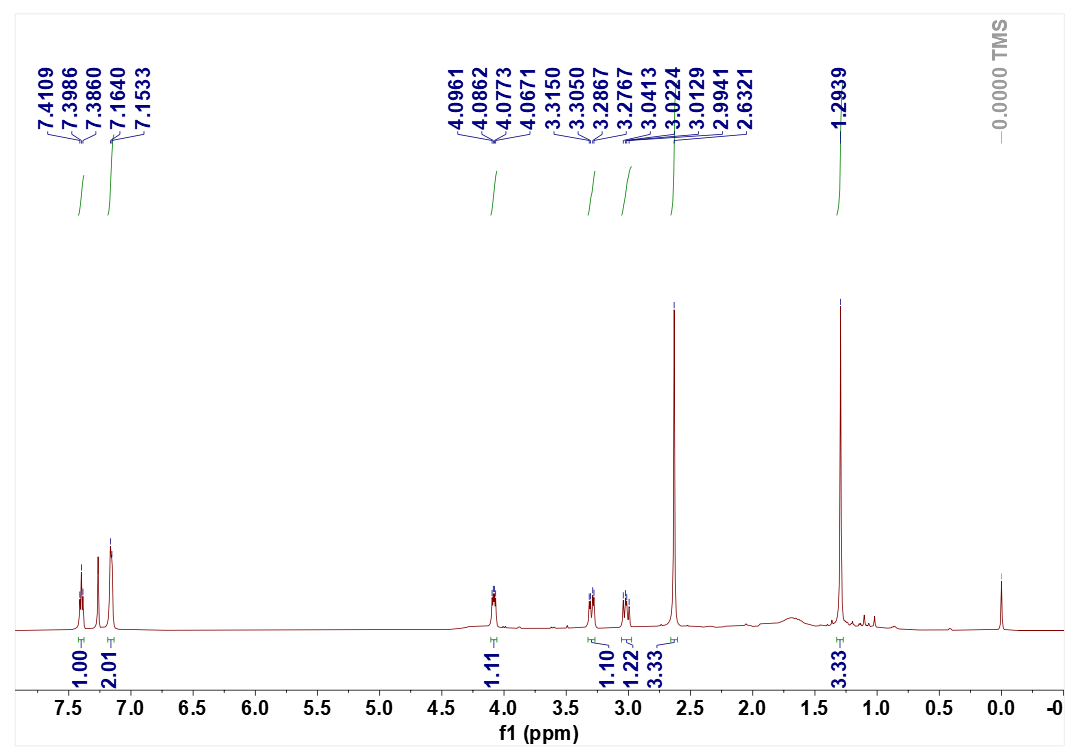


# Fig. S56. ^13^C NMR and DEPT spectrum of 7 (150 MHz, CDCl_3_)


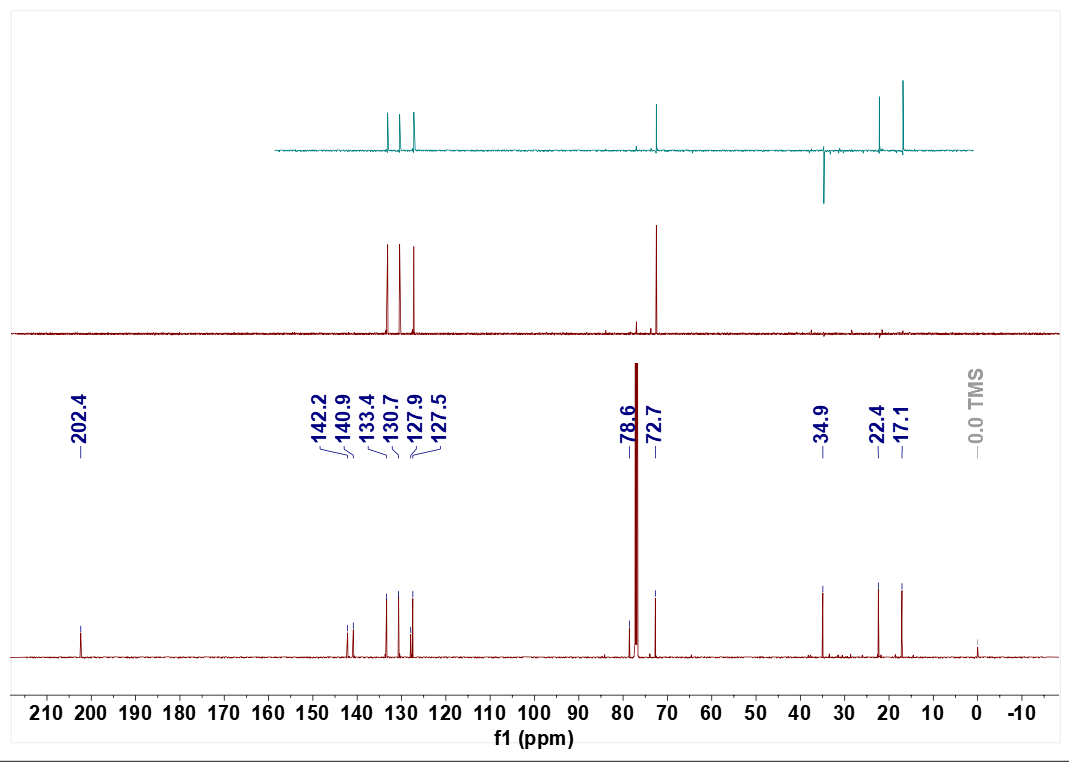


Fig. S57. ^1^H-^1^H COSY spectrum of 7


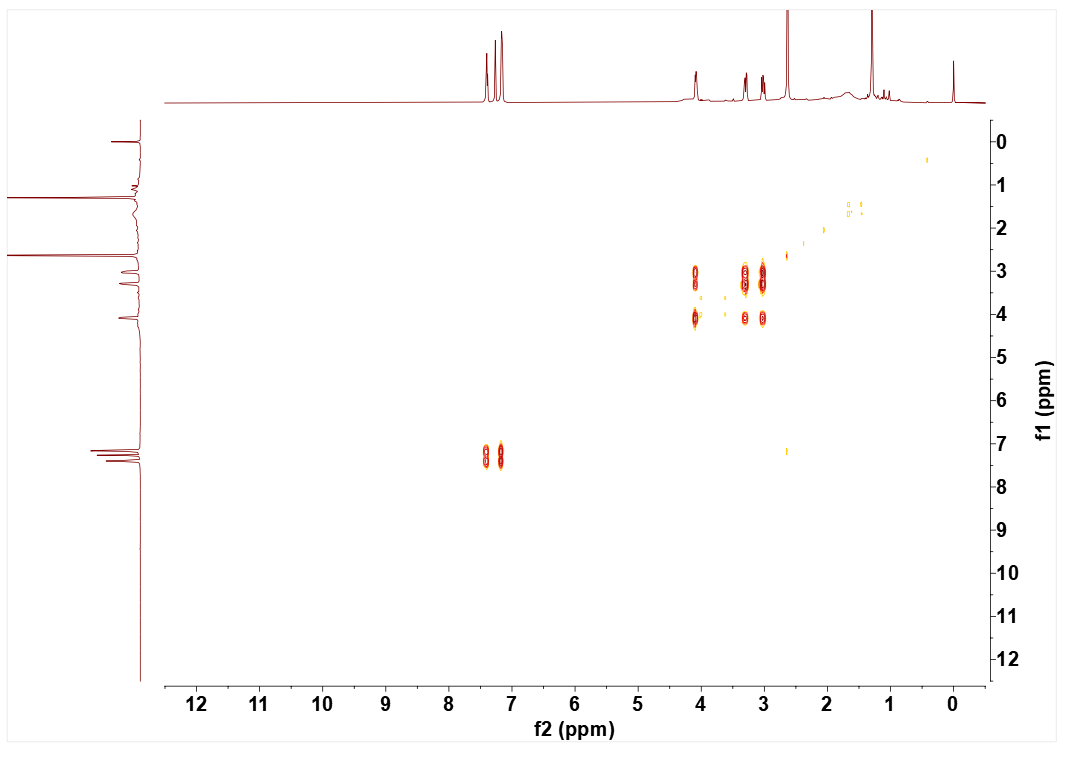


# Fig. S58. HSQC spectrum of 7


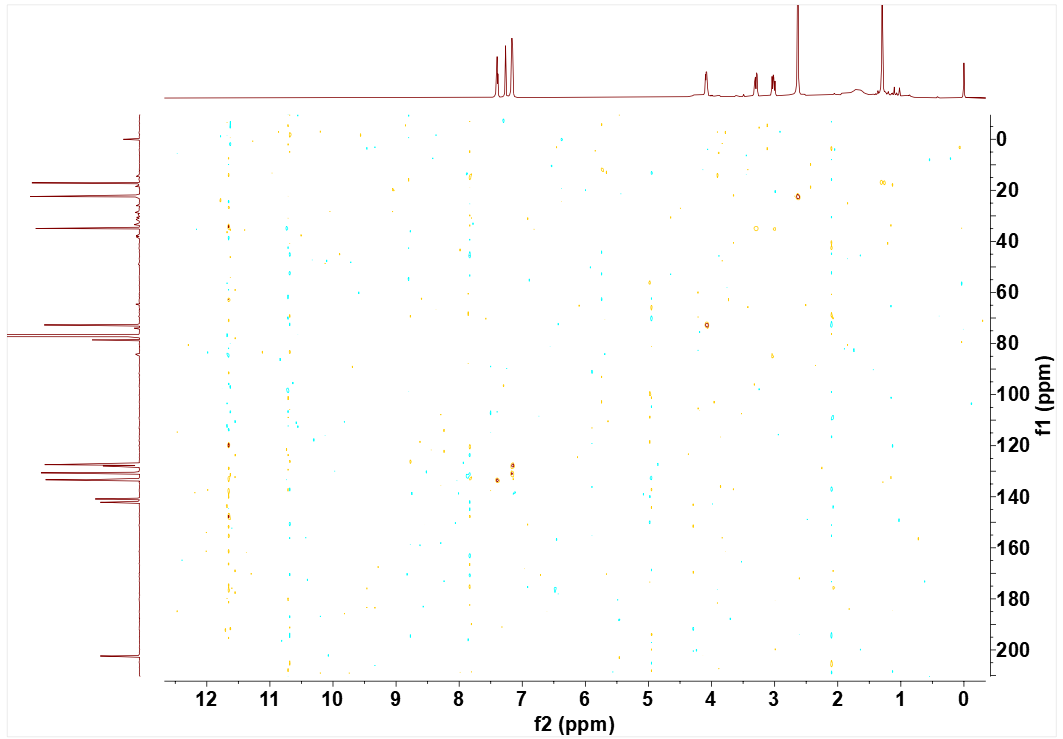


# Fig. S59. HMBC spectrum of 7


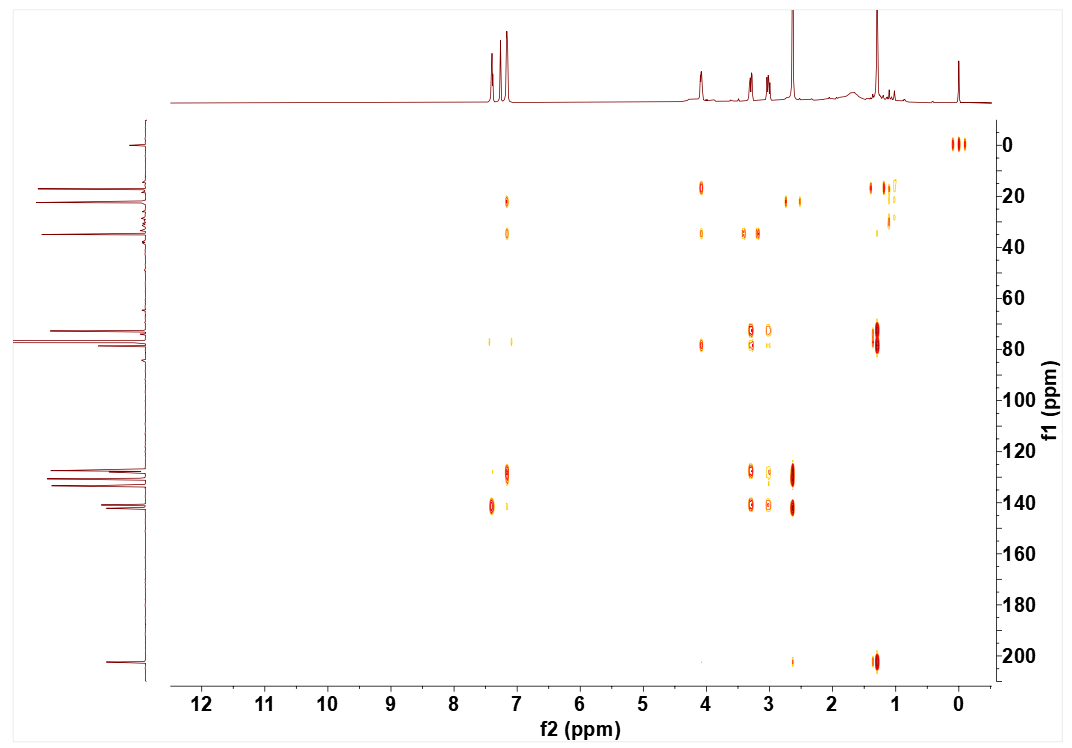


# Fig. S60. NOESY spectrum of 7


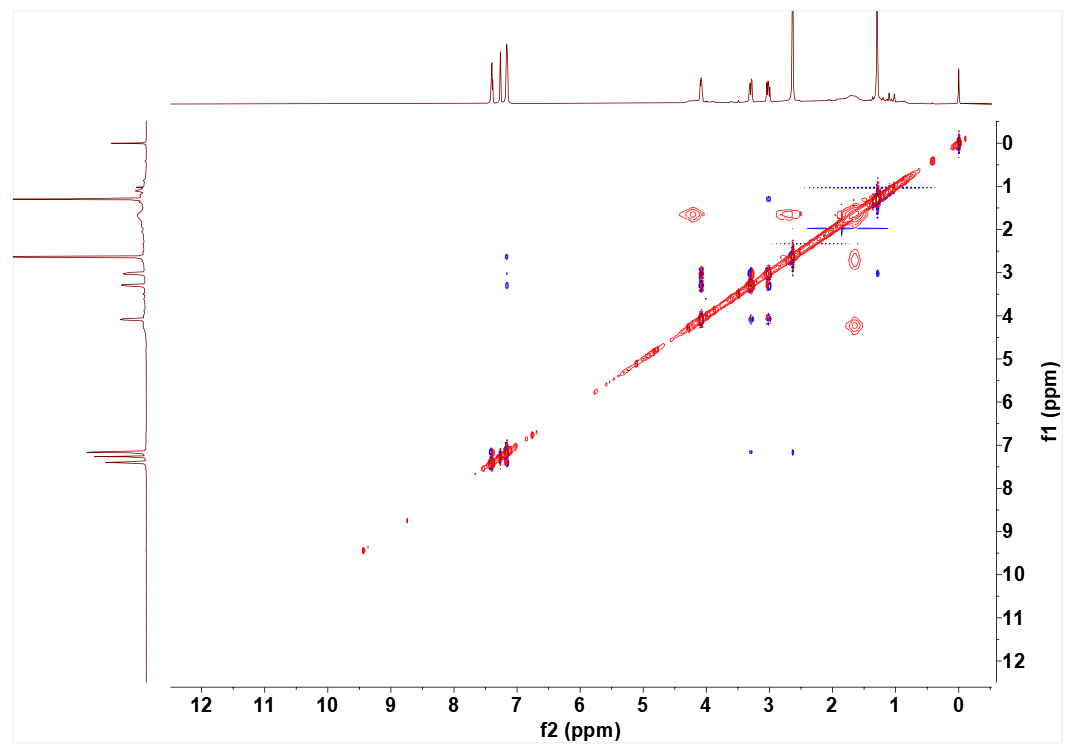


# Fig. S61. HR-ESI-MS spectrum of 7


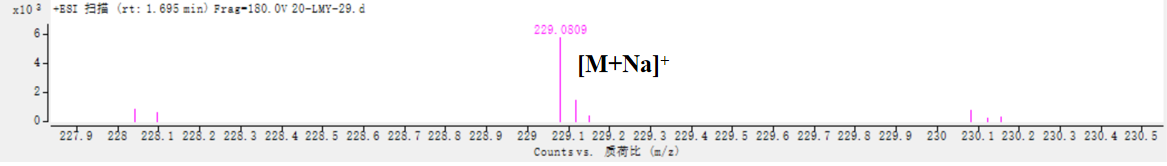


# Fig. S62. UV spectrum (methanol) of 7


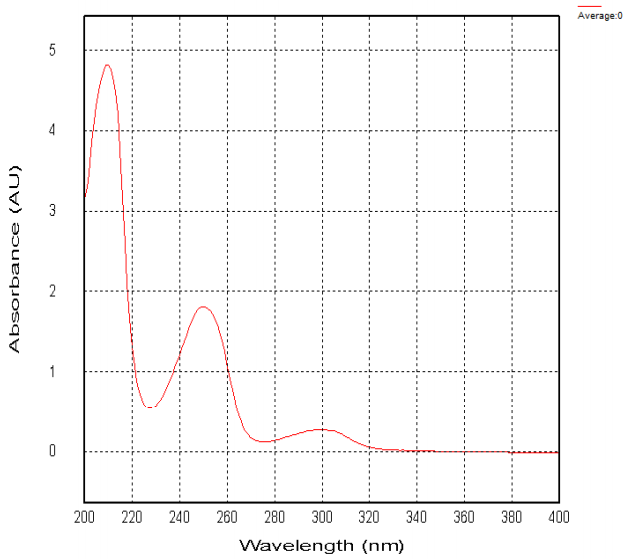


# Fig. S63. ^1^H NMR spectrum of 8 (600 MHz, CDCl_3_)


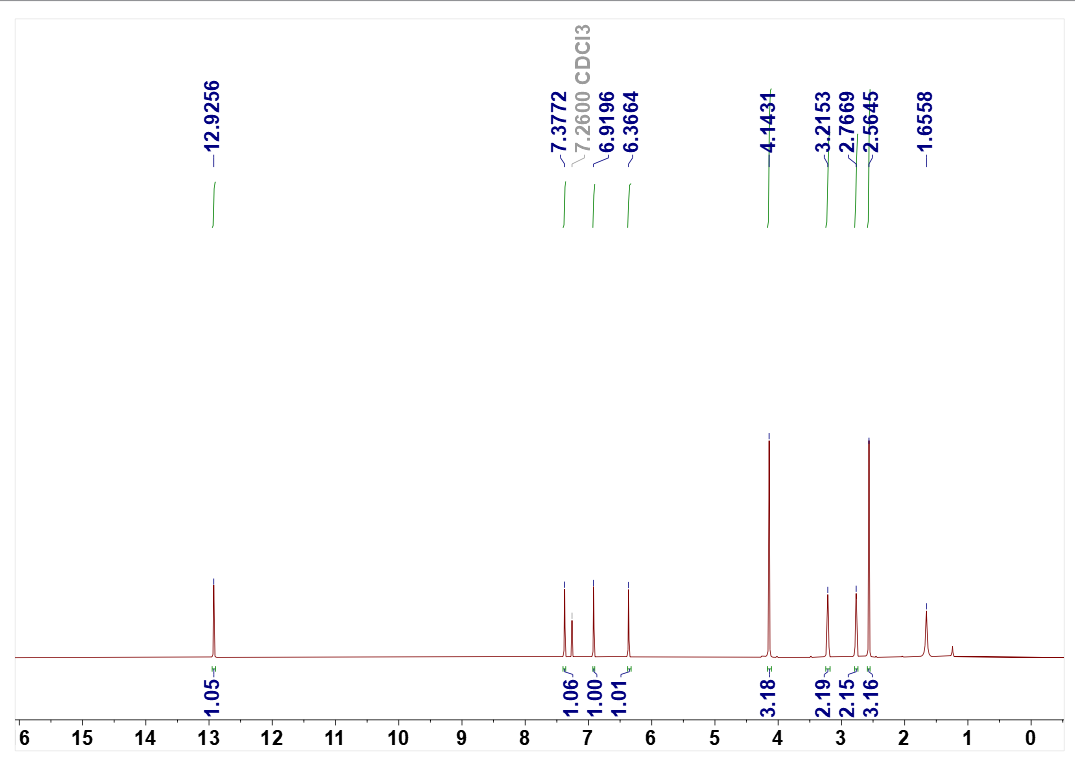


# Fig. S64. ^13^C NMR and DEPT spectrum of 8 (150 MHz, CDCl_3_)


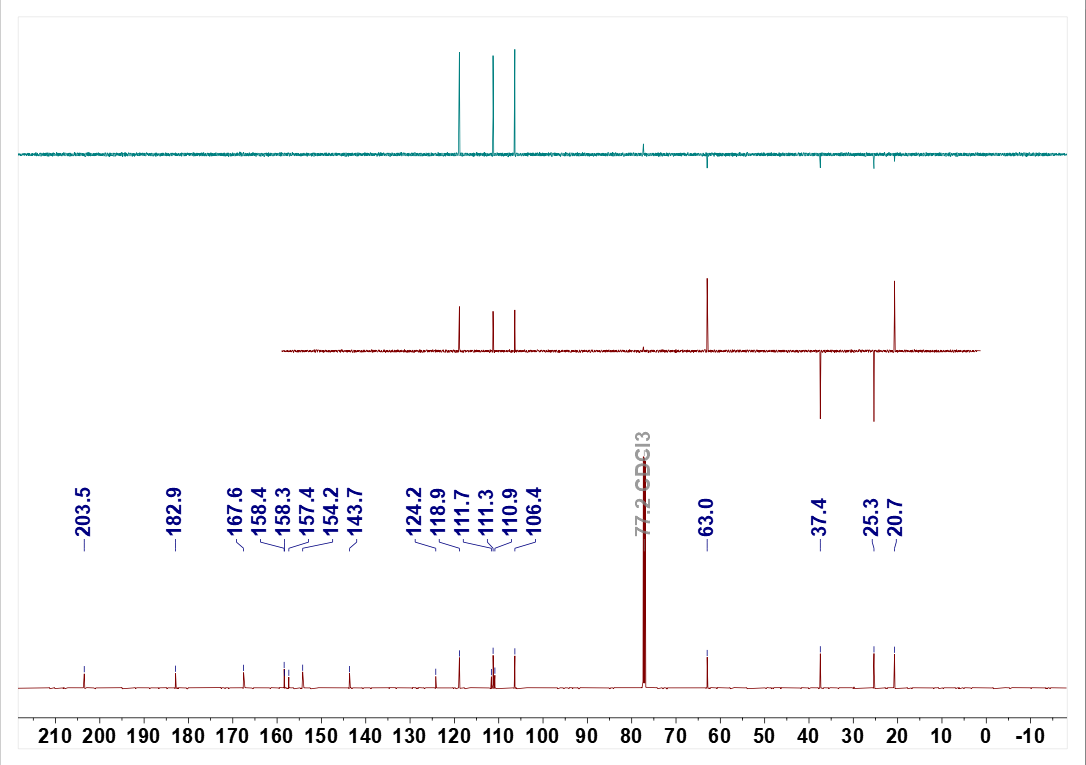


# Fig. S65. HMBC spectrum of 8


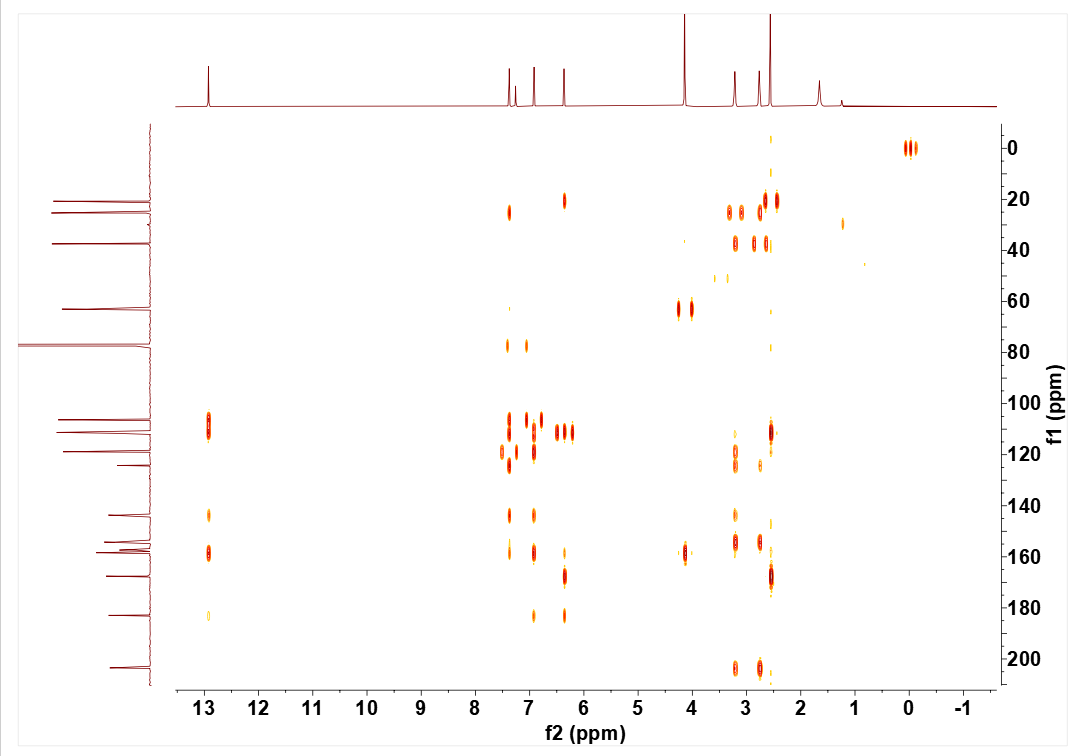


# Fig. S66. HR-ESI-MS spectrum of 8


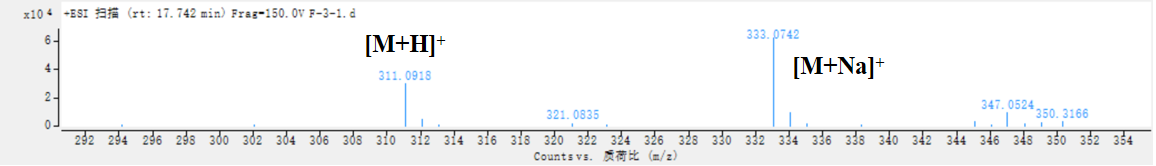


# Table S67. Experimental ¹³C NMR chemical shifts (CDCl₃) of 2 and the calculated values for diastereoisomers 2a, 2a', 2b and 2b' obtained by GIAO-STS ¹³C NMR calculations.

| No. | **2*** | | | | |
| --- | --- | --- | --- | --- | --- |
|  | Exptl. | **2a** | **2a'** | **2b** | **2b'** |
| 1 | 74.4 | 75.96 | 76 | 77.02 | 76.88 |
| 2 | 205 | 209.36 | 209.32 | 208.21 | 208.34 |
| 3 | 81.4 | 83.25 | 83.32 | 83.5 | 83.3 |
| 4 | 196.5 | 196.53 | 196.57 | 196.5 | 196.62 |
| 5 | 107 | 106.62 | 106.64 | 107.68 | 107.66 |
| 6 | 151.4 | 148.2 | 148.21 | 149.25 | 149.28 |
| 7 | 130.8 | 127.72 | 127.79 | 127.71 | 127.68 |
| 8 | 149.7 | 149.14 | 149.15 | 149.2 | 149.18 |
| 9 | 106.7 | 106.94 | 107 | 106.01 | 105.89 |
| 10 | 136.9 | 137.77 | 137.78 | 139.9 | 139.89 |
| 11 | 31.9 | 35.54 | 35.56 | 34.32 | 34.19 |
| 12 | 23.9 | 23.83 | 23.85 | 22.33 | 22.1 |
| 1' | 165.9 | 166.83 | 166.83 | 166.03 | 166.09 |
| 2' | 116.9 | 115.01 | 115.02 | 115.09 | 114.98 |
| 3' | 148 | 148.15 | 148.16 | 147.8 | 147.82 |
| 4' | 127.5 | 124.33 | 124.34 | 124.37 | 124.34 |
| 5' | 143.2 | 144 | 144.04 | 143.71 | 143.72 |
| 6' | 128.2 | 125.91 | 125.79 | 125.9 | 125.8 |
| 7' | 147.5 | 148.52 | 148.44 | 148.16 | 148.17 |
| 8' | 38.8 | 38.92 | 38.83 | 39.09 | 39.43 |
| 9' | 29.4 | 28.85 | 28.44 | 28.91 | 28.54 |
| 10' | 11.7 | 11.75 | 11.76 | 11.85 | 12.17 |
| 11' | 19.6 | 19.16 | 19.48 | 19.77 | 20.23 |
|  | **MAE** | 1.36 | 1.36 | 1.38 | 1.47 |
|  | **RMS** | 1.88 | 1.88 | 1.79 | 1.82 |
|  | Pmean | 24.92% | 24.55% | 26.11% | 24.31% |
|  | Prel | 19.23% | 13.69% | 56.21% | 10.88% |

# Table S68. Experimental ¹³C NMR chemical shifts (CDCl₃) of 3 and the calculated values for diastereoisomers 3a, 3a', 3b and 3b' obtained by GIAO-STS ¹³C NMR calculations.

| No. | **3*** | | | | |
| --- | --- | --- | --- | --- | --- |
|  | Exptl. | **3a** | **3a'** | **3b** | **3b'** |
| 1 | 75.4 | 76.46 | 76.73 | 76.99 | 76.88 |
| 2 | 205.2 | 208.46 | 208.19 | 206.75 | 206.83 |
| 3 | 83.1 | 84.81 | 85.03 | 83.66 | 83.54 |
| 4 | 191.2 | 192.45 | 192.35 | 192.9 | 193 |
| 5 | 121.6 | 122.6 | 122.59 | 123.81 | 123.73 |
| 6 | 116.9 | 116.72 | 116.76 | 117.45 | 117.41 |
| 7 | 151.2 | 149.55 | 149.44 | 149.13 | 149.15 |
| 8 | 150.7 | 147.77 | 147.83 | 147.86 | 147.84 |
| 9 | 117.2 | 117.49 | 117.64 | 116.68 | 116.61 |
| 10 | 138.2 | 137.71 | 137.84 | 139.5 | 139.48 |
| 11 | 34.1 | 35.62 | 36 | 33.53 | 33.32 |
| 12 | 23.2 | 23.14 | 23.55 | 22.92 | 22.69 |
| 13 | 197.8 | 196.92 | 196.85 | 197.51 | 197.66 |
| 1' | 165.4 | 166.4 | 166.33 | 166.05 | 166.07 |
| 2' | 117 | 115.11 | 115.27 | 114.64 | 114.57 |
| 3' | 147.9 | 148.12 | 148.12 | 148.21 | 148.19 |
| 4' | 127.5 | 124.4 | 124.49 | 124.22 | 124.2 |
| 5' | 143.2 | 143.96 | 144.06 | 144.19 | 144.15 |
| 6' | 128.1 | 125.92 | 125.92 | 125.72 | 125.57 |
| 7' | 147.5 | 148.48 | 148.49 | 148.6 | 148.64 |
| 8' | 38.8 | 39.55 | 38.79 | 39.47 | 39.91 |
| 9' | 29.4 | 28.32 | 28.82 | 29.85 | 29.18 |
| 10' | 11.7 | 12.22 | 11.7 | 12.49 | 12.85 |
| 11' | 19.6 | 19.71 | 19.13 | 19.77 | 20.41 |
|  | **MAE** | 1.20 | 1.17 | 1.22 | 1.28 |
|  | **RMS** | 1.51 | 1.49 | 1.5 | 1.55 |
|  | ***P***mean | 36.11% | 36.51% | 38.35% | 35.13% |
|  | ***P***rel | 14.18% | 18.40% | 60.11% | 7.31% |

# Table S69. Geometry data of conformers of structure 1a

1a-c45 , delta G = 0.0000 kcal/mol, population = 36.86 %

C 2.630878 -2.330615 -0.363201

C 1.597939 -2.112678 0.749416

C 1.460016 -0.723784 1.372212

C 2.048041 0.388414 0.485414

C 3.365439 0.093281 -0.098229

C 4.315087 1.126448 -0.304741

C 5.547503 0.787772 -0.852124

C 5.852894 -0.548161 -1.160999

C 4.919517 -1.548696 -0.961482

C 3.678802 -1.236469 -0.425588

C 1.880727 -2.402699 -1.708304

C 2.235116 -0.734240 2.700258

C 4.020238 2.566418 0.043957

C 5.040361 4.622833 0.669764

C -0.794349 -0.466492 0.699806

C -2.120329 -0.080839 1.162645

C -3.170842 -0.059238 0.318341

C -4.505154 0.310629 0.684267

C -5.531262 0.315962 -0.196249

C -6.885886 0.677556 0.129168

C -7.889981 0.666781 -0.760033

C -9.311783 1.031811 -0.472363

C -10.250858 -0.165707 -0.718983

C -9.981649 -1.358801 0.194141

C -9.735711 2.235914 -1.327359

O 0.108594 -0.440507 1.719909

O -0.477318 -0.790189 -0.428180

O 0.961062 -3.050864 1.167343

O 3.289027 -3.570233 -0.108984

O 1.477932 1.458926 0.394593

O 5.235945 3.236322 0.407444

O 6.546661 1.675097 -1.130306

O 7.069711 -0.848376 -1.672092

H -10.770644 2.511140 -1.113095

H -8.973634 -1.751971 0.049151

H -10.689043 -2.167640 0.001225

H -11.280317 0.177814 -0.581680

H -10.164871 -0.470740 -1.767413

H -9.388928 1.310508 0.584043

H -7.675891 0.368965 -1.785836

H -10.076792 -1.074960 1.245509

H -9.102318 3.102294 -1.130372

H -9.663463 1.999473 -2.392153

H -7.088349 0.969411 1.156597

H -5.334806 0.026919 -1.225958

H -4.685614 0.596718 1.716590

H -2.997406 -0.341898 -0.716214

H -2.221899 0.188121 2.205847

H 5.165422 -2.566958 -1.223958

H 2.597931 -4.203601 0.131975

H 1.124366 -3.187346 -1.666298

H 1.388248 -1.461062 -1.938535

H 2.607854 -2.638008 -2.485187

H 1.786705 -1.473966 3.361311

H 3.281808 -0.984200 2.536790

H 2.170289 0.251393 3.160400

H 3.564713 3.076488 -0.814316

H 3.315790 2.637010 0.870802

H 6.341250 2.504688 -0.642084

H 7.583354 -0.026207 -1.718013

H 4.650129 5.137205 -0.213986

H 4.344607 4.759459 1.502669

H 6.010088 5.037145 0.935953

1a-c21 , delta G = 0.4311 kcal/mol, population = 17.79 %

C -2.629787 2.254052 -0.294179

C -1.605261 1.959937 0.809653

C -1.457315 0.526356 1.321513

C -2.021337 -0.516220 0.339319

C -3.341976 -0.188878 -0.222401

C -4.285737 -1.205963 -0.503322

C -5.529830 -0.834303 -1.000125

C -5.847140 0.515290 -1.213890

C -4.915122 1.502946 -0.947010

C -3.668622 1.160126 -0.443841

C -1.868112 2.428533 -1.623284

C -2.249341 0.421453 2.635772

C -4.026066 -2.682316 -0.275234

C -5.456658 -3.103152 1.593192

C 0.808698 0.348737 0.661431

C 2.131905 -0.060682 1.110732

C 3.194766 -0.002574 0.283691

C 4.527193 -0.389575 0.638231

C 5.567021 -0.307603 -0.222182

C 6.919952 -0.684689 0.092326

C 7.939107 -0.577089 -0.773058

C 9.359738 -0.955466 -0.497774

C 10.286954 0.272803 -0.589481

C 9.990280 1.347286 0.453195

C 9.816027 -2.051369 -1.473217

O -0.107922 0.230575 1.663556

O 0.502824 0.760470 -0.440514

O -0.984695 2.868519 1.309016

O -3.298237 3.467361 0.044081

O -1.433177 -1.564714 0.158719

O -5.194489 -3.364721 0.212427

O -6.524837 -1.718297 -1.308225

O -7.070569 0.847577 -1.688205

H 9.186358 -2.938835 -1.392575

H 10.072319 0.941703 1.464992

H 8.980285 1.745500 0.338772

H 11.318734 -0.073193 -0.478227

H 10.210621 0.697330 -1.596223

H 9.420204 -1.350098 0.522152

H 7.738783 -0.182700 -1.768572

H 10.691476 2.179738 0.368815

H 9.768096 -1.695038 -2.505509

H 10.847895 -2.342346 -1.265170

H 7.107819 -1.072372 1.090398

H 5.383893 0.069418 -1.225548

H 4.694773 -0.762578 1.644645

H 3.033750 0.362993 -0.726596

H 2.221396 -0.413430 2.129805

H -5.168648 2.536801 -1.129065

H -2.614684 4.083212 0.344716

H -1.366365 1.509599 -1.916740

H -2.590149 2.714210 -2.387977

H -1.118015 3.213170 -1.517659

H -2.179587 -0.597364 3.016176

H -1.817305 1.109449 3.360503

H -3.296546 0.674003 2.479641

H -3.776154 -3.169164 -1.219276

H -3.196896 -2.844292 0.407967

H -6.319176 -2.564663 -0.851310

H -7.584164 0.030788 -1.790374

H -5.631904 -2.037832 1.770132

H -6.348381 -3.666444 1.858261

H -4.615648 -3.437574 2.206658

1a-c37 , delta G = 0.7172 kcal/mol, population = 10.97 %

C 3.191267 -2.257627 0.149412

C 1.734383 -2.135301 0.614370

C 1.056133 -0.765372 0.579459

C 1.768713 0.227486 -0.356400

C 3.234660 0.260119 -0.229066

C 3.953284 1.467461 -0.403134

C 5.333126 1.445425 -0.235898

C 6.000258 0.258323 0.100782

C 5.292198 -0.920447 0.257156

C 3.913467 -0.925044 0.103878

C 3.196473 -2.896174 -1.254015

C 1.101313 -0.193210 2.006020

C 3.303322 2.793937 -0.745053

C 3.571137 3.961119 1.323880

C -0.619241 -1.444338 -0.946276

C -2.039599 -1.443380 -1.260318

C -2.991994 -0.896619 -0.477394

C -4.390154 -0.892211 -0.790760

C -5.320869 -0.333509 0.015104

C -6.732592 -0.306326 -0.264251

C -7.638870 0.260858 0.545648

C -9.113531 0.310903 0.300573

C -9.606666 1.766690 0.181402

C -9.013229 2.523455 -1.003898

C -9.863944 -0.423908 1.422062

O -0.324523 -0.874899 0.255517

O 0.247220 -1.912968 -1.659777

O 1.167417 -3.100882 1.068714

O 3.871893 -3.107044 1.070951

O 1.116974 0.982206 -1.051956

O 3.928877 3.892047 -0.058457

O 6.136948 2.541558 -0.373073

O 7.343516 0.270068 0.266669

H -9.549301 -1.466472 1.489801

H -7.926614 2.594748 -0.927331

H -9.413590 3.537432 -1.061972

H -10.697213 1.748879 0.097542

H -9.377793 2.294020 1.113740

H -9.322542 -0.196140 -0.647514

H -7.295772 0.730602 1.466773

H -9.244956 2.017947 -1.945005

H -9.675835 0.049740 2.389116

H -10.940447 -0.400860 1.239734

H -7.064244 -0.773994 -1.187894

H -4.995282 0.132807 0.941960

H -4.700986 -1.361471 -1.719880

H -2.692285 -0.425359 0.452844

H -2.289365 -1.920480 -2.199483

H 5.820435 -1.829940 0.502293

H 3.282499 -3.855719 1.241015

H 2.678548 -3.855559 -1.224090

H 2.699323 -2.259336 -1.981720

H 4.234307 -3.050839 -1.548290

H 0.543657 -0.854935 2.666579

H 2.127668 -0.113737 2.359550

H 0.643365 0.795651 2.010660

H 3.425058 3.007051 -1.808258

H 2.238286 2.787910 -0.530743

H 5.564415 3.338292 -0.305534

H 7.653096 1.180593 0.138358

H 3.876094 3.057241 1.859582

H 4.087625 4.822114 1.741749

H 2.491017 4.095285 1.428019

1a-c46 , delta G = 1.1119 kcal/mol, population = 5.63 %

C 2.741957 -2.261178 -0.260616

C 1.720707 -1.976112 0.848522

C 1.543538 -0.539943 1.343246

C 2.071969 0.502960 0.341720

C 3.393701 0.199914 -0.231050

C 4.309646 1.235481 -0.535688

C 5.556770 0.887400 -1.041823

C 5.903708 -0.456689 -1.243473

C 4.998814 -1.462753 -0.953150

C 3.750424 -1.143605 -0.439071

C 1.971659 -2.478441 -1.578487

C 2.346006 -0.398103 2.647506

C 4.019225 2.708266 -0.322515

C 5.450924 3.176277 1.533555

C -0.731283 -0.421778 0.701775

C -2.059842 -0.039256 1.158778

C -3.126183 -0.118076 0.337905

C -4.463585 0.243358 0.700483

C -5.505703 0.149948 -0.156006

C -6.862751 0.504862 0.166834

C -7.882530 0.401383 -0.698696

C -9.307040 0.753277 -0.406292

C -10.187441 -0.511875 -0.486611

C -11.644785 -0.283349 -0.088344

C -9.785567 1.853190 -1.366897

O 0.190692 -0.272904 1.694924

O -0.424929 -0.836188 -0.399037

O 1.125567 -2.892163 1.364915

O 3.445622 -3.450737 0.091167

O 1.457138 1.535007 0.155352

O 5.175545 3.420020 0.152131

O 6.527022 1.791229 -1.370740

O 7.129217 -0.765755 -1.727970

H -9.136635 2.728395 -1.308798

H -12.156163 0.388228 -0.779570

H -11.712814 0.151114 0.912440

H -10.139625 -0.911363 -1.505311

H -9.751330 -1.273868 0.164884

H -9.362489 1.138124 0.617697

H -7.680245 0.029533 -1.702179

H -12.193818 -1.226899 -0.080029

H -9.777772 1.493104 -2.399264

H -10.800899 2.171197 -1.128267

H -7.052498 0.875777 1.171069

H -5.320865 -0.216671 -1.162894

H -4.632957 0.607800 1.709725

H -2.963937 -0.479559 -0.673653

H -2.150182 0.312805 2.178027

H 5.275396 -2.492469 -1.124745

H 2.781153 -4.080401 0.405583

H 1.441693 -1.578617 -1.881582

H 2.693106 -2.757179 -2.346294

H 1.244614 -3.281455 -1.451846

H 2.253112 0.623191 3.016112

H 1.940166 -1.088175 3.385302

H 3.397866 -0.624646 2.483050

H 3.755447 3.179239 -1.270842

H 3.189836 2.860679 0.362523

H 6.308615 2.636227 -0.917362

H 7.622049 0.061647 -1.845712

H 5.647547 2.116179 1.719153

H 6.333256 3.758913 1.788018

H 4.607364 3.500141 2.149212

1a-c48 , delta G = 1.1314 kcal/mol, population = 5.45 %

C -3.188585 -2.230598 0.185051

C -1.722534 -2.256134 -0.264363

C -1.025493 -0.933339 -0.585320

C -1.745215 0.280661 0.031757

C -3.211078 0.292644 -0.159945

C -3.925135 1.494946 -0.365960

C -5.299104 1.430150 -0.561451

C -5.970367 0.199719 -0.576346

C -5.265159 -0.975233 -0.382290

C -3.892489 -0.937227 -0.178832

C -3.230964 -2.427773 1.713959

C -1.033137 -0.768192 -2.114354

C -3.287221 2.864755 -0.495664

C -3.868952 3.768423 1.640677

C 0.613996 -1.197931 1.101590

C 2.028283 -1.120375 1.433238

C 2.998226 -0.774461 0.562318

C 4.389601 -0.695419 0.895822

C 5.338922 -0.332646 0.004280

C 6.744325 -0.237347 0.300529

C 7.666521 0.145460 -0.594996

C 9.135047 0.264755 -0.337048

C 9.607900 1.722893 -0.503409

C 8.983495 2.691159 0.497689

C 9.918539 -0.667500 -1.273660

O 0.346668 -0.960264 -0.212537

O -0.268632 -1.464261 1.894890

O -1.163793 -3.314416 -0.431789

O -3.860471 -3.310134 -0.458932

O -1.098100 1.171880 0.543643

O -4.029513 3.865227 0.222577

O -6.081007 2.529924 -0.776220

O -7.308642 0.164244 -0.777130

H 9.748068 -0.398647 -2.319413

H 9.206162 2.388342 1.524159

H 7.897459 2.726707 0.393046

H 10.696915 1.739348 -0.401436

H 9.388737 2.049120 -1.525785

H 9.329046 -0.038099 0.697472

H 7.340913 0.399691 -1.603056

H 9.368462 3.703003 0.357005

H 10.990318 -0.592857 -1.077598

H 9.616672 -1.707370 -1.138948

H 7.055919 -0.488604 1.311215

H 5.034542 -0.089479 -1.010954

H 4.678655 -0.939224 1.914226

H 2.719975 -0.536216 -0.459072

H 2.257626 -1.357545 2.464429

H -5.792967 -1.917398 -0.378150

H -3.259990 -4.068519 -0.419591

H -2.725566 -3.356821 1.979837

H -2.742325 -1.608340 2.235413

H -4.277263 -2.482903 2.014011

H -2.050304 -0.776370 -2.501083

H -0.558942 0.177700 -2.374978

H -0.472863 -1.590098 -2.556812

H -2.254808 2.860294 -0.163221

H -3.325907 3.182114 -1.539768

H -5.590878 3.312653 -0.441447

H -7.624981 1.077337 -0.861385

H -4.223079 2.803452 2.014444

H -2.817882 3.893976 1.912318

H -4.460333 4.568817 2.079459

1a-c84 , delta G = 1.1521 kcal/mol, population = 5.26 %

C 2.732387 -2.332047 -0.343282

C 1.701738 -2.121793 0.773083

C 1.542385 -0.729651 1.383373

C 2.106377 0.383903 0.482992

C 3.426224 0.106422 -0.103725

C 4.356659 1.153946 -0.324139

C 5.591959 0.832058 -0.875111

C 5.918679 -0.500877 -1.174863

C 5.003886 -1.515698 -0.961560

C 3.760888 -1.220532 -0.421268

C 1.976750 -2.429172 -1.683742

C 2.324175 -0.713536 2.707485

C 4.039213 2.591562 0.013962

C 5.028086 4.667837 0.624903

C -0.719816 -0.520218 0.720641

C -2.049639 -0.151879 1.186251

C -3.106051 -0.163171 0.349078

C -4.444008 0.189909 0.718049

C -5.476737 0.160066 -0.154210

C -6.834176 0.508257 0.174412

C -7.845481 0.464525 -0.706012

C -9.270198 0.810159 -0.407043

C -10.158683 -0.441863 -0.566095

C -11.616708 -0.227017 -0.162946

C -9.732316 1.966473 -1.307512

O 0.188037 -0.466613 1.735357

O -0.403097 -0.851719 -0.405172

O 1.084096 -3.067045 1.203709

O 3.413186 -3.557740 -0.081368

O 1.516814 1.443074 0.383838

O 5.244979 3.283074 0.370093

O 6.573930 1.734481 -1.165704

O 7.137701 -0.784320 -1.690303

H -10.747896 2.277650 -1.061021

H -12.171785 -1.165720 -0.213218

H -12.119963 0.487351 -0.816132

H -10.107653 -0.781463 -1.606141

H -9.731419 -1.243663 0.042013

H -9.332339 1.136661 0.636702

H -7.635823 0.149459 -1.727280

H -11.687728 0.148157 0.861290

H -9.078448 2.832394 -1.192861

H -9.716511 1.666490 -2.358837

H -7.031873 0.821390 1.196654

H -5.283557 -0.146946 -1.179331

H -4.621720 0.493376 1.745888

H -2.934753 -0.460339 -0.681770

H -2.149275 0.131869 2.225730

H 5.266159 -2.531707 -1.216827

H 2.734535 -4.200378 0.170185

H 1.233720 -3.225794 -1.630809

H 1.467516 -1.498018 -1.920075

H 2.703892 -2.659465 -2.462109

H 2.242949 0.274760 3.159216

H 1.893201 -1.455679 3.377379

H 3.374629 -0.945024 2.540900

H 3.574387 3.087653 -0.847522

H 3.335048 2.657464 0.841374

H 6.357472 2.563706 -0.681689

H 7.636681 0.046225 -1.745953

H 4.627067 5.170789 -0.260561

H 4.332838 4.798282 1.459223

H 5.991916 5.099028 0.885639

1a-c74 , delta G = 1.2268 kcal/mol, population = 4.64 %

C 3.275319 -2.232908 0.199280

C 1.817634 -2.106712 0.661037

C 1.129552 -0.743149 0.583393

C 1.838955 0.226343 -0.378679

C 3.303915 0.273936 -0.243513

C 4.014306 1.482145 -0.442322

C 5.393103 1.474945 -0.265730

C 6.067297 0.301264 0.102423

C 5.367164 -0.878773 0.283357

C 3.989400 -0.897374 0.122766

C 3.285688 -2.904318 -1.188733

C 1.166483 -0.128104 1.992370

C 3.356510 2.795398 -0.818283

C 3.598732 4.005982 1.228721

C -0.539615 -1.483772 -0.921615

C -1.959837 -1.502289 -1.235978

C -2.913130 -0.913119 -0.485540

C -4.311421 -0.928373 -0.798160

C -5.241131 -0.316931 -0.030277

C -6.653634 -0.309047 -0.307481

C -7.557663 0.316713 0.461223

C -9.031661 0.356289 0.207499

C -9.473904 1.804153 -0.094225

C -10.944294 1.937462 -0.487498

C -9.788599 -0.242509 1.403006

O -0.249487 -0.871183 0.259937

O 0.330145 -1.973015 -1.616981

O 1.257945 -3.061325 1.146495

O 3.959831 -3.056690 1.140697

O 1.185889 0.954927 -1.100298

O 3.966695 3.912966 -0.149446

O 6.189087 2.574239 -0.422718

O 7.409456 0.327164 0.275675

H -9.446312 -1.257640 1.610066

H -11.173582 2.965089 -0.776088

H -11.612335 1.670181 0.332598

H -9.266667 2.426940 0.782689

H -8.848044 2.190710 -0.902989

H -9.243782 -0.251468 -0.678779

H -7.212196 0.850693 1.345413

H -11.181975 1.291737 -1.336878

H -9.627059 0.359526 2.301352

H -10.861396 -0.282271 1.212017

H -6.987944 -0.844461 -1.192750

H -4.913968 0.210877 0.862442

H -4.622883 -1.457554 -1.694296

H -2.614083 -0.388606 0.415975

H -2.208168 -2.029526 -2.148335

H 5.900604 -1.778048 0.553691

H 3.370772 -3.800085 1.333891

H 2.771301 -3.864690 -1.137661

H 2.788245 -2.286731 -1.932592

H 4.324924 -3.061871 -1.476574

H 0.610995 -0.773460 2.670706

H 2.191122 -0.031253 2.346404

H 0.701931 0.857388 1.965711

H 3.486464 2.987185 -1.884567

H 2.289578 2.784845 -0.613476

H 5.610429 3.367900 -0.375368

H 7.713102 1.236758 0.128063

H 4.103319 4.880583 1.632669

H 2.516539 4.130760 1.323065

H 3.909559 3.116330 1.784470

1a-c85 , delta G = 1.4351 kcal/mol, population = 3.26 %

C 2.749240 -2.278924 -0.072572

C 1.677796 -1.950769 0.976044

C 1.422274 -0.488437 1.343512

C 1.941101 0.490647 0.274474

C 3.295029 0.202287 -0.226668

C 4.175595 1.249983 -0.588523

C 5.454760 0.918887 -1.020853

C 5.867784 -0.419757 -1.092207

C 4.996778 -1.438950 -0.748547

C 3.716942 -1.136035 -0.307477

C 2.036960 -2.633894 -1.393353

C 2.172715 -0.204546 2.655353

C 3.810619 2.719915 -0.517991

C 5.156061 3.425270 1.328875

C -0.832143 -0.522934 0.624135

C -2.189540 -0.157880 1.003877

C -3.219797 -0.335208 0.152665

C -4.581806 0.003058 0.436933

C -5.583125 -0.180859 -0.453139

C -6.962662 0.150030 -0.210791

C -7.930689 -0.037282 -1.121026

C -9.400014 0.224677 -0.957624

C -9.778350 1.107018 0.237590

C -9.328588 2.562141 0.109830

C -10.144912 -1.123537 -0.909963

O 0.048102 -0.253485 1.629773

O -0.471628 -1.015619 -0.426932

O 1.104905 -2.847973 1.547787

O 3.486487 -3.405718 0.396579

O 1.289204 1.473573 -0.019743

O 4.915293 3.528147 -0.076600

O 6.395699 1.835411 -1.396260

O 7.123207 -0.711519 -1.505688

H -9.886814 -1.752323 -1.764176

H -9.648493 3.147117 0.974426

H -9.762123 3.021975 -0.781815

H -10.866820 1.080673 0.338611

H -9.384247 0.666187 1.158646

H -9.731827 0.739502 -1.868804

H -7.646460 -0.466122 -2.079919

H -8.244205 2.649313 0.030612

H -9.886793 -1.669056 0.000527

H -11.224807 -0.962523 -0.920118

H -7.198914 0.572124 0.760373

H -5.343499 -0.604964 -1.425304

H -4.804980 0.427009 1.411889

H -3.005193 -0.762385 -0.822903

H -2.330875 0.265460 1.989642

H 5.323810 -2.465489 -0.822327

H 2.836518 -4.032938 0.744478

H 2.796882 -2.939361 -2.112319

H 1.341155 -3.456974 -1.226254

H 1.480549 -1.785985 -1.785403

H 3.237923 -0.396943 2.541846

H 2.024520 0.838372 2.934431

H 1.771218 -0.849258 3.435331

H 3.557547 3.090074 -1.512822

H 2.951624 2.891941 0.124585

H 6.121586 2.706486 -1.031227

H 7.584177 0.124682 -1.677682

H 5.399706 2.398451 1.617508

H 5.999048 4.074560 1.553604

H 4.277451 3.758454 1.887895

1a-c88 , delta G = 1.4596 kcal/mol, population = 3.13 %

C 2.812451 -2.227662 -0.352512

C 1.787953 -2.027339 0.771867

C 1.546386 -0.618325 1.315191

C 2.024921 0.480514 0.349222

C 3.357715 0.256268 -0.234159

C 4.227165 1.341114 -0.500195

C 5.486647 1.066807 -1.020865

C 5.889862 -0.252194 -1.276368

C 5.030149 -1.306777 -1.022988

C 3.771313 -1.061715 -0.493807

C 2.044328 -2.428420 -1.674330

C 2.345106 -0.483985 2.622522

C 3.875115 2.790548 -0.228439

C 5.289597 3.241050 1.645146

C -0.734332 -0.590577 0.682949

C -2.078935 -0.294329 1.156261

C -3.144441 -0.413385 0.338949

C -4.498687 -0.144635 0.718621

C -5.539374 -0.279398 -0.134196

C -6.914064 -0.021911 0.207468

C -7.923902 -0.165806 -0.665002

C -9.380248 0.093157 -0.419304

C -9.880911 1.156166 -1.424457

C -11.398623 1.327300 -1.449683

C -9.710565 0.458199 1.026888

O 0.183575 -0.426021 1.677471

O -0.412059 -0.953524 -0.431459

O 1.241132 -2.987054 1.261900

O 3.567899 -3.398368 -0.048835

O 1.364422 1.489817 0.198148

O 5.001051 3.530516 0.275300

O 6.416626 2.023739 -1.314257

O 7.125649 -0.489033 -1.775325

H -9.258694 1.414317 1.302920

H -11.895809 0.374306 -1.648942

H -11.694045 2.027926 -2.233047

H -9.400065 2.110619 -1.187739

H -9.536215 0.877140 -2.424262

H -9.908001 -0.839301 -0.665299

H -7.680589 -0.487839 -1.675739

H -11.781671 1.712995 -0.503822

H -10.787379 0.544982 1.168352

H -9.347445 -0.301899 1.720640

H -7.112726 0.300720 1.224182

H -5.338656 -0.604813 -1.152231

H -4.682956 0.179696 1.738856

H -2.967626 -0.737167 -0.682964

H -2.182676 0.023589 2.185414

H 5.349972 -2.316282 -1.234829

H 2.933088 -4.064626 0.251250

H 1.477415 -1.540405 -1.943136

H 2.772698 -2.650720 -2.453931

H 1.350390 -3.263808 -1.574103

H 1.975041 -1.218044 3.336292

H 3.406004 -0.654214 2.448422

H 2.204850 0.518156 3.026887

H 3.591053 3.288000 -1.157144

H 3.040743 2.880072 0.461679

H 6.164429 2.839639 -0.826396

H 7.581882 0.362912 -1.861327

H 6.146727 3.849746 1.923592

H 4.434298 3.502231 2.274261

H 5.531844 2.183506 1.785938

1a-c70 , delta G = 1.5142 kcal/mol, population = 2.85 %

C -2.697381 2.278137 -0.219137

C -1.701748 1.991242 0.912364

C -1.529383 0.553629 1.403608

C -2.025073 -0.488124 0.383876

C -3.328313 -0.189409 -0.230911

C -4.222188 -1.230526 -0.581737

C -5.449987 -0.888219 -1.136555

C -5.802235 0.455414 -1.334668

C -4.919644 1.466346 -0.997785

C -3.688528 1.152904 -0.440055

C -1.894283 2.520969 -1.512890

C -2.363246 0.400253 2.686476

C -3.920334 -2.702278 -0.375005

C -5.456262 -3.244195 1.375834

C 0.759124 0.441317 0.814111

C 2.076243 0.050659 1.296784

C 3.155524 0.105075 0.491097

C 4.481268 -0.275274 0.876686

C 5.534708 -0.220670 0.030808

C 6.879340 -0.602122 0.374857

C 7.908905 -0.545512 -0.483782

C 9.318161 -0.936277 -0.163893

C 10.267903 0.285310 -0.172159

C 10.404399 1.008210 -1.512104

C 9.798081 -2.044892 -1.113870

O -0.183946 0.292244 1.787155

O 0.477255 0.859457 -0.291862

O -1.123124 2.907157 1.447461

O -3.424025 3.455243 0.128892

O -1.401263 -1.518069 0.215266

O -5.090429 -3.445279 0.008393

O -6.396675 -1.794929 -1.520824

O -7.010085 0.758241 -1.865009

H 9.739287 -1.729969 -2.157405

H 9.448752 1.410679 -1.853632

H 10.788356 0.348568 -2.292181

H 9.920670 0.992595 0.585882

H 11.253739 -0.060607 0.153513

H 9.330556 -1.335920 0.854726

H 7.718876 -0.196793 -1.496535

H 11.097900 1.846282 -1.421374

H 10.835922 -2.309702 -0.899192

H 9.186178 -2.941365 -1.002380

H 7.049971 -0.955807 1.388794

H 5.369386 0.130949 -0.984795

H 4.630834 -0.625957 1.893902

H 3.013397 0.456869 -0.526861

H 2.146161 -0.293849 2.320214

H -5.198841 2.495270 -1.169868

H -2.774052 4.092528 0.457697

H -1.345237 1.631080 -1.811552

H -2.597355 2.800518 -2.297230

H -1.180805 3.330939 -1.356462

H -1.981756 1.090263 3.437233

H -3.412333 0.619441 2.496304

H -2.271729 -0.621874 3.053028

H -3.582720 -3.150606 -1.310771

H -3.136348 -2.852214 0.362169

H -6.180168 -2.650238 -1.085651

H -7.487967 -0.072705 -2.015151

H -4.642552 -3.559911 2.034278

H -5.695262 -2.195128 1.574184

H -6.334837 -3.857643 1.561646

1a-c1 , delta G = 1.5512 kcal/mol, population = 2.68 %

C -3.729050 -0.866526 1.665502

C -3.942461 -1.965193 0.616416

C -2.966039 -2.005378 -0.560866

C -3.186938 -0.616547 -1.224348

C -2.891901 0.523599 -0.335293

C -2.396143 1.732149 -0.868490

C -2.214080 2.809040 -0.011705

C -2.541473 2.705967 1.349338

C -3.020182 1.514267 1.862030

C -3.178270 0.407959 1.034901

C -2.801781 -1.407433 2.770646

C -3.195386 -3.164011 -1.500011

C -2.013363 1.894003 -2.325280

C 0.369875 1.997150 -2.114699

C -0.583036 -1.638517 -0.665025

C 0.641513 -1.750931 0.113627

C 1.822369 -1.343589 -0.396195

C 3.072481 -1.386387 0.298049

C 4.229798 -0.957501 -0.255848

C 5.507417 -0.972478 0.404794

C 6.639653 -0.535392 -0.167009

C 7.991383 -0.526677 0.472070

C 8.532895 0.911401 0.598451

C 7.694487 1.808616 1.505072

C 8.964523 -1.401990 -0.332737

O -1.657656 -2.073050 0.055692

O -0.690768 -1.205761 -1.794767

O -4.894986 -2.705082 0.689304

O -5.000551 -0.554028 2.240163

O -3.655862 -0.521226 -2.337884

O -0.826932 2.694551 -2.476018

O -1.721308 4.020279 -0.404406

O -2.369795 3.775960 2.163111

H 9.080551 -1.017690 -1.349405

H 8.137228 2.802834 1.590098

H 7.622021 1.387681 2.511290

H 9.555814 0.854185 0.981514

H 8.602225 1.350613 -0.402396

H 7.899405 -0.943939 1.480466

H 6.594896 -0.148550 -1.184403

H 6.678589 1.924909 1.122619

H 9.949426 -1.409685 0.138842

H 8.607608 -2.430933 -0.399036

H 5.537586 -1.357847 1.420680

H 4.208244 -0.569986 -1.271700

H 3.077236 -1.777227 1.311451

H 1.828673 -0.950264 -1.408849

H 0.562656 -2.155265 1.114095

H -3.275026 1.465458 2.911176

H -5.534200 -1.361746 2.185759

H -3.211696 -2.336823 3.169355

H -1.797635 -1.589904 2.398413

H -2.761667 -0.676064 3.576446

H -3.045692 -4.102579 -0.968327

H -4.212238 -3.135859 -1.884626

H -2.503780 -3.097826 -2.336717

H -1.864232 0.929178 -2.802966

H -2.794550 2.428019 -2.867802

H -1.273874 3.887117 -1.270472

H -1.999993 4.499268 1.633228

H 0.359596 1.709423 -1.059366

H 0.481084 1.097412 -2.722555

H 1.196885 2.679339 -2.298716

1a-c77 , delta G = 1.9051 kcal/mol, population = 1.47 %

C 2.775724 -2.226179 -0.412780

C 1.741801 -2.079867 0.711191

C 1.472986 -0.694367 1.300087

C 1.939876 0.444426 0.375938

C 3.278762 0.261240 -0.207768

C 4.131053 1.368902 -0.432844

C 5.396008 1.133415 -0.959026

C 5.821648 -0.169171 -1.258842

C 4.978828 -1.245795 -1.045259

C 3.714751 -1.039929 -0.511604

C 2.017787 -2.398643 -1.744344

C 2.259959 -0.590593 2.617305

C 3.754227 2.802125 -0.112226

C 5.161383 3.213674 1.776060

C -0.803168 -0.682635 0.651281

C -2.156960 -0.433710 1.125509

C -3.212209 -0.523293 0.291239

C -4.574396 -0.298403 0.670766

C -5.603151 -0.389132 -0.202196

C -6.984699 -0.171538 0.138614

C -7.981910 -0.256699 -0.755326

C -9.444192 -0.040377 -0.506423

C -9.991776 1.051534 -1.458145

C -9.428834 2.450168 -1.211709

C -9.802200 0.225458 0.955340

O 0.104289 -0.538465 1.658512

O -0.465686 -0.994704 -0.474028

O 1.207181 -3.064227 1.164391

O 3.550689 -3.392222 -0.141533

O 1.265398 1.448694 0.256381

O 4.867196 3.544164 0.416729

O 6.311124 2.114615 -1.216993

O 7.062537 -0.367981 -1.761740

H -9.512062 -0.612630 1.591375

H -8.339522 2.458366 -1.289511

H -9.693805 2.822861 -0.220695

H -9.777838 0.750669 -2.487995

H -11.080974 1.069965 -1.361684

H -9.944805 -0.970693 -0.806808

H -7.723361 -0.496710 -1.785143

H -9.822075 3.157209 -1.944597

H -9.310023 1.120683 1.338442

H -10.879441 0.367891 1.058043

H -7.200825 0.072131 1.173663

H -5.386187 -0.641953 -1.237392

H -4.775244 -0.044345 1.707686

H -3.019884 -0.785042 -0.745558

H -2.276006 -0.173420 2.169083

H 5.315796 -2.241953 -1.291038

H 2.926128 -4.080487 0.128869

H 1.338438 -3.248995 -1.674083

H 1.436076 -1.512769 -1.987413

H 2.753487 -2.582984 -2.526955

H 1.896440 -1.353382 3.303787

H 3.324716 -0.738554 2.446178

H 2.100790 0.395500 3.052984

H 3.461241 3.325794 -1.023549

H 2.918706 2.853600 0.580340

H 6.043785 2.910280 -0.704429

H 7.504824 0.493870 -1.816020

H 6.006546 3.829008 2.075693

H 4.301246 3.436597 2.413243

H 5.424207 2.156842 1.880703

# Table S70. Geometry data of conformers of structure 1a'

1a'-c45 , delta G = 0.0000 kcal/mol, population = 40.17 %

C -2.758936 -2.322399 -0.414045

C -1.722192 -2.201124 0.710560

C -1.502090 -0.843688 1.376480

C -1.997876 0.328964 0.513620

C -3.322108 0.143404 -0.096421

C -4.191787 1.247248 -0.288478

C -5.429476 1.015638 -0.878480

C -5.817744 -0.284392 -1.242916

C -4.963199 -1.354676 -1.053002

C -3.719460 -1.150410 -0.473132

C -2.001749 -2.438444 -1.751553

C -2.297429 -0.837497 2.692802

C -3.811498 2.650283 0.123617

C -4.717777 4.747485 0.792703

C 0.776550 -0.721261 0.741101

C 2.118066 -0.448386 1.233156

C 3.180449 -0.464606 0.401494

C 4.531398 -0.205391 0.796309

C 5.564330 -0.230010 -0.077353

C 6.935974 0.027385 0.272385

C 7.941205 0.005252 -0.616255

C 9.380857 0.265552 -0.307028

C 9.897898 1.496559 -1.077982

C 9.193427 2.798109 -0.704349

C 10.227478 -0.973218 -0.638765

O -0.138742 -0.664875 1.749637

O 0.452126 -0.983568 -0.402221

O -1.143484 -3.188144 1.100755

O -3.517527 -3.508698 -0.179687

O -1.351200 1.358144 0.455588

O -4.990481 3.380778 0.489137

O -6.355044 1.980307 -1.148686

O -7.037494 -0.481902 -1.796311

H 11.278100 -0.790103 -0.403625

H 9.294317 3.003130 0.364636

H 8.126713 2.752985 -0.932388

H 10.970355 1.589889 -0.884150

H 9.792528 1.309256 -2.151849

H 9.472188 0.473314 0.764308

H 7.713069 -0.219097 -1.657548

H 9.618020 3.643724 -1.248939

H 10.156652 -1.216618 -1.702044

H 9.894587 -1.842613 -0.069503

H 7.150150 0.253300 1.313893

H 5.358471 -0.456418 -1.120829

H 4.720344 0.020020 1.841915

H 3.003697 -0.689711 -0.646622

H 2.224808 -0.230603 2.287741

H -5.272918 -2.343321 -1.357947

H -2.881291 -4.214518 0.003429

H -1.313295 -3.283644 -1.714062

H -1.431404 -1.536559 -1.961269

H -2.734696 -2.600806 -2.541386

H -1.910366 -1.622414 3.340422

H -3.355544 -1.012855 2.508017

H -2.176694 0.129455 3.180273

H -3.124337 2.642407 0.967769

H -3.306317 3.162908 -0.704588

H -6.104544 2.776553 -0.624892

H -7.494182 0.374189 -1.826337

H -5.665706 5.209500 1.058111

H -4.026784 4.819472 1.637217

H -4.287019 5.260580 -0.072246

1a'-c25 , delta G = 0.3144 kcal/mol, population = 23.62 %

C -2.745765 -2.244418 -0.365658

C -1.719960 -2.046529 0.758768

C -1.491913 -0.641963 1.317584

C -1.969562 0.460806 0.356986

C -3.300007 0.237639 -0.229179

C -4.172888 1.321905 -0.484840

C -5.428548 1.049092 -1.015542

C -5.823927 -0.268648 -1.289284

C -4.960794 -1.322633 -1.044634

C -3.706493 -1.079432 -0.504103

C -1.976674 -2.442240 -1.686992

C -2.301496 -0.523048 2.620237

C -3.830663 2.768422 -0.185228

C -5.262995 3.178343 1.685787

C 0.797475 -0.593449 0.704653

C 2.132699 -0.286044 1.193328

C 3.210759 -0.388830 0.388496

C 4.555130 -0.102271 0.787004

C 5.608451 -0.219808 -0.054070

C 6.972996 0.062189 0.304586

C 8.001627 -0.055706 -0.549000

C 9.434692 0.224476 -0.226087

C 9.990505 1.355887 -1.113365

C 9.294639 2.698730 -0.906711

C 10.275569 -1.051464 -0.388179

O -0.131988 -0.444893 1.691139

O 0.488425 -0.951915 -0.416365

O -1.157719 -3.005597 1.233306

O -3.502260 -3.415684 -0.063998

O -1.307794 1.470493 0.207639

O -4.964198 3.493082 0.321581

O -6.360574 2.005547 -1.299952

O -7.056370 -0.506385 -1.796745

H 11.320877 -0.851933 -0.142896

H 9.369481 3.020195 0.135423

H 8.233980 2.640708 -1.159049

H 11.058370 1.458371 -0.899953

H 9.912163 1.051739 -2.162550

H 9.495371 0.551007 0.817420

H 7.799839 -0.382310 -1.568361

H 9.745992 3.473731 -1.529284

H 10.233761 -1.414876 -1.418211

H 9.915388 -1.846954 0.266166

H 7.161194 0.390303 1.323673

H 5.426579 -0.546736 -1.075048

H 4.721638 0.223182 1.809801

H 3.053563 -0.711234 -0.637058

H 2.222122 0.028911 2.224740

H -5.275272 -2.330399 -1.272288

H -2.866469 -4.100144 0.188891

H -1.286467 -3.281271 -1.590723

H -1.406860 -1.554194 -1.950157

H -2.703465 -2.656774 -2.470114

H -1.931420 -1.259110 3.331941

H -3.359729 -0.699205 2.437543

H -2.173704 0.477610 3.031980

H -3.004088 2.849173 0.515240

H -3.541247 3.282909 -1.102980

H -6.117069 2.813840 -0.793964

H -7.521847 0.342288 -1.867344

H -6.125948 3.777589 1.966061

H -5.500167 2.117532 1.805676

H -4.413880 3.432827 2.325243

1a'-c46 , delta G = 0.4713 kcal/mol, population = 18.12 %

C -2.803018 -2.236630 -0.319368

C -1.777824 -2.003818 0.799005

C -1.563180 -0.584807 1.325962

C -2.050199 0.491300 0.340091

C -3.378433 0.243134 -0.241020

C -4.260544 1.313455 -0.522463

C -5.513759 1.017317 -1.046450

C -5.897857 -0.309932 -1.288440

C -5.025809 -1.350344 -1.018550

C -3.773610 -1.083536 -0.484397

C -2.033084 -2.459223 -1.636299

C -2.374498 -0.444224 2.625279

C -3.930529 2.769599 -0.257886

C -5.366165 3.213315 1.602825

C 0.725643 -0.527874 0.712319

C 2.057672 -0.195975 1.193592

C 3.137152 -0.310504 0.392244

C 4.479117 -0.003412 0.783519

C 5.533920 -0.140937 -0.052663

C 6.897622 0.154857 0.298603

C 7.928257 0.002992 -0.547617

C 9.360121 0.297466 -0.230650

C 9.881959 1.416891 -1.156244

C 11.300939 1.881605 -0.832544

C 10.198184 -0.986001 -0.345249

O -0.205373 -0.366400 1.695288

O 0.420246 -0.914999 -0.400164

O -1.205775 -2.946476 1.294169

O -3.549475 -3.406713 0.010498

O -1.396955 1.502846 0.167560

O -5.070214 3.496805 0.231144

O -6.453780 1.958733 -1.353551

O -7.128086 -0.570288 -1.790102

H 9.802116 -1.766633 0.306161

H 11.582333 2.726624 -1.464034

H 12.035294 1.091182 -0.994541

H 9.833216 1.068088 -2.193272

H 9.199381 2.267916 -1.082951

H 9.416929 0.658125 0.801844

H 7.728485 -0.363485 -1.553482

H 11.378897 2.202981 0.209372

H 11.235421 -0.805867 -0.061147

H 10.188352 -1.362861 -1.371506

H 7.083733 0.517859 1.306282

H 5.354342 -0.499657 -1.063325

H 4.642575 0.352634 1.796554

H 2.983209 -0.660952 -0.624561

H 2.143696 0.145415 2.216848

H -5.331693 -2.365924 -1.221829

H -2.907763 -4.080395 0.276897

H -2.758521 -2.697776 -2.413703

H -1.336261 -3.290334 -1.520863

H -1.470509 -1.573073 -1.920650

H -1.997944 -1.160646 3.353421

H -3.430940 -0.634219 2.446255

H -2.256303 0.566581 3.014513

H -3.104803 2.874263 0.440441

H -3.645094 3.264110 -1.187799

H -6.216877 2.780830 -0.867064

H -7.600831 0.272409 -1.881087

H -6.234770 3.811181 1.868156

H -5.593281 2.153636 1.749122

H -4.519554 3.491629 2.235610

1a'-c67 , delta G = 0.7229 kcal/mol, population = 11.84 %

C -3.073946 -2.378209 0.102763

C -1.605970 -2.208940 0.515286

C -1.009155 -0.803933 0.581472

C -1.812863 0.223363 -0.233374

C -3.271130 0.158548 -0.062714

C -4.060820 1.335061 -0.123070

C -5.436845 1.213753 0.036478

C -6.022025 -0.039093 0.283969

C -5.244713 -1.181297 0.340323

C -3.870103 -1.089637 0.176497

C -3.105983 -2.925290 -1.337876

C -1.025876 -0.361354 2.054244

C -3.454118 2.699066 -0.355695

C -3.786345 5.021720 0.046993

C 0.636652 -1.234479 -1.065643

C 2.035531 -1.105198 -1.437497

C 2.985639 -0.557181 -0.650331

C 4.360907 -0.416934 -1.023959

C 5.285079 0.144179 -0.210716

C 6.674546 0.310465 -0.545928

C 7.571546 0.867742 0.281927

C 9.022856 1.071035 -0.016579

C 9.906684 0.285859 0.973077

C 9.728103 -1.227785 0.890191

C 9.367441 2.568062 0.022333

O 0.361666 -0.803389 0.196196

O -0.232412 -1.691776 -1.786558

O -0.961551 -3.176101 0.848174

O -3.669393 -3.320980 0.994181

O -1.231722 1.076109 -0.878568

O -4.243443 3.696067 0.308084

O -6.320439 2.251033 -0.022440

O -7.362221 -0.123247 0.456193

H 8.774749 3.128340 -0.702608

H 9.946639 -1.590834 -0.117478

H 8.705078 -1.522118 1.132599

H 10.950227 0.544491 0.771641

H 9.692398 0.632940 1.989456

H 9.224536 0.692707 -1.024223

H 7.236287 1.210152 1.260184

H 10.398276 -1.740014 1.583303

H 10.424201 2.721549 -0.206234

H 9.172056 2.984067 1.013997

H 6.995948 -0.039271 -1.523601

H 4.970989 0.500010 0.767619

H 4.659007 -0.777424 -2.004282

H 2.701613 -0.191670 0.331081

H 2.272632 -1.477695 -2.426081

H -5.716158 -2.137351 0.513526

H -3.064615 -4.074619 1.047152

H -4.145054 -3.119841 -1.602159

H -2.536065 -3.853650 -1.392641

H -2.677364 -2.213858 -2.039812

H -0.410064 -1.046612 2.634269

H -2.039998 -0.366585 2.449096

H -0.619595 0.646757 2.130438

H -2.433421 2.747535 0.019309

H -3.418579 2.918199 -1.430238

H -5.798637 3.081224 0.075163

H -7.726981 0.775072 0.407678

H -4.442917 5.697233 0.590163

H -2.759119 5.146685 0.400570

H -3.830486 5.245715 -1.023005

1a'-c68 , delta G = 1.1007 kcal/mol, population = 6.25 %

C -3.215853 -2.267219 0.274054

C -1.747422 -2.093518 0.685657

C -1.102762 -0.712102 0.572673

C -1.861819 0.218603 -0.388207

C -3.322535 0.229892 -0.218703

C -4.068779 1.414690 -0.426262

C -5.445093 1.371016 -0.236016

C -6.079893 0.184280 0.160298

C -5.343675 -0.971337 0.354801

C -3.967595 -0.953718 0.179163

C -3.248965 -2.982525 -1.091011

C -1.127223 -0.073696 1.971924

C -3.447663 2.740989 -0.820212

C -3.734094 3.978988 1.206492

C 0.555912 -1.415672 -0.965521

C 1.964571 -1.377064 -1.320551

C 2.914227 -0.740745 -0.602137

C 4.300234 -0.693557 -0.958773

C 5.223593 -0.039685 -0.216957

C 6.624653 0.034120 -0.536937

C 7.521235 0.691920 0.214557

C 8.985642 0.791219 -0.073229

C 9.787802 0.112889 1.057627

C 11.293145 0.051206 0.803971

C 9.386049 2.262825 -0.264149

O 0.272278 -0.804893 0.218233

O -0.313275 -1.946737 -1.633459

O -1.142356 -3.028142 1.156405

O -3.852000 -3.076542 1.262147

O -1.247561 0.947051 -1.144618

O -4.093542 3.850360 -0.173295

O -6.273723 2.443176 -0.403023

O -7.420558 0.172633 0.348540

H 10.438157 2.352382 -0.536326

H 11.744075 1.044327 0.780498

H 11.509195 -0.437536 -0.149646

H 9.590792 0.641923 1.996129

H 9.403398 -0.902369 1.187893

H 9.191101 0.251513 -1.003814

H 7.177430 1.200398 1.114189

H 11.792184 -0.517714 1.590931

H 9.225011 2.828866 0.657312

H 8.793630 2.726207 -1.054692

H 6.956280 -0.475894 -1.437949

H 4.899385 0.470649 0.686872

H 4.608180 -1.207643 -1.864716

H 2.621269 -0.224609 0.306276

H 2.210092 -1.898905 -2.236869

H -5.848831 -1.880276 0.646355

H -3.264770 -3.828330 1.425183

H -2.714261 -3.930934 -1.024506

H -2.784690 -2.376925 -1.865774

H -4.291216 -3.169764 -1.348114

H -0.533777 -0.687177 2.647709

H -2.145622 -0.004640 2.349034

H -0.699436 0.926932 1.917185

H -2.382830 2.764799 -0.605155

H -3.575005 2.911678 -1.890412

H -5.716707 3.254095 -0.375093

H -7.754556 1.070962 0.195755

H -2.656500 4.134647 1.301785

H -4.265539 4.846123 1.591137

H -4.022686 3.091167 1.776053

# Table S71. Geometry data of conformers of structure 2a

2a-c1 , delta G = 0.0000 kcal/mol, population = 44.84 %

C 3.177798 -1.776793 -0.724106

C 2.115034 -1.946702 0.373383

C 1.887832 -0.842704 1.409426

C 2.395777 0.532876 0.969683

C 3.577270 0.573168 0.139717

C 4.331141 1.769946 0.101635

C 5.557351 1.802271 -0.547662

C 6.039545 0.654435 -1.170542

C 5.283197 -0.520654 -1.181108

C 4.064302 -0.565998 -0.537341

C 2.432561 -1.656719 -2.067854

C 2.657578 -1.238378 2.679451

O 3.934017 2.910790 0.699668

C -0.348807 -0.456327 0.781598

C -1.720619 -0.398248 1.260620

C -2.733456 -0.072922 0.431775

C -4.109793 0.001857 0.818107

C -5.095533 0.340949 -0.043474

C -6.489905 0.421802 0.302707

C -7.450822 0.764746 -0.568179

C -8.910398 0.866950 -0.258452

C -9.730800 -0.101188 -1.133291

C -9.401396 -1.573123 -0.899555

C -9.393786 2.312713 -0.452719

O 0.513770 -0.782743 1.785214

O 0.045025 -0.257168 -0.352990

O 1.510248 -2.989615 0.450235

O 4.004212 -2.940567 -0.716662

O 1.885789 1.543521 1.461182

O 6.333094 2.927337 -0.594717

O 7.237548 0.672708 -1.797675

H -9.252654 2.632146 -1.488468

H -8.358979 -1.791377 -1.139494

H -10.030177 -2.218301 -1.516163

H -10.791214 0.075449 -0.931095

H -9.570750 0.154712 -2.186260

H -9.060581 0.587747 0.789935

H -7.167054 0.995875 -1.594230

H -9.563693 -1.848019 0.145988

H -10.456971 2.394773 -0.216875

H -8.844941 3.001736 0.191104

H -6.760741 0.186563 1.328841

H -4.831580 0.569994 -1.073158

H -4.358247 -0.227428 1.850408

H -2.492751 0.148884 -0.604114

H -1.889489 -0.626182 2.304817

H 5.661846 -1.382686 -1.709834

H 3.414889 -3.704897 -0.779392

H 3.171196 -1.614092 -2.867474

H 1.789378 -2.526388 -2.209366

H 1.814129 -0.761732 -2.091271

H 2.279574 -2.194701 3.036938

H 3.723009 -1.327655 2.471028

H 2.503514 -0.475018 3.440780

H 5.884795 3.640391 -0.118253

H 7.629135 1.554443 -1.707374

H 3.044322 2.729927 1.096033

2a-c4 , delta G = 0.3621 kcal/mol, population = 24.32 %

C 3.449604 -1.882728 -0.027181

C 1.967107 -1.812810 0.374311

C 1.315576 -0.467612 0.706872

C 2.064484 0.740508 0.139318

C 3.503225 0.652243 0.046852

C 4.246687 1.854338 -0.021261

C 5.632108 1.823624 0.051813

C 6.290142 0.603900 0.182988

C 5.570583 -0.593648 0.205992

C 4.192969 -0.576398 0.137194

C 3.504343 -2.315473 -1.505198

C 1.301340 -0.330229 2.237122

O 3.674585 3.070077 -0.128942

C -0.254804 -0.599384 -1.040488

C -1.653589 -0.572040 -1.431074

C -2.680968 -0.406243 -0.572150

C -4.059447 -0.389827 -0.960444

C -5.068920 -0.218355 -0.077227

C -6.464540 -0.201209 -0.427697

C -7.450897 -0.019983 0.463073

C -8.913358 -0.000323 0.149967

C -9.536092 1.366736 0.496548

C -8.973856 2.525297 -0.322736

C -9.632977 -1.129350 0.903782

O -0.049991 -0.457613 0.298702

O 0.678768 -0.742513 -1.810105

O 1.341429 -2.839268 0.494857

O 4.079389 -2.860722 0.801081

O 1.441098 1.786000 -0.065333

O 6.394120 2.958253 0.010818

O 7.639528 0.564139 0.263499

H -10.699538 -1.125451 0.669107

H -9.467694 3.464057 -0.064563

H -9.119886 2.353505 -1.392356

H -10.616373 1.295359 0.340119

H -9.391296 1.559431 1.564923

H -9.037624 -0.165865 -0.925556

H -7.188568 0.125213 1.510372

H -7.902895 2.650972 -0.152466

H -9.225813 -2.105274 0.634853

H -9.525139 -1.004004 1.984320

H -6.714055 -0.343552 -1.476082

H -4.824956 -0.083036 0.973762

H -4.288804 -0.524407 -2.013648

H -2.462550 -0.278501 0.482986

H -1.823214 -0.699738 -2.492607

H 6.109621 -1.527442 0.266063

H 3.559450 -3.672523 0.723319

H 3.046381 -1.565614 -2.147088

H 4.548299 -2.450666 -1.785735

H 2.966117 -3.255760 -1.632155

H 0.834265 0.616240 2.505648

H 0.729773 -1.155340 2.658694

H 2.315329 -0.355626 2.634263

H 5.812459 3.726506 -0.078242

H 7.985905 1.468100 0.221582

H 2.697066 2.920399 -0.188374

2a-c6 , delta G = 0.8810 kcal/mol, population = 10.12 %

C 3.258199 -1.744401 -0.796539

C 2.198406 -1.962521 0.295487

C 1.968877 -0.902555 1.375951

C 2.468209 0.492165 0.990903

C 3.645429 0.573037 0.157807

C 4.391219 1.775526 0.163963

C 5.613525 1.842567 -0.489924

C 6.099834 0.724173 -1.161215

C 5.351272 -0.454657 -1.215044

C 4.136234 -0.534589 -0.567237

C 2.510848 -1.582742 -2.134595

C 2.743647 -1.346148 2.626891

O 3.989680 2.888808 0.809177

C -0.271161 -0.507888 0.766991

C -1.643083 -0.480668 1.248127

C -2.660993 -0.148938 0.428002

C -4.038332 -0.106471 0.815390

C -5.030265 0.230818 -0.039815

C -6.425990 0.277796 0.307504

C -7.394465 0.616282 -0.557151

C -8.856079 0.671082 -0.243414

C -9.615276 -0.362236 -1.102510

C -11.102197 -0.476843 -0.770677

C -9.385821 2.098628 -0.452449

O 0.595323 -0.864571 1.756803

O 0.120023 -0.262854 -0.359551

O 1.598976 -3.010745 0.331806

O 4.093637 -2.901590 -0.831045

O 1.954874 1.478977 1.525576

O 6.381044 2.974174 -0.495911

O 7.294133 0.775830 -1.793557

H -10.435755 2.176247 -0.168415

H -11.642317 0.444531 -0.993338

H -11.250195 -0.704019 0.288277

H -9.490011 -0.102159 -2.159081

H -9.139327 -1.336974 -0.965633

H -8.992002 0.400621 0.809264

H -7.116978 0.873524 -1.578448

H -11.565209 -1.277412 -1.350801

H -8.821357 2.813311 0.148436

H -9.296713 2.392748 -1.501705

H -6.691143 0.020669 1.330016

H -4.770830 0.484929 -1.064751

H -4.282650 -0.361838 1.842548

H -2.424021 0.102846 -0.601883

H -1.808295 -0.740314 2.285482

H 5.733163 -1.292047 -1.779861

H 3.509856 -3.667472 -0.920794

H 1.874547 -2.452019 -2.306413

H 1.885323 -0.692426 -2.125888

H 3.248183 -1.505840 -2.932837

H 2.370212 -2.317690 2.946108

H 3.808791 -1.422896 2.412118

H 2.589064 -0.615157 3.419257

H 5.930240 3.664222 0.011094

H 7.680521 1.655823 -1.670026

H 3.103177 2.686070 1.202228

2a-c12 , delta G = 0.9821 kcal/mol, population = 8.53 %

C 3.193881 -1.749197 -0.781409

C 2.160353 -1.959343 0.336980

C 1.949625 -0.886758 1.408878

C 2.431064 0.505764 0.993742

C 3.589537 0.581945 0.134215

C 4.328336 1.788411 0.107901

C 5.535774 1.853053 -0.573281

C 6.014023 0.728071 -1.239370

C 5.271378 -0.455546 -1.260770

C 4.070949 -0.532837 -0.586075

C 2.415073 -1.605274 -2.103493

C 2.756790 -1.309350 2.646444

O 3.934566 2.908145 0.746694

C -0.306213 -0.512571 0.848053

C -1.666947 -0.485945 1.360247

C -2.703378 -0.161746 0.560609

C -4.072131 -0.119316 0.977361

C -5.082491 0.213306 0.142133

C -6.470664 0.258986 0.518655

C -7.457830 0.592450 -0.327001

C -8.911370 0.643115 0.026408

C -9.716822 -0.454314 -0.709616

C -9.736069 -0.350817 -2.234570

C -9.480998 2.047033 -0.230653

O 0.585161 -0.852185 1.821579

O 0.057033 -0.279775 -0.290371

O 1.567685 -3.010122 0.400124

O 4.033604 -2.902877 -0.822478

O 1.923128 1.496962 1.525373

O 6.296470 2.988634 -0.611443

O 7.194119 0.777519 -1.897991

H -9.350263 2.347577 -1.271776

H -8.732803 -0.423825 -2.659103

H -10.174825 0.589816 -2.571904

H -9.312211 -1.427412 -0.418471

H -10.743569 -0.418897 -0.332676

H -9.001777 0.433414 1.096630

H -7.196642 0.851856 -1.350617

H -10.330466 -1.160724 -2.661581

H -10.549061 2.073965 -0.003046

H -8.979234 2.787573 0.394141

H -6.713550 0.004526 1.547437

H -4.845238 0.463963 -0.889022

H -4.294150 -0.370774 2.010502

H -2.489122 0.083944 -0.475689

H -1.808182 -0.738299 2.402927

H 5.645640 -1.298570 -1.822283

H 3.451536 -3.672350 -0.889827

H 3.133380 -1.533103 -2.919341

H 1.779432 -2.479386 -2.251553

H 1.785464 -0.717907 -2.089347

H 2.616691 -0.569062 3.432858

H 2.397316 -2.278893 2.987022

H 3.816896 -1.382546 2.406953

H 5.853334 3.682783 -0.103264

H 7.578010 1.661226 -1.794870

H 3.058026 2.705977 1.161684

2a-c18 , delta G = 1.0881 kcal/mol, population = 7.13 %

C 3.352605 -1.581133 -1.002360

C 2.283874 -2.001182 0.020370

C 1.965605 -1.106367 1.221743

C 2.391561 0.352317 1.036521

C 3.581878 0.607242 0.259013

C 4.256864 1.836474 0.447566

C 5.487814 2.055808 -0.154408

C 6.051926 1.066018 -0.954546

C 5.374495 -0.133820 -1.186329

C 4.152016 -0.365535 -0.590634

C 2.626787 -1.287169 -2.329381

C 2.732774 -1.665976 2.429853

O 3.777543 2.830103 1.222161

C -0.276527 -0.753301 0.597245

C -1.658577 -0.837797 1.040607

C -2.672183 -0.474779 0.228221

C -4.058156 -0.522505 0.582173

C -5.044391 -0.157268 -0.268290

C -6.448143 -0.190082 0.048144

C -7.402882 0.173127 -0.822783

C -8.883747 0.195833 -0.589552

C -9.412960 1.626219 -0.844498

C -10.936098 1.731486 -0.886762

C -9.305979 -0.348144 0.774009

O 0.582945 -1.191314 1.560223

O 0.129217 -0.356618 -0.479969

O 1.749552 -3.078315 -0.096535

O 4.256042 -2.675119 -1.162732

O 1.807788 1.230645 1.677497

O 6.189937 3.217348 0.012414

O 7.254723 1.268027 -1.538978

H -10.391639 -0.389838 0.854975

H -11.353985 1.050342 -1.632738

H -11.244678 2.744918 -1.150501

H -9.012615 2.287503 -0.069477

H -9.004456 1.982427 -1.794636

H -9.330244 -0.442838 -1.365090

H -7.089906 0.509617 -1.809443

H -11.389109 1.491561 0.076236

H -8.924910 -1.358162 0.933656

H -8.934414 0.286947 1.582230

H -6.716678 -0.529471 1.043053

H -4.772729 0.186510 -1.263560

H -4.314054 -0.869546 1.579202

H -2.423818 -0.120041 -0.768184

H -1.834411 -1.202779 2.043941

H 5.816801 -0.865970 -1.845385

H 3.720574 -3.454059 -1.367749

H 3.375332 -1.060036 -3.087545

H 2.051362 -2.162599 -2.633519

H 1.945336 -0.445723 -2.222067

H 3.805744 -1.656290 2.242231

H 2.515613 -1.053490 3.303801

H 2.408224 -2.689583 2.609000

H 5.686343 3.811444 0.586761

H 7.584499 2.144273 -1.289289

H 2.894489 2.530042 1.555887

2a-c10 , delta G = 1.6190 kcal/mol, population = 2.91 %

C 3.489234 -1.877911 0.012018

C 2.006690 -1.765811 0.403018

C 1.384445 -0.399637 0.704101

C 2.163476 0.778887 0.115609

C 3.600422 0.656220 0.034370

C 4.371421 1.839412 -0.053395

C 5.755331 1.779029 0.029929

C 6.384558 0.547767 0.191092

C 5.637797 -0.632546 0.233511

C 4.261447 -0.585627 0.154724

C 3.542174 -2.341334 -1.456696

C 1.366243 -0.230272 2.231109

O 3.827737 3.065240 -0.189973

C -0.179954 -0.537420 -1.047818

C -1.576495 -0.499881 -1.445187

C -2.605924 -0.307566 -0.594205

C -3.982638 -0.291363 -0.988489

C -4.995458 -0.101186 -0.112926

C -6.389844 -0.096057 -0.469045

C -7.381688 0.089894 0.415050

C -8.841917 0.091244 0.089836

C -9.450369 1.476816 0.393233

C -10.914888 1.616376 -0.018845

C -9.551096 -1.033707 0.860721

O 0.021385 -0.368075 0.288718

O 0.754584 -0.712799 -1.809620

O 1.356867 -2.774983 0.540594

O 4.091654 -2.853198 0.863446

O 1.565350 1.833627 -0.115036

O 6.542845 2.895268 -0.029140

O 7.732109 0.479092 0.282354

H -9.490202 -0.859801 1.938465

H -11.263094 2.639081 0.137993

H -11.565411 0.956006 0.556525

H -9.345038 1.681883 1.463937

H -8.855868 2.232959 -0.126532

H -8.954667 -0.098582 -0.983021

H -7.126301 0.251336 1.461416

H -11.047433 1.377311 -1.077230

H -10.604931 -1.096179 0.587936

H -9.090956 -1.999709 0.647332

H -6.633907 -0.259252 -1.515840

H -4.755845 0.054609 0.936223

H -4.208194 -0.446862 -2.039647

H -2.390921 -0.160147 0.459064

H -1.743248 -0.648420 -2.504471

H 6.155235 -1.576740 0.316671

H 3.553003 -3.653941 0.799486

H 2.983293 -3.271492 -1.567805

H 3.104926 -1.594311 -2.116113

H 4.584325 -2.505726 -1.728104

H 2.377658 -0.269826 2.633636

H 0.919117 0.731669 2.477683

H 0.774588 -1.033639 2.666726

H 5.978978 3.674166 -0.138110

H 8.099354 1.373957 0.225546

H 2.847479 2.936319 -0.252781

2a-c13 , delta G = 1.7984 kcal/mol, population = 2.15 %

C 3.321730 -1.624825 -0.929153

C 2.242274 -2.026028 0.089857

C 1.887302 -1.093690 1.251466

C 2.291360 0.364708 1.020334

C 3.489741 0.612752 0.253320

C 4.139315 1.860396 0.407031

C 5.376306 2.080206 -0.182228

C 5.971885 1.072042 -0.935196

C 5.320057 -0.147911 -1.133297

C 4.091797 -0.380065 -0.549812

C 2.614857 -1.394984 -2.278984

C 2.642779 -1.595628 2.491974

O 3.629246 2.872700 1.136470

C -0.350072 -0.799068 0.579529

C -1.736804 -0.880476 1.008518

C -2.743508 -0.554225 0.172263

C -4.132895 -0.596106 0.513239

C -5.111566 -0.267644 -0.360696

C -6.518344 -0.290614 -0.057944

C -7.464099 0.032877 -0.953522

C -8.947746 0.050425 -0.741634

C -9.527380 1.427228 -1.148345

C -9.066843 2.592698 -0.274743

C -9.391221 -0.381684 0.655541

O 0.500720 -1.189910 1.570142

O 0.066002 -0.439115 -0.506626

O 1.729326 -3.116411 0.004075

O 4.247327 -2.707108 -1.032150

O 1.681484 1.255095 1.619124

O 6.054529 3.259830 -0.046672

O 7.180857 1.274345 -1.506585

H -10.479299 -0.343321 0.731479

H -7.978092 2.678867 -0.271596

H -9.393351 2.474992 0.759954

H -9.252431 1.623232 -2.188963

H -10.618432 1.356684 -1.125116

H -9.365663 -0.668609 -1.459455

H -7.140948 0.327522 -1.950449

H -9.476241 3.535073 -0.643488

H -9.075856 -1.403766 0.872063

H -8.979748 0.265978 1.431477

H -6.797917 -0.586396 0.947885

H -4.830566 0.038068 -1.365761

H -4.397724 -0.906042 1.520128

H -2.486376 -0.234449 -0.833714

H -1.921848 -1.209355 2.022636

H 5.786648 -0.895448 -1.757406

H 3.729175 -3.503228 -1.214350

H 2.063312 -2.293056 -2.560628

H 1.914181 -0.564944 -2.215378

H 3.372423 -1.180094 -3.031746

H 2.332515 -2.617625 2.702816

H 3.718559 -1.573938 2.322331

H 2.399957 -0.955851 3.339165

H 5.531035 3.864533 0.498057

H 7.490753 2.165110 -1.284335

H 2.746453 2.568343 1.467074

# Table S72. Geometry data of conformers of structure 2a'

2a'-c1 , delta G = 0.0000 kcal/mol, population = 38.37 %

C -3.303397 -1.719841 -0.774116

C -2.238432 -2.006941 0.297130

C -1.910777 -0.959935 1.365040

C -2.317879 0.464565 0.980321

C -3.504252 0.624167 0.172270

C -4.163434 1.876071 0.187764

C -5.392483 2.025951 -0.439031

C -5.971033 0.941707 -1.092924

C -5.308612 -0.287074 -1.156321

C -4.088167 -0.449709 -0.534421

C -2.574954 -1.618850 -2.128551

C -2.687190 -1.339383 2.635899

O -3.670666 2.961155 0.817666

C 0.336575 -0.716830 0.707280

C 1.716802 -0.760710 1.162729

C 2.734364 -0.479876 0.323356

C 4.118750 -0.494704 0.686870

C 5.108161 -0.197069 -0.185808

C 6.510130 -0.197693 0.138134

C 7.473818 0.109823 -0.742838

C 8.942027 0.120351 -0.458492

C 9.533966 1.529842 -0.657819

C 8.964760 2.578107 0.294459

C 9.663797 -0.899829 -1.352486

O -0.529792 -1.015898 1.716136

O -0.061742 -0.456861 -0.413396

O -1.714066 -3.094776 0.328120

O -4.219380 -2.814826 -0.783526

O -1.723990 1.415602 1.496257

O -6.079037 3.208471 -0.433912

O -7.172819 1.074624 -1.698726

H 10.734053 -0.907377 -1.135377

H 9.130114 2.290095 1.335918

H 7.889315 2.702242 0.153784

H 10.617866 1.462900 -0.526616

H 9.367134 1.837725 -1.695592

H 9.092752 -0.166120 0.587847

H 7.186373 0.377258 -1.759028

H 9.438512 3.548987 0.137113

H 9.274629 -1.907018 -1.195540

H 9.536004 -0.648195 -2.408506

H 6.784184 -0.461681 1.156380

H 4.840929 0.064521 -1.206858

H 4.370327 -0.756808 1.710563

H 2.490932 -0.221344 -0.703381

H 1.887621 -1.022437 2.198665

H -5.761144 -1.097877 -1.707673

H -3.693616 -3.620722 -0.881162

H -2.006710 -2.532608 -2.307845

H -1.886474 -0.776276 -2.138602

H -3.321502 -1.492587 -2.911769

H -2.376403 -2.332752 2.955058

H -3.759617 -1.341954 2.444932

H -2.463604 -0.615905 3.418593

H -5.569400 3.867259 0.058709

H -7.493261 1.980203 -1.571824

H -2.792303 2.697684 1.192580

2a'-c4 , delta G = 0.2654 kcal/mol, population = 24.50 %

C 3.360478 1.923114 0.010807

C 1.880436 1.765882 0.396359

C 1.301036 0.381962 0.700774

C 2.117916 -0.773449 0.117490

C 3.550224 -0.606560 0.038810

C 4.357685 -1.765521 -0.045145

C 5.738802 -1.662301 0.042215

C 6.328977 -0.411896 0.203894

C 5.545990 0.744955 0.241839

C 4.172058 0.655497 0.158583

C 3.405445 2.386204 -1.458150

C 1.285394 0.217328 2.228286

O 3.852484 -3.007694 -0.181548

C -0.261299 0.452134 -1.056579

C -1.651174 0.334812 -1.461751

C -2.672428 0.081907 -0.616855

C -4.041153 -0.034607 -1.022109

C -5.044985 -0.290056 -0.152888

C -6.430474 -0.414698 -0.521321

C -7.411965 -0.666968 0.357471

C -8.862648 -0.812977 0.023962

C -9.707106 0.256310 0.744797

C -9.382223 1.686714 0.322697

C -9.351066 -2.223569 0.388230

O -0.059171 0.303798 0.282321

O 0.667547 0.668197 -1.814899

O 1.198835 2.754553 0.528600

O 3.929094 2.917530 0.863472

O 1.553123 -1.846779 -0.111828

O 6.560617 -2.753753 -0.012973

O 7.673360 -0.301281 0.299908

H -10.407438 -2.338442 0.136068

H -8.345573 1.943854 0.548748

H -10.025730 2.402887 0.837299

H -10.761844 0.044725 0.546260

H -9.568287 0.144900 1.825516

H -8.984068 -0.670669 -1.055124

H -7.154018 -0.782016 1.409583

H -9.527859 1.818830 -0.752587

H -8.784968 -2.987461 -0.147055

H -9.238311 -2.407675 1.459804

H -6.676285 -0.294499 -1.573330

H -4.804050 -0.411886 0.900447

H -4.267410 0.089254 -2.077311

H -2.456388 -0.043874 0.439030

H -1.819229 0.465270 -2.523177

H 6.034084 1.704663 0.324997

H 3.367162 3.701742 0.795131

H 2.818516 3.298485 -1.572806

H 2.994408 1.625205 -2.118468

H 4.443198 2.582455 -1.725160

H 0.667071 1.002461 2.660098

H 2.294342 0.291422 2.632155

H 0.869544 -0.757849 2.477558

H 6.021242 -3.549966 -0.120840

H 8.068469 -1.184418 0.246643

H 2.868784 -2.909274 -0.246421

2a'-c6 , delta G = 0.6896 kcal/mol, population = 11.96 %

C -3.360771 -1.682208 -0.833924

C -2.298665 -1.992141 0.233898

C -1.988498 -0.976398 1.336617

C -2.409198 0.455085 0.994965

C -3.593733 0.627197 0.186716

C -4.267464 1.870319 0.239434

C -5.495892 2.025713 -0.387245

C -6.059149 0.956326 -1.077912

C -5.382011 -0.261927 -1.178177

C -4.161994 -0.430071 -0.556814

C -2.625787 -1.528151 -2.179762

C -2.768314 -1.405023 2.589739

O -3.789817 2.940361 0.905621

C 0.260859 -0.689162 0.702469

C 1.637934 -0.731893 1.167545

C 2.659717 -0.420086 0.344409

C 4.040996 -0.433495 0.719646

C 5.036528 -0.109873 -0.136652

C 6.435538 -0.113602 0.200378

C 7.408153 0.209915 -0.665364

C 8.872321 0.218119 -0.359269

C 9.432617 1.649076 -0.504867

C 10.897249 1.788740 -0.093460

C 9.604521 -0.784094 -1.265719

O -0.609405 -1.028525 1.695130

O -0.131986 -0.399832 -0.412879

O -1.762896 -3.074862 0.233264

O -4.263269 -2.787176 -0.885222

O -1.828238 1.395697 1.543655

O -6.196407 3.199404 -0.347428

O -7.260174 1.094682 -1.684031

H 10.664120 -0.841702 -1.014942

H 11.561128 1.220946 -0.747103

H 11.051374 1.434644 0.929253

H 9.306144 1.972988 -1.543466

H 8.821514 2.319841 0.105064

H 9.007187 -0.092837 0.682241

H 7.132215 0.492898 -1.680274

H 11.210565 2.833712 -0.134739

H 9.179445 -1.783536 -1.161520

H 9.520748 -0.487684 -2.314804

H 6.699926 -0.398502 1.215728

H 4.777555 0.173927 -1.153895

H 4.285155 -0.718308 1.739082

H 2.422708 -0.136966 -0.677331

H 1.802986 -1.019976 2.197410

H -5.822798 -1.059719 -1.757308

H -3.726710 -3.583203 -1.003211

H -3.369174 -1.382931 -2.962714

H -2.047504 -2.429786 -2.386329

H -1.945647 -0.679057 -2.157463

H -2.448578 -2.404872 2.878373

H -3.839539 -1.412747 2.392383

H -2.557114 -0.704953 3.396806

H -5.696480 3.848239 0.167839

H -7.591521 1.992029 -1.529609

H -2.909913 2.675365 1.275750

2a'-c9 , delta G = 0.9965 kcal/mol, population = 7.12 %

C -3.451238 -1.916676 0.025490

C -1.969335 -1.772148 0.408616

C -1.375122 -0.392176 0.702497

C -2.180924 0.767482 0.112446

C -3.615166 0.614856 0.037898

C -4.410868 1.781442 -0.051689

C -5.792815 1.692881 0.038522

C -6.395514 0.449545 0.208353

C -5.624304 -0.714946 0.252217

C -4.249642 -0.639985 0.166487

C -3.502432 -2.388039 -1.440660

C -1.355341 -0.217101 2.228815

O -3.893199 3.017558 -0.196413

C 0.184859 -0.497403 -1.055445

C 1.576620 -0.410049 -1.461372

C 2.601884 -0.162396 -0.619715

C 3.973181 -0.082679 -1.024978

C 4.981494 0.169168 -0.159845

C 6.370016 0.251982 -0.528942

C 7.356697 0.506676 0.343721

C 8.810909 0.597043 0.004220

C 9.592125 -0.511791 0.740671

C 11.071147 -0.585880 0.364777

C 9.343254 1.999665 0.337790

O -0.014661 -0.331732 0.281796

O -0.747528 -0.706125 -1.811532

O -1.297946 -2.766940 0.547053

O -4.028948 -2.900185 0.884626

O -1.605632 1.833233 -0.125657

O -6.603534 2.792288 -0.021859

O -7.740846 0.353330 0.306740

H 10.387062 2.106972 0.041149

H 11.549536 -1.438531 0.850540

H 11.614234 0.310987 0.666259

H 9.490687 -0.357264 1.820254

H 9.115720 -1.470686 0.519634

H 8.922959 0.430451 -1.072540

H 7.101254 0.661309 1.391080

H 11.194564 -0.705022 -0.714793

H 8.765796 2.766656 -0.180587

H 9.275361 2.192479 1.411870

H 6.613637 0.097200 -1.577068

H 4.742303 0.320951 0.889966

H 4.198138 -0.234980 -2.076734

H 2.387414 -0.013481 0.433450

H 1.743100 -0.561299 -2.520288

H -6.121935 -1.669130 0.341897

H -3.474964 -3.690381 0.820547

H -4.542356 -2.575657 -1.705378

H -2.924626 -3.306681 -1.550564

H -3.084735 -1.635105 -2.105982

H -0.744859 -1.005874 2.665094

H -2.364322 -0.277328 2.634853

H -0.928480 0.755161 2.470721

H -6.056359 3.582275 -0.135983

H -8.126916 1.240144 0.248580

H -2.910781 2.908601 -0.263263

2a'-c11 , delta G = 1.2789 kcal/mol, population = 4.42 %

C 3.173731 1.836182 -0.589667

C 2.093640 1.924268 0.501088

C 1.858324 0.750466 1.455321

C 2.379942 -0.589265 0.929370

C 3.570184 -0.568584 0.111594

C 4.329402 -1.757503 0.000182

C 5.562071 -1.741248 -0.637209

C 6.045631 -0.552113 -1.175923

C 5.285449 0.618669 -1.112964

C 4.060106 0.616097 -0.479548

C 2.449672 1.817127 -1.949914

C 2.606698 1.061182 2.761035

O 3.931303 -2.937666 0.515497

C -0.363698 0.387460 0.767363

C -1.739837 0.266974 1.220760

C -2.733431 -0.024131 0.356407

C -4.111431 -0.166806 0.715932

C -5.074231 -0.475143 -0.182592

C -6.469675 -0.628089 0.134335

C -7.397285 -0.943504 -0.782913

C -8.869237 -1.119332 -0.562575

C -9.666345 -0.223588 -1.541876

C -9.482926 1.277165 -1.323374

C -9.315183 -0.940272 0.888230

O 0.479223 0.655738 1.804576

O 0.049804 0.277357 -0.372567

O 1.481545 2.956304 0.640773

O 3.998683 2.996458 -0.481875

O 1.870113 -1.633137 1.346089

O 6.343316 -2.857509 -0.752885

O 7.249597 -0.524247 -1.791235

H -10.393752 -1.085985 0.969601

H -10.038581 1.845241 -2.071826

H -8.431465 1.562826 -1.398906

H -9.370113 -0.480053 -2.563336

H -10.724971 -0.483048 -1.452921

H -9.099187 -2.152877 -0.855446

H -7.068581 -1.084490 -1.811059

H -9.841782 1.589114 -0.341051

H -8.828626 -1.664373 1.543941

H -9.084321 0.057879 1.263666

H -6.756574 -0.476230 1.169756

H -4.787783 -0.620571 -1.221487

H -4.380168 -0.020683 1.758231

H -2.473063 -0.163903 -0.689046

H -1.927165 0.413496 2.276328

H 5.666692 1.516234 -1.576705

H 3.410230 3.763711 -0.502119

H 1.831857 0.926897 -2.049780

H 3.200350 1.833556 -2.739197

H 1.808292 2.695391 -2.035895

H 2.215618 1.987308 3.178854

H 3.674164 1.173125 2.574860

H 2.448348 0.245088 3.464539

H 5.892839 -3.603764 -0.332667

H 7.642051 -1.409611 -1.759112

H 3.037581 -2.786733 0.915408

2a'-c23 , delta G = 1.3993 kcal/mol, population = 3.61 %

C -3.374389 -1.920264 -0.025235

C -1.884462 -1.770741 0.323142

C -1.298429 -0.395657 0.653615

C -2.132651 0.775660 0.129684

C -3.566108 0.607633 0.079221

C -4.377316 1.766742 0.046657

C -5.756054 1.657927 0.160963

C -6.340458 0.402106 0.299992

C -5.554868 -0.753444 0.287679

C -4.183254 -0.658665 0.176317

C -3.458771 -2.346663 -1.503639

C -1.236675 -0.277586 2.184149

O -3.877470 3.013496 -0.065162

C 0.211436 -0.403060 -1.150957

C 1.589847 -0.275090 -1.591114

C 2.638123 -0.087001 -0.762595

C 3.997032 0.037784 -1.197329

C 5.027069 0.215807 -0.339491

C 6.405288 0.344265 -0.733339

C 7.412440 0.508837 0.137967

C 8.856840 0.641944 -0.231479

C 9.680181 -0.585236 0.228314

C 9.722054 -0.826101 1.737238

C 9.433548 1.958543 0.312048

O 0.048971 -0.302674 0.197758

O -0.740011 -0.587747 -1.889255

O -1.197439 -2.761159 0.404457

O -3.918430 -2.936792 0.817580

O -1.575893 1.857769 -0.077172

O -6.580720 2.748585 0.154739

O -7.682188 0.285588 0.422483

H 9.328563 2.024070 1.396590

H 8.726523 -1.011227 2.145385

H 10.150913 0.023023 2.272332

H 9.277240 -1.472160 -0.268071

H 10.700630 -0.456206 -0.145289

H 8.920964 0.670466 -1.323479

H 7.176226 0.553221 1.198863

H 10.335519 -1.699897 1.964636

H 10.495734 2.039178 0.070311

H 8.917578 2.815614 -0.123589

H 6.622553 0.302878 -1.797879

H 4.814978 0.265887 0.725879

H 4.193314 -0.015778 -2.264368

H 2.453357 -0.026156 0.304837

H 1.725339 -0.345518 -2.662820

H -6.039428 -1.716232 0.354272

H -3.361742 -3.720177 0.709136

H -4.503020 -2.538620 -1.747433

H -2.873767 -3.254503 -1.657237

H -3.067334 -1.568254 -2.155633

H -0.604421 -1.074104 2.572781

H -2.232405 -0.365918 2.616952

H -0.814366 0.690445 2.449874

H -6.045062 3.548902 0.059465

H -8.079969 1.168913 0.403329

H -2.895023 2.919199 -0.153118

2a'-c15 , delta G = 1.4288 kcal/mol, population = 3.43 %

C -3.231662 -1.689071 -0.879276

C -2.104720 -1.976434 0.126986

C -1.807186 -0.990024 1.259222

C -2.322378 0.427141 0.995414

C -3.548902 0.561930 0.244883

C -4.288893 1.760574 0.377786

C -5.550524 1.868523 -0.189980

C -6.081636 0.796721 -0.902096

C -5.341537 -0.374795 -1.081810

C -4.088275 -0.496374 -0.518167

C -2.570600 -1.444806 -2.249683

C -2.509759 -1.513939 2.521756

O -3.843728 2.829832 1.067423

C 0.394864 -0.552471 0.548991

C 1.789590 -0.533669 0.959223

C 2.763085 -0.164394 0.101942

C 4.156705 -0.116474 0.424631

C 5.103610 0.252618 -0.467893

C 6.512669 0.315919 -0.183220

C 7.427419 0.680169 -1.095191

C 8.907126 0.833425 -0.892449

C 9.461062 0.194273 0.386074

C 9.423537 -1.333858 0.390685

C 9.266758 2.330282 -0.965949

O -0.414184 -0.974881 1.561679

O -0.058711 -0.247809 -0.538925

O -1.506677 -3.023662 0.056334

O -4.072362 -2.841956 -0.932181

O -1.771272 1.377385 1.557776

O -6.314193 2.996622 -0.071749

O -7.313850 0.889165 -1.451755

H 8.884202 2.787682 -1.880310

H 10.004835 -1.735629 -0.443043

H 8.406926 -1.718005 0.298141

H 10.497543 0.522989 0.500465

H 8.926093 0.590908 1.254532

H 9.395123 0.346350 -1.746863

H 7.072920 0.940485 -2.090583

H 9.848407 -1.732071 1.314236

H 8.839127 2.866814 -0.116022

H 10.350388 2.462365 -0.946814

H 6.818710 0.050547 0.823280

H 4.793295 0.523071 -1.474368

H 4.451511 -0.391321 1.433388

H 2.474105 0.117740 -0.906592

H 2.008778 -0.828466 1.977023

H -5.761072 -1.173059 -1.675858

H -3.498452 -3.600582 -1.107030

H -1.955311 -2.304933 -2.517334

H -1.936683 -0.560661 -2.224281

H -3.356595 -1.311962 -2.992192

H -2.120941 -2.503642 2.755182

H -3.585768 -1.577706 2.364657

H -2.307217 -0.835498 3.349230

H -5.830443 3.652619 0.449854

H -7.687617 1.759248 -1.246519

H -2.934167 2.603566 1.387731

2a'-c20 , delta G = 1.5468 kcal/mol, population = 2.81 %

C -3.341658 -1.670828 -0.877226

C -2.227183 -1.997732 0.130755

C -1.896922 -1.023057 1.264296

C -2.365839 0.410602 1.003262

C -3.586010 0.587319 0.251015

C -4.285921 1.809825 0.384554

C -5.541735 1.960809 -0.186263

C -6.106939 0.908259 -0.900888

C -5.406221 -0.287160 -1.080714

C -4.158771 -0.451199 -0.514932

C -2.669752 -1.445448 -2.245646

C -2.614723 -1.525846 2.526775

O -3.807145 2.862196 1.077612

C 0.317264 -0.652630 0.553849

C 1.711936 -0.671320 0.964508

C 2.694011 -0.326246 0.106983

C 4.088786 -0.308896 0.428212

C 5.040842 0.039625 -0.466933

C 6.452291 0.078802 -0.186991

C 7.367293 0.426011 -1.105934

C 8.852634 0.528752 -0.922706

C 9.345194 0.075034 0.455885

C 10.867194 0.022984 0.581996

C 9.301335 1.961947 -1.266273

O -0.503719 -1.053313 1.565610

O -0.127318 -0.334225 -0.533883

O -1.665566 -3.064993 0.060494

O -4.220063 -2.794944 -0.935092

O -1.785172 1.341143 1.569018

O -6.267756 3.113570 -0.068226

O -7.334140 1.042849 -1.453062

H 8.920044 2.267653 -2.242291

H 11.318321 1.012998 0.499511

H 11.303238 -0.608675 -0.196455

H 8.936987 0.743479 1.220881

H 8.939706 -0.918830 0.663768

H 9.304724 -0.134708 -1.673680

H 7.011669 0.687431 -2.100829

H 11.159985 -0.391160 1.548561

H 8.925537 2.666919 -0.520776

H 10.388213 2.039437 -1.294760

H 6.758984 -0.186996 0.819035

H 4.732644 0.314277 -1.472971

H 4.379355 -0.587955 1.437010

H 2.411216 -0.037933 -0.901577

H 1.923172 -0.970830 1.982600

H -5.851068 -1.070104 -1.676715

H -3.670883 -3.572103 -1.108049

H -2.084489 -2.325767 -2.514968

H -2.005085 -0.584380 -2.216161

H -3.449151 -1.282830 -2.989182

H -2.258672 -2.528350 2.757859

H -3.692487 -1.553649 2.371189

H -2.388691 -0.855972 3.355133

H -5.763142 3.752979 0.454188

H -7.678974 1.924583 -1.246917

H -2.906744 2.604570 1.400330

2a'-c10 , delta G = 1.6309 kcal/mol, population = 2.44 %

C -3.266586 -1.565935 -1.059571

C -2.239026 -2.016462 -0.007619

C -1.969816 -1.157708 1.231304

C -2.384228 0.307001 1.070887

C -3.539561 0.589076 0.251357

C -4.217706 1.815690 0.445987

C -5.420471 2.058414 -0.202166

C -5.952798 1.094895 -1.054466

C -5.270690 -0.101345 -1.290658

C -4.076291 -0.356577 -0.649295

C -2.490746 -1.244572 -2.351370

C -2.787712 -1.749689 2.389492

O -3.768914 2.784420 1.268901

C 0.295109 -0.793529 0.707360

C 1.659373 -0.895034 1.200182

C 2.701573 -0.507149 0.437219

C 4.074471 -0.566802 0.837575

C 5.088079 -0.172898 0.033514

C 6.480890 -0.213401 0.392767

C 7.467537 0.183134 -0.425834

C 8.925961 0.162241 -0.089457

C 9.499812 1.592366 0.053790

C 9.445054 2.456612 -1.205879

C 9.705867 -0.670437 -1.118682

O -0.601917 -1.256345 1.622940

O -0.067543 -0.364234 -0.372783

O -1.700205 -3.090193 -0.134064

O -4.168746 -2.650893 -1.279309

O -1.826122 1.163808 1.761973

O -6.125012 3.217981 -0.032277

O -7.128132 1.319664 -1.684664

H 10.773273 -0.661839 -0.886946

H 8.419374 2.609952 -1.547110

H 10.008871 2.012625 -2.028120

H 8.961848 2.096393 0.861216

H 10.539278 1.499403 0.383121

H 9.036107 -0.318698 0.887241

H 7.202203 0.547907 -1.415696

H 9.874169 3.440757 -1.008827

H 9.363920 -1.706572 -1.117368

H 9.577301 -0.279055 -2.129533

H 6.727708 -0.587556 1.383352

H 4.849007 0.203560 -0.958246

H 4.296338 -0.946257 1.830835

H 2.487747 -0.120351 -0.555180

H 1.797843 -1.292113 2.197149

H -5.687410 -0.812465 -1.988295

H -3.630848 -3.425386 -1.494298

H -1.814428 -0.405655 -2.200645

H -3.209908 -1.000893 -3.132462

H -1.903849 -2.113611 -2.651726

H -3.851459 -1.735317 2.155037

H -2.610057 -1.161192 3.288449

H -2.470029 -2.777770 2.554765

H -5.644723 3.792971 0.580194

H -7.465667 2.189578 -1.423485

H -2.902441 2.470568 1.632218

2a'-c32 , delta G = 1.9879 kcal/mol, population = 1.33 %

C -3.515708 -1.862548 0.141946

C -2.036944 -1.755565 0.548471

C -1.387074 -0.385911 0.760381

C -2.130859 0.765228 0.079133

C -3.568997 0.668865 -0.015505

C -4.311180 1.859513 -0.198530

C -5.697125 1.835392 -0.132557

C -6.356672 0.632728 0.105120

C -5.637989 -0.557743 0.242296

C -4.259980 -0.546617 0.181475

C -3.557630 -2.428503 -1.290621

C -1.384177 -0.107376 2.271550

O -3.737750 3.060391 -0.413346

C 0.197454 -0.669724 -0.956444

C 1.599168 -0.672393 -1.336645

C 2.619830 -0.439414 -0.485095

C 4.001259 -0.451699 -0.862217

C 5.003948 -0.220614 0.015273

C 6.402702 -0.231835 -0.324680

C 7.375663 -0.004400 0.571660

C 8.853548 0.023048 0.320277

C 9.412538 1.401071 0.744235

C 10.937878 1.476621 0.769687

C 9.247574 -0.352712 -1.107061

O -0.018369 -0.413657 0.363986

O -0.729820 -0.879913 -1.718351

O -1.412927 -2.766527 0.767873

O -4.153141 -2.761738 1.049706

O -1.505376 1.787671 -0.215475

O -6.458086 2.961511 -0.282472

O -7.706610 0.600504 0.179817

H 10.330989 -0.405750 -1.209068

H 11.357305 0.697145 1.411234

H 11.268818 2.441844 1.157833

H 9.011098 2.161472 0.066856

H 9.026340 1.639478 1.739331

H 9.298470 -0.715096 1.002775

H 7.081898 0.202784 1.598986

H 11.369002 1.358809 -0.225404

H 8.842817 -1.326828 -1.386370

H 8.880005 0.386300 -1.823581

H 6.651042 -0.439782 -1.360149

H 4.751342 -0.013093 1.052428

H 4.239004 -0.660804 -1.901347

H 2.393065 -0.230686 0.555300

H 1.777465 -0.883919 -2.383278

H -6.177902 -1.482213 0.383392

H -3.633124 -3.577224 1.049379

H -3.022604 -3.378531 -1.323877

H -3.089380 -1.743339 -1.994315

H -4.599308 -2.585005 -1.568318

H -0.820034 -0.891804 2.773194

H -2.401363 -0.091109 2.660959

H -0.914529 0.858085 2.454367

H -5.875114 3.717670 -0.440034

H -8.052031 1.497195 0.055124

H -2.759982 2.905999 -0.452212

# Table S73. Geometry data of conformers of structure 2b

2b-c3 , delta G = 0.0000 kcal/mol, population = 31.79 %

C 3.329212 2.048538 0.179937

C 1.818839 1.796733 0.208137

C 1.283226 0.607466 1.003512

C 2.213885 -0.618967 0.926051

C 3.555740 -0.478594 0.413005

C 4.342394 -1.649300 0.272503

C 5.592468 -1.585874 -0.326307

C 6.068660 -0.370515 -0.808124

C 5.312078 0.795655 -0.665352

C 4.074595 0.747637 -0.061585

C 3.796946 2.668612 1.515649

C 1.063882 0.983107 2.473768

O 3.933356 -2.868022 0.674952

C -0.094248 -0.200901 -0.754341

C -1.449715 -0.496282 -1.189477

C -2.550631 -0.305278 -0.433400

C -3.886478 -0.589660 -0.865436

C -4.968827 -0.379921 -0.082556

C -6.326860 -0.649351 -0.475381

C -7.385230 -0.426401 0.317941

C -8.813124 -0.691663 -0.039909

C -9.637879 0.610922 -0.028092

C -9.184321 1.637114 -1.062900

C -9.419482 -1.723506 0.923548

O -0.020285 0.261315 0.526783

O 0.902220 -0.345152 -1.436933

O 1.076678 2.548743 -0.379515

O 3.604528 2.982159 -0.849837

O 1.759235 -1.694965 1.329784

O 6.381353 -2.690743 -0.491179

O 7.276628 -0.303685 -1.411211

H -10.458325 -1.930023 0.657400

H -9.233536 1.220778 -2.072438

H -8.154151 1.952214 -0.886450

H -10.685460 0.349506 -0.203723

H -9.592978 1.046987 0.975629

H -8.843526 -1.102703 -1.054650

H -7.214636 -0.017634 1.313226

H -9.817127 2.526341 -1.037022

H -8.864794 -2.662730 0.896289

H -9.402629 -1.350352 1.950877

H -6.484542 -1.052631 -1.472411

H -4.818454 0.021177 0.916960

H -4.020112 -0.988690 -1.866874

H -2.430378 0.090508 0.569655

H -1.519820 -0.884821 -2.197526

H 5.706980 1.727073 -1.042641

H 2.744010 3.301991 -1.166674

H 4.818439 3.015269 1.364468

H 3.170364 3.520894 1.781176

H 3.799724 1.947808 2.330179

H 2.001127 1.189976 2.981855

H 0.579058 0.139669 2.961876

H 0.419657 1.859066 2.534435

H 5.902296 -3.469199 -0.173054

H 7.664626 -1.191156 -1.440968

H 3.013713 -2.753931 1.030518

2b-c2 , delta G = 0.3483 kcal/mol, population = 17.65 %

C 3.775652 -1.517702 1.015844

C 2.479789 -2.069912 0.414790

C 2.119214 -1.705695 -1.024982

C 2.525522 -0.263072 -1.386364

C 3.407041 0.481120 -0.519117

C 3.670023 1.836717 -0.838617

C 4.407651 2.630670 0.027974

C 4.876214 2.098802 1.225247

C 4.636972 0.760702 1.549632

C 3.916461 -0.040814 0.691674

C 4.996513 -2.283208 0.459057

C 2.765461 -2.679522 -2.017123

O 3.214380 2.434741 -1.956354

C -0.104420 -1.050773 -0.505353

C -1.505435 -1.346327 -0.765256

C -2.487024 -0.643623 -0.164415

C -3.887058 -0.871884 -0.358157

C -4.841901 -0.143580 0.263618

C -6.257502 -0.342391 0.097636

C -7.187835 0.394506 0.722745

C -8.667750 0.222956 0.591511

C -9.327366 1.502880 0.042260

C -8.868298 1.878053 -1.364341

C -9.279274 -0.159917 1.948600

O 0.711474 -1.877442 -1.222292

O 0.343767 -0.195555 0.233299

O 1.775879 -2.796057 1.077189

O 3.738415 -1.722856 2.417249

O 2.070466 0.201412 -2.437444

O 4.676418 3.945503 -0.235402

O 5.579233 2.870460 2.084181

H -10.359010 -0.295175 1.855723

H -9.384230 2.772048 -1.719769

H -9.074263 1.069755 -2.070854

H -10.411180 1.355057 0.048832

H -9.124275 2.327635 0.733757

H -8.859639 -0.589372 -0.117632

H -6.861834 1.189620 1.392249

H -7.794959 2.075275 -1.393384

H -8.848436 -1.087648 2.328349

H -9.100382 0.624018 2.689078

H -6.571498 -1.136386 -0.575231

H -4.533934 0.651275 0.938667

H -4.180076 -1.668875 -1.035637

H -2.199999 0.152471 0.516832

H -1.721679 -2.151839 -1.454544

H 5.017056 0.369880 2.481558

H 2.955548 -2.270030 2.593021

H 5.210310 -2.025283 -0.575656

H 5.855006 -1.997407 1.065559

H 4.844474 -3.359565 0.546270

H 2.393609 -2.435412 -3.010542

H 2.481332 -3.699451 -1.762270

H 3.848482 -2.597066 -2.017990

H 4.244172 4.193796 -1.064999

H 5.649211 3.765836 1.720250

H 2.670035 1.757298 -2.436213

2b-c6 , delta G = 0.4123 kcal/mol, population = 15.84 %

C 3.778340 -1.469797 1.126654

C 2.493788 -2.029125 0.508416

C 2.185699 -1.727768 -0.958025

C 2.632701 -0.313653 -1.376362

C 3.512878 0.450673 -0.525468

C 3.828865 1.777569 -0.911329

C 4.573278 2.593431 -0.071389

C 4.993994 2.114217 1.165264

C 4.701975 0.804689 1.555600

C 3.975303 -0.019570 0.724449

C 4.997168 -2.298970 0.662217

C 2.844258 -2.760281 -1.880508

O 3.420659 2.327542 -2.071302

C -0.038159 -1.011913 -0.534210

C -1.436639 -1.298343 -0.816132

C -2.423218 -0.569650 -0.255740

C -3.820894 -0.796028 -0.467728

C -4.783726 -0.053952 0.124750

C -6.196836 -0.260907 -0.053357

C -7.138243 0.474527 0.557214

C -8.615031 0.285257 0.411210

C -9.255951 1.560450 -0.174366

C -10.746287 1.424745 -0.482145

C -9.229518 -0.101096 1.766707

O 0.781801 -1.883060 -1.191560

O 0.405941 -0.132124 0.177654

O 1.759708 -2.717321 1.178564

O 3.681042 -1.587569 2.535677

O 2.214828 0.109131 -2.459964

O 4.893410 3.881928 -0.399604

O 5.700960 2.909470 1.999067

H -10.296794 -0.304117 1.672468

H -11.337545 1.270737 0.421786

H -10.932169 0.580704 -1.151476

H -9.095543 2.388520 0.524364

H -8.721382 1.822147 -1.091554

H -8.788362 -0.535173 -0.293539

H -6.825099 1.277189 1.223383

H -11.121892 2.325883 -0.970712

H -8.751205 -0.995660 2.168372

H -9.101489 0.706485 2.492636

H -6.499383 -1.066602 -0.717750

H -4.485104 0.754406 0.787833

H -4.105646 -1.607705 -1.131172

H -2.142808 0.243573 0.407797

H -1.647493 -2.123726 -1.483257

H 5.044954 0.454462 2.517588

H 2.898748 -2.135848 2.710328

H 4.813362 -3.363695 0.810452

H 5.252447 -2.111720 -0.378437

H 5.842032 -1.996995 1.279861

H 3.928140 -2.695206 -1.851087

H 2.507803 -2.559670 -2.895994

H 2.534504 -3.761693 -1.585259

H 4.487652 4.098544 -1.251178

H 5.808370 3.781008 1.589238

H 2.864633 1.643139 -2.527263

2b-c11 , delta G = 0.4970 kcal/mol, population = 13.72 %

C 3.770211 -1.398739 1.176442

C 2.505199 -2.023055 0.580182

C 2.188154 -1.783515 -0.895732

C 2.588551 -0.370322 -1.363288

C 3.430528 0.457433 -0.533558

C 3.691765 1.783643 -0.960111

C 4.393607 2.657316 -0.141832

C 4.824968 2.236206 1.112183

C 4.587398 0.928061 1.542514

C 3.903721 0.047189 0.733547

C 5.021065 -2.187009 0.727602

C 2.878006 -2.827371 -1.781731

O 3.268705 2.277822 -2.139609

C -0.058102 -1.124677 -0.496031

C -1.446810 -1.459626 -0.773400

C -2.454843 -0.736157 -0.245438

C -3.845360 -1.000293 -0.460905

C -4.828293 -0.252970 0.090570

C -6.235365 -0.486257 -0.102052

C -7.195539 0.270201 0.451606

C -8.666381 0.062408 0.266752

C -9.304106 1.205746 -0.559010

C -9.229281 2.596939 0.069928

C -9.361411 -0.125277 1.624145

O 0.789573 -1.991512 -1.122777

O 0.357168 -0.209425 0.187905

O 1.793290 -2.709602 1.275433

O 3.685915 -1.485050 2.588258

O 2.163953 -0.002616 -2.464352

O 4.660649 3.947489 -0.508785

O 5.490658 3.087448 1.924458

H -10.438650 -0.245640 1.488734

H -9.707629 3.332493 -0.579502

H -8.196867 2.917905 0.221779

H -8.823111 1.226506 -1.540643

H -10.352466 0.943340 -0.731497

H -8.803475 -0.855822 -0.312480

H -6.897699 1.101228 1.087091

H -9.737066 2.633531 1.035262

H -8.979670 -1.011045 2.134280

H -9.197448 0.731942 2.279653

H -6.516531 -1.324883 -0.734439

H -4.551823 0.585721 0.725030

H -4.107290 -1.841330 -1.096561

H -2.198602 0.106750 0.390300

H -1.631919 -2.312584 -1.412949

H 4.940318 0.623188 2.516255

H 2.911165 -2.037443 2.782596

H 5.854179 -1.833084 1.333533

H 4.883529 -3.254315 0.904139

H 5.264890 -2.016167 -0.318572

H 2.600710 -3.826576 -1.449112

H 3.959296 -2.727687 -1.757930

H 2.533723 -2.674715 -2.802910

H 4.255526 4.118979 -1.370868

H 5.563225 3.949438 1.487710

H 2.748315 1.554599 -2.577446

2b-c7 , delta G = 0.8308 kcal/mol, population = 7.81 %

C 3.439629 2.056054 0.065148

C 1.924408 1.836924 0.105487

C 1.366286 0.714641 0.978455

C 2.281124 -0.526792 0.997256

C 3.616205 -0.452851 0.454316

C 4.373224 -1.648245 0.376373

C 5.607640 -1.655648 -0.257745

C 6.097508 -0.487563 -0.833610

C 5.370876 0.703685 -0.752347

C 4.149487 0.725180 -0.116342

C 3.930825 2.719096 1.370884

C 1.133148 1.201649 2.413247

O 3.947693 -2.826427 0.871724

C -0.005469 -0.201263 -0.732735

C -1.360543 -0.509977 -1.159611

C -2.466293 -0.249668 -0.431723

C -3.801223 -0.548441 -0.856621

C -4.889128 -0.267493 -0.104319

C -6.245966 -0.551092 -0.491592

C -7.310793 -0.260503 0.271057

C -8.736323 -0.535424 -0.089975

C -9.513607 0.792777 -0.210212

C -10.952394 0.630016 -0.697463

C -9.366556 -1.483557 0.942427

O 0.062883 0.349173 0.513291

O 0.995041 -0.406051 -1.393600

O 1.197339 2.559013 -0.536111

O 3.734100 2.937670 -1.003011

O 1.814658 -1.559144 1.491601

O 6.367163 -2.787774 -0.366605

O 7.290430 -0.490395 -1.469426

H -10.387658 -1.747299 0.665213

H -10.984128 0.092622 -1.648916

H -11.419837 1.604756 -0.849791

H -9.505623 1.295733 0.762696

H -8.971310 1.444924 -0.900083

H -8.754622 -1.026058 -1.069034

H -7.147822 0.217148 1.236201

H -11.565888 0.079919 0.017566

H -8.790891 -2.406932 1.022720

H -9.394317 -1.014653 1.929794

H -6.397022 -1.027494 -1.457068

H -4.744416 0.205799 0.863963

H -3.929609 -1.020066 -1.826713

H -2.350513 0.217711 0.540584

H -1.426133 -0.970269 -2.137284

H 5.775929 1.598941 -1.199832

H 2.880080 3.232840 -1.359044

H 3.326928 3.595991 1.606810

H 3.923861 2.029435 2.212054

H 4.959017 3.034787 1.198684

H 0.482008 2.074478 2.397927

H 2.063793 1.457447 2.911178

H 0.650121 0.395758 2.962734

H 5.881237 -3.530688 0.018864

H 7.659026 -1.386305 -1.446175

H 3.040951 -2.666071 1.241902

2b-c26 , delta G = 1.0222 kcal/mol, population = 5.65 %

C 3.607322 1.975568 0.049198

C 2.076689 1.912245 0.058564

C 1.387385 0.859293 0.924681

C 2.170723 -0.467640 0.975802

C 3.524496 -0.531529 0.480373

C 4.165383 -1.795209 0.447921

C 5.417205 -1.931563 -0.135067

C 6.038160 -0.826260 -0.708379

C 5.426742 0.429967 -0.672226

C 4.189923 0.580035 -0.084893

C 4.127732 2.619588 1.353600

C 1.170580 1.381242 2.349743

O 3.610009 -2.918664 0.941879

C -0.024048 0.068385 -0.813925

C -1.390860 -0.148948 -1.258481

C -2.485992 0.150568 -0.529402

C -3.831657 -0.075911 -0.964689

C -4.908203 0.227879 -0.204844

C -6.275049 0.007592 -0.599467

C -7.320769 0.317326 0.183075

C -8.774187 0.104561 -0.117615

C -9.389891 -0.791443 0.982113

C -10.912755 -0.895242 0.926600

C -9.046414 -0.427755 -1.523291

O 0.065686 0.619946 0.430332

O 0.969342 -0.210778 -1.458384

O 1.441412 2.705865 -0.595695

O 4.012289 2.796857 -1.031504

O 1.586183 -1.445338 1.456087

O 6.067996 -3.132859 -0.196744

O 7.248324 -0.954093 -1.296929

H -10.117207 -0.475947 -1.718208

H -11.255251 -1.390932 0.017120

H -11.374279 0.095146 0.962571

H -8.943873 -1.788319 0.907026

H -9.094448 -0.394398 1.957531

H -9.258824 1.086971 -0.024765

H -7.114151 0.753263 1.158686

H -11.290091 -1.469765 1.774765

H -8.601810 0.215635 -2.284337

H -8.638584 -1.434119 -1.648665

H -6.436375 -0.431711 -1.578270

H -4.744955 0.667023 0.776533

H -3.977835 -0.515153 -1.947373

H -2.352036 0.588637 0.454171

H -1.474556 -0.587072 -2.244915

H 5.932794 1.274164 -1.116092

H 3.202430 3.191311 -1.394419

H 3.609073 3.558389 1.550941

H 4.027617 1.956335 2.210012

H 5.186926 2.825941 1.205164

H 2.110422 1.545202 2.868785

H 0.593979 0.634726 2.892814

H 0.613346 2.316190 2.311847

H 5.497267 -3.818561 0.178403

H 7.527439 -1.880793 -1.248437

H 2.707988 -2.666148 1.270429

2b-c12 , delta G = 1.3799 kcal/mol, population = 3.09 %

C 3.591327 -1.519463 1.221965

C 2.288427 -2.025222 0.595406

C 2.038129 -1.773427 -0.890730

C 2.583290 -0.408866 -1.356887

C 3.474210 0.345079 -0.507983

C 3.866767 1.638814 -0.933326

C 4.618322 2.453137 -0.098097

C 4.973854 2.004719 1.170102

C 4.607464 0.726350 1.599544

C 3.871316 -0.095630 0.774826

C 4.773613 -2.425301 0.810929

C 2.652330 -2.886075 -1.748252

O 3.526017 2.159928 -2.127925

C -0.146465 -0.897211 -0.571519

C -1.549991 -1.094097 -0.900625

C -2.498884 -0.268988 -0.413210

C -3.899273 -0.387308 -0.684813

C -4.820646 0.456810 -0.167429

C -6.235795 0.370657 -0.415416

C -7.127216 1.213838 0.128161

C -8.614952 1.202194 -0.052918

C -9.318253 1.084194 1.322148

C -9.081128 -0.239730 2.046262

C -9.120964 0.167176 -1.056978

O 0.633627 -1.850645 -1.159270

O 0.332026 -0.019682 0.120715

O 1.493301 -2.629617 1.276476

O 3.462784 -1.587081 2.631195

O 2.227069 -0.014316 -2.472632

O 5.009291 3.711910 -0.463049

O 5.688662 2.799452 1.997664

H -10.207618 0.226481 -1.140360

H -8.015774 -0.419124 2.205660

H -9.479878 -1.085427 1.483152

H -8.979966 1.909609 1.955487

H -10.390602 1.233252 1.167513

H -8.882867 2.195724 -0.436504

H -6.752976 1.992732 0.790143

H -9.569512 -0.235769 3.022535

H -8.698028 0.338737 -2.048339

H -8.865776 -0.850245 -0.756742

H -6.569343 -0.424570 -1.073984

H -4.483474 1.259308 0.484354

H -4.221419 -1.195322 -1.335437

H -2.181851 0.545027 0.232679

H -1.795598 -1.926744 -1.546545

H 4.901434 0.398220 2.585247

H 2.632644 -2.059214 2.807721

H 4.531079 -3.473795 0.987420

H 5.061214 -2.284219 -0.228488

H 5.619679 -2.148485 1.438637

H 2.274378 -3.851895 -1.416209

H 3.737120 -2.886018 -1.694535

H 2.351728 -2.712714 -2.779885

H 4.643337 3.913157 -1.336141

H 5.851699 3.647696 1.558383

H 2.955259 1.484110 -2.578111

2b-c13 , delta G = 1.5462 kcal/mol, population = 2.33 %

C -3.678483 -1.594869 -1.037262

C -2.342785 -2.069694 -0.457511

C -1.998846 -1.714678 0.988352

C -2.504519 -0.313012 1.385984

C -3.434902 0.391118 0.536346

C -3.784987 1.719294 0.885186

C -4.571450 2.483250 0.034528

C -5.004854 1.948773 -1.174758

C -4.681435 0.635676 -1.527219

C -3.911094 -0.135887 -0.685491

C -4.842441 -2.444240 -0.480027

C -2.565768 -2.756333 1.959891

O -3.368902 2.321110 2.016352

C 0.168891 -0.887286 0.476192

C 1.588861 -1.083210 0.725781

C 2.511909 -0.291686 0.142480

C 3.925707 -0.415480 0.328731

C 4.819678 0.401382 -0.273693

C 6.246488 0.307119 -0.112539

C 7.109527 1.134465 -0.722020

C 8.604385 1.155357 -0.582058

C 9.220165 -0.104332 0.038082

C 9.121401 -1.348869 -0.843675

C 9.013985 2.417261 0.202222

O -0.580797 -1.789320 1.174458

O -0.344725 -0.049892 -0.239992

O -1.594694 -2.728797 -1.141204

O -3.642894 -1.775183 -2.441664

O -2.081492 0.155235 2.448626

O -4.923470 3.772423 0.325531

O -5.755022 2.692594 -2.018275

H 10.100415 2.525631 0.207655

H 9.628251 -1.185934 -1.798085

H 8.085828 -1.612828 -1.062005

H 10.274399 0.105133 0.239101

H 8.763574 -0.292889 1.014703

H 9.015009 1.262744 -1.594530

H 6.698752 1.914053 -1.360507

H 9.590779 -2.208148 -0.360591

H 8.584168 3.318287 -0.239342

H 8.669185 2.350935 1.236546

H 6.611507 -0.481642 0.536868

H 4.449430 1.187255 -0.927722

H 4.281643 -1.204563 0.985026

H 2.161981 0.497575 -0.516961

H 1.868017 -1.887294 1.393839

H -5.035547 0.240581 -2.467526

H -2.823333 -2.259231 -2.634707

H -5.723631 -2.205471 -1.074220

H -4.624602 -3.507849 -0.582893

H -5.061079 -2.212504 0.560036

H -2.207404 -3.745898 1.680182

H -3.651863 -2.755581 1.966257

H -2.207883 -2.508217 2.957428

H -4.504633 4.031473 1.158686

H -5.881347 3.573938 -1.635724

H -2.782250 1.670372 2.482694

2b-c20 , delta G = 1.5995 kcal/mol, population = 2.13 %

C -3.562175 -1.965165 0.122315

C -2.030775 -1.924943 0.133145

C -1.326587 -0.845288 0.953360

C -2.087499 0.495432 0.940995

C -3.441215 0.557512 0.445105

C -4.063974 1.827789 0.359242

C -5.316431 1.956413 -0.224099

C -5.955868 0.836073 -0.745589

C -5.362252 -0.426009 -0.656631

C -4.124943 -0.568087 -0.068202

C -4.093794 -2.550589 1.449580

C -1.124640 -1.303905 2.402158

O -3.490369 2.963591 0.801342

C 0.100478 -0.157782 -0.814687

C 1.470725 0.018340 -1.266421

C 2.561156 -0.265423 -0.524091

C 3.909779 -0.078140 -0.968663

C 4.982530 -0.363792 -0.196470

C 6.351872 -0.178808 -0.599784

C 7.393946 -0.465629 0.195734

C 8.849473 -0.296007 -0.119035

C 9.531699 0.573002 0.965960

C 9.044730 2.019892 1.020659

C 9.134460 0.206475 -1.533811

O 0.000197 -0.650798 0.453205

O -0.887749 0.107197 -1.473030

O -1.406642 -2.756912 -0.483091

O -3.977201 -2.821790 -0.926965

O -1.486712 1.484930 1.374791

O -5.950291 3.163081 -0.336200

O -7.166784 0.955524 -1.334356

H 8.685701 1.184203 -1.716561

H 9.534860 2.559783 1.833051

H 7.966662 2.069088 1.188325

H 9.371279 0.099259 1.939023

H 10.610345 0.553117 0.786792

H 9.300111 -1.293286 -0.023964

H 7.183241 -0.854242 1.190519

H 9.260906 2.554182 0.093799

H 10.210830 0.300006 -1.688426

H 8.746600 -0.484292 -2.284416

H 6.519600 0.215690 -1.596537

H 4.813964 -0.757302 0.803206

H 4.061559 0.315502 -1.969657

H 2.420955 -0.658001 0.477662

H 1.561487 0.411169 -2.271145

H -5.882209 -1.281362 -1.061329

H -3.173132 -3.245751 -1.268770

H -3.985502 -1.856361 2.279991

H -5.155559 -2.747701 1.307120

H -3.588705 -3.488235 1.683908

H -0.533633 -0.544926 2.911436

H -0.587771 -2.251397 2.411121

H -2.069451 -1.422078 2.924854

H -5.369081 3.856098 0.008155

H -7.432050 1.887525 -1.325978

H -2.590772 2.712568 1.137635

# Table S74. Geometry data of conformers of structure 2b'

2b'-c1 , delta G = 0.0000 kcal/mol, population = 39.71 %

C -3.615799 -1.555557 1.133838

C -2.334800 -2.060543 0.463003

C -2.087789 -1.721305 -1.006113

C -2.600801 -0.317062 -1.382768

C -3.465015 0.406179 -0.481374

C -3.828163 1.732623 -0.824146

C -4.552372 2.513314 0.065496

C -4.908165 1.998249 1.307984

C -4.570798 0.687503 1.656206

C -3.862526 -0.101046 0.776298

C -4.826014 -2.403454 0.681129

C -2.734879 -2.764506 -1.924232

O -3.484095 2.316391 -1.988451

C 0.118988 -0.922455 -0.644758

C 1.516026 -1.131008 -0.994395

C 2.487008 -0.365659 -0.456052

C 3.883690 -0.503041 -0.738520

C 4.827598 0.277163 -0.164796

C 6.240405 0.166488 -0.414809

C 7.162048 0.933047 0.187076

C 8.640087 0.847405 -0.024662

C 9.353153 0.382926 1.262644

C 8.972846 -1.028128 1.702204

C 9.193073 2.202136 -0.490852

O -0.687017 -1.815225 -1.289696

O -0.334572 -0.082408 0.108076

O -1.556493 -2.736774 1.094222

O -3.474292 -1.709996 2.535010

O -2.242660 0.136238 -2.475454

O -4.915272 3.800773 -0.219630

O -5.595740 2.759020 2.188814

H 8.718740 2.523813 -1.419406

H 9.522108 -1.320461 2.599219

H 9.200779 -1.755347 0.918526

H 10.431938 0.436087 1.088806

H 9.134094 1.096563 2.064042

H 8.834959 0.103418 -0.804391

H 6.830401 1.682893 0.904347

H 7.906210 -1.103090 1.921296

H 9.016319 2.972908 0.263880

H 10.269940 2.137353 -0.660030

H 6.560000 -0.589772 -1.127313

H 4.512419 1.042310 0.540558

H 4.183591 -1.273809 -1.442763

H 2.192821 0.410662 0.244688

H 1.738299 -1.924209 -1.695979

H -4.864524 0.308052 2.623418

H -2.665773 -2.230245 2.672141

H -4.610534 -3.466793 0.791618

H -5.117568 -2.192213 -0.345348

H -5.659181 -2.142372 1.332424

H -2.445021 -2.532016 -2.947288

H -2.371033 -3.756099 -1.659098

H -3.818710 -2.748247 -1.853838

H -4.551432 4.045403 -1.082464

H -5.740772 3.636136 1.803137

H -2.933742 1.655309 -2.483738

2b'-c5 , delta G = 0.1155 kcal/mol, population = 32.67 %

C 3.528950 -1.992203 0.139602

C 1.998876 -1.916259 0.133897

C 1.311905 -0.819509 0.946068

C 2.105764 0.502386 0.942750

C 3.463153 0.533169 0.454217

C 4.114296 1.789023 0.367378

C 5.371058 1.888407 -0.212518

C 5.987166 0.752755 -0.728988

C 5.365681 -0.495743 -0.638120

C 4.123675 -0.608634 -0.053500

C 4.034472 -2.580962 1.475395

C 1.080183 -1.273692 2.391665

O 3.564447 2.938485 0.804454

C -0.077677 -0.096599 -0.838306

C -1.438158 0.112231 -1.305930

C -2.543715 -0.145752 -0.576931

C -3.881840 0.074600 -1.038213

C -4.971289 -0.185054 -0.280552

C -6.329474 0.036615 -0.701741

C -7.396523 -0.216760 0.070539

C -8.823660 0.004715 -0.318617

C -9.498475 1.028674 0.615737

C -8.873425 2.420056 0.560220

C -9.592229 -1.325654 -0.306714

O -0.003945 -0.592131 0.430159

O 0.923831 0.144988 -1.485315

O 1.361987 -2.733045 -0.489561

O 3.935145 -2.864554 -0.899955

O 1.525987 1.505161 1.374529

O 6.031735 3.080452 -0.325932

O 7.202132 0.843716 -1.314469

H -10.632118 -1.168081 -0.600845

H -7.827961 2.399601 0.873649

H -9.406228 3.115499 1.211580

H -10.556297 1.088772 0.343823

H -9.464890 0.644914 1.641065

H -8.844111 0.409650 -1.336036

H -7.234398 -0.615733 1.071190

H -8.905193 2.821937 -0.455854

H -9.145986 -2.045521 -0.994585

H -9.588196 -1.765956 0.693761

H -6.479371 0.437770 -1.700849

H -4.826916 -0.583667 0.720793

H -4.011147 0.472876 -2.040515

H -2.425426 -0.541816 0.426249

H -1.507525 0.507508 -2.311414

H 5.867696 -1.363763 -1.038354

H 3.124649 -3.267497 -1.251928

H 3.508441 -3.507156 1.709146

H 3.931219 -1.880332 2.301039

H 5.093569 -2.799943 1.345556

H 0.519065 -2.207059 2.392750

H 2.014837 -1.416728 2.926174

H 0.502435 -0.500224 2.894313

H 5.464491 3.787394 0.013366

H 7.487707 1.769710 -1.308570

H 2.658805 2.708406 1.139184

2b'-c6 , delta G = 0.8704 kcal/mol, population = 9.12 %

C -3.740488 -1.478566 1.193446

C -2.459720 -2.034025 0.563839

C -2.183756 -1.760792 -0.914249

C -2.656265 -0.360883 -1.353124

C -3.525114 0.412627 -0.498801

C -3.860175 1.729294 -0.902848

C -4.592688 2.555392 -0.062353

C -4.982744 2.096757 1.192028

C -4.672326 0.797006 1.600456

C -3.957159 -0.037185 0.769371

C -4.959176 -2.325980 0.763369

C -2.846212 -2.820767 -1.802105

O -3.481511 2.260219 -2.081619

C 0.038550 -1.006138 -0.553163

C 1.434740 -1.276658 -0.860997

C 2.419361 -0.506930 -0.354624

C 3.815655 -0.705252 -0.600620

C 4.773132 0.093200 -0.076623

C 6.185159 -0.071043 -0.301095

C 7.118594 0.740079 0.219354

C 8.593841 0.609271 0.008464

C 9.299355 0.360803 1.358136

C 10.797450 0.086498 1.236738

C 9.134774 1.858386 -0.705710

O -0.782560 -1.903840 -1.171959

O -0.402966 -0.116520 0.148016

O -1.702710 -2.695323 1.235496

O -3.619956 -1.572247 2.602289

O -2.267200 0.042614 -2.454588

O -4.929949 3.835052 -0.407615

O -5.677591 2.902539 2.025917

H 9.001296 2.746939 -0.082757

H 11.341262 0.951675 0.854424

H 10.987419 -0.752843 0.562634

H 9.131894 1.226281 2.008138

H 8.816135 -0.490146 1.845818

H 8.773169 -0.260860 -0.632248

H 6.798764 1.569933 0.847980

H 11.222864 -0.165252 2.210105

H 10.197960 1.757146 -0.925861

H 8.610292 2.024057 -1.647996

H 6.493751 -0.901156 -0.931729

H 4.470037 0.921937 0.558702

H 4.103537 -1.536457 -1.237997

H 2.137718 0.323558 0.286663

H 1.645155 -2.119240 -1.506410

H -4.992175 0.462055 2.575761

H -2.822578 -2.099429 2.773734

H -5.234329 -2.157498 -0.275392

H -5.795897 -2.021648 1.390845

H -4.763227 -3.386523 0.925281

H -2.530471 -2.638082 -2.827567

H -2.519032 -3.811833 -1.491239

H -3.930058 -2.768045 -1.754496

H -4.545237 4.038302 -1.272143

H -5.800322 3.765370 1.602226

H -2.931120 1.571236 -2.537306

2b'-c7 , delta G = 1.0373 kcal/mol, population = 6.88 %

C -3.566679 1.996257 0.081960

C -2.038805 1.885367 0.086241

C -1.380982 0.801393 0.938667

C -2.206491 -0.500578 0.977136

C -3.559167 -0.518119 0.475115

C -4.235809 -1.762136 0.419845

C -5.486437 -1.854085 -0.174536

C -6.071413 -0.723082 -0.735412

C -5.425009 0.514680 -0.675410

C -4.188781 0.620244 -0.077626

C -4.067212 2.629955 1.398773

C -1.142128 1.300934 2.368203

O -3.716277 -2.908134 0.900860

C 0.001514 -0.012679 -0.813812

C 1.360962 -0.254041 -1.269095

C 2.466829 0.025279 -0.548443

C 3.805016 -0.218775 -0.997414

C 4.893266 0.070179 -0.248646

C 6.252634 -0.165062 -0.658851

C 7.318421 0.129103 0.101100

C 8.745763 -0.106580 -0.280124

C 9.386516 -1.121010 0.690742

C 10.811158 -1.527624 0.317684

C 9.510257 1.226298 -0.311190

O -0.068803 0.526126 0.437079

O -1.001045 -0.261906 -1.456145

O -1.380530 2.664349 -0.562871

O -3.948462 2.849924 -0.981692

O -1.654469 -1.500131 1.450750

O -6.170582 -3.035266 -0.259254

O -7.280019 -0.807556 -1.334869

H 10.537753 1.084212 -0.647554

H 10.851962 -1.918719 -0.702253

H 11.176257 -2.308127 0.988064

H 9.373976 -0.698119 1.701067

H 8.753779 -2.012119 0.721067

H 8.765630 -0.541925 -1.285007

H 7.154712 0.573524 1.081872

H 11.505692 -0.688724 0.381991

H 9.029292 1.933616 -0.988540

H 9.539536 1.676565 0.684696

H 6.404732 -0.607045 -1.640396

H 4.746898 0.509454 0.735264

H 3.935480 -0.657329 -1.982611

H 2.348527 0.461676 0.437859

H 1.429165 -0.688209 -2.258478

H -5.903202 1.379951 -1.109364

H -3.127052 3.217658 -1.346745

H -3.983890 1.948344 2.242423

H -5.120619 2.867825 1.256427

H -3.522882 3.550476 1.612366

H -0.553250 2.216546 2.337377

H -2.073891 1.491934 2.892491

H -0.589509 0.529730 2.901665

H -5.622319 -3.742827 0.108728

H -7.585508 -1.726639 -1.303009

H -2.810310 -2.686479 1.240296

2b'-c8 , delta G = 1.4502 kcal/mol, population = 3.42 %

C -3.659330 -1.358167 1.339278

C -2.399218 -1.975659 0.725434

C -2.172194 -1.843243 -0.779966

C -2.653886 -0.485664 -1.329772

C -3.476536 0.376403 -0.515473

C -3.804270 1.659220 -1.020842

C -4.483927 2.572233 -0.227055

C -4.828500 2.233609 1.077833

C -4.525546 0.967916 1.587068

C -3.862992 0.048525 0.804127

C -4.901668 -2.213890 1.006527

C -2.865321 -2.978699 -1.542034

O -3.466191 2.075197 -2.256482

C 0.064542 -1.072246 -0.564353

C 1.448850 -1.379093 -0.891981

C 2.453415 -0.585374 -0.468697

C 3.841678 -0.814260 -0.733161

C 4.817645 -0.000230 -0.270791

C 6.224251 -0.195609 -0.503802

C 7.174918 0.617460 -0.018004

C 8.646688 0.439869 -0.226384

C 9.359316 0.016480 1.081140

C 9.282010 1.021887 2.229864

C 9.265278 1.707462 -0.834584

O -0.780546 -2.017040 -1.070439

O -0.349034 -0.119089 0.066701

O -1.618646 -2.568813 1.432776

O -3.508710 -1.345989 2.747554

O -2.306877 -0.190635 -2.478861

O -4.812195 3.823988 -0.669489

O -5.472546 3.124355 1.864795

H 10.343277 1.584202 -0.960335

H 9.732252 1.979365 1.962181

H 9.815772 0.641946 3.102949

H 8.936064 -0.937482 1.406945

H 10.409052 -0.175237 0.838228

H 8.786146 -0.379993 -0.937643

H 6.867311 1.474523 0.577018

H 8.250407 1.210020 2.533848

H 8.829178 1.920327 -1.811887

H 9.097497 2.578737 -0.198774

H 6.513381 -1.054895 -1.104008

H 4.534960 0.864918 0.324169

H 4.107697 -1.684421 -1.326509

H 2.196017 0.290617 0.120145

H 1.633664 -2.269708 -1.478015

H -4.812040 0.726398 2.599645

H -2.687474 -1.827513 2.939674

H -4.718756 -3.262483 1.243594

H -5.202690 -2.118782 -0.034349

H -5.715633 -1.847565 1.630832

H -2.529650 -3.937305 -1.149077

H -3.946915 -2.917521 -1.464385

H -2.582863 -2.895428 -2.589848

H -4.462921 3.942869 -1.564241

H -5.598306 3.948997 1.371682

H -2.948692 1.336039 -2.670495

2b'-c29 , delta G = 1.4947 kcal/mol, population = 3.18 %

C 3.539334 -1.993153 -0.117011

C 2.015216 -1.883552 -0.017257

C 1.409587 -0.862873 0.945115

C 2.235153 0.436525 1.023435

C 3.563186 0.487000 0.460739

C 4.241447 1.731521 0.459930

C 5.464258 1.863416 -0.182660

C 6.019900 0.772719 -0.844139

C 5.371328 -0.465110 -0.841094

C 4.161811 -0.610918 -0.198191

C 4.104825 -2.738894 1.112512

C 1.261130 -1.455754 2.351039

O 3.748499 2.842503 1.040900

C -0.073391 0.057968 -0.664295

C -1.457071 0.306102 -1.033697

C -2.522497 -0.040527 -0.281938

C -3.883555 0.205540 -0.654258

C -4.933223 -0.147114 0.121714

C -6.312711 0.084283 -0.219395

C -7.332688 -0.271945 0.577137

C -8.797817 -0.060107 0.332690

C -9.125290 0.497763 -1.056798

C -10.620570 0.546911 -1.367654

C -9.375109 0.807694 1.467083

O 0.069646 -0.562557 0.541967

O 0.891099 0.365851 -1.338948

O 1.318643 -2.616428 -0.680013

O 3.856274 -2.754824 -1.268820

O 1.708605 1.402424 1.587121

O 6.147604 3.047482 -0.217846

O 7.201051 0.896645 -1.489802

H -8.980902 1.824885 1.405346

H -11.078589 -0.438790 -1.250529

H -10.789482 0.871056 -2.396222

H -8.699966 1.502252 -1.151381

H -8.628827 -0.121149 -1.809130

H -9.271880 -1.048406 0.415423

H -7.093159 -0.751652 1.524358

H -11.151226 1.241133 -0.714322

H -10.462632 0.856808 1.412415

H -9.108984 0.401329 2.444598

H -6.504164 0.571078 -1.169769

H -4.737276 -0.639385 1.071408

H -4.064851 0.699239 -1.604754

H -2.350795 -0.537846 0.667011

H -1.580750 0.803386 -1.987358

H 5.825595 -1.297735 -1.356888

H 3.015547 -3.107084 -1.603859

H 3.563437 -3.671549 1.275261

H 4.072809 -2.133838 2.015777

H 5.146734 -2.968772 0.893397

H 2.225367 -1.663574 2.805905

H 0.733534 -0.727118 2.963783

H 0.681816 -2.376411 2.298146

H 5.618851 3.728171 0.222273

H 7.511666 1.810990 -1.409167

H 2.857253 2.599230 1.403778

2b'-c19 , delta G = 1.7821 kcal/mol, population = 1.95 %

C -3.735717 -1.471809 1.210223

C -2.404556 -2.003468 0.670716

C -2.080232 -1.809866 -0.810019

C -2.595220 -0.461262 -1.352463

C -3.510636 0.334030 -0.569502

C -3.863128 1.618003 -1.054557

C -4.631840 2.473293 -0.277774

C -5.044633 2.074926 0.989875

C -4.719471 0.805952 1.476879

C -3.967042 -0.056116 0.710174

C -4.908167 -2.368545 0.755300

C -2.655999 -2.953812 -1.652849

O -3.465427 2.090790 -2.251474

C 0.094463 -0.931671 -0.420207

C 1.511618 -1.165409 -0.653496

C 2.443368 -0.316623 -0.173871

C 3.855386 -0.474749 -0.348150

C 4.758252 0.402800 0.145531

C 6.184460 0.276061 -0.004137

C 7.053172 1.169681 0.494655

C 8.551042 1.127397 0.419614

C 9.101438 -0.026207 -0.426508

C 10.613752 0.028099 -0.638908

C 9.125219 1.137280 1.848820

O -0.664656 -1.903499 -1.005338

O -0.409091 -0.014100 0.198149

O -1.642833 -2.575299 1.415239

O -3.689382 -1.508547 2.625083

O -2.190584 -0.113727 -2.467374

O -4.985524 3.725111 -0.700198

O -5.776788 2.909777 1.760947

H 10.207815 1.263778 1.838563

H 11.162442 -0.099579 0.295271

H 10.914318 0.984363 -1.075205

H 8.832425 -0.977570 0.043939

H 8.608148 -0.011842 -1.402225

H 8.864147 2.068037 -0.055422

H 6.648077 2.021407 1.037836

H 10.935892 -0.762885 -1.318707

H 8.700001 1.953876 2.435207

H 8.893256 0.198945 2.358369

H 6.542210 -0.591073 -0.548993

H 4.396274 1.267796 0.696202

H 4.202090 -1.342759 -0.901637

H 2.102530 0.549309 0.386725

H 1.781551 -2.046160 -1.221127

H -5.059859 0.516537 2.459739

H -2.854510 -1.945051 2.861105

H -4.692821 -3.416826 0.964511

H -5.138744 -2.242899 -0.300260

H -5.781329 -2.066122 1.332116

H -3.741988 -2.953688 -1.648632

H -2.308322 -2.819068 -2.675387

H -2.293936 -3.905858 -1.267480

H -4.582305 3.890714 -1.564312

H -5.906785 3.745137 1.287320

H -2.889492 1.389534 -2.653862

2b'-c16 , delta G = 1.9126 kcal/mol, population = 1.57 %

C -3.432298 2.021971 0.186849

C -1.912252 1.839449 0.146008

C -1.286129 0.691114 0.935710

C -2.165129 -0.575579 0.928672

C -3.530167 -0.507871 0.465120

C -4.264797 -1.716306 0.371622

C -5.534681 -1.723181 -0.187928

C -6.082184 -0.541179 -0.676922

C -5.377993 0.661996 -0.578110

C -4.121909 0.683352 -0.013363

C -3.870843 2.625288 1.539548

C -1.006011 1.109323 2.383894

O -3.785291 -2.906372 0.782009

C 0.036016 -0.093211 -0.876265

C 1.380709 -0.335010 -1.373964

C 2.506620 -0.083054 -0.674679

C 3.832574 -0.313135 -1.165537

C 4.938794 -0.042363 -0.436544

C 6.288693 -0.256292 -0.887706

C 7.370793 0.025095 -0.145671

C 8.788459 -0.185065 -0.577656

C 9.485023 -1.284108 0.259976

C 9.622576 -0.989374 1.753669

C 9.564346 1.141197 -0.546564

O 0.005260 0.389575 0.398967

O -0.985423 -0.294994 -1.505220

O -1.233847 2.610468 -0.491872

O -3.798367 2.935441 -0.831948

O -1.646529 -1.620354 1.337437

O -6.274983 -2.866881 -0.307414

O -7.309554 -0.542362 -1.243325

H 10.606639 0.981102 -0.831767

H 10.206066 -0.085443 1.936870

H 10.129927 -1.814148 2.257584

H 8.932463 -2.217887 0.125358

H 10.478664 -1.446132 -0.168940

H 8.772389 -0.542158 -1.611895

H 7.221724 0.443397 0.847336

H 8.649782 -0.863163 2.232917

H 9.126741 1.861224 -1.239811

H 9.552171 1.588795 0.448910

H 6.418528 -0.667519 -1.885741

H 4.815779 0.366701 0.563502

H 3.938261 -0.720974 -2.166737

H 2.415849 0.322857 0.327394

H 1.419438 -0.735787 -2.378917

H -5.827732 1.567281 -0.957456

H -2.969403 3.275309 -1.206806

H -3.275179 3.507541 1.776753

H -3.804991 1.907212 2.353837

H -4.913333 2.922139 1.431633

H -0.466730 0.295365 2.864860

H -0.390919 2.008059 2.390041

H -1.921982 1.297519 2.936076

H -5.749338 -3.615753 0.008132

H -7.655254 -1.447641 -1.247348

H -2.860952 -2.742401 1.104194

2b'-c17 , delta G = 1.9421 kcal/mol, population = 1.49 %

C -3.678083 -1.547040 1.090970

C -2.345050 -2.047980 0.527408

C -1.996536 -1.740612 -0.928580

C -2.497244 -0.352476 -1.374496

C -3.432888 0.379723 -0.555167

C -3.786019 1.692805 -0.955118

C -4.581052 2.484186 -0.138291

C -5.019727 1.992967 1.087347

C -4.692668 0.695441 1.490327

C -3.913952 -0.103826 0.682672

C -4.842454 -2.419456 0.570524

C -2.565338 -2.811976 -1.866221

O -3.365509 2.253912 -2.105315

C 0.172344 -0.908925 -0.430973

C 1.592381 -1.119880 -0.667602

C 2.518335 -0.328744 -0.088480

C 3.932345 -0.469472 -0.261023

C 4.829504 0.340833 0.345406

C 6.256881 0.229505 0.201006

C 7.121560 1.046244 0.822524

C 8.621334 0.982305 0.785426

C 9.211281 0.136201 -0.348845

C 8.995293 0.721126 -1.744239

C 9.133330 0.503711 2.158150

O -0.578524 -1.825701 -1.108517

O -0.340601 -0.050348 0.260231

O -1.603795 -2.693560 1.231192

O -3.638564 -1.669336 2.501868

O -2.067104 0.079954 -2.449461

O -4.936541 3.760165 -0.479013

O -5.778134 2.764317 1.898107

H 10.222624 0.565478 2.200619

H 7.936117 0.822081 -1.984193

H 9.452703 0.089120 -2.508029

H 10.285228 0.039299 -0.167374

H 8.808748 -0.880207 -0.295683

H 8.978611 2.011794 0.653021

H 6.711887 1.821760 1.466674

H 9.446434 1.713713 -1.819781

H 8.726045 1.109904 2.969443

H 8.839802 -0.533858 2.332266

H 6.620863 -0.561243 -0.446793

H 4.461480 1.134896 0.990750

H 4.285521 -1.267772 -0.907599

H 2.171118 0.472741 0.557526

H 1.869326 -1.937289 -1.320242

H -5.050989 0.334573 2.442715

H -2.828836 -2.163160 2.710771

H -5.062582 -2.230722 -0.477951

H -5.723095 -2.157174 1.155529

H -4.623468 -3.477775 0.716700

H -2.204523 -2.598932 -2.870785

H -2.211666 -3.793103 -1.552836

H -3.651482 -2.806875 -1.874789

H -4.512583 3.990454 -1.318011

H -5.905927 3.630151 1.482059

H -2.772630 1.588963 -2.543084

# Table S75. Geometry data of conformers of structure 3a

3a-c6 , delta G = 0.0000 kcal/mol, population = 37.36 %

C 2.899783 -1.980395 -0.492492

C 1.855541 -1.899188 0.630093

C 1.679024 -0.587763 1.399482

C 2.243863 0.623105 0.640284

C 3.575182 0.413263 0.034291

C 4.514612 1.481296 -0.068809

C 5.750638 1.242623 -0.681977

C 6.066187 -0.041545 -1.172431

C 5.149737 -1.063361 -1.058659

C 3.906593 -0.847516 -0.454668

C 2.160628 -1.970672 -1.844283

C 2.446085 -0.721265 2.725531

C 4.307337 2.792144 0.543427

C -0.571658 -0.325803 0.725928

C -1.917552 -0.054603 1.206707

C -2.957375 0.023916 0.351797

C -4.312509 0.278772 0.736463

C -5.326546 0.344891 -0.155859

C -6.702298 0.589455 0.187855

C -7.692851 0.645324 -0.714896

C -9.135177 0.895572 -0.408922

C -10.008644 -0.296505 -0.847244

C -9.691622 -1.596475 -0.112755

C -9.606791 2.190061 -1.089342

O 0.319150 -0.377583 1.758389

O -0.223323 -0.506862 -0.424813

O 1.239871 -2.889982 0.942466

O 3.613136 -3.205422 -0.333539

O 1.653518 1.683518 0.641290

O 6.708034 2.173171 -0.825099

O 7.266649 -0.259901 -1.750797

H -9.513052 2.113340 -2.175664

H -10.360501 -2.398881 -0.429870

H -9.806421 -1.472318 0.967260

H -11.055921 -0.026831 -0.682791

H -9.892691 -0.439785 -1.926926

H -9.242073 1.013745 0.674716

H -7.449934 0.499471 -1.766821

H -8.666048 -1.920452 -0.299986

H -10.655720 2.383759 -0.854936

H -9.017197 3.046641 -0.759000

H -6.933547 0.730411 1.240640

H -5.102254 0.202426 -1.210272

H -4.520948 0.419803 1.793185

H -2.757658 -0.117768 -0.706637

H -2.045111 0.077273 2.273087

H 5.399108 -2.040434 -1.446223

H 2.951203 -3.893212 -0.173741

H 1.442380 -2.790674 -1.874540

H 1.624631 -1.036725 -1.995316

H 2.897937 -2.104947 -2.635544

H 2.013809 -1.540986 3.296572

H 3.500425 -0.924762 2.546556

H 2.350527 0.205541 3.290440

H 3.412160 2.936679 1.151133

O 5.111342 3.718636 0.416541

H 6.354033 3.014628 -0.429674

H 7.772722 0.567461 -1.738295

3a-c11 , delta G = 0.3131 kcal/mol, population = 22.01 %

C 3.318334 -2.025850 0.023246

C 1.854780 -1.935776 0.478652

C 1.191133 -0.563356 0.617479

C 1.937038 0.535817 -0.154675

C 3.407361 0.515356 -0.007818

C 4.166030 1.722945 -0.023422

C 5.560477 1.651091 0.080128

C 6.201341 0.401874 0.215226

C 5.452659 -0.753827 0.237760

C 4.058633 -0.707326 0.130766

C 3.343481 -2.516917 -1.436727

C 1.201528 -0.189854 2.109012

C 3.556830 3.051392 -0.015949

C -0.435362 -1.012839 -1.035512

C -1.844852 -0.989171 -1.387831

C -2.835266 -0.615010 -0.551683

C -4.223699 -0.605711 -0.903817

C -5.198581 -0.242800 -0.040052

C -6.602804 -0.228206 -0.354941

C -7.555527 0.127807 0.519485

C -9.025467 0.148528 0.243968

C -9.602015 1.567091 0.420482

C -9.024254 2.592654 -0.551175

C -9.751880 -0.847714 1.161168

O -0.179518 -0.612764 0.242386

O 0.459508 -1.365407 -1.780776

O 1.266537 -2.942761 0.792319

O 3.978165 -2.967475 0.867835

O 1.322580 1.395290 -0.752254

O 6.367692 2.724081 0.083692

O 7.546166 0.349061 0.323919

H -10.823763 -0.846966 0.951934

H -7.946983 2.706926 -0.415924

H -9.486537 3.571369 -0.408969

H -10.686957 1.509652 0.293546

H -9.428738 1.890758 1.452424

H -9.185667 -0.156427 -0.795715

H -7.257740 0.427433 1.523673

H -9.196034 2.288858 -1.587076

H -9.375091 -1.861761 1.018884

H -9.613445 -0.579355 2.211719

H -6.888195 -0.526156 -1.360661

H -4.918340 0.059028 0.966350

H -4.490286 -0.911242 -1.911631

H -2.577932 -0.303073 0.455253

H -2.055646 -1.308928 -2.400426

H 5.954787 -1.705447 0.334595

H 3.396905 -3.738735 0.931027

H 2.868721 -1.800690 -2.102749

H 4.383576 -2.659718 -1.729292

H 2.811122 -3.465564 -1.512722

H 0.630999 -0.935976 2.659278

H 2.218542 -0.159057 2.496151

H 0.739711 0.788750 2.236942

H 2.469392 3.114716 0.056318

O 4.223591 4.087818 -0.063273

H 5.783978 3.524766 -0.005754

H 7.896341 1.252913 0.289423

3a-c15 , delta G = 0.4920 kcal/mol, population = 16.27 %

C 2.999810 -1.980697 -0.491941

C 1.959339 -1.916700 0.635307

C 1.763189 -0.607735 1.404151

C 2.302516 0.612287 0.641127

C 3.634256 0.425693 0.028506

C 4.553658 1.510380 -0.081049

C 5.790543 1.293093 -0.700461

C 6.126813 0.014071 -1.190612

C 5.229719 -1.023967 -1.070369

C 3.985965 -0.829648 -0.460306

C 2.254626 -1.986916 -1.840363

C 2.538624 -0.726338 2.726717

C 4.326131 2.818342 0.530123

C -0.494435 -0.387273 0.740319

C -1.842751 -0.138846 1.226477

C -2.887840 -0.080904 0.376340

C -4.245295 0.151402 0.767001

C -5.264891 0.197351 -0.120223

C -6.642592 0.421629 0.229990

C -7.638535 0.463193 -0.667987

C -9.082931 0.688103 -0.350269

C -9.908819 -0.548642 -0.763013

C -11.384038 -0.472264 -0.373262

C -9.579840 1.970851 -1.035789

O 0.401485 -0.421509 1.769062

O -0.147659 -0.563973 -0.411577

O 1.363120 -2.917952 0.951967

O 3.736211 -3.192210 -0.334162

O 1.693231 1.661944 0.644183

O 6.730136 2.240552 -0.850143

O 7.328010 -0.183264 -1.775017

H -8.968772 2.826868 -0.745623

H -11.496971 -0.299985 0.700341

H -11.894913 -1.406107 -0.615765

H -9.818264 -0.687235 -1.845731

H -9.459005 -1.430917 -0.299693

H -9.180867 0.813789 0.733403

H -7.398913 0.322699 -1.721144

H -11.903286 0.331687 -0.897091

H -9.529472 1.871522 -2.123469

H -10.613384 2.187824 -0.764238

H -6.869932 0.562092 1.283842

H -5.043760 0.055359 -1.175375

H -4.450761 0.292312 1.824328

H -2.690814 -0.222000 -0.682688

H -1.967694 -0.006426 2.293111

H 5.494702 -1.997046 -1.457647

H 3.087591 -3.891789 -0.171021

H 1.551513 -2.820108 -1.865961

H 1.700747 -1.063315 -1.990456

H 2.990695 -2.108894 -2.634762

H 3.595393 -0.912324 2.543024

H 2.430053 0.199503 3.290848

H 2.122971 -1.552489 3.300826

H 3.431514 2.948268 1.141918

O 5.112994 3.758741 0.397758

H 6.363187 3.076049 -0.453774

H 7.818876 0.653245 -1.766256

3a-c28 , delta G = 0.8936 kcal/mol, population = 8.25 %

C 3.115556 -1.889717 -0.697139

C 2.068002 -2.023333 0.417760

C 1.769980 -0.820345 1.315974

C 2.218204 0.510192 0.690371

C 3.562580 0.490158 0.078049

C 4.397109 1.646519 0.092738

C 5.650715 1.587978 -0.528543

C 6.085695 0.395516 -1.143569

C 5.269478 -0.713879 -1.142732

C 4.011115 -0.677488 -0.532964

C 2.379671 -1.810671 -2.048379

C 2.547549 -1.018325 2.627753

C 4.066872 2.862868 0.832822

C -0.494512 -0.691510 0.651832

C -1.857010 -0.566461 1.145200

C -2.899676 -0.486102 0.293750

C -4.268694 -0.353727 0.690490

C -5.283935 -0.275657 -0.199550

C -6.672240 -0.134669 0.153762

C -7.652881 -0.056617 -0.759458

C -9.119809 0.120342 -0.505706

C -9.602278 1.404513 -1.219721

C -11.120629 1.566108 -1.257885

C -9.502503 0.096590 0.972989

O 0.396946 -0.772868 1.682190

O -0.131865 -0.735147 -0.507907

O 1.549829 -3.094814 0.621626

O 3.941412 -3.052128 -0.658042

O 1.529912 1.504375 0.794970

O 6.516812 2.613346 -0.565925

O 7.301690 0.350406 -1.728877

H -9.157457 -0.818324 1.457583

H -11.399481 2.445083 -1.842281

H -11.544049 1.688939 -0.259840

H -9.145487 2.267893 -0.725761

H -9.218657 1.396580 -2.243985

H -9.625638 -0.719475 -1.003036

H -7.373104 -0.102474 -1.810243

H -11.594741 0.695101 -1.718004

H -9.069083 0.947223 1.505210

H -10.584272 0.144870 1.092875

H -6.906125 -0.090325 1.212292

H -5.049387 -0.318225 -1.260570

H -4.485958 -0.312952 1.754018

H -2.690703 -0.521587 -0.771816

H -1.993920 -0.536896 2.218162

H 5.609775 -1.619060 -1.624226

H 3.347149 -3.812064 -0.580378

H 1.738763 -2.684862 -2.167644

H 1.761375 -0.918501 -2.111725

H 3.127181 -1.798244 -2.841423

H 2.196188 -1.930778 3.106220

H 3.616386 -1.100688 2.438002

H 2.364438 -0.169128 3.285368

H 3.160223 2.861072 1.440852

O 4.781750 3.867536 0.811630

H 6.086401 3.371863 -0.087179

H 7.728137 1.215595 -1.626850

3a-c19 , delta G = 1.0536 kcal/mol, population = 6.30 %

C 3.383726 -2.012657 0.071608

C 1.918376 -1.904866 0.517114

C 1.261686 -0.526145 0.622797

C 2.016622 0.551765 -0.170279

C 3.486135 0.526892 -0.017652

C 4.251268 1.729764 -0.059199

C 5.645022 1.653046 0.050011

C 6.278772 0.403975 0.216603

C 5.523860 -0.746930 0.263819

C 4.130436 -0.695665 0.151272

C 3.414008 -2.538961 -1.375927

C 1.267849 -0.120342 2.105805

C 3.648955 3.061127 -0.083663

C -0.358526 -1.003492 -1.027881

C -1.765619 -0.978356 -1.389294

C -2.758474 -0.577288 -0.568611

C -4.144478 -0.566802 -0.929984

C -5.122902 -0.176967 -0.082075

C -6.524917 -0.164356 -0.407125

C -7.483839 0.212046 0.452323

C -8.950353 0.233072 0.157674

C -9.488842 1.675823 0.262181

C -10.952781 1.825119 -0.148357

C -9.689898 -0.729248 1.100819

O -0.107407 -0.576405 0.242250

O 0.538483 -1.378376 -1.759611

O 1.323316 -2.901615 0.850087

O 4.034546 -2.936845 0.942224

O 1.408942 1.400934 -0.789205

O 6.457824 2.721611 0.030609

O 7.622939 0.346575 0.330834

H -10.752476 -0.777783 0.860861

H -11.622826 1.287275 0.524042

H -11.115877 1.442955 -1.159507

H -9.353604 2.028782 1.290186

H -8.870447 2.318987 -0.369746

H -9.098441 -0.108813 -0.872260

H -7.194500 0.530119 1.453041

H -11.250367 2.875440 -0.137443

H -9.281306 -1.737987 1.023884

H -9.592312 -0.402159 2.139527

H -6.803691 -0.485481 -1.407738

H -4.847987 0.147717 0.918674

H -4.406485 -0.894976 -1.931881

H -2.505148 -0.243454 0.432303

H -1.972540 -1.320299 -2.395419

H 6.020677 -1.698605 0.384567

H 3.449743 -3.704087 1.019862

H 2.877021 -3.486414 -1.431917

H 2.946645 -1.836644 -2.061751

H 4.454877 -2.694138 -1.659215

H 0.689324 -0.850164 2.669412

H 2.283347 -0.088753 2.496908

H 0.812858 0.864195 2.210545

H 2.561762 3.131714 -0.014612

O 4.321200 4.092722 -0.153711

H 5.878425 3.522924 -0.079044

H 7.978071 1.247537 0.277014

3a-c26 , delta G = 1.4828 kcal/mol, population = 3.05 %

C 3.038384 -2.017329 -0.285405

C 1.949244 -1.913486 0.792265

C 1.633068 -0.550501 1.413422

C 2.131715 0.620918 0.552875

C 3.499930 0.464055 0.018237

C 4.355153 1.591177 -0.159699

C 5.630082 1.397258 -0.704941

C 6.064328 0.102663 -1.058323

C 5.228238 -0.975868 -0.872202

C 3.949237 -0.806300 -0.331437

C 2.351260 -2.200549 -1.652317

C 2.348273 -0.482728 2.773358

C 4.020395 2.932753 0.313700

C -0.600347 -0.531948 0.636329

C -1.977149 -0.283080 1.034056

C -2.981818 -0.339543 0.136277

C -4.358675 -0.090487 0.438194

C -5.336176 -0.145912 -0.494892

C -6.727362 0.112317 -0.233210

C -7.674889 0.055187 -1.181804

C -9.151436 0.264878 -1.006330

C -9.561557 0.961606 0.296836

C -9.129066 2.424647 0.385996

C -9.871319 -1.088710 -1.162882

O 0.246798 -0.417495 1.700219

O -0.193553 -0.815768 -0.473769

O 1.408036 -2.917231 1.189505

O 3.840728 -3.157211 0.016115

O 1.458781 1.622596 0.422464

O 6.516796 2.385320 -0.906349

O 7.299958 -0.069883 -1.574848

H -9.590334 -1.584277 -2.094043

H -9.558033 3.003731 -0.435608

H -8.044977 2.531813 0.333352

H -10.651020 0.907850 0.374312

H -9.175374 0.398185 1.151953

H -9.480664 0.899353 -1.839499

H -7.366138 -0.211605 -2.190602

H -9.465212 2.875324 1.321858

H -9.614363 -1.754265 -0.335777

H -10.953699 -0.945546 -1.166343

H -6.990926 0.368859 0.787568

H -5.066194 -0.396417 -1.518048

H -4.614173 0.159016 1.464146

H -2.732524 -0.585441 -0.892171

H -2.155283 -0.040896 2.073520

H 5.569097 -1.961736 -1.153226

H 3.230548 -3.878230 0.227268

H 1.697395 -3.072647 -1.619274

H 1.752765 -1.331200 -1.913661

H 3.125628 -2.355413 -2.403434

H 1.959118 -1.274579 3.410967

H 3.422930 -0.611553 2.656513

H 2.151299 0.484838 3.234148

H 3.090919 3.060833 0.871799

O 4.755100 3.905389 0.126396

H 6.083075 3.228253 -0.604496

H 7.737912 0.793898 -1.627699

3a-c41 , delta G = 1.4878 kcal/mol, population = 3.02 %

C 3.364090 -2.033193 0.099072

C 1.919279 -1.912656 0.604060

C 1.267395 -0.530648 0.693498

C 1.987454 0.519779 -0.166387

C 3.461808 0.501531 -0.069378

C 4.223381 1.702492 -0.176285

C 5.620055 1.629815 -0.108621

C 6.260581 0.387070 0.077652

C 5.509329 -0.762338 0.185165

C 4.113093 -0.714866 0.116488

C 3.332755 -2.589948 -1.337172

C 1.341383 -0.077809 2.161218

C 3.618554 3.031785 -0.230406

C -0.430787 -1.058189 -0.863504

C -1.852859 -1.025097 -1.160157

C -2.793895 -0.539571 -0.324042

C -4.195385 -0.508397 -0.617715

C -5.113081 -0.010422 0.241574

C -6.529220 0.036824 -0.012729

C -7.411519 0.544772 0.862208

C -8.897632 0.661678 0.692792

C -9.437297 -0.018000 -0.570941

C -10.962966 -0.060365 -0.645412

C -9.297908 2.146710 0.778649

O -0.117145 -0.588252 0.377740

O 0.427341 -1.472103 -1.620213

O 1.341220 -2.898280 0.995042

O 4.051037 -2.939325 0.960002

O 1.353400 1.345240 -0.790804

O 6.430303 2.697539 -0.188824

O 7.607766 0.333958 0.150636

H -8.890954 2.610249 1.679236

H -11.290871 -0.627557 -1.518526

H -11.397150 0.937842 -0.721411

H -9.041931 0.495903 -1.453111

H -9.054489 -1.041427 -0.610301

H -9.349340 0.159527 1.559963

H -7.025402 0.942753 1.798863

H -11.384660 -0.541289 0.241170

H -8.914655 2.694098 -0.085796

H -10.381480 2.261977 0.805487

H -6.868544 -0.363514 -0.962162

H -4.772196 0.385218 1.195445

H -4.520023 -0.905912 -1.575120

H -2.481521 -0.144205 0.636921

H -2.115503 -1.424563 -2.131461

H 6.010929 -1.709732 0.318952

H 3.463769 -3.697711 1.089489

H 4.361075 -2.749029 -1.661268

H 2.796431 -3.539321 -1.350559

H 2.834675 -1.902982 -2.016965

H 0.785063 -0.786001 2.772730

H 2.373330 -0.039781 2.505694

H 0.895895 0.912219 2.254835

H 2.533330 3.103930 -0.136773

O 4.287118 4.060741 -0.354033

H 5.846506 3.494549 -0.306182

H 7.959752 1.232879 0.056706

3a-c31 , delta G = 1.7156 kcal/mol, population = 2.06 %

C 3.358645 -2.006652 0.206329

C 1.881013 -1.906771 0.611375

C 1.194886 -0.538792 0.627972

C 1.941692 0.509514 -0.211210

C 3.408298 0.525433 -0.034388

C 4.149062 1.740079 -0.136753

C 5.541974 1.700142 -0.000726

C 6.198533 0.477569 0.251929

C 5.466963 -0.684407 0.356793

C 4.074939 -0.670428 0.218324

C 3.434188 -2.623929 -1.203131

C 1.166308 -0.052136 2.086027

C 3.520393 3.054399 -0.252468

C -0.382854 -1.136924 -1.024422

C -1.782233 -1.150323 -1.415760

C -2.795584 -0.714400 -0.639204

C -4.173910 -0.730734 -1.028815

C -5.169014 -0.295392 -0.223572

C -6.564324 -0.294560 -0.576557

C -7.535010 0.141598 0.240755

C -8.994604 0.162779 -0.090988

C -9.524448 1.608536 -0.246405

C -9.431079 2.484724 1.002704

C -9.796536 -0.635195 0.948771

O -0.165089 -0.638636 0.226086

O 0.534797 -1.536047 -1.716574

O 1.298881 -2.896878 0.984533

O 4.009306 -2.857682 1.149052

O 1.326841 1.309012 -0.886687

O 6.332953 2.782724 -0.072358

O 7.541376 0.456685 0.391123

H -9.484810 -1.680802 0.958692

H -9.831421 3.479481 0.798487

H -8.397746 2.609683 1.332338

H -8.977680 2.086058 -1.063976

H -10.569103 1.544811 -0.566125

H -9.123104 -0.324121 -1.062496

H -7.255368 0.508428 1.225914

H -10.000263 2.067546 1.835318

H -9.654905 -0.236440 1.954998

H -10.863682 -0.598281 0.718540

H -6.826581 -0.671056 -1.562314

H -4.913048 0.083265 0.763186

H -4.414923 -1.112794 -2.016717

H -2.565290 -0.325014 0.347034

H -1.963850 -1.547751 -2.406243

H 5.981044 -1.615990 0.543524

H 3.443939 -3.635619 1.257319

H 2.922951 -3.587228 -1.208176

H 2.963779 -1.980567 -1.942763

H 4.484450 -2.769241 -1.455279

H 0.590605 -0.760688 2.678911

H 2.174017 0.019273 2.491692

H 0.692594 0.928204 2.128752

H 2.431021 3.105955 -0.206398

O 4.172959 4.093868 -0.374251

H 5.739101 3.563241 -0.240090

H 7.878307 1.360483 0.289006

3a-c35 , delta G = 1.8380 kcal/mol, population = 1.67 %

C 3.275171 -2.049623 0.102146

C 1.830141 -1.911231 0.602087

C 1.195626 -0.520953 0.688177

C 1.932755 0.519408 -0.169612

C 3.406099 0.483429 -0.064195

C 4.182935 1.674887 -0.166031

C 5.578165 1.585125 -0.088548

C 6.202001 0.334568 0.101959

C 5.435997 -0.805691 0.203734

C 4.041014 -0.741015 0.125676

C 3.241803 -2.601829 -1.335869

C 1.270268 -0.068315 2.156002

C 3.594570 3.011297 -0.225017

C -0.505103 -1.022305 -0.875475

C -1.924493 -0.956934 -1.179925

C -2.856662 -0.442229 -0.351345

C -4.255096 -0.370710 -0.652099

C -5.159345 0.161030 0.201321

C -6.571938 0.255110 -0.056608

C -7.438187 0.793266 0.815847

C -8.915234 0.989661 0.631942

C -9.544438 0.182077 -0.509127

C -9.566048 -1.327354 -0.270255

C -9.201775 2.495309 0.469842

O -0.188320 -0.559283 0.367669

O 0.348070 -1.454372 -1.627551

O 1.238667 -2.889276 0.991929

O 3.946915 -2.967045 0.962838

O 1.312087 1.351217 -0.799000

O 6.401888 2.642906 -0.162846

O 7.547887 0.264865 0.184498

H -10.278031 2.678904 0.462483

H -10.128423 -1.566064 0.635968

H -8.561495 -1.735165 -0.150927

H -10.570983 0.535220 -0.640102

H -9.029734 0.411332 -1.447420

H -9.394775 0.672917 1.567305

H -7.038536 1.177422 1.752329

H -10.041717 -1.848172 -1.103598

H -8.762908 3.074742 1.284249

H -8.783171 2.863246 -0.469562

H -6.922423 -0.136994 -1.005791

H -4.808562 0.549650 1.154511

H -4.588044 -0.762314 -1.609057

H -2.538383 -0.051526 0.609556

H -2.191748 -1.352985 -2.151364

H 5.925118 -1.759169 0.340499

H 3.347676 -3.716273 1.090940

H 4.269203 -2.772818 -1.656803

H 2.693656 -3.544332 -1.353779

H 2.754712 -1.906786 -2.015414

H 2.301321 -0.043676 2.504303

H 0.837545 0.927572 2.247436

H 0.702293 -0.768754 2.765722

H 2.509591 3.096497 -0.139679

O 4.276337 4.032145 -0.343688

H 5.828645 3.446994 -0.283793

H 7.911573 1.159413 0.093436

# **Table S76**. Geometry data of conformers of structure 3a'

3a'-c4 , delta G = 0.0000 kcal/mol, population = 36.11 %

C -3.049031 -1.952357 -0.576860

C -1.990821 -2.008107 0.535202

C -1.710938 -0.755601 1.368758

C -2.170080 0.532734 0.669700

C -3.512062 0.467310 0.059246

C -4.352930 1.617711 0.004443

C -5.599272 1.519561 -0.625626

C -6.022888 0.292068 -1.176962

C -5.201541 -0.811119 -1.106847

C -3.949239 -0.735529 -0.488099

C -2.321169 -1.961530 -1.934261

C -2.492262 -0.891284 2.686561

C -4.038516 2.869515 0.688931

C 0.558699 -0.636544 0.708208

C 1.915745 -0.478744 1.204131

C 2.966559 -0.426255 0.358882

C 4.329711 -0.269381 0.764377

C 5.354331 -0.221570 -0.118228

C 6.736906 -0.061972 0.245990

C 7.735609 -0.013857 -0.648980

C 9.186190 0.149144 -0.324409

C 9.750152 1.440814 -0.949217

C 9.104905 2.716170 -0.413389

C 9.979167 -1.075704 -0.806278

O -0.338894 -0.674203 1.735969

O 0.202799 -0.738764 -0.451172

O -1.444428 -3.055609 0.786489

O -3.872809 -3.110878 -0.452182

O -1.487874 1.536806 0.713551

O -6.469115 2.536633 -0.728214

O -7.232441 0.206592 -1.770876

H 9.612612 -1.991301 -0.339279

H 9.558796 3.602702 -0.860554

H 9.225628 2.788313 0.670678

H 10.827006 1.465208 -0.759177

H 9.628631 1.388931 -2.036384

H 9.290202 0.225099 0.763120

H 7.492993 -0.099064 -1.707508

H 8.034858 2.741408 -0.628754

H 9.894524 -1.190480 -1.889936

H 11.037327 -0.964729 -0.560143

H 6.965476 0.025426 1.305040

H 5.132935 -0.306425 -1.179415

H 4.535723 -0.186093 1.827561

H 2.770407 -0.507328 -0.706627

H 2.045173 -0.403229 2.275793

H -5.532980 -1.742265 -1.542821

H -3.279996 -3.872932 -0.388316

H -1.685079 -2.844740 -2.003534

H -3.071252 -1.992739 -2.724117

H -1.700322 -1.076655 -2.054352

H -2.328901 -0.002256 3.294732

H -2.128758 -1.768585 3.218723

H -3.558225 -1.002350 2.496754

H -3.148727 2.896740 1.320681

O -4.750446 3.873021 0.600004

H -6.047878 3.323651 -0.290931

H -7.667867 1.072080 -1.721412

3a'-c10 , delta G = 0.3683 kcal/mol, population = 19.38 %

C -3.245934 -2.060300 0.075032

C -1.773493 -1.902481 0.481735

C -1.167730 -0.501060 0.587153

C -1.975017 0.550562 -0.187678

C -3.437497 0.475327 -0.003366

C -4.243390 1.651460 -0.025235

C -5.631790 1.527669 0.104770

C -6.219105 0.256581 0.277020

C -5.424488 -0.868040 0.306153

C -4.035727 -0.769451 0.171734

C -3.293751 -2.590151 -1.370520

C -1.166913 -0.103617 2.072940

C -3.683718 3.000620 -0.043888

C 0.450910 -0.901371 -1.091288

C 1.848870 -0.801845 -1.470789

C 2.826419 -0.335589 -0.664184

C 4.203829 -0.236919 -1.041520

C 5.159214 0.230276 -0.205038

C 6.553054 0.345977 -0.542390

C 7.483265 0.803070 0.309929

C 8.941352 0.947130 0.011154

C 9.787597 0.055467 0.941664

C 9.525153 -1.438474 0.771127

C 9.365906 2.418329 0.140900

O 0.196609 -0.496578 0.185288

O -0.438144 -1.314550 -1.814483

O -1.129509 -2.879690 0.780942

O -3.844023 -3.002772 0.964031

O -1.410359 1.418407 -0.823163

O -6.479793 2.568052 0.103242

O -7.558079 0.150308 0.414049

H 8.799938 3.054097 -0.541887

H 9.712898 -1.752081 -0.259152

H 8.490274 -1.691649 1.009834

H 10.842250 0.268052 0.744463

H 9.600876 0.351817 1.979328

H 9.116270 0.623194 -1.020099

H 7.172755 1.101196 1.310597

H 10.173630 -2.026726 1.423345

H 9.198834 2.780412 1.158541

H 10.428081 2.529751 -0.087009

H 6.849726 0.040438 -1.542449

H 4.869335 0.542786 0.795314

H 4.478532 -0.554345 -2.043326

H 2.564958 -0.011271 0.337793

H 2.064239 -1.134904 -2.478188

H -5.887758 -1.836298 0.428475

H -3.261112 -3.774721 0.990200

H -4.335334 -2.776138 -1.631248

H -2.864732 -1.873720 -2.067072

H -2.730624 -3.521559 -1.438558

H -2.176683 -0.112268 2.478988

H -0.749601 0.897433 2.176457

H -0.552279 -0.813157 2.624061

H -2.599366 3.104669 0.026347

O -4.387605 4.011821 -0.106092

H -5.930903 3.388800 -0.011580

H -7.949074 1.037194 0.373837

3a'-c12 , delta G = 0.7122 kcal/mol, population = 10.84 %

C -3.117677 -1.936949 -0.582311

C -2.062100 -1.990316 0.532422

C -1.787902 -0.737415 1.367240

C -2.248960 0.549913 0.667551

C -3.589307 0.480989 0.053979

C -4.433397 1.628951 -0.002052

C -5.678022 1.527485 -0.634957

C -6.096814 0.299067 -1.187898

C -5.272371 -0.801737 -1.116636

C -4.021685 -0.722824 -0.495028

C -2.386460 -1.943191 -1.937939

C -2.572295 -0.876084 2.682919

C -4.124169 2.881331 0.683744

C 0.483004 -0.610184 0.712687

C 1.837727 -0.443899 1.212120

C 2.890695 -0.386966 0.369835

C 4.251365 -0.218545 0.778995

C 5.278540 -0.166214 -0.100379

C 6.658518 0.006675 0.268101

C 7.661186 0.054574 -0.622868

C 9.107102 0.242633 -0.289313

C 9.616618 1.564164 -0.902946

C 11.050695 1.919829 -0.513750

C 9.920054 -0.970287 -0.768749

O -0.417137 -0.651790 1.738089

O 0.130730 -0.715302 -0.447542

O -1.513621 -3.036474 0.784774

O -3.938355 -3.097961 -0.460398

O -1.569614 1.555841 0.713339

O -6.550624 2.542060 -0.738939

O -7.304821 0.210401 -1.784495

H 10.968004 -0.880619 -0.481098

H 11.770646 1.200873 -0.907422

H 11.164851 1.947715 0.573023

H 9.532165 1.502789 -1.993134

H 8.948041 2.368803 -0.585181

H 9.202214 0.316565 0.799232

H 7.425255 -0.044849 -1.681429

H 11.321555 2.903158 -0.903142

H 9.530818 -1.893176 -0.335774

H 9.873705 -1.059718 -1.857360

H 6.881647 0.103158 1.327678

H 5.061559 -0.258002 -1.161901

H 4.453160 -0.128824 1.842473

H 2.698262 -0.472544 -0.695998

H 1.963541 -0.364668 2.283945

H -5.600121 -1.733604 -1.553849

H -3.343419 -3.858210 -0.394765

H -1.767937 -1.056427 -2.056082

H -1.747595 -2.824480 -2.006159

H -3.134532 -1.976173 -2.729631

H -2.207408 -1.752453 3.215673

H -3.637384 -0.990492 2.490227

H -2.413383 0.013198 3.291892

H -3.235858 2.910850 1.317477

O -4.838842 3.882784 0.593719

H -6.132684 3.330096 -0.300347

H -7.742909 1.074565 -1.735346

3a'-c21 , delta G = 0.9344 kcal/mol, population = 7.45 %

C -3.053052 -1.899372 -0.724558

C -2.020405 -2.035923 0.403641

C -1.760229 -0.848868 1.333874

C -2.210460 0.488108 0.726620

C -3.537471 0.465554 0.080674

C -4.381709 1.615014 0.093585

C -5.611407 1.563707 -0.573721

C -6.015623 0.382079 -1.230015

C -5.192927 -0.722245 -1.222506

C -3.957003 -0.692375 -0.568056

C -2.294435 -1.810207 -2.062107

C -2.564622 -1.081436 2.624102

C -4.090734 2.809661 0.882643

C 0.521177 -0.675504 0.726533

C 1.870484 -0.576918 1.257862

C 2.933722 -0.426117 0.440473

C 4.291116 -0.329763 0.882783

C 5.329456 -0.162310 0.031336

C 6.706821 -0.072320 0.437311

C 7.722293 0.113101 -0.420924

C 9.164664 0.209112 -0.033214

C 9.711441 1.645602 -0.215248

C 9.691854 2.185035 -1.645474

C 9.999861 -0.826285 -0.802035

O -0.394901 -0.796922 1.731101

O 0.185980 -0.670702 -0.443428

O -1.474709 -3.097798 0.586978

O -3.877833 -3.063791 -0.702882

O -1.536607 1.488948 0.867523

O -6.482268 2.584001 -0.618909

O -7.208743 0.340706 -1.860654

H 11.056884 -0.731859 -0.543512

H 8.676835 2.238390 -2.044247

H 10.284544 1.565515 -2.320837

H 9.135741 2.313857 0.430905

H 10.738748 1.656374 0.161477

H 9.239777 -0.019742 1.034059

H 7.496329 0.199374 -1.481498

H 10.108505 3.193612 -1.675242

H 9.677903 -1.840321 -0.559288

H 9.903036 -0.696062 -1.881535

H 6.916964 -0.162856 1.500192

H 5.125038 -0.091026 -1.034213

H 4.481203 -0.396924 1.950086

H 2.753202 -0.374931 -0.629602

H 1.983797 -0.626790 2.332860

H -5.510297 -1.618009 -1.736161

H -3.285843 -3.829002 -0.688212

H -1.651634 -2.683559 -2.178542

H -3.026192 -1.788965 -2.869296

H -1.676087 -0.916620 -2.105164

H -2.215937 -2.000310 3.092169

H -3.628072 -1.168424 2.409390

H -2.405228 -0.243617 3.302033

H -3.217280 2.789516 1.536960

O -4.805274 3.814894 0.855533

H -6.075868 3.334226 -0.108770

H -7.648091 1.199479 -1.757585

3a'-c20 , delta G = 1.1603 kcal/mol, population = 5.08 %

C -3.330215 -2.051831 0.113061

C -1.856399 -1.897589 0.515838

C -1.241019 -0.498920 0.600352

C -2.041050 0.546768 -0.189753

C -3.504175 0.483773 -0.005941

C -4.302299 1.664734 -0.047423

C -5.691708 1.552113 0.081555

C -6.287855 0.287743 0.272011

C -5.500706 -0.841400 0.320302

C -4.111002 -0.754103 0.187298

C -3.383161 -2.607749 -1.322389

C -1.236442 -0.079247 2.079934

C -3.733743 3.009793 -0.084943

C 0.372553 -0.938861 -1.072561

C 1.771807 -0.864798 -1.452777

C 2.757211 -0.407262 -0.650717

C 4.136599 -0.341314 -1.027865

C 5.102126 0.112449 -0.195607

C 6.498775 0.188166 -0.533154

C 7.441371 0.632131 0.312934

C 8.903948 0.716451 0.011452

C 9.684385 -0.242176 0.936215

C 11.176633 -0.333205 0.621254

C 9.386923 2.169284 0.145635

O 0.123108 -0.510974 0.197403

O -0.521636 -1.351671 -1.789713

O -1.218548 -2.874630 0.828518

O -3.934247 -2.973719 1.019723

O -1.470214 1.401972 -0.836780

O -6.532777 2.597940 0.062133

O -7.627778 0.192414 0.407565

H 9.275729 2.518093 1.175775

H 11.338928 -0.618832 -0.421328

H 11.657021 -1.084274 1.251427

H 9.542588 0.076055 1.974499

H 9.238235 -1.236912 0.853184

H 9.061386 0.390972 -1.022244

H 7.140264 0.956722 1.307955

H 11.688791 0.615009 0.791725

H 10.437453 2.262685 -0.131143

H 8.809164 2.831618 -0.501054

H 6.787204 -0.138370 -1.529151

H 4.819316 0.443265 0.800865

H 4.405035 -0.676428 -2.025616

H 2.501477 -0.066944 0.347431

H 1.982026 -1.213684 -2.455890

H -5.970538 -1.804581 0.456714

H -3.358878 -3.750859 1.056573

H -4.426247 -2.791015 -1.578996

H -2.949509 -1.907468 -2.032356

H -2.826847 -3.544319 -1.373644

H -2.246029 -0.074750 2.486568

H -0.812166 0.920334 2.168268

H -0.626453 -0.784680 2.641393

H -2.648928 3.107778 -0.013467

O -4.430837 4.024488 -0.164398

H -5.978175 3.413132 -0.064499

H -8.012757 1.081173 0.353152

3a'-c31 , delta G = 1.2318 kcal/mol, population = 4.51 %

C -3.099412 -1.958421 -0.521045

C -1.996085 -1.974126 0.547643

C -1.701716 -0.700004 1.343134

C -2.213725 0.565543 0.639128

C -3.579414 0.464357 0.088635

C -4.442298 1.598992 0.046880

C -5.711448 1.467409 -0.529060

C -6.134634 0.222745 -1.041059

C -5.291792 -0.864822 -0.983818

C -4.017163 -0.756189 -0.417632

C -2.426804 -1.979316 -1.906553

C -2.420471 -0.819216 2.697479

C -4.122115 2.869130 0.693787

C 0.534754 -0.559638 0.579923

C 1.910082 -0.375045 1.011717

C 2.921219 -0.326849 0.118928

C 4.299597 -0.148104 0.458244

C 5.281226 -0.105521 -0.472323

C 6.678420 0.071177 -0.176286

C 7.624667 0.112086 -1.128407

C 9.098920 0.311763 -0.937959

C 9.543945 0.323260 0.528711

C 11.060778 0.348678 0.713599

C 9.534969 1.580151 -1.695741

O -0.316053 -0.589316 1.646999

O 0.129262 -0.689075 -0.560398

O -1.422448 -3.007414 0.797105

O -3.895673 -3.128989 -0.342363

O -1.548253 1.581675 0.631830

O -6.603209 2.467086 -0.613686

O -7.365082 0.105491 -1.584614

H 10.620592 1.677493 -1.707325

H 11.502794 1.274610 0.342238

H 11.533498 -0.482978 0.184344

H 9.099917 1.187077 1.034050

H 9.143173 -0.565457 1.023936

H 9.592302 -0.538285 -1.429917

H 7.307248 0.012576 -2.164653

H 11.321356 0.261901 1.770152

H 9.190453 1.554888 -2.731181

H 9.114850 2.469486 -1.219825

H 6.948204 0.172372 0.869527

H 5.010195 -0.210735 -1.520162

H 4.553723 -0.044069 1.509140

H 2.678009 -0.430730 -0.934869

H 2.086196 -0.277678 2.074955

H -5.623458 -1.809538 -1.389498

H -3.286129 -3.878126 -0.283413

H -3.207566 -2.037327 -2.664573

H -1.827261 -1.086045 -2.066072

H -1.778155 -2.852405 -1.986821

H -3.492641 -0.946093 2.559537

H -2.240350 0.083551 3.280255

H -2.021897 -1.681412 3.229260

H -3.206815 2.924248 1.286107

O -4.855557 3.858005 0.616767

H -6.178603 3.269849 -0.209699

H -7.812525 0.964883 -1.536165

3a'-c25 , delta G = 1.3335 kcal/mol, population = 3.79 %

C -2.904417 -2.035750 -0.259998

C -1.843367 -1.830466 0.831355

C -1.659502 -0.443478 1.451334

C -2.230281 0.676995 0.569575

C -3.566371 0.406535 0.003496

C -4.502466 1.461066 -0.209374

C -5.740198 1.164971 -0.792880

C -6.059559 -0.162686 -1.147244

C -5.147593 -1.170454 -0.924195

C -3.903480 -0.898080 -0.345880

C -2.182875 -2.195460 -1.611307

C -2.411824 -0.427065 2.792618

C -4.290661 2.823830 0.272324

C 0.585240 -0.251092 0.726557

C 1.932922 0.081189 1.156960

C 2.958754 0.091677 0.279719

C 4.314763 0.407103 0.608784

C 5.304285 0.411483 -0.314580

C 6.682606 0.717759 -0.038095

C 7.630984 0.716173 -0.988526

C 9.090145 1.009744 -0.817337

C 9.937763 -0.147474 -1.399834

C 9.785829 -1.478368 -0.665349

C 9.498784 1.374344 0.609537

O -0.294111 -0.198026 1.769237

O 0.223034 -0.555979 -0.394842

O -1.213991 -2.778673 1.236190

O -3.627434 -3.225110 0.054649

O -1.635778 1.728893 0.444283

O -6.694233 2.078954 -1.029613

O -7.259717 -0.438299 -1.701050

H 8.974683 2.266993 0.955222

H 10.379311 -2.255075 -1.151440

H 8.744845 -1.809099 -0.656663

H 9.664406 -0.280053 -2.450712

H 10.986334 0.162525 -1.389095

H 9.301120 1.878422 -1.455768

H 7.331022 0.468117 -2.005093

H 10.121082 -1.408421 0.370949

H 9.284105 0.567992 1.312773

H 10.570381 1.577020 0.651059

H 6.938040 0.955915 0.989204

H 5.054029 0.166867 -1.344215

H 4.544217 0.650942 1.642047

H 2.744615 -0.156389 -0.756205

H 2.075185 0.321580 2.202331

H -5.400921 -2.181325 -1.208708

H -2.972252 -3.915819 0.227830

H -2.930081 -2.418568 -2.372514

H -1.644928 -1.289508 -1.880334

H -1.469464 -3.018129 -1.551294

H -1.975767 -1.180043 3.446777

H -3.468778 -0.644316 2.649640

H -2.308773 0.556528 3.249694

H -3.400465 3.020579 0.872505

O -5.087107 3.739230 0.049671

H -6.338546 2.954746 -0.721805

H -7.765596 0.385629 -1.780333

3a'-c38 , delta G = 1.3793 kcal/mol, population = 3.51 %

C -3.418416 -1.994799 0.182295

C -1.951326 -1.901787 0.626900

C -1.264619 -0.535285 0.677439

C -1.980762 0.517655 -0.180624

C -3.450562 0.539169 -0.047795

C -4.182126 1.757521 -0.167109

C -5.579166 1.723143 -0.084603

C -6.248832 0.501208 0.136170

C -5.525702 -0.664240 0.260077

C -4.129510 -0.655572 0.174123

C -3.456110 -2.611842 -1.228435

C -1.286639 -0.056611 2.138748

C -3.541621 3.068415 -0.239490

C 0.375697 -1.128030 -0.920509

C 1.788215 -1.144149 -1.254880

C 2.770627 -0.704714 -0.438972

C 4.162797 -0.725533 -0.770957

C 5.122799 -0.280385 0.072448

C 6.531507 -0.285904 -0.221573

C 7.458537 0.166798 0.638107

C 8.940904 0.224976 0.423325

C 9.413155 1.690947 0.562145

C 10.930707 1.855814 0.617643

C 9.404921 -0.414942 -0.884102

O 0.109013 -0.635796 0.322604

O -0.516183 -1.521279 -1.651516

O -1.377216 -2.897792 0.997989

O -4.100091 -2.841261 1.107403

O -1.343011 1.314369 -0.839658

O -6.361655 2.809583 -0.178034

O -7.595836 0.482363 0.226340

H 10.492717 -0.426777 -0.943791

H 11.409186 1.547618 -0.313182

H 11.358312 1.259481 1.427929

H 9.003892 2.267267 -0.273706

H 8.975052 2.111572 1.471754

H 9.397241 -0.327922 1.256551

H 7.118212 0.557400 1.595217

H 11.197832 2.899588 0.794143

H 9.058715 -1.446607 -0.966240

H 9.028607 0.139373 -1.747684

H 6.828245 -0.679679 -1.187943

H 4.824204 0.110530 1.042256

H 4.445709 -1.118709 -1.743214

H 2.501380 -0.309174 0.534995

H 2.011392 -1.545495 -2.235315

H -6.050786 -1.594794 0.419304

H -3.569951 -3.645795 1.196389

H -2.954828 -3.580425 -1.218233

H -4.498804 -2.746365 -1.515070

H -2.957318 -1.971359 -1.952022

H -0.731421 -0.767840 2.747729

H -2.307506 0.013240 2.509726

H -0.815986 0.923855 2.203032

H -2.456037 3.112843 -0.135060

O -4.180660 4.113727 -0.384381

H -5.759902 3.588659 -0.317121

H -7.929888 1.387082 0.121372

3a'-c36 , delta G = 1.4954 kcal/mol, population = 2.89 %

C -3.343390 -2.012565 0.125288

C -1.880159 -1.924215 0.582988

C -1.199059 -0.556819 0.670306

C -1.914039 0.513925 -0.166657

C -3.384856 0.525951 -0.043203

C -4.120904 1.743741 -0.138143

C -5.518225 1.701660 -0.065786

C -6.184090 0.472034 0.121269

C -5.456812 -0.693091 0.221777

C -4.060140 -0.676575 0.145300

C -3.367697 -2.593451 -1.300984

C -1.231386 -0.112853 2.142417

C -3.485667 3.058665 -0.175075

C 0.453356 -1.103851 -0.931611

C 1.868253 -1.111428 -1.255825

C 2.846753 -0.715768 -0.413169

C 4.241391 -0.731452 -0.734773

C 5.197954 -0.343552 0.140284

C 6.609275 -0.345690 -0.140054

C 7.531431 0.036211 0.757881

C 9.017455 0.077083 0.570261

C 9.559962 1.484258 0.921108

C 9.088725 2.597924 -0.012681

C 9.495759 -0.410560 -0.796695

O 0.177212 -0.644314 0.321904

O -0.433022 -1.477396 -1.679477

O -1.304440 -2.926455 0.934359

O -4.028108 -2.885200 1.023390

O -1.275439 1.329666 -0.801140

O -6.304810 2.786764 -0.138534

O -7.531555 0.445524 0.201987

H 9.087666 0.192478 -1.609300

H 9.475825 3.564202 0.316321

H 7.998584 2.663276 -0.032144

H 9.264042 1.722080 1.947048

H 10.652410 1.436812 0.917034

H 9.437254 -0.596938 1.329317

H 7.184276 0.364942 1.735772

H 9.430440 2.438196 -1.036871

H 10.584213 -0.354639 -0.853471

H 9.203098 -1.447348 -0.971285

H 6.912927 -0.676721 -1.127705

H 4.894002 -0.005321 1.128079

H 4.528976 -1.074464 -1.724495

H 2.572495 -0.363863 0.576062

H 2.097298 -1.472509 -2.250471

H -5.978897 -1.629443 0.354798

H -3.493410 -3.688167 1.098417

H -2.861200 -3.559269 -1.312114

H -4.407615 -2.725943 -1.598421

H -2.867359 -1.931611 -2.003959

H -0.764812 0.867537 2.232415

H -0.676860 -0.836114 2.737708

H -2.254520 -0.055480 2.509345

H -2.401122 3.105169 -0.061032

O -4.128154 4.104322 -0.300702

H -5.705717 3.571369 -0.256041

H -7.868807 1.350991 0.115369

3a'-c34 , delta G = 1.5412 kcal/mol, population = 2.67 %

C -3.265767 -2.048849 0.119315

C -1.782784 -1.888276 0.483985

C -1.171415 -0.486922 0.554148

C -1.995885 0.556035 -0.214045

C -3.453672 0.486106 0.006683

C -4.257908 1.663494 -0.013741

C -5.643193 1.544529 0.149878

C -6.228664 0.277339 0.354152

C -5.435501 -0.848254 0.381796

C -4.049968 -0.754621 0.214038

C -3.353232 -2.605094 -1.314365

C -1.130178 -0.068179 2.033479

C -3.696675 3.011185 -0.064633

C 0.401676 -0.922032 -1.158493

C 1.792267 -0.850616 -1.570204

C 2.796808 -0.399327 -0.788676

C 4.168572 -0.340242 -1.193929

C 5.153223 0.104705 -0.379564

C 6.543984 0.170191 -0.742713

C 7.505146 0.604460 0.087728

C 8.962010 0.675459 -0.247224

C 9.783602 -0.355367 0.564164

C 9.766513 -0.168064 2.081225

C 9.489666 2.106754 -0.061728

O 0.182053 -0.493431 0.116653

O -0.509074 -1.334395 -1.854669

O -1.132474 -2.862739 0.778439

O -3.842238 -2.973360 1.041113

O -1.445569 1.414655 -0.874178

O -6.489167 2.586534 0.152449

O -7.564329 0.175885 0.523220

H 10.559598 2.149998 -0.277498

H 8.756827 -0.250206 2.488398

H 10.167279 0.803935 2.373862

H 9.414081 -1.354748 0.318895

H 10.816457 -0.308626 0.205934

H 9.076321 0.406589 -1.301597

H 7.218590 0.933906 1.084001

H 10.377304 -0.933348 2.563929

H 8.977269 2.797564 -0.733300

H 9.336415 2.460094 0.959568

H 6.811452 -0.156892 -1.744479

H 4.891932 0.434978 0.622972

H 4.414998 -0.674860 -2.197499

H 2.563986 -0.060002 0.215385

H 1.979010 -1.198693 -2.578231

H -5.897464 -1.813575 0.529469

H -3.263976 -3.748938 1.061524

H -4.401706 -2.793428 -1.543929

H -2.941460 -1.902905 -2.035380

H -2.793959 -3.538997 -1.379970

H -0.504080 -0.772257 2.578672

H -2.128921 -0.066467 2.466042

H -0.706401 0.932481 2.111337

H -2.610933 3.114304 -0.019216

O -4.400246 4.022651 -0.126556

H -5.941581 3.404218 0.011950

H -7.954443 1.063001 0.479719

3a'-c29 , delta G = 1.6133 kcal/mol, population = 2.36 %

C -2.929442 -2.046769 -0.159502

C -1.886404 -1.801224 0.941160

C -1.697866 -0.388085 1.497271

C -2.234332 0.695450 0.550137

C -3.560704 0.414939 -0.033110

C -4.475922 1.469964 -0.321232

C -5.702979 1.161688 -0.920622

C -6.032934 -0.177613 -1.216961

C -5.141763 -1.185086 -0.920868

C -3.908076 -0.900727 -0.326007

C -2.182750 -2.289182 -1.484650

C -2.479271 -0.298957 2.819017

C -4.256770 2.851905 0.098582

C 0.564137 -0.254174 0.813991

C 1.903737 0.095449 1.256076

C 2.950263 0.049980 0.404919

C 4.299072 0.381135 0.748339

C 5.313019 0.320135 -0.145914

C 6.685075 0.639425 0.147784

C 7.661036 0.556713 -0.771506

C 9.118224 0.840589 -0.567984

C 9.967405 -0.429095 -0.817979

C 9.846643 -1.019952 -2.221146

C 9.451224 1.448897 0.793433

O -0.337025 -0.142468 1.833150

O 0.224825 -0.615444 -0.297768

O -1.277154 -2.735900 1.403937

O -3.678397 -3.206379 0.202267

O -1.622800 1.732446 0.387725

O -6.637550 2.075315 -1.226108

O -7.223179 -0.464132 -1.786364

H 8.888864 2.366957 0.972611

H 10.073865 -0.270104 -2.983487

H 8.845213 -1.405513 -2.419378

H 11.011913 -0.166545 -0.628320

H 9.693766 -1.181031 -0.071243

H 9.406030 1.562279 -1.344859

H 7.381509 0.239391 -1.772351

H 10.546737 -1.847565 -2.350345

H 9.228905 0.751555 1.604839

H 10.514237 1.689210 0.845211

H 6.909845 0.955827 1.160901

H 5.089802 0.007013 -1.163127

H 4.502301 0.691545 1.769145

H 2.760626 -0.259576 -0.619147

H 2.022580 0.396470 2.288590

H -5.402869 -2.205573 -1.160508

H -3.038641 -3.898847 0.420098

H -2.917291 -2.538235 -2.250112

H -1.622267 -1.407916 -1.787847

H -1.486256 -3.120045 -1.366368

H -2.069327 -1.027254 3.516599

H -3.536042 -0.507473 2.662131

H -2.371354 0.702857 3.233308

H -3.379475 3.066329 0.711606

O -5.034353 3.765505 -0.188748

H -6.276972 2.960505 -0.952670

H -7.714368 0.361767 -1.919380

3a'-c42 , delta G = 1.9214 kcal/mol, population = 1.40 %

C -3.354325 -1.994907 0.297338

C -1.879036 -1.881940 0.709189

C -1.191105 -0.515537 0.682750

C -1.918899 0.495906 -0.214583

C -3.386771 0.524705 -0.065452

C -4.119499 1.735820 -0.240528

C -5.515727 1.706348 -0.144441

C -6.183583 0.497415 0.143530

C -5.459309 -0.660111 0.321839

C -4.063763 -0.656718 0.223750

C -3.419476 -2.694871 -1.072860

C -1.189766 0.033305 2.119121

C -3.479605 3.041242 -0.384736

C 0.420469 -1.181978 -0.914086

C 1.826217 -1.209432 -1.275318

C 2.824188 -0.744125 -0.493386

C 4.209378 -0.773104 -0.852895

C 5.186529 -0.309052 -0.039936

C 6.588548 -0.319785 -0.364391

C 7.534267 0.143317 0.469524

C 9.011022 0.188019 0.216889

C 9.539041 1.641882 0.267535

C 9.328134 2.358854 1.599326

C 9.441743 -0.493847 -1.080739

O 0.176414 -0.635238 0.310801

O -0.484485 -1.608385 -1.609673

O -1.298307 -2.859833 1.116342

O -4.020709 -2.782505 1.284195

O -1.289640 1.259800 -0.919163

O -6.299021 2.786907 -0.286512

O -7.529824 0.483667 0.244771

H 10.529459 -0.483712 -1.165727

H 9.820756 3.333048 1.592741

H 9.746679 1.779973 2.426837

H 10.609021 1.607984 0.044617

H 9.069641 2.210681 -0.541220

H 9.485388 -0.341062 1.055214

H 7.210378 0.536228 1.429400

H 8.271373 2.529496 1.811157

H 9.110783 -1.533122 -1.119451

H 9.035354 0.023842 -1.952992

H 6.862970 -0.727410 -1.331597

H 4.908357 0.102075 0.927603

H 4.472554 -1.188582 -1.821396

H 2.573957 -0.319283 0.473247

H 2.030360 -1.643233 -2.246034

H -5.983163 -1.580912 0.533344

H -3.496695 -3.587088 1.403390

H -2.920751 -3.663092 -1.014128

H -4.467429 -2.842699 -1.332465

H -2.932380 -2.100376 -1.842314

H -2.204982 0.125280 2.500564

H -0.714935 1.013814 2.128825

H -0.628110 -0.649766 2.753917

H -2.393296 3.090617 -0.291009

O -4.119964 4.077991 -0.577476

H -5.698367 3.557609 -0.469978

H -7.864671 1.382038 0.096541

# Table S77. Geometry data of conformers of structure 3b

3b-c1 , delta G = 0.0000 kcal/mol, population = 28.89 %

C 3.815790 -1.670651 0.948930

C 2.499250 -2.233238 0.405647

C 2.129494 -1.894727 -1.029379

C 2.567945 -0.455508 -1.427120

C 3.233783 0.399760 -0.427905

C 3.310016 1.813113 -0.622451

C 4.021550 2.589186 0.299811

C 4.614163 1.990792 1.432193

C 4.495528 0.633591 1.629199

C 3.813948 -0.163351 0.706558

C 5.054285 -2.301483 0.286289

C 2.732463 -2.913485 -2.000629

C 2.543582 2.532277 -1.640692

O 2.673277 3.743882 -1.835477

C -0.096340 -1.171221 -0.543162

C -1.499302 -1.483346 -0.770261

C -2.474041 -0.751722 -0.192485

C -3.876235 -0.990158 -0.352476

C -4.820604 -0.235372 0.253963

C -6.238777 -0.440380 0.123542

C -7.156023 0.319894 0.740186

C -8.638283 0.144074 0.647367

C -9.312266 1.411288 0.086544

C -8.883083 1.759837 -1.336216

C -9.217053 -0.209584 2.026666

O 0.712832 -2.035989 -1.218682

O 0.355694 -0.269409 0.136127

O 1.787604 -2.903569 1.117102

O 3.883615 -1.959117 2.332345

O 2.341143 -0.105063 -2.569692

O 4.161837 3.921594 0.208921

O 5.282990 2.757827 2.319017

H -10.298591 -0.346861 1.962125

H -9.100758 0.937220 -2.022377

H -7.811189 1.959487 -1.391139

H -10.395318 1.261402 0.118317

H -9.097115 2.249792 0.757590

H -8.845393 -0.683879 -0.039040

H -6.815616 1.138740 1.372937

H -9.408944 2.645199 -1.698587

H -8.776680 -1.128803 2.415985

H -9.021114 0.590331 2.745344

H -6.566216 -1.258835 -0.512541

H -4.500741 0.587726 0.888447

H -4.179879 -1.816843 -0.988347

H -2.178172 0.078022 0.443357

H -1.722764 -2.325117 -1.412171

H 4.934967 0.183571 2.507255

H 3.050284 -2.401749 2.564319

H 4.992374 -3.389385 0.313802

H 5.190757 -1.964410 -0.738881

H 5.922469 -1.985521 0.863937

H 2.383393 -3.910724 -1.737199

H 3.817824 -2.897908 -1.987502

H 2.389728 -2.657984 -3.001278

H 1.806232 1.971514 -2.215338

H 3.675922 4.205826 -0.612791

H 5.243861 3.680429 2.021760

3b-c4 , delta G = 0.2146 kcal/mol, population = 20.10 %

C 3.901199 -1.616985 1.004225

C 2.585707 -2.205696 0.485707

C 2.212614 -1.924411 -0.960643

C 2.639576 -0.497245 -1.411041

C 3.299635 0.398659 -0.443861

C 3.362650 1.804734 -0.688387

C 4.068816 2.619344 0.204309

C 4.668822 2.066611 1.355795

C 4.562869 0.716152 1.600870

C 3.886706 -0.119037 0.708551

C 5.142347 -2.261444 0.360421

C 2.823451 -2.973464 -1.894045

C 2.587416 2.480189 -1.729710

O 2.705523 3.685182 -1.967878

C -0.018838 -1.196746 -0.507448

C -1.419363 -1.530257 -0.718336

C -2.399212 -0.768390 -0.190387

C -3.800072 -1.020642 -0.340164

C -4.748032 -0.223186 0.202854

C -6.165681 -0.435798 0.078230

C -7.085792 0.378974 0.616698

C -8.567800 0.207671 0.506011

C -9.167911 1.374488 -0.307752

C -10.664862 1.238923 -0.581870

C -9.191675 0.096224 1.905675

O 0.797117 -2.084725 -1.143301

O 0.426159 -0.260664 0.128877

O 1.876653 -2.848100 1.224948

O 3.975729 -1.856552 2.396484

O 2.410465 -0.190648 -2.565631

O 4.197070 3.948920 0.065834

O 5.332246 2.870581 2.213549

H -10.263591 -0.094249 1.846347

H -11.253563 1.283649 0.335459

H -10.887866 0.290203 -1.077002

H -8.971338 2.312124 0.223034

H -8.632081 1.438980 -1.258465

H -8.767026 -0.721037 -0.039483

H -6.747904 1.248297 1.179120

H -11.010649 2.043751 -1.233422

H -8.736158 -0.719139 2.469744

H -9.043205 1.021884 2.468303

H -6.489976 -1.305980 -0.487046

H -4.431294 0.644824 0.776075

H -4.099580 -1.891053 -0.916840

H -2.108637 0.101231 0.392414

H -1.636611 -2.410022 -1.309414

H 5.007911 0.301641 2.493482

H 3.141510 -2.285937 2.649201

H 5.088305 -3.348214 0.425011

H 5.273909 -1.958172 -0.675877

H 6.009742 -1.920149 0.924697

H 2.480040 -3.962786 -1.595662

H 3.908591 -2.951121 -1.880235

H 2.480578 -2.756220 -2.903614

H 1.854102 1.892545 -2.282186

H 3.707779 4.199330 -0.764774

H 5.283906 3.781656 1.883874

3b-c3 , delta G = 0.6896 kcal/mol, population = 9.01 %

C 3.192386 2.253162 0.123262

C 1.685025 1.984017 0.125818

C 1.165699 0.907033 1.063119

C 2.150159 -0.292704 1.184559

C 3.408977 -0.275527 0.417070

C 4.148868 -1.481220 0.214940

C 5.383389 -1.419538 -0.442104

C 5.867134 -0.192147 -0.943286

C 5.123240 0.954085 -0.778732

C 3.902629 0.920118 -0.100205

C 3.689503 2.893802 1.432232

C 0.871955 1.485789 2.450126

C 3.618929 -2.814567 0.503058

O 4.297811 -3.838052 0.383627

C -0.147803 -0.239141 -0.570466

C -1.488109 -0.620425 -0.984066

C -2.613247 -0.307038 -0.308117

C -3.931748 -0.686652 -0.718327

C -5.040944 -0.354041 -0.019870

C -6.381419 -0.717230 -0.396298

C -7.469513 -0.368705 0.306566

C -8.879960 -0.725505 -0.040042

C -9.724827 0.538441 -0.294183

C -9.250774 1.367644 -1.484712

C -9.502413 -1.575834 1.078254

O -0.112604 0.435551 0.612547

O 0.868342 -0.495077 -1.189334

O 0.957744 2.604076 -0.614929

O 3.478525 3.159416 -0.925001

O 1.827448 -1.197230 1.931149

O 6.162826 -2.487386 -0.677064

O 7.051273 -0.156092 -1.590060

H -10.528682 -1.848828 0.823505

H -9.255549 0.772205 -2.401386

H -8.233470 1.734433 -1.335407

H -10.761070 0.226056 -0.452591

H -9.722834 1.152018 0.613159

H -8.869121 -1.317269 -0.961628

H -7.339542 0.223237 1.211790

H -9.899426 2.231196 -1.643605

H -8.933456 -2.492687 1.240153

H -9.525246 -1.020751 2.019657

H -6.499955 -1.304320 -1.303436

H -4.927670 0.228922 0.891061

H -4.029636 -1.267987 -1.630692

H -2.526750 0.269405 0.606974

H -1.525539 -1.188717 -1.904727

H 5.489347 1.887279 -1.180999

H 2.638596 3.325608 -1.384435

H 3.694713 2.189304 2.260953

H 4.714904 3.219554 1.260040

H 3.084732 3.763776 1.688710

H 0.154936 2.299910 2.355237

H 1.768698 1.852960 2.939092

H 0.443970 0.688907 3.054950

H 2.572320 -2.895502 0.796682

H 5.688686 -3.277734 -0.299414

H 7.413319 -1.055520 -1.622716

3b-c7 , delta G = 0.8704 kcal/mol, population = 6.64 %

C -3.683305 -0.937793 -1.581437

C -3.815453 -2.078200 -0.546897

C -2.789642 -2.039255 0.597868

C -3.158501 -0.712509 1.308890

C -2.987905 0.481089 0.448027

C -2.595643 1.727844 1.003957

C -2.456300 2.835127 0.160395

C -2.690694 2.704699 -1.224005

C -3.068293 1.486442 -1.743049

C -3.220424 0.362177 -0.920477

C -5.001984 -0.758237 -2.317815

C -2.833632 -3.242689 1.504946

C -2.207422 1.877034 2.404476

O -1.882473 2.963614 2.888147

C -0.459616 -1.386358 0.622815

C 0.763470 -1.426044 -0.158175

C 1.903036 -0.875766 0.313750

C 3.149572 -0.857013 -0.385201

C 4.263462 -0.283595 0.127074

C 5.537178 -0.235306 -0.537993

C 6.625237 0.341490 -0.004910

C 7.970274 0.427629 -0.651726

C 9.033582 -0.311306 0.185212

C 8.787592 -1.812679 0.307624

C 8.367389 1.898362 -0.853948

O -1.506414 -1.934048 -0.076577

O -0.609686 -0.930929 1.738034

O -4.676639 -2.917377 -0.597079

O -2.724136 -1.372600 -2.562888

O -3.650028 -0.703521 2.415566

O -2.076553 4.051854 0.582579

O -2.536995 3.776690 -2.034036

H 9.343808 1.965546 -1.338194

H 9.581099 -2.293905 0.882438

H 8.755527 -2.284307 -0.677973

H 10.009138 -0.134398 -0.276632

H 9.076468 0.143291 1.180631

H 7.913729 -0.056073 -1.632621

H 6.544653 0.794427 0.982465

H 7.838456 -2.022578 0.804524

H 7.639315 2.422130 -1.475261

H 8.430125 2.417923 0.105556

H 5.604303 -0.694276 -1.520878

H 4.205907 0.177671 1.110004

H 3.189750 -1.322174 -1.365764

H 1.873745 -0.409350 1.294483

H 0.725740 -1.906690 -1.126814

H -3.231974 1.411035 -2.808904

H -1.889873 -1.529379 -2.098208

H -5.310241 -1.707166 -2.751183

H -5.777359 -0.413961 -1.634086

H -4.884556 -0.026752 -3.114705

H -3.829076 -3.351219 1.928835

H -2.119945 -3.118981 2.316856

H -2.594720 -4.139088 0.934921

H -2.187669 0.979722 3.025939

H -1.928107 3.981885 1.563582

H -2.266735 4.536346 -1.495404

3b-c14 , delta G = 0.9381 kcal/mol, population = 5.92 %

C 3.702956 -1.525158 1.314363

C 2.391221 -2.129255 0.804323

C 2.114337 -2.048222 -0.687717

C 2.620878 -0.712207 -1.305889

C 3.270621 0.285232 -0.435467

C 3.409620 1.639485 -0.868615

C 4.111241 2.543044 -0.062078

C 4.629997 2.135242 1.185397

C 4.447993 0.838845 1.610459

C 3.775865 -0.086516 0.808211

C 4.947005 -2.305286 0.852096

C 2.738049 -3.236686 -1.424951

C 2.712340 2.192166 -2.030283

O 2.896277 3.345963 -2.427086

C -0.108999 -1.193351 -0.492288

C -1.506976 -1.512865 -0.738242

C -2.484502 -0.650481 -0.391628

C -3.884105 -0.878894 -0.584284

C -4.826194 0.020765 -0.219920

C -6.243747 -0.164147 -0.386338

C -7.146428 0.757393 -0.013210

C -8.637827 0.654715 -0.122803

C -9.303893 0.763156 1.269719

C -9.038680 2.072280 2.010075

C -9.123184 -0.595228 -0.855162

O 0.707039 -2.191878 -0.934424

O 0.334976 -0.184720 0.022462

O 1.609614 -2.624018 1.582957

O 3.685869 -1.561479 2.728829

O 2.463579 -0.561591 -2.502472

O 4.307541 3.833726 -0.376664

O 5.289722 3.025141 1.956893

H -10.209517 -0.573524 -0.954313

H -9.313937 2.933128 1.394996

H -7.989182 2.181738 2.288121

H -10.381643 0.644699 1.126795

H -8.975389 -0.084177 1.879553

H -8.969066 1.534385 -0.692440

H -6.770976 1.678394 0.424445

H -9.625543 2.119718 2.929256

H -8.695740 -0.667162 -1.856797

H -8.858171 -1.502692 -0.307139

H -6.566883 -1.097740 -0.834580

H -4.503538 0.954528 0.234565

H -4.187549 -1.816923 -1.040291

H -2.192648 0.288233 0.070977

H -1.724068 -2.465690 -1.202553

H 4.829226 0.537356 2.575050

H 2.817259 -1.912814 2.986357

H 4.835463 -3.368292 1.066274

H 5.157414 -2.161630 -0.205301

H 5.794150 -1.921012 1.419510

H 2.346059 -4.163475 -1.008679

H 3.820972 -3.240914 -1.352791

H 2.456598 -3.158375 -2.473131

H 1.976059 1.562623 -2.530385

H 3.868784 3.984549 -1.257859

H 5.299397 3.881770 1.501795

3b-c15 , delta G = 1.0329 kcal/mol, population = 5.04 %

C 3.860052 -1.581552 1.048566

C 2.559850 -2.197572 0.523254

C 2.200613 -1.943240 -0.931499

C 2.609083 -0.515227 -1.396482

C 3.243507 0.404232 -0.434113

C 3.287504 1.807593 -0.697550

C 3.971226 2.645364 0.191183

C 4.566857 2.118013 1.356798

C 4.479153 0.769587 1.619468

C 3.825760 -0.088302 0.731646

C 5.118340 -2.215592 0.428336

C 2.840449 -2.994772 -1.842628

C 2.513308 2.456483 -1.756369

O 2.615671 3.659719 -2.010187

C -0.048863 -1.247192 -0.518193

C -1.440319 -1.608787 -0.742924

C -2.441541 -0.859527 -0.237720

C -3.834788 -1.143138 -0.402422

C -4.807452 -0.362754 0.121195

C -6.217831 -0.612461 -0.017146

C -7.165140 0.181055 0.506171

C -8.639911 -0.043431 0.382195

C -9.305555 1.019885 -0.524305

C -9.196184 2.464712 -0.037790

C -9.294985 -0.107844 1.770591

O 0.790548 -2.130342 -1.129653

O 0.371185 -0.293876 0.109329

O 1.851078 -2.838999 1.263576

O 3.922027 -1.800597 2.444831

O 2.388215 -0.227932 -2.557583

O 4.080461 3.974703 0.035436

O 5.208101 2.944066 2.210430

H -10.374263 -0.247674 1.677347

H -9.705229 3.137433 -0.730519

H -8.156493 2.791143 0.029622

H -8.866129 0.940862 -1.522318

H -10.361488 0.751465 -0.626734

H -8.790348 -1.010363 -0.107615

H -6.852217 1.057952 1.068581

H -9.653733 2.598676 0.943982

H -8.891183 -0.939064 2.350732

H -9.121627 0.808520 2.337917

H -6.512615 -1.495746 -0.578460

H -4.518657 0.518084 0.689634

H -4.107659 -2.026012 -0.973343

H -2.176380 0.022906 0.337933

H -1.633186 -2.500273 -1.324928

H 4.920603 0.374474 2.522606

H 3.090938 -2.237837 2.694336

H 5.080067 -3.301979 0.508196

H 5.257113 -1.925299 -0.610747

H 5.973901 -1.852991 0.997367

H 2.510710 -3.985599 -1.533928

H 3.924776 -2.953555 -1.817244

H 2.505355 -2.797802 -2.858947

H 1.794926 1.850018 -2.308172

H 3.596773 4.205881 -0.803954

H 5.148939 3.849661 1.867670

3b-c26 , delta G = 1.1107 kcal/mol, population = 4.42 %

C 3.295387 2.248946 0.026252

C 1.784164 2.003970 0.028696

C 1.241341 0.972195 1.002810

C 2.205077 -0.237602 1.178726

C 3.472044 -0.268851 0.425186

C 4.197254 -1.491784 0.280192

C 5.439101 -1.473770 -0.365680

C 5.944311 -0.274760 -0.912466

C 5.214667 0.887089 -0.802819

C 3.987438 0.897668 -0.135663

C 3.792800 2.936244 1.311335

C 0.947784 1.608238 2.364516

C 3.647177 -2.805035 0.618640

O 4.314199 -3.840969 0.549873

C -0.078180 -0.212697 -0.596994

C -1.421102 -0.586716 -1.008572

C -2.546105 -0.237253 -0.350313

C -3.867336 -0.608904 -0.759077

C -4.975738 -0.243516 -0.075940

C -6.319301 -0.598323 -0.449930

C -7.405127 -0.226420 0.244852

C -8.818260 -0.571682 -0.104065

C -9.616267 0.715695 -0.398021

C -11.043202 0.468106 -0.884798

C -9.450402 -1.398977 1.026752

O -0.041198 0.504338 0.560857

O 0.938635 -0.507127 -1.197377

O 1.071393 2.607187 -0.739550

O 3.603263 3.106413 -1.056450

O 1.860132 -1.107976 1.955397

O 6.206351 -2.560562 -0.547400

O 7.135132 -0.281115 -1.547725

H -9.503562 -0.814105 1.949057

H -11.049122 -0.170265 -1.772110

H -11.527868 1.409830 -1.149507

H -9.635276 1.332819 0.506762

H -9.073090 1.290607 -1.152870

H -8.808743 -1.179802 -1.015033

H -7.270624 0.377385 1.141267

H -11.656778 -0.014007 -0.122250

H -10.461991 -1.712829 0.767758

H -8.861295 -2.295056 1.227774

H -6.441462 -1.202241 -1.345636

H -4.859310 0.359780 0.821263

H -3.967865 -1.211718 -1.657105

H -2.457434 0.363992 0.548486

H -1.460503 -1.182038 -1.911930

H 5.597204 1.798087 -1.239227

H 2.768808 3.268920 -1.527014

H 3.200557 3.826058 1.525284

H 3.779745 2.267849 2.169311

H 4.824776 3.237514 1.134059

H 0.247129 2.431462 2.233321

H 1.847807 1.976560 2.846555

H 0.500552 0.842649 2.995207

H 2.596724 -2.860630 0.904223

H 5.717737 -3.327762 -0.141204

H 7.485469 -1.185733 -1.539160

3b-c13 , delta G = 1.1383 kcal/mol, population = 4.22 %

C 3.726019 -1.628225 1.226096

C 2.405685 -2.172076 0.673179

C 2.134993 -1.986513 -0.810971

C 2.672186 -0.625391 -1.341180

C 3.346075 0.298282 -0.409790

C 3.522906 1.673040 -0.757309

C 4.244843 2.505639 0.105766

C 4.747508 2.007188 1.326681

C 4.529938 0.692089 1.669133

C 3.837376 -0.163799 0.809106

C 4.954242 -2.409784 0.724741

C 2.734176 -3.137612 -1.624143

C 2.847787 2.315586 -1.885472

O 3.063993 3.486958 -2.207904

C -0.069399 -1.102154 -0.551628

C -1.473715 -1.373390 -0.818050

C -2.434082 -0.524316 -0.398183

C -3.837754 -0.712716 -0.603822

C -4.765758 0.164960 -0.158686

C -6.185914 0.012553 -0.336200

C -7.078495 0.903394 0.124071

C -8.572153 0.830330 0.019928

C -9.178029 0.870991 1.442176

C -10.694988 1.047054 1.471154

C -9.081710 -0.355513 -0.797172

O 0.725528 -2.082036 -1.068044

O 0.395938 -0.144618 0.036545

O 1.613554 -2.706020 1.414405

O 3.698137 -1.748436 2.635909

O 2.516988 -0.394272 -2.525201

O 4.476611 3.807554 -0.127285

O 5.426882 2.829638 2.153816

H -8.837641 -1.302837 -0.309668

H -10.995277 1.942865 0.921178

H -11.050297 1.151628 2.498109

H -8.897719 -0.048951 1.965080

H -8.712542 1.692956 1.993582

H -8.894972 1.754190 -0.480891

H -6.698963 1.774730 0.654343

H -11.213559 0.194368 1.030507

H -10.164170 -0.309485 -0.912269

H -8.644720 -0.365602 -1.797142

H -6.520242 -0.871844 -0.868083

H -4.429526 1.050978 0.374645

H -4.156683 -1.603220 -1.137853

H -2.124092 0.367757 0.138938

H -1.710365 -2.281326 -1.356884

H 4.898896 0.321343 2.614152

H 2.823800 -2.104215 2.866350

H 4.817176 -3.479938 0.880443

H 5.172019 -2.213850 -0.322756

H 5.807948 -2.077156 1.314546

H 2.322862 -4.081185 -1.268726

H 3.816808 -3.168557 -1.553239

H 2.455113 -2.985464 -2.664803

H 2.099444 1.738300 -2.428651

H 4.044853 4.024205 -0.998434

H 5.460354 3.712441 1.753055

3b-c47 , delta G = 1.1446 kcal/mol, population = 4.18 %

C -3.300225 -2.211773 -0.063381

C -1.781812 -2.019057 -0.096340

C -1.172623 -1.061027 0.913371

C -2.085042 0.171020 1.182872

C -3.373928 0.285456 0.475720

C -4.059570 1.538280 0.419169

C -5.322198 1.596659 -0.182477

C -5.888103 0.446043 -0.772130

C -5.196917 -0.743716 -0.748447

C -3.948802 -0.831337 -0.126993

C -3.781974 -2.955998 1.195583

C -0.864944 -1.779144 2.230423

C -3.452285 2.812524 0.805183

O -4.083904 3.872790 0.812070

C 0.142702 0.151482 -0.668007

C 1.485841 0.481289 -1.114662

C 2.616787 0.048259 -0.519157

C 3.937732 0.371286 -0.967952

C 5.053024 -0.073544 -0.345768

C 6.396416 0.228787 -0.765193

C 7.483341 -0.221037 -0.118021

C 8.915748 0.052123 -0.466168

C 9.629477 0.810886 0.677978

C 9.655374 0.079864 2.018692

C 9.104991 0.784079 -1.794113

O 0.113206 -0.617949 0.456128

O -0.880092 0.520700 -1.214577

O -1.114823 -2.603364 -0.918459

O -3.671164 -2.994907 -1.182275

O -1.684472 0.986975 1.991228

O -6.055444 2.717027 -0.280606

O -7.098689 0.526052 -1.363542

H 8.639889 0.244787 -2.621116

H 10.083173 -0.920628 1.912479

H 8.657219 -0.026900 2.446636

H 10.656293 1.001962 0.353672

H 9.152529 1.788908 0.795640

H 9.409174 -0.926819 -0.542660

H 7.324900 -0.843061 0.758805

H 10.263605 0.625477 2.742530

H 8.673996 1.787511 -1.758204

H 10.168202 0.888550 -2.015765

H 6.503479 0.849792 -1.648289

H 4.942387 -0.703164 0.534065

H 4.032410 1.002253 -1.847096

H 2.533815 -0.586903 0.356606

H 1.519923 1.113864 -1.992626

H -5.625873 -1.617024 -1.217523

H -2.858210 -3.155459 -1.689612

H -3.720832 -2.340078 2.089937

H -4.828332 -3.213247 1.033838

H -3.213606 -3.875076 1.338967

H -1.764753 -2.137487 2.720410

H -0.369319 -1.065874 2.885842

H -0.201951 -2.620897 2.035703

H -2.391768 2.817433 1.057228

H -5.525391 3.444673 0.146380

H -7.415948 1.440389 -1.296674

3b-c53 , delta G = 1.4590 kcal/mol, population = 2.45 %

C -3.288163 -2.209994 -0.156620

C -1.769236 -2.030979 -0.092167

C -1.214874 -1.102615 0.975095

C -2.132123 0.131185 1.218874

C -3.374646 0.274649 0.437894

C -4.044272 1.535517 0.367636

C -5.265380 1.620844 -0.311596

C -5.804194 0.489344 -0.960574

C -5.126920 -0.707963 -0.920438

C -3.920932 -0.822442 -0.224924

C -3.850309 -2.981762 1.051560

C -0.992508 -1.853808 2.290888

C -3.451877 2.794165 0.822171

O -4.073132 3.860538 0.813055

C 0.202916 0.136314 -0.493645

C 1.572940 0.469157 -0.846282

C 2.662373 0.012675 -0.193586

C 4.010036 0.341994 -0.548303

C 5.083207 -0.132494 0.124197

C 6.450281 0.177305 -0.200977

C 7.496038 -0.310990 0.483810

C 8.953664 -0.092621 0.196593

C 9.266873 1.080043 -0.740074

C 8.962846 2.455104 -0.146371

C 9.558234 -1.405849 -0.337933

O 0.099506 -0.660533 0.606590

O -0.781799 0.525392 -1.093803

O -1.057847 -2.602415 -0.885547

O -3.596408 -2.960946 -1.315865

O -1.774424 0.923773 2.069445

O -5.980153 2.750999 -0.430881

O -6.974316 0.595017 -1.624883

H 10.641894 -1.314360 -0.434857

H 9.227996 3.249963 -0.846262

H 9.535903 2.614283 0.770579

H 10.329800 1.029547 -0.991526

H 8.731836 0.947240 -1.685622

H 9.438054 0.112932 1.159938

H 7.288573 -0.977137 1.318887

H 7.906724 2.567267 0.102005

H 9.345752 -2.243532 0.328946

H 9.143116 -1.642569 -1.320070

H 6.610989 0.841181 -1.043976

H 4.917043 -0.793659 0.971439

H 4.161370 1.002692 -1.397200

H 2.521608 -0.647266 0.656099

H 1.665080 1.125368 -1.702399

H -5.533245 -1.565906 -1.435614

H -2.754801 -3.120545 -1.774468

H -3.298281 -3.909228 1.204554

H -3.838140 -2.389574 1.963680

H -4.886974 -3.225696 0.821283

H -0.327495 -2.698282 2.116238

H -1.923046 -2.212702 2.719059

H -0.529420 -1.161108 2.990678

H -2.410173 2.782263 1.142816

H -5.472065 3.462847 0.046426

H -7.287343 1.510713 -1.556910

3b-c42 , delta G = 1.4596 kcal/mol, population = 2.45 %

C 3.195976 2.266881 -0.003754

C 1.691283 1.983425 -0.012033

C 1.163903 0.960286 0.979551

C 2.156754 -0.219853 1.192163

C 3.428982 -0.237061 0.446877

C 4.182038 -1.445767 0.330148

C 5.427308 -1.412512 -0.308415

C 5.909605 -0.213025 -0.874360

C 5.153167 0.933966 -0.792415

C 3.921344 0.929611 -0.133747

C 3.663977 2.989809 1.272625

C 0.838771 1.618957 2.323177

C 3.658054 -2.763906 0.689866

O 4.347563 -3.786279 0.645008

C -0.107125 -0.300985 -0.601733

C -1.435302 -0.718976 -1.018873

C -2.578352 -0.372350 -0.391016

C -3.882964 -0.789703 -0.809115

C -5.013854 -0.421239 -0.165706

C -6.339947 -0.820232 -0.557126

C -7.452906 -0.431136 0.083629

C -8.845891 -0.818578 -0.304284

C -9.628395 0.380572 -0.892487

C -9.836418 1.562603 0.054106

C -9.581259 -1.460302 0.881923

O -0.101663 0.449857 0.534865

O 0.923730 -0.589566 -1.180654

O 0.972421 2.549167 -0.802795

O 3.493635 3.111416 -1.099237

O 1.829207 -1.079438 1.988029

O 6.219246 -2.485509 -0.464798

O 7.104897 -0.204578 -1.501323

H -10.605620 -1.716917 0.602870

H -10.388968 1.273655 0.949912

H -10.406941 2.349818 -0.442296

H -9.103693 0.723395 -1.788231

H -10.602060 0.007734 -1.225005

H -8.774357 -1.564911 -1.101399

H -7.351532 0.216161 0.951967

H -8.887091 1.998329 0.371902

H -9.075868 -2.372647 1.202591

H -9.624486 -0.785803 1.739067

H -6.424904 -1.471202 -1.423868

H -4.931585 0.221480 0.707557

H -3.951512 -1.430031 -1.683913

H -2.518948 0.262557 0.486803

H -1.447569 -1.345387 -1.901774

H 5.518150 1.844777 -1.244003

H 2.661316 3.240475 -1.583746

H 4.689742 3.313562 1.098894

H 3.047694 3.868233 1.464558

H 3.659695 2.337083 2.142698

H 1.722692 2.024896 2.804924

H 0.407342 0.855640 2.967514

H 0.115953 2.418149 2.165943

H 2.607002 -2.836609 0.969631

H 5.745107 -3.255307 -0.046793

H 7.475650 -1.100593 -1.472566

3b-c48 , delta G = 1.4709 kcal/mol, population = 2.41 %

C -3.287677 -2.210341 -0.156400

C -1.768827 -2.030312 -0.092472

C -1.214599 -1.101963 0.974868

C -2.132172 0.131700 1.218375

C -3.374860 0.274456 0.437561

C -4.044855 1.535095 0.367181

C -5.266276 1.619851 -0.311458

C -5.805051 0.487998 -0.959853

C -5.127358 -0.709076 -0.919754

C -3.921012 -0.822984 -0.224720

C -3.849437 -2.982286 1.051778

C -0.992338 -1.852941 2.290790

C -3.452394 2.794087 0.820736

O -4.073942 3.860249 0.811765

C 0.203150 0.137870 -0.493143

C 1.573198 0.470653 -0.845745

C 2.662583 0.013676 -0.193310

C 4.010276 0.342954 -0.547955

C 5.083395 -0.132113 0.124261

C 6.450515 0.177510 -0.200874

C 7.496165 -0.311435 0.483636

C 8.953855 -0.093418 0.196510

C 9.267393 1.079223 -0.740055

C 8.964066 2.454322 -0.146079

C 9.558077 -1.406780 -0.338044

O 0.099742 -0.659831 0.606459

O -0.781577 0.527662 -1.092859

O -1.057400 -2.600709 -0.886601

O -3.595821 -2.961549 -1.315524

O -1.774714 0.924661 2.068700

O -5.981450 2.749793 -0.430782

O -6.975523 0.593095 -1.623621

H 9.345305 -2.244472 0.328740

H 7.908050 2.566871 0.102597

H 9.229344 3.249204 -0.845906

H 10.330266 1.028317 -0.991697

H 8.732125 0.946758 -1.685529

H 9.438237 0.111984 1.159921

H 7.288497 -0.977921 1.318393

H 9.537440 2.613099 0.770746

H 9.142951 -1.643303 -1.320237

H 10.641778 -1.315628 -0.434905

H 6.611343 0.841747 -1.043559

H 4.917118 -0.793657 0.971175

H 4.161700 1.004032 -1.396532

H 2.521781 -0.646655 0.656061

H 1.665355 1.127280 -1.701538

H -5.533605 -1.567309 -1.434512

H -2.754440 -3.119410 -1.775156

H -4.885911 -3.226929 0.821350

H -3.296867 -3.909380 1.205082

H -3.837877 -2.389901 1.963781

H -1.922896 -2.211750 2.718969

H -0.529270 -1.160129 2.990494

H -0.327315 -2.697474 2.116370

H -2.410371 2.782506 1.140443

H -5.473410 3.461943 0.046046

H -7.288754 1.508740 -1.555889

3b-c12 , delta G = 1.4853 kcal/mol, population = 2.35 %

C 3.651493 -1.706140 1.140546

C 2.321610 -2.212114 0.574831

C 2.042238 -1.959601 -0.897623

C 2.590695 -0.582834 -1.373224

C 3.282161 0.292722 -0.408864

C 3.472152 1.679339 -0.698125

C 4.205239 2.466993 0.197315

C 4.707624 1.911776 1.393585

C 4.479631 0.585001 1.679385

C 3.775234 -0.226090 0.786353

C 4.867383 -2.477929 0.595855

C 2.622546 -3.081065 -1.764343

C 2.802467 2.376637 -1.796632

O 3.029892 3.558853 -2.068130

C -0.149200 -1.059424 -0.589098

C -1.557695 -1.295769 -0.866126

C -2.502298 -0.438778 -0.426818

C -3.907849 -0.589057 -0.649681

C -4.819759 0.302137 -0.198041

C -6.240165 0.190074 -0.400309

C -7.117369 1.097002 0.057277

C -8.608536 1.074128 -0.091212

C -9.285347 1.203547 1.295341

C -9.042646 0.022852 2.233911

C -9.145173 -0.109353 -0.895325

O 0.629898 -2.028503 -1.147927

O 0.331727 -0.134816 0.038026

O 1.529380 -2.769843 1.298197

O 3.632591 -1.887367 2.543943

O 2.428552 -0.299746 -2.544972

O 4.449352 3.775265 0.019322

O 5.397843 2.691286 2.252622

H -8.744330 -0.112288 -1.910383

H -7.975306 -0.137657 2.400614

H -9.459277 -0.903276 1.834060

H -8.928207 2.122693 1.768944

H -10.359417 1.334299 1.136813

H -8.872980 1.991646 -0.634438

H -6.725673 1.949865 0.608832

H -9.509748 0.201210 3.204293

H -8.890156 -1.063976 -0.432455

H -10.232927 -0.052698 -0.964732

H -6.589076 -0.676645 -0.951833

H -4.469165 1.168382 0.358076

H -4.241895 -1.459048 -1.207693

H -2.176437 0.430746 0.137199

H -1.810309 -2.181014 -1.434659

H 4.848899 0.169684 2.605564

H 2.757393 -2.247094 2.764910

H 4.719365 -3.552446 0.704410

H 5.080011 -2.237717 -0.443426

H 5.728866 -2.181513 1.193593

H 2.205275 -4.034983 -1.445417

H 3.705467 -3.125168 -1.705551

H 2.335349 -2.882535 -2.794937

H 2.049109 1.830749 -2.364632

H 4.016896 4.032966 -0.840420

H 5.437774 3.590473 1.890732

3b-c21 , delta G = 1.6008 kcal/mol, population = 1.93 %

C -3.342576 -2.194901 0.049277

C -1.819565 -2.040804 0.010440

C -1.190917 -1.049140 0.974294

C -2.076028 0.215479 1.177899

C -3.354977 0.327582 0.452446

C -4.006346 1.593145 0.324216

C -5.259544 1.653506 -0.296856

C -5.848036 0.489735 -0.836472

C -5.189848 -0.715325 -0.742225

C -3.953049 -0.803302 -0.099174

C -3.847764 -2.846820 1.349375

C -0.903842 -1.710200 2.325527

C -3.369717 2.869048 0.652965

O -3.972394 3.944570 0.596792

C 0.164039 0.055495 -0.654696

C 1.519246 0.331799 -1.101557

C 2.632244 -0.103962 -0.474906

C 3.966791 0.164781 -0.919016

C 5.060706 -0.286711 -0.264415

C 6.418215 -0.039731 -0.673797

C 7.481523 -0.500483 0.003114

C 8.928564 -0.297084 -0.330018

C 9.676011 0.306361 0.884242

C 9.236648 1.719674 1.262535

C 9.175245 0.488264 -1.617723

O 0.106975 -0.658958 0.504089

O -0.844625 0.422720 -1.228001

O -1.165521 -2.679578 -0.780899

O -3.730571 -3.034403 -1.021345

O -1.662502 1.059187 1.950394

O -5.961460 2.786125 -0.460978

O -7.047852 0.571626 -1.449191

H 10.247370 0.589436 -1.795467

H 8.166984 1.756819 1.479703

H 9.437617 2.432550 0.461011

H 9.535358 -0.358827 1.741338

H 10.746394 0.299830 0.660406

H 9.352499 -1.301163 -0.467152

H 7.297834 -1.082453 0.904643

H 9.770908 2.063551 2.150149

H 8.738934 -0.019078 -2.479868

H 8.749271 1.491732 -1.569922

H 6.558319 0.549008 -1.574449

H 4.919807 -0.878654 0.636962

H 4.090204 0.757814 -1.820713

H 2.522286 -0.699242 0.425592

H 1.578327 0.924760 -2.005409

H -5.635827 -1.600380 -1.171703

H -2.922116 -3.239800 -1.519716

H -3.306884 -3.771594 1.551297

H -3.769683 -2.178172 2.203824

H -4.901021 -3.082994 1.200708

H -0.253157 -2.570058 2.173122

H -1.811756 -2.032250 2.825203

H -0.400314 -0.975589 2.950629

H -2.312787 2.858942 0.919521

H -5.416725 3.520591 -0.065270

H -7.340369 1.496489 -1.434173

# **Table S78.** Geometry data of conformers of structure **3b'**

3b'-c1 , delta G = 0.0000 kcal/mol, population = 26.08 %

C -3.744494 -1.710768 1.015398

C -2.429309 -2.242084 0.438471

C -2.113387 -1.910022 -1.010548

C -2.600843 -0.486168 -1.408387

C -3.260139 0.361015 -0.397578

C -3.379407 1.770346 -0.601202

C -4.080265 2.536237 0.337949

C -4.619645 1.933351 1.494296

C -4.460499 0.581414 1.697846

C -3.789242 -0.206116 0.759662

C -4.984987 -2.380625 0.396767

C -2.719825 -2.953840 -1.952805

C -2.667570 2.500097 -1.651003

C 0.106386 -1.115570 -0.615906

C 1.508406 -1.379742 -0.902277

C 2.477563 -0.584859 -0.404165

C 3.878501 -0.762033 -0.637445

C 4.815568 0.069941 -0.128407

C 6.231659 -0.065650 -0.343789

C 7.142410 0.777530 0.165412

C 8.621021 0.686853 -0.039258

C 9.357287 0.510847 1.303457

C 9.004069 -0.781458 2.034983

C 9.131789 1.935138 -0.775843

O -0.700219 -2.017196 -1.244166

O -0.347308 -0.219597 0.069999

O -1.677000 -2.882484 1.135432

O -3.763615 -1.988050 2.402631

O -2.416400 -0.141793 -2.560310

O -4.258110 3.863608 0.240980

O -5.278020 2.691370 2.396570

H 8.644151 2.048662 -1.745312

H 7.943819 -0.817028 2.292751

H 9.577747 -0.878189 2.958710

H 10.432687 0.539762 1.105534

H 9.137596 1.373618 1.941403

H 8.829803 -0.192056 -0.658578

H 6.798667 1.609546 0.778846

H 9.221641 -1.653528 1.412805

H 8.935822 2.837253 -0.190579

H 10.209508 1.867255 -0.939089

H 6.563499 -0.900919 -0.955197

H 4.490735 0.905842 0.486539

H 4.187024 -1.601015 -1.254556

H 2.177346 0.254272 0.217189

H 1.735279 -2.231435 -1.529805

H -4.859065 0.127786 2.593390

H -2.909929 -2.400874 2.614706

H -5.843367 -2.081884 0.997792

H -5.161716 -2.058033 -0.626865

H -4.893121 -3.466198 0.432763

H -2.340777 -3.939824 -1.688353

H -3.804444 -2.962604 -1.909934

H -2.410797 -2.702480 -2.965405

H -1.938303 1.953208 -2.248770

O -2.833967 3.706739 -1.849453

H -3.806298 4.152924 -0.598459

H -5.270678 3.612154 2.091249

3b'-c5 , delta G = 0.1424 kcal/mol, population = 20.50 %

C 3.269379 -2.223181 0.134332

C 1.754101 -2.006839 0.095390

C 1.171047 -0.954164 1.022743

C 2.107510 0.280570 1.172929

C 3.386299 0.311226 0.439069

C 4.087385 1.543316 0.258744

C 5.339014 1.528363 -0.368188

C 5.877707 0.321251 -0.862535

C 5.171993 -0.851589 -0.718635

C 3.935527 -0.863995 -0.068890

C 3.755230 -2.847821 1.454815

C 0.864169 -1.548094 2.400541

C 3.503395 2.855802 0.537867

C -0.143944 0.145159 -0.641674

C -1.488300 0.454931 -1.099713

C -2.617041 0.058960 -0.475095

C -3.939885 0.359262 -0.934214

C -5.052447 -0.063575 -0.292248

C -6.397179 0.214393 -0.722373

C -7.488656 -0.224147 -0.077641

C -8.903334 0.027074 -0.493209

C -9.681015 0.775348 0.606687

C -9.127656 2.163506 0.917716

C -9.597531 -1.300185 -0.837107

O -0.112317 -0.530930 0.540562

O 0.877068 0.457169 -1.225786

O 1.070308 -2.645234 -0.670801

O 3.616104 -3.116654 -0.906489

O 1.732800 1.169931 1.913285

O 6.085210 2.624355 -0.580052

O 7.076870 0.330236 -1.481994

H -9.622906 -1.960392 0.033732

H -9.731614 2.665718 1.675862

H -9.123715 2.791460 0.022925

H -10.723931 0.857850 0.287238

H -9.684231 0.162256 1.514393

H -8.892453 0.656223 -1.389662

H -7.358868 -0.821837 0.823872

H -8.101551 2.110002 1.286761

H -10.627195 -1.121060 -1.153947

H -9.076312 -1.821470 -1.641501

H -6.515804 0.812938 -1.622007

H -4.938733 -0.657185 0.611746

H -4.038494 0.951179 -1.839669

H -2.530564 -0.528311 0.433101

H -1.525142 1.034337 -2.013491

H 5.580207 -1.769864 -1.114635

H 2.796687 -3.307375 -1.392670

H 3.178195 -3.741532 1.692866

H 4.796925 -3.132749 1.310072

H 3.710270 -2.146198 2.284666

H 0.390114 -0.769991 2.995400

H 0.182025 -2.389124 2.285562

H 1.761550 -1.881308 2.911985

H 2.447898 2.898383 0.806802

O 4.148126 3.903220 0.437663

H 5.573151 3.395537 -0.212022

H 7.406541 1.242348 -1.504351

3b'-c4 , delta G = 0.2905 kcal/mol, population = 15.96 %

C -3.818867 -1.651157 1.100036

C -2.510984 -2.215627 0.537937

C -2.201884 -1.947384 -0.925922

C -2.680600 -0.537740 -1.380237

C -3.328464 0.355770 -0.402499

C -3.439234 1.755944 -0.664981

C -4.130884 2.565574 0.243656

C -4.669039 2.015705 1.426676

C -4.517918 0.672505 1.686508

C -3.855774 -0.158654 0.779916

C -5.067735 -2.339277 0.519635

C -2.822709 -3.024840 -1.819655

C -2.726942 2.435718 -1.747566

C 0.026114 -1.158120 -0.572677

C 1.425222 -1.454452 -0.840786

C 2.403993 -0.656418 -0.367026

C 3.803870 -0.868160 -0.576650

C 4.749838 -0.034777 -0.086627

C 6.166395 -0.205531 -0.272912

C 7.084311 0.642281 0.216035

C 8.564750 0.507899 0.049019

C 9.235394 0.343640 1.429131

C 10.738607 0.077685 1.364926

C 9.118244 1.716516 -0.722659

O -0.791100 -2.076198 -1.162400

O -0.417566 -0.226801 0.071509

O -1.758170 -2.830454 1.257131

O -3.830149 -1.867618 2.498233

O -2.500653 -0.243307 -2.546645

O -4.300489 3.888871 0.091081

O -5.318212 2.815750 2.298793

H 8.614987 1.826468 -1.684447

H 10.955123 -0.796223 0.744987

H 11.138063 -0.114622 2.362555

H 9.043071 1.243513 2.023172

H 8.745617 -0.482365 1.951862

H 8.763968 -0.397403 -0.534506

H 6.745711 1.507527 0.784271

H 11.285877 0.925449 0.949954

H 8.968245 2.638067 -0.153828

H 10.186400 1.607311 -0.912737

H 6.491638 -1.071350 -0.844284

H 4.432161 0.830685 0.489925

H 4.104082 -1.736830 -1.155579

H 2.113161 0.212302 0.217007

H 1.642172 -2.335454 -1.430176

H -4.915385 0.260346 2.602355

H -2.977784 -2.276799 2.722315

H -5.248097 -2.063018 -0.516834

H -4.983498 -3.422671 0.605251

H -5.920647 -2.006990 1.110748

H -2.448061 -4.001723 -1.517833

H -3.906957 -3.024607 -1.767824

H -2.520370 -2.817641 -2.844172

H -2.003293 1.859197 -2.323941

O -2.886643 3.633808 -1.996539

H -3.851275 4.139024 -0.762155

H -5.305267 3.722780 1.954948

3b'-c9 , delta G = 0.6670 kcal/mol, population = 8.45 %

C 3.345451 -2.213715 0.055300

C 1.829354 -2.003075 0.025742

C 1.243410 -0.983351 0.987340

C 2.174756 0.249505 1.178585

C 3.455154 0.308807 0.449430

C 4.154406 1.547957 0.314249

C 5.407366 1.557595 -0.310191

C 5.949139 0.369845 -0.846448

C 5.245394 -0.808605 -0.745541

C 4.007781 -0.846544 -0.098991

C 3.833625 -2.883681 1.352787

C 0.940391 -1.623556 2.345059

C 3.568939 2.848799 0.640826

C -0.076403 0.162487 -0.640839

C -1.421789 0.487833 -1.084772

C -2.548548 0.088224 -0.458867

C -3.873177 0.406436 -0.900456

C -4.982026 -0.012686 -0.249667

C -6.329932 0.284497 -0.656851

C -7.415542 -0.143819 0.005005

C -8.833538 0.143056 -0.375616

C -9.513546 0.975688 0.732004

C -10.935296 1.421257 0.393882

C -9.579960 -1.170988 -0.653816

O -0.041833 -0.548967 0.520548

O 0.943306 0.490040 -1.218674

O 1.146784 -2.621249 -0.757915

O 3.694868 -3.068282 -1.016934

O 1.794586 1.113062 1.946298

O 6.152274 2.661534 -0.480520

O 7.149525 0.402888 -1.462566

H -10.597336 -0.982013 -0.997562

H -11.617018 0.574536 0.301728

H -10.958398 1.973881 -0.548985

H -9.518493 0.392328 1.659111

H -8.896128 1.857166 0.924759

H -8.829559 0.741179 -1.293198

H -7.277140 -0.750287 0.899051

H -11.328702 2.076364 1.173560

H -9.068068 -1.752087 -1.422430

H -9.636727 -1.781687 0.251379

H -6.455603 0.889669 -1.551231

H -4.862454 -0.617669 0.646009

H -3.976068 1.011062 -1.796985

H -2.458929 -0.515234 0.438363

H -1.461129 1.085665 -1.986500

H 5.656011 -1.711612 -1.172918

H 2.875145 -3.247558 -1.506901

H 3.787226 -2.212298 2.207246

H 3.258877 -3.786714 1.558988

H 4.875948 -3.160475 1.197352

H 1.840234 -1.966877 2.845354

H 0.461338 -0.868193 2.964622

H 0.263934 -2.465047 2.203103

H 2.513176 2.880834 0.910027

O 4.213160 3.899679 0.580251

H 5.638310 3.417886 -0.084961

H 7.477742 1.315742 -1.451789

3b'-c22 , delta G = 0.9413 kcal/mol, population = 5.31 %

C 3.894306 -1.607326 -1.019379

C 2.567742 -2.196279 -0.529969

C 2.176081 -1.937778 0.915628

C 2.609428 -0.523839 1.400039

C 3.291681 0.384645 0.460570

C 3.365241 1.784933 0.734261

C 4.092460 2.609957 -0.131588

C 4.703008 2.074223 -1.285629

C 4.587162 0.730071 -1.559228

C 3.889947 -0.115876 -0.693691

C 5.120741 -2.276301 -0.371820

C 2.763194 -3.010749 1.837118

C 2.582535 2.447386 1.778268

C -0.041160 -1.182901 0.438103

C -1.447739 -1.499363 0.633453

C -2.413304 -0.730199 0.089780

C -3.818027 -0.966994 0.227099

C -4.753607 -0.170755 -0.338797

C -6.174125 -0.372458 -0.224861

C -7.075905 0.437671 -0.801467

C -8.569497 0.331766 -0.729015

C -9.140953 1.642627 -0.140453

C -10.660226 1.763143 -0.242718

C -9.070086 -0.906406 0.012924

O 0.756586 -2.086395 1.075754

O 0.422675 -0.248319 -0.186825

O 1.866839 -2.823761 -1.289448

O 3.985222 -1.819007 -2.415231

O 2.365075 -0.237430 2.556734

O 4.232609 3.934902 0.035947

O 5.386642 2.888312 -2.117544

H -10.155895 -0.977406 -0.042205

H -10.993484 2.741044 0.110066

H -11.171023 1.007535 0.355873

H -8.828589 1.718349 0.905870

H -8.679898 2.486417 -0.661764

H -8.927645 0.276274 -1.766885

H -6.704806 1.284412 -1.375843

H -10.992083 1.650779 -1.278290

H -8.658223 -1.821067 -0.416804

H -8.790866 -0.870918 1.069110

H -6.501354 -1.226004 0.359305

H -4.423980 0.684927 -0.923290

H -4.131407 -1.826203 0.813238

H -2.107178 0.131922 -0.496217

H -1.681850 -2.373755 1.226114

H 5.040968 0.328923 -2.453534

H 3.153609 -2.242233 -2.686244

H 5.057956 -3.361012 -0.459617

H 5.998999 -1.931306 -0.916715

H 5.240150 -1.995740 0.672291

H 3.848628 -2.998347 1.839833

H 2.407265 -2.810305 2.845643

H 2.415283 -3.990672 1.513960

H 1.836231 1.856326 2.309281

O 2.709232 3.646230 2.041949

H 3.733992 4.173588 0.864537

H 5.342784 3.793020 -1.770176

3b'-c14 , delta G = 1.0241 kcal/mol, population = 4.62 %

C -3.690487 -1.609045 1.214267

C -2.400604 -2.183320 0.622272

C -2.155097 -1.976107 -0.863377

C -2.662736 -0.591004 -1.358302

C -3.294546 0.333527 -0.399211

C -3.445074 1.717694 -0.721810

C -4.129471 2.553170 0.168567

C -4.619119 2.047517 1.391854

C -4.426921 0.722239 1.709246

C -3.772634 -0.136085 0.822113

C -4.950753 -2.349599 0.729733

C -2.803895 -3.093613 -1.685333

C -2.778636 2.362564 -1.853941

C 0.078234 -1.163222 -0.624841

C 1.468392 -1.465368 -0.930317

C 2.463666 -0.668547 -0.490341

C 3.856228 -0.894105 -0.732372

C 4.823274 -0.078333 -0.254178

C 6.233349 -0.271854 -0.465680

C 7.176007 0.541514 0.035129

C 8.650650 0.364250 -0.151253

C 9.343203 -0.060464 1.166685

C 9.249095 0.944098 2.314899

C 9.278421 1.632392 -0.748778

O -0.754384 -2.105592 -1.151631

O -0.347378 -0.211410 0.001255

O -1.612356 -2.760391 1.334775

O -3.633142 -1.750799 2.621291

O -2.520581 -0.342396 -2.540536

O -4.335234 3.863613 -0.039734

O -5.261672 2.872723 2.245061

H 10.358258 1.509314 -0.857652

H 9.703255 1.901724 2.054535

H 9.770099 0.563496 3.195364

H 8.914766 -1.014515 1.485306

H 10.396373 -0.252361 0.939397

H 8.800887 -0.454943 -0.861068

H 6.859423 1.398023 0.626220

H 8.213196 1.132058 2.604088

H 8.857745 1.845798 -1.732689

H 9.100591 2.503249 -0.115167

H 6.532435 -1.130316 -1.062124

H 4.529911 0.786057 0.336685

H 4.133556 -1.763451 -1.321659

H 2.194245 0.206516 0.094510

H 1.665766 -2.355405 -1.513024

H -4.786023 0.345494 2.655695

H -2.769833 -2.147658 2.824641

H -4.840122 -3.425110 0.868571

H -5.781988 -2.001516 1.342175

H -5.184845 -2.133866 -0.310284

H -2.416035 -4.055743 -1.354242

H -3.885636 -3.092038 -1.594945

H -2.539051 -2.931255 -2.728137

H -2.055155 1.777363 -2.421657

O -2.974810 3.543426 -2.153812

H -3.917206 4.083527 -0.916813

H -5.281439 3.762361 1.858724

3b'-c10 , delta G = 1.0912 kcal/mol, population = 4.12 %

C 3.675338 -0.649660 -1.676010

C 3.958686 -1.831993 -0.721768

C 2.976724 -1.968091 0.452926

C 3.234812 -0.655202 1.235733

C 2.903726 0.561420 0.457750

C 2.397447 1.721976 1.101063

C 2.097134 2.850592 0.330626

C 2.283562 2.824303 -1.066766

C 2.775068 1.688575 -1.670854

C 3.091021 0.546957 -0.922679

C 4.946244 -0.274848 -2.423873

C 3.176137 -3.213494 1.278544

C 2.054659 1.747700 2.520942

C 0.597025 -1.557540 0.589507

C -0.643166 -1.657769 -0.158554

C -1.811310 -1.250645 0.383425

C -3.078004 -1.297704 -0.276965

C -4.219969 -0.866232 0.307637

C -5.515394 -0.885981 -0.315694

C -6.629826 -0.444038 0.287223

C -7.999980 -0.438394 -0.310810

C -8.542240 0.999911 -0.432490

C -7.730173 1.887500 -1.371929

C -8.949405 -1.305045 0.530947

O 1.666144 -1.953674 -0.174940

O 0.741297 -1.168484 1.730424

O 4.896407 -2.574784 -0.853296

O 2.745206 -1.126303 -2.665582

O 3.766278 -0.660650 2.323726

O 1.599967 3.990099 0.838198

O 1.972423 3.913391 -1.805702

H -9.948547 -1.313467 0.090436

H -6.702484 2.003590 -1.022400

H -8.172511 2.882400 -1.450604

H -9.576515 0.941565 -0.783522

H -8.579870 1.447564 0.566252

H -7.939306 -0.864277 -1.317923

H -6.553759 -0.050280 1.300043

H -7.690640 1.458642 -2.376625

H -8.593249 -2.334459 0.593767

H -9.032398 -0.912757 1.547742

H -5.575857 -1.278173 -1.327500

H -4.169130 -0.472077 1.319733

H -3.110561 -1.693962 -1.287664

H -1.791124 -0.854778 1.394957

H -0.593896 -2.062855 -1.160742

H 2.899140 1.689897 -2.744702

H 1.942667 -1.398397 -2.198599

H 5.347509 -1.151808 -2.927232

H 4.726280 0.490132 -3.166038

H 5.695803 0.109968 -1.732855

H 3.018818 -4.093447 0.656822

H 4.189584 -3.238613 1.671976

H 2.477858 -3.220240 2.112926

H 2.163591 0.821384 3.088408

O 1.629532 2.761128 3.079851

H 1.502619 3.849621 1.817956

H 1.639256 4.605248 -1.213622

3b'-c61 , delta G = 1.2180 kcal/mol, population = 3.33 %

C -3.141679 2.260693 -0.222278

C -1.642068 1.955378 -0.171344

C -1.150301 1.023779 0.923380

C -2.164259 -0.118560 1.222895

C -3.421382 -0.187205 0.455116

C -4.189458 -1.391710 0.433930

C -5.422630 -1.401360 -0.228330

C -5.877346 -0.252370 -0.910063

C -5.105376 0.887140 -0.919119

C -3.885576 0.927371 -0.239607

C -3.624444 3.110877 0.967056

C -0.845712 1.802783 2.206411

C -3.688754 -2.678209 0.918978

C 0.139693 -0.391381 -0.506255

C 1.475251 -0.836885 -0.867335

C 2.606841 -0.426512 -0.257144

C 3.922338 -0.860863 -0.619655

C 5.036558 -0.425294 0.011021

C 6.376167 -0.834301 -0.320745

C 7.460713 -0.373607 0.323083

C 8.890661 -0.726082 0.043599

C 9.700750 0.525103 -0.370998

C 9.750133 1.637347 0.674501

C 9.066234 -1.842505 -0.984686

O 0.117320 0.460663 0.556613

O -0.882674 -0.735820 -1.069688

O -0.898640 2.431569 -0.997635

O -3.403478 2.998745 -1.400788

O -1.867053 -0.904573 2.102242

O -6.226237 -2.474535 -0.301700

O -7.061044 -0.285420 -1.557981

H 10.123124 -2.092908 -1.088520

H 10.419331 2.437625 0.353112

H 10.119215 1.259966 1.631818

H 10.720029 0.196539 -0.592787

H 9.287343 0.911543 -1.307869

H 9.317744 -1.069737 0.996177

H 7.302235 0.336461 1.130281

H 8.768788 2.082644 0.846409

H 8.532625 -2.748108 -0.691175

H 8.700247 -1.536654 -1.967845

H 6.482468 -1.547936 -1.131124

H 4.928019 0.284372 0.827906

H 4.013579 -1.568777 -1.438501

H 2.528617 0.279744 0.562837

H 1.503460 -1.536863 -1.692756

H -5.448566 1.757283 -1.459294

H -2.559448 3.069897 -1.876915

H -3.648575 2.546344 1.896413

H -2.998729 3.995616 1.085934

H -4.641258 3.429778 0.739753

H -0.445348 1.098012 2.932364

H -0.104027 2.571045 1.992752

H -1.733138 2.268407 2.623262

H -2.642379 -2.738472 1.218856

O -4.391561 -3.691816 0.955793

H -5.770320 -3.208324 0.194133

H -7.446228 -1.169767 -1.454552

3b'-c15 , delta G = 1.2211 kcal/mol, population = 3.31 %

C -3.757862 -1.709326 1.082977

C -2.400809 -2.209317 0.579358

C -2.056394 -1.959653 -0.879896

C -2.591720 -0.588164 -1.384308

C -3.328644 0.286857 -0.453897

C -3.511008 1.671589 -0.755933

C -4.287864 2.458635 0.102186

C -4.841978 1.904591 1.275981

C -4.620648 0.579894 1.576753

C -3.872552 -0.230795 0.719610

C -4.943652 -2.488148 0.484958

C -2.590220 -3.087754 -1.767541

C -2.793344 2.368368 -1.824052

C 0.114231 -1.046245 -0.474605

C 1.535199 -1.280786 -0.680638

C 2.456034 -0.422322 -0.196227

C 3.871220 -0.575487 -0.344168

C 4.759964 0.313147 0.155791

C 6.189188 0.195558 0.032063

C 7.042009 1.100012 0.538958

C 8.541046 1.069031 0.489682

C 9.114444 -0.080097 -0.347171

C 10.629174 -0.011476 -0.536824

C 9.090314 1.082718 1.928717

O -0.633821 -2.020781 -1.065502

O -0.399079 -0.120037 0.123725

O -1.638889 -2.760193 1.339620

O -3.801885 -1.887523 2.486168

O -2.381767 -0.308308 -2.549223

O -4.529259 3.765275 -0.091181

O -5.574914 2.683234 2.099747

H 10.172098 1.216023 1.937173

H 10.969372 -0.800566 -1.209972

H 11.164800 -0.131766 0.405905

H 8.847304 -1.033568 0.120025

H 8.635555 -0.070895 -1.330086

H 8.854959 2.012192 0.020353

H 6.621008 1.952325 1.068991

H 10.926882 0.946861 -0.970516

H 8.649940 1.896635 2.507601

H 8.855467 0.142939 2.434225

H 6.563123 -0.672965 -0.499658

H 4.382673 1.181378 0.690895

H 4.232338 -1.447311 -0.882121

H 2.102204 0.449324 0.347247

H 1.816942 -2.168187 -1.231798

H -5.030206 0.165997 2.486465

H -2.934462 -2.238519 2.748015

H -5.833175 -2.194836 1.041776

H -5.108785 -2.250016 -0.563360

H -4.795616 -3.561814 0.602011

H -2.179326 -4.037426 -1.428323

H -3.674251 -3.140503 -1.754511

H -2.261022 -2.889521 -2.785548

H -2.011277 1.824141 -2.353626

O -3.012982 3.548562 -2.110082

H -4.058934 4.022429 -0.930911

H -5.603620 3.580842 1.732925

3b'-c26 , delta G = 1.3278 kcal/mol, population = 2.77 %

C -3.251686 2.253688 -0.160463

C -1.744964 1.987986 -0.103712

C -1.238304 1.032721 0.963421

C -2.222285 -0.147119 1.213467

C -3.473807 -0.221299 0.437282

C -4.212711 -1.442772 0.372924

C -5.442364 -1.460207 -0.295671

C -5.921761 -0.300870 -0.942144

C -5.177572 0.856435 -0.910039

C -3.961899 0.903767 -0.223521

C -3.765917 3.056078 1.048970

C -0.967642 1.774473 2.275665

C -3.684812 -2.732235 0.820031

C 0.100238 -0.295099 -0.502636

C 1.447700 -0.706133 -0.859721

C 2.563487 -0.310437 -0.211873

C 3.889776 -0.714641 -0.569902

C 4.988220 -0.298487 0.100281

C 6.337640 -0.678470 -0.226352

C 7.405691 -0.240477 0.458740

C 8.847237 -0.554202 0.193044

C 9.616261 0.766303 -0.045886

C 11.134724 0.605876 -0.084878

C 9.067111 -1.566942 -0.929144

O 0.047440 0.516421 0.590508

O -0.908009 -0.634697 -1.093848

O -1.006672 2.514423 -0.903684

O -3.522695 3.019403 -1.319286

O -1.907113 -0.957103 2.064486

O -6.220041 -2.548949 -0.407803

O -7.101464 -0.341293 -1.596731

H 8.512404 -2.488713 -0.746221

H 11.622802 1.578963 -0.166721

H 11.459830 0.004011 -0.934664

H 9.261878 1.209067 -0.982083

H 9.351352 1.470326 0.748132

H 9.248374 -0.981935 1.122923

H 7.231343 0.433730 1.295170

H 11.500165 0.124404 0.826066

H 8.745904 -1.162260 -1.892319

H 10.121331 -1.828956 -1.012978

H 6.466189 -1.349883 -1.068882

H 4.858135 0.371124 0.947242

H 4.002671 -1.383077 -1.418675

H 2.462848 0.357938 0.636923

H 1.499014 -1.369728 -1.713590

H -5.539341 1.735241 -1.423391

H -2.675890 3.130313 -1.782694

H -3.784224 2.464079 1.961211

H -3.163421 3.951928 1.199670

H -4.788237 3.356517 0.821304

H -1.872663 2.195888 2.701719

H -0.549863 1.057191 2.979170

H -0.249721 2.573404 2.095861

H -2.639122 -2.777978 1.124601

O -4.364099 -3.762465 0.820384

H -5.749172 -3.287562 0.066556

H -7.465503 -1.237565 -1.523827

3b'-c13 , delta G = 1.3950 kcal/mol, population = 2.47 %

C -3.726547 -1.733038 1.012477

C -2.357216 -2.221207 0.530848

C -1.976811 -1.937024 -0.913172

C -2.496503 -0.551681 -1.395420

C -3.254896 0.300779 -0.461314

C -3.426623 1.693132 -0.732258

C -4.225488 2.459583 0.124196

C -4.812069 1.876836 1.267961

C -4.600214 0.544522 1.540902

C -3.830207 -0.245451 0.683833

C -4.897704 -2.493620 0.364632

C -2.491425 -3.040638 -1.841974

C -2.677126 2.415485 -1.760753

C 0.186362 -1.039401 -0.436611

C 1.611010 -1.281064 -0.605048

C 2.523812 -0.429629 -0.093725

C 3.941313 -0.592760 -0.202791

C 4.822709 0.292546 0.315988

C 6.253186 0.162995 0.231977

C 7.100996 1.066991 0.746847

C 8.600791 1.004957 0.765030

C 9.225361 -0.025578 -0.182614

C 9.065820 0.308013 -1.665611

C 9.071665 0.781257 2.215462

O -0.550374 -1.999295 -1.064419

O -0.337992 -0.119201 0.161458

O -1.613234 -2.788345 1.296911

O -3.806631 -1.946880 2.409051

O -2.258394 -0.242667 -2.547455

O -4.459370 3.771350 -0.042135

O -5.566159 2.635451 2.091337

H 8.637400 1.517772 2.894095

H 9.549185 -0.446291 -2.289623

H 9.524841 1.273001 -1.894593

H 10.291186 -0.090563 0.052896

H 8.813191 -1.016669 0.031351

H 8.961253 1.997018 0.462758

H 6.673613 1.930684 1.252344

H 8.017462 0.363959 -1.961387

H 8.777181 -0.211817 2.562243

H 10.158838 0.859436 2.278556

H 6.633333 -0.716025 -0.277769

H 4.437916 1.167427 0.834595

H 4.310909 -1.469784 -0.726388

H 2.161263 0.443763 0.441104

H 1.902349 -2.168188 -1.151619

H -5.034608 0.108312 2.428397

H -2.945408 -2.302556 2.684708

H -4.754993 -3.570285 0.458155

H -5.800902 -2.211908 0.905161

H -5.034209 -2.228383 -0.681191

H -3.575540 -3.090020 -1.857401

H -2.136115 -2.817925 -2.846065

H -2.091769 -3.999916 -1.516745

H -1.880527 1.883252 -2.280820

O -2.886318 3.603147 -2.022464

H -3.965378 4.048930 -0.861452

H -5.583880 3.542280 1.747252

3b'-c65 , delta G = 1.6284 kcal/mol, population = 1.66 %

C 3.360206 -2.193936 -0.178418

C 1.840628 -2.018851 -0.119482

C 1.279037 -1.099000 0.951409

C 2.191131 0.136050 1.206805

C 3.438464 0.286673 0.435014

C 4.107078 1.548864 0.379011

C 5.333547 1.640427 -0.289536

C 5.878937 0.514227 -0.942287

C 5.202654 -0.683978 -0.916479

C 3.991210 -0.804982 -0.231441

C 3.917934 -2.975808 1.025362

C 1.054163 -1.859001 2.261734

C 3.509648 2.803638 0.837821

C -0.136929 0.142657 -0.516575

C -1.507023 0.469380 -0.874926

C -2.596312 0.005807 -0.227101

C -3.945372 0.323641 -0.587390

C -5.015446 -0.158894 0.084095

C -6.386337 0.131296 -0.245584

C -7.423983 -0.368160 0.444372

C -8.883811 -0.122010 0.201364

C -9.176439 0.699561 -1.059164

C -10.662843 0.804048 -1.397990

C -9.506748 0.497832 1.466379

O -0.035175 -0.659163 0.580289

O 0.848942 0.539325 -1.109813

O 1.134166 -2.586866 -0.919731

O 3.675316 -2.933723 -1.343100

O 1.826009 0.923215 2.059288

O 6.048076 2.772143 -0.394767

O 7.054402 0.625895 -1.596051

H -10.592952 0.546327 1.388250

H -11.216615 1.360872 -0.640635

H -11.116000 -0.187336 -1.481847

H -8.757035 1.703748 -0.939234

H -8.653517 0.243549 -1.904232

H -9.346562 -1.110436 0.070070

H -7.205551 -1.013424 1.293275

H -10.805854 1.316418 -2.351152

H -9.262597 -0.091780 2.351998

H -9.127126 1.510907 1.619261

H -6.556805 0.783656 -1.095558

H -4.843536 -0.814555 0.934508

H -4.099932 0.979827 -1.439169

H -2.454293 -0.652939 0.623305

H -1.599012 1.125618 -1.731014

H 5.614321 -1.537615 -1.434566

H 2.835815 -3.092813 -1.805726

H 3.365735 -3.904800 1.168220

H 4.955568 -3.217332 0.796931

H 3.902005 -2.391470 1.942487

H 1.984331 -2.216906 2.691538

H 0.585608 -1.172305 2.963822

H 0.393029 -2.704906 2.079428

H 2.465262 2.788450 1.149483

O 4.130012 3.870557 0.841846

H 5.535501 3.479906 0.083820

H 7.365870 1.541394 -1.518921

3b'-c21 , delta G = 1.7225 kcal/mol, population = 1.42 %

C 3.280983 -2.231170 0.097952

C 1.767330 -2.011947 0.027027

C 1.163993 -0.977201 0.961334

C 2.100560 0.249734 1.165682

C 3.397050 0.294448 0.464525

C 4.105889 1.528149 0.330729

C 5.371605 1.524179 -0.267561

C 5.917610 0.327737 -0.779552

C 5.205558 -0.845920 -0.680041

C 3.954747 -0.869970 -0.058978

C 3.732035 -2.883963 1.417406

C 0.817161 -1.601662 2.315877

C 3.520770 2.835469 0.630848

C -0.099809 0.165247 -0.713536

C -1.429128 0.509460 -1.190795

C -2.576474 0.132574 -0.588749

C -3.884958 0.474330 -1.059958

C -5.015586 0.077345 -0.433235

C -6.347914 0.403560 -0.868432

C -7.456737 -0.001120 -0.230102

C -8.856339 0.324272 -0.649267

C -9.552743 1.263069 0.365172

C -9.731362 0.698520 1.774343

C -9.661425 -0.961783 -0.891473

O -0.103844 -0.535260 0.454960

O 0.938109 0.470941 -1.270453

O 1.100894 -2.636132 -0.765665

O 3.652119 -3.103223 -0.952510

O 1.709378 1.122341 1.917443

O 6.125981 2.622268 -0.434296

O 7.130617 0.347559 -1.371101

H -9.218711 -1.548353 -1.697962

H -10.240099 1.424126 2.411695

H -8.772515 0.468398 2.242989

H -8.980060 2.192932 0.418044

H -10.532713 1.523935 -0.046062

H -8.802208 0.871273 -1.595473

H -7.345955 -0.616178 0.660253

H -10.331132 -0.213313 1.773328

H -9.689877 -1.591093 0.000059

H -10.690531 -0.721394 -1.167754

H -6.441386 1.012935 -1.764016

H -4.927583 -0.530187 0.464401

H -3.956778 1.080636 -1.958391

H -2.517729 -0.469023 0.312221

H -1.437626 1.103359 -2.095953

H 5.620009 -1.755843 -1.088619

H 2.843173 -3.287770 -1.458236

H 3.147565 -3.781109 1.621956

H 4.776649 -3.167940 1.293397

H 3.666837 -2.199262 2.260043

H 0.132899 -2.434829 2.162543

H 1.698694 -1.953824 2.842134

H 0.332743 -0.834829 2.916939

H 2.459971 2.875714 0.878361

O 4.171524 3.882380 0.571763

H 5.607996 3.385658 -0.058124

H 7.463266 1.258841 -1.364190

# Table S79. Geometry data of conformers of structure 4a

4a-c7 , delta G = 0.0000 kcal/mol, population = 42.44 %

C 3.422132 -1.633508 -0.550534

C 2.352709 -1.620616 0.549115

C 2.090037 -0.322020 1.306852

C 2.572284 0.928385 0.542497

C 3.910389 0.812995 -0.055229

C 4.770103 1.933256 -0.142464

C 6.061268 1.746582 -0.618966

C 6.506308 0.488093 -1.017960

C 5.655703 -0.612446 -0.955718

C 4.370966 -0.453474 -0.471546

C 2.699721 -1.610073 -1.913736

C 2.866986 -0.398604 2.632377

C 4.361815 3.315398 0.288658

C -0.183393 -0.196445 0.651344

C -1.534515 0.014689 1.148491

C -2.590993 0.043394 0.309935

C -3.947898 0.249057 0.716376

C -4.979030 0.269852 -0.159326

C -6.355463 0.473669 0.207032

C -7.362633 0.485381 -0.679495

C -8.806050 0.697203 -0.351225

C -9.647499 -0.537001 -0.732977

C -9.276913 -1.799049 0.041508

C -9.334009 1.951930 -1.063953

O 0.718658 -0.202545 1.672797

O 0.157341 -0.364265 -0.504874

O 1.785128 -2.646612 0.844483

O 4.173462 -2.837039 -0.419866

O 1.904182 1.943748 0.569500

H 6.736001 2.592966 -0.673835

O 7.762961 0.275862 -1.485257

H -10.383273 2.119762 -0.811986

H -9.377246 -1.638971 1.118239

H -8.245018 -2.097849 -0.153149

H -10.699758 -0.294530 -0.558679

H -9.544576 -0.714331 -1.808803

H -8.895783 0.847104 0.729883

H -7.133487 0.330177 -1.733092

H -9.925583 -2.632651 -0.234715

H -8.765970 2.837849 -0.775465

H -9.260865 1.840731 -2.148867

H -6.572469 0.622905 1.261665

H -4.768775 0.123546 -1.216094

H -4.143183 0.393431 1.775090

H -2.405214 -0.098133 -0.751124

H -1.653555 0.149364 2.215608

H 6.002939 -1.578427 -1.290340

H 3.533012 -3.548600 -0.276751

H 2.127298 -0.694749 -2.043896

H 3.454657 -1.685277 -2.695861

H 2.018320 -2.458818 -1.981706

H 2.485859 -1.235224 3.215604

H 3.930897 -0.541148 2.452505

H 2.721164 0.528062 3.186545

H 3.611348 3.729751 -0.384935

H 5.227894 3.975934 0.294618

H 3.916727 3.311105 1.283915

H 8.262640 1.102940 -1.494991

4a-c6 , delta G = 0.2428 kcal/mol, population = 28.15 %

C 3.526290 -1.629116 -0.533828

C 2.467584 -1.632560 0.576341

C 2.180416 -0.334801 1.326518

C 2.624578 0.921165 0.548358

C 3.956914 0.834294 -0.066794

C 4.787756 1.976929 -0.177455

C 6.072448 1.820490 -0.675488

C 6.542410 0.571349 -1.074851

C 5.722725 -0.550654 -0.988996

C 4.441554 -0.421592 -0.480755

C 2.791053 -1.647300 -1.889747

C 2.969745 -0.381179 2.645875

C 4.351095 3.351000 0.251935

C -0.098855 -0.264874 0.686361

C -1.451441 -0.078100 1.188847

C -2.510445 -0.064885 0.353000

C -3.869759 0.118101 0.761972

C -4.901399 0.129223 -0.113370

C -6.280730 0.311558 0.253803

C -7.287148 0.321848 -0.634060

C -8.733721 0.505300 -0.300707

C -9.524004 -0.763759 -0.684472

C -10.995823 -0.729006 -0.275941

C -9.279856 1.760109 -1.000274

O 0.809157 -0.245948 1.702370

O 0.239095 -0.432712 -0.470787

O 1.927925 -2.669591 0.884988

O 4.315987 -2.807310 -0.392718

O 1.933281 1.920794 0.577624

H 6.733750 2.673519 -0.752929

O 7.813033 0.507897 -1.548156

H -10.315925 1.948730 -0.717433

H -11.099775 -0.542024 0.796105

H -11.478329 -1.683321 -0.496084

H -9.442542 -0.915747 -1.766000

H -9.040376 -1.623724 -0.213284

H -8.822339 0.644557 0.781920

H -7.054833 0.185267 -1.689312

H -11.547983 0.048425 -0.806048

H -8.692613 2.639830 -0.732228

H -9.240853 1.643786 -2.086694

H -6.500097 0.447671 1.309848

H -4.689009 -0.006861 -1.171079

H -4.066395 0.253516 1.821598

H -2.324476 -0.200717 -0.708766

H -1.569302 0.053991 2.256407

H 6.077097 -1.517499 -1.319591

H 3.699097 -3.538641 -0.246367

H 2.134455 -2.516673 -1.938353

H 2.190650 -0.751111 -2.026573

H 3.539885 -1.711661 -2.678661

H 2.615782 -1.223113 3.238520

H 4.035597 -0.497003 2.458121

H 2.804323 0.545526 3.194453

H 3.578802 3.741510 -0.411167

H 5.200372 4.032818 0.237948

H 3.923654 3.342579 1.254892

H 8.035229 -0.399912 -1.793427

4a-c12 , delta G = 0.6457 kcal/mol, population = 14.25 %

C 3.844998 -1.582830 0.009848

C 2.379763 -1.663404 0.455766

C 1.574929 -0.372307 0.579016

C 2.184926 0.796404 -0.222302

C 3.640431 0.950132 -0.086249

C 4.245227 2.228989 -0.106863

C 5.610075 2.324155 0.132619

C 6.378950 1.188187 0.376275

C 5.791583 -0.074339 0.374394

C 4.432757 -0.192326 0.152116

C 3.916508 -2.010482 -1.471083

C 1.568252 0.025616 2.064981

C 3.471953 3.497655 -0.343106

C -0.039918 -1.003277 -1.034947

C -1.454400 -1.071673 -1.362552

C -2.448657 -0.694660 -0.530791

C -3.840844 -0.745732 -0.862273

C -4.811274 -0.352407 -0.005642

C -6.219309 -0.376253 -0.301194

C -7.162906 0.028474 0.562674

C -8.635929 0.025746 0.304894

C -9.215257 1.451684 0.394149

C -8.651475 2.412413 -0.649444

C -9.347269 -0.908259 1.296388

O 0.208945 -0.574269 0.232383

O 0.862201 -1.304840 -1.796047

O 1.906378 -2.731508 0.767844

O 4.599504 -2.486498 0.812233

O 1.451597 1.570676 -0.806379

H 6.082654 3.299543 0.139323

O 7.713713 1.251554 0.614859

H -9.193953 -0.570319 2.324478

H -7.574203 2.543584 -0.529685

H -9.120238 3.395288 -0.570884

H -10.301021 1.382083 0.281548

H -9.032754 1.843184 1.400554

H -8.808177 -0.347327 -0.710128

H -6.853863 0.394472 1.541042

H -8.828174 2.036749 -1.660709

H -10.421909 -0.921892 1.102345

H -8.971000 -1.929359 1.216019

H -6.515262 -0.739498 -1.282060

H -4.522615 0.013490 0.976816

H -4.113792 -1.113704 -1.847192

H -2.190024 -0.322304 0.455114

H -1.666210 -1.444158 -2.356881

H 6.401167 -0.950037 0.539486

H 4.081652 -3.302394 0.870120

H 4.965248 -2.020926 -1.766823

H 3.495380 -3.009894 -1.585083

H 3.362833 -1.326323 -2.109464

H 1.014310 0.956354 2.182700

H 1.082956 -0.762111 2.638843

H 2.582030 0.166860 2.434714

H 4.103885 4.362643 -0.145681

H 2.589331 3.555121 0.294374

H 3.115638 3.553109 -1.372017

H 8.018729 2.167794 0.579961

4a-c31 , delta G = 0.8823 kcal/mol, population = 9.56 %

C 3.462274 -1.667742 -0.433118

C 2.441577 -1.599893 0.709924

C 2.165910 -0.255647 1.377405

C 2.572386 0.947497 0.501736

C 3.881713 0.827297 -0.155515

C 4.694759 1.964946 -0.373736

C 5.964202 1.784333 -0.907118

C 6.432384 0.514353 -1.237131

C 5.627181 -0.604943 -1.045179

C 4.365672 -0.451723 -0.501097

C 2.681642 -1.792306 -1.757826

C 2.996625 -0.202641 2.670647

C 4.261911 3.362372 -0.023035

C -0.131471 -0.240624 0.804204

C -1.470927 -0.029802 1.332304

C -2.548642 -0.049523 0.520967

C -3.898754 0.157155 0.948944

C -4.944594 0.150316 0.090587

C -6.316594 0.358511 0.471364

C -7.332906 0.362793 -0.405642

C -8.771121 0.580379 -0.053714

C -9.622509 -0.685632 -0.313192

C -9.685315 -1.153030 -1.767208

C -9.326304 1.808263 -0.793042

O 0.805696 -0.152769 1.789481

O 0.173764 -0.478128 -0.349873

O 1.920729 -2.616322 1.107089

O 4.269151 -2.825696 -0.233086

O 1.876238 1.944324 0.492156

H 6.604052 2.645314 -1.061615

O 7.668915 0.308235 -1.757648

H -10.382628 1.951153 -0.554193

H -8.696666 -1.409157 -2.153022

H -10.111856 -0.389727 -2.420446

H -9.231214 -1.494326 0.310091

H -10.635937 -0.479762 0.044503

H -8.825414 0.780911 1.020508

H -7.107250 0.206096 -1.458036

H -10.310643 -2.043806 -1.851909

H -8.785158 2.710508 -0.503582

H -9.236486 1.698759 -1.875314

H -6.520851 0.522154 1.526624

H -4.749885 -0.020159 -0.965577

H -4.075405 0.329373 2.006723

H -2.385552 -0.231092 -0.537716

H -1.562281 0.152440 2.395053

H 5.990963 -1.581991 -1.325873

H 3.665099 -3.550563 -0.017458

H 2.031298 -2.666868 -1.718545

H 2.068642 -0.912975 -1.940876

H 3.404210 -1.910915 -2.564773

H 4.057802 -0.314903 2.456193

H 2.832999 0.756150 3.161629

H 2.676059 -1.008242 3.329211

H 3.872136 3.419464 0.993533

H 3.461835 3.701281 -0.681531

H 5.103369 4.047876 -0.115015

H 8.138215 1.147613 -1.852721

4a-c32 , delta G = 1.3843 kcal/mol, population = 4.09 %

C 3.909239 -1.575263 0.071994

C 2.438761 -1.623629 0.505792

C 1.648481 -0.320083 0.581348

C 2.277099 0.814899 -0.253059

C 3.733664 0.955783 -0.114900

C 4.353709 2.226174 -0.176479

C 5.718964 2.313491 0.063361

C 6.473851 1.177338 0.346887

C 5.871423 -0.077318 0.386412

C 4.511633 -0.187030 0.165136

C 3.989563 -2.062926 -1.389742

C 1.636943 0.124938 2.053746

C 3.595765 3.495323 -0.456027

C 0.040287 -0.987972 -1.023438

C -1.372215 -1.061143 -1.358355

C -2.370334 -0.649853 -0.547782

C -3.760725 -0.711079 -0.885101

C -4.735344 -0.282518 -0.050440

C -6.142191 -0.322183 -0.350613

C -7.091148 0.114522 0.491991

C -8.562271 0.099882 0.221411

C -9.100592 1.545811 0.173246

C -10.572395 1.649873 -0.223073

C -9.283531 -0.750805 1.278797

O 0.282950 -0.518617 0.230834

O 0.945991 -1.319429 -1.767718

O 1.949177 -2.675756 0.845865

O 4.646386 -2.453780 0.917785

O 1.556034 1.578814 -0.865413

H 6.202998 3.282982 0.038527

O 7.808745 1.232842 0.586588

H -8.877673 -1.763359 1.304541

H -10.871387 2.695640 -0.318019

H -11.227857 1.186593 0.515892

H -8.945881 2.013091 1.151699

H -8.495683 2.112609 -0.539673

H -8.726517 -0.354600 -0.761437

H -6.788293 0.520815 1.456033

H -10.753940 1.162377 -1.184499

H -9.164894 -0.311966 2.273168

H -10.350567 -0.821906 1.066012

H -6.432772 -0.729697 -1.315724

H -4.451537 0.123858 0.917421

H -4.028876 -1.118716 -1.855610

H -2.116403 -0.241242 0.424881

H -1.579047 -1.469382 -2.339617

H 6.470252 -0.953904 0.583224

H 4.124899 -3.265715 0.994461

H 3.558448 -3.061602 -1.467150

H 3.448730 -1.399360 -2.060158

H 5.040580 -2.096391 -1.675493

H 1.136273 -0.637197 2.648566

H 2.650348 0.262699 2.425940

H 1.096141 1.066927 2.138384

H 2.712073 3.582770 0.176491

H 3.243128 3.521829 -1.487362

H 4.236940 4.358945 -0.284078

H 8.124438 2.143906 0.523377

4a-c37 , delta G = 1.9729 kcal/mol, population = 1.51 %

C 3.515901 -1.669224 -0.250242

C 2.406831 -1.551413 0.802382

C 2.031556 -0.168642 1.328568

C 2.463131 0.973046 0.384548

C 3.825221 0.854178 -0.155054

C 4.612725 2.002712 -0.406473

C 5.928139 1.831341 -0.817416

C 6.467027 0.559073 -0.995653

C 5.688666 -0.573272 -0.771249

C 4.381039 -0.427815 -0.347375

C 2.845623 -1.925982 -1.616116

C 2.757798 0.025454 2.670478

C 4.102085 3.405083 -0.217523

C -0.216105 -0.300162 0.591078

C -1.596312 -0.093301 1.002141

C -2.614924 -0.251943 0.131630

C -3.996617 -0.054795 0.448106

C -4.985025 -0.213246 -0.462369

C -6.383267 -0.010831 -0.190438

C -7.338537 -0.165532 -1.120940

C -8.821170 -0.018913 -0.935250

C -9.250553 0.726398 0.334002

C -8.886046 2.210652 0.341188

C -9.474483 -1.413047 -1.007753

O 0.641446 -0.086811 1.628596

O 0.181556 -0.629572 -0.511111

O 1.895042 -2.552752 1.246891

O 4.341027 -2.776314 0.102032

O 1.733512 1.933663 0.232454

H 6.548517 2.702247 -0.994199

O 7.748879 0.362626 -1.396729

H -10.562459 -1.322148 -1.006310

H -9.349885 2.725392 -0.504052

H -7.808535 2.363894 0.270048

H -10.335547 0.626921 0.426668

H -8.829962 0.227927 1.212850

H -9.191388 0.551010 -1.797163

H -7.029349 -0.470878 -2.118548

H -9.233132 2.693663 1.256735

H -9.177943 -1.945726 -1.913212

H -9.178144 -2.017186 -0.147201

H -6.645507 0.287349 0.819310

H -4.718512 -0.507713 -1.474673

H -4.247187 0.239789 1.463187

H -2.374958 -0.545626 -0.886521

H -1.767909 0.200651 2.029350

H 6.108674 -1.554206 -0.935766

H 3.745293 -3.502487 0.335635

H 2.224603 -2.820497 -1.556475

H 2.218784 -1.088727 -1.913575

H 3.632562 -2.076917 -2.354500

H 2.522320 1.013508 3.064489

H 2.416428 -0.735956 3.369887

H 3.835540 -0.059195 2.544768

H 4.923077 4.115871 -0.302480

H 3.626399 3.532444 0.755166

H 3.348420 3.650788 -0.966041

H 8.191875 1.210163 -1.536064

# Table S80. Geometry data of conformers of structure 4a'

4a'-c2 , delta G = 0.0000 kcal/mol, population = 47.65 %

C -3.495483 -1.576776 -0.586342

C -2.439850 -1.652679 0.523995

C -2.109897 -0.390369 1.316288

C -2.509897 0.904407 0.578967

C -3.844269 0.882945 -0.037805

C -4.637068 2.055286 -0.109107

C -5.925302 1.958741 -0.613341

C -6.435321 0.740190 -1.055779

C -5.653198 -0.410493 -1.007452

C -4.369607 -0.341468 -0.493977

C -2.759529 -1.575490 -1.941969

C -2.903343 -0.452866 2.632499

C -4.156548 3.398058 0.369861

C 0.172208 -0.388642 0.681809

C 1.530393 -0.280727 1.192378

C 2.592382 -0.311368 0.360791

C 3.958014 -0.213663 0.778049

C 4.992829 -0.254579 -0.092660

C 6.378857 -0.159982 0.281860

C 7.387307 -0.201358 -0.602360

C 8.841722 -0.110396 -0.266995

C 9.486773 1.120816 -0.933794

C 8.915720 2.452109 -0.452741

C 9.566673 -1.396533 -0.693089

O -0.737188 -0.361348 1.696269

O -0.169021 -0.504616 -0.480726

O -1.937346 -2.717320 0.799641

O -4.324970 -2.731448 -0.484482

O -1.784524 1.878146 0.638908

H -6.557994 2.835299 -0.661104

O -7.706470 0.734177 -1.532041

H 10.626868 -1.337888 -0.437796

H 9.429590 3.291991 -0.924438

H 9.027874 2.555749 0.629725

H 10.561707 1.088104 -0.734059

H 9.370829 1.034831 -2.019432

H 8.939498 0.001297 0.818041

H 7.150549 -0.311150 -1.659946

H 7.852040 2.536652 -0.684096

H 9.142374 -2.271322 -0.197689

H 9.487042 -1.546389 -1.772867

H 6.601854 -0.047451 1.339742

H 4.777803 -0.365657 -1.152741

H 4.156702 -0.103671 1.840204

H 2.404181 -0.419056 -0.703802

H 1.650339 -0.176767 2.262804

H -6.038816 -1.353187 -1.370894

H -3.733549 -3.487676 -0.361419

H -2.130971 -0.694916 -2.050753

H -3.509414 -1.591176 -2.732365

H -2.130849 -2.463304 -2.018248

H -2.705262 0.447873 3.212586

H -2.582097 -1.327025 3.196450

H -3.972196 -0.522615 2.439259

H -3.727579 3.338592 1.370434

H -4.984022 4.106208 0.384033

H -3.373717 3.788668 -0.280710

H -7.958017 -0.157706 -1.805989

4a'-c5 , delta G = 0.7009 kcal/mol, population = 14.58 %

C 3.645419 -1.679696 -0.010838

C 2.167534 -1.555694 -0.399460

C 1.548956 -0.166516 -0.529338

C 2.347162 0.922806 0.215310

C 3.805877 0.860038 0.038617

C 4.591905 2.039359 0.015674

C 5.949112 1.931412 -0.248431

C 6.541780 0.691314 -0.475632

C 5.778390 -0.471853 -0.433680

C 4.419013 -0.387893 -0.186382

C 3.712814 -2.114213 1.467941

C 1.535640 0.196491 -2.023847

C 4.014802 3.411719 0.232440

C -0.066608 -0.547794 1.158273

C -1.472033 -0.492366 1.524183

C -2.465616 -0.117965 0.690433

C -3.850499 -0.087823 1.053631

C -4.827746 0.272510 0.190371

C -6.229878 0.297517 0.512852

C -7.185268 0.640509 -0.364662

C -8.653267 0.673367 -0.081769

C -9.411895 -0.321057 -0.983437

C -9.019554 -1.779085 -0.759643

C -9.199413 2.097220 -0.270192

O 0.182742 -0.173623 -0.125854

O 0.827433 -0.908871 1.903120

O 1.531892 -2.551292 -0.658122

O 4.234979 -2.679679 -0.838648

O 1.752177 1.813617 0.790369

H 6.567100 2.818722 -0.289898

O 7.875170 0.675870 -0.729717

H -8.694903 2.803839 0.390809

H -9.608163 -2.444908 -1.393806

H -9.186059 -2.072685 0.280113

H -10.482524 -0.195424 -0.797931

H -9.244631 -0.045644 -2.030170

H -8.810895 0.374472 0.959903

H -6.890810 0.919324 -1.375685

H -7.964516 -1.946326 -0.984876

H -9.054761 2.433651 -1.300025

H -10.269149 2.126794 -0.052449

H -6.511926 0.012706 1.523267

H -4.551424 0.558933 -0.821549

H -4.113268 -0.377544 2.067055

H -2.214078 0.174770 -0.323802

H -1.680443 -0.793306 2.543170

H 6.238893 -1.437789 -0.589165

H 3.625932 -3.431840 -0.841908

H 3.157083 -3.042945 1.602130

H 3.287091 -1.357935 2.122904

H 4.759396 -2.275551 1.724767

H 1.105137 1.189896 -2.145571

H 0.928386 -0.531562 -2.559101

H 2.544022 0.194104 -2.433359

H 3.138268 3.582806 -0.392958

H 4.762413 4.170249 0.004200

H 3.689465 3.541096 1.264846

H 8.179464 -0.229927 -0.874579

4a'-c13 , delta G = 0.8609 kcal/mol, population = 11.12 %

C 3.831119 -1.640652 -0.099179

C 2.351153 -1.606502 -0.501903

C 1.633536 -0.261269 -0.566135

C 2.341247 0.837971 0.252224

C 3.800964 0.896094 0.088768

C 4.493774 2.131166 0.139225

C 5.856558 2.141351 -0.117489

C 6.542965 0.964359 -0.407755

C 5.870939 -0.254484 -0.437848

C 4.507765 -0.287622 -0.199273

C 3.914408 -2.142131 1.357468

C 1.615192 0.181321 -2.039062

C 3.811292 3.441009 0.425457

C 0.026581 -0.830228 1.076819

C -1.380200 -0.828550 1.441951

C -2.376621 -0.397474 0.639591

C -3.760561 -0.388319 1.007443

C -4.735861 0.047977 0.177610

C -6.135859 0.076914 0.509472

C -7.087179 0.509933 -0.332362

C -8.552300 0.553580 -0.034174

C -9.307616 -0.407476 -0.976905

C -10.799551 -0.533087 -0.672439

C -9.068727 1.996890 -0.145510

O 0.266656 -0.383627 -0.186204

O 0.929804 -1.201032 1.805491

O 1.796067 -2.630805 -0.825713

O 4.501947 -2.552001 -0.966012

O 1.676426 1.643292 0.874849

H 6.405961 3.073540 -0.103742

O 7.875043 1.064925 -0.648815

H -8.506593 2.661827 0.512202

H -10.962307 -0.835826 0.365238

H -11.261018 -1.285010 -1.315590

H -9.166529 -0.071686 -2.009737

H -8.841526 -1.393915 -0.905211

H -8.704923 0.207535 0.993583

H -6.791777 0.859543 -1.320618

H -11.329285 0.407047 -0.833373

H -8.965094 2.364811 -1.169815

H -10.121258 2.061811 0.131879

H -6.418860 -0.273954 1.498789

H -4.458573 0.404665 -0.811485

H -4.023258 -0.748769 1.997920

H -2.126643 -0.029875 -0.350315

H -1.584315 -1.201391 2.437759

H 6.405465 -1.172491 -0.640448

H 3.943556 -3.340424 -1.025272

H 4.967221 -2.235482 1.622470

H 3.430458 -3.116153 1.437102

H 3.423977 -1.454520 2.042310

H 2.626467 0.263746 -2.432789

H 1.124259 1.150987 -2.114741

H 1.061671 -0.553474 -2.621313

H 4.498255 4.266300 0.242945

H 3.474113 3.489226 1.461142

H 2.925799 3.577122 -0.195915

H 8.249111 0.194165 -0.837711

4a'-c9 , delta G = 0.9701 kcal/mol, population = 9.25 %

C -3.569043 -1.566022 -0.589821

C -2.516841 -1.639838 0.523970

C -2.190576 -0.376410 1.315966

C -2.589407 0.917207 0.575909

C -3.921752 0.893975 -0.045066

C -4.715249 2.065605 -0.120322

C -6.001738 1.967419 -0.628663

C -6.509316 0.747937 -1.071330

C -5.726434 -0.402065 -1.019096

C -4.444572 -0.331400 -0.501535

C -2.828858 -1.565498 -1.943154

C -2.988177 -0.438330 2.629657

C -4.237403 3.409342 0.358592

C 0.093325 -0.370370 0.688419

C 1.449490 -0.253539 1.202301

C 2.513512 -0.276726 0.373060

C 3.877352 -0.164670 0.792493

C 4.913722 -0.193382 -0.076860

C 6.298056 -0.079380 0.299022

C 7.308289 -0.108298 -0.584109

C 8.759574 0.017529 -0.244489

C 9.338457 1.292537 -0.893962

C 10.787389 1.585678 -0.507717

C 9.514680 -1.247999 -0.680719

O -0.819111 -0.345093 1.700221

O -0.244133 -0.490714 -0.474780

O -2.014519 -2.703807 0.802521

O -4.397788 -2.721345 -0.489382

O -1.864957 1.891569 0.636957

H -6.634968 2.843407 -0.679561

O -7.778899 0.740353 -1.551729

H 9.471069 -1.367949 -1.766532

H 11.469975 0.816224 -0.871491

H 10.897817 1.645176 0.578166

H 9.255450 1.202854 -1.982301

H 8.710577 2.139412 -0.604119

H 8.851144 0.119859 0.842046

H 7.075150 -0.227746 -1.641229

H 11.112003 2.539222 -0.928833

H 10.563915 -1.201127 -0.387687

H 9.077602 -2.137141 -0.223537

H 6.517964 0.037711 1.357204

H 4.701340 -0.308260 -1.137069

H 4.073218 -0.051041 1.854787

H 2.328288 -0.387509 -0.691732

H 1.566139 -0.146527 2.272783

H -6.110139 -1.345492 -1.382661

H -3.806076 -3.476823 -0.363146

H -2.200594 -0.684589 -2.050849

H -3.576266 -1.582505 -2.735858

H -2.199308 -2.452925 -2.016585

H -2.792943 0.463212 3.209440

H -2.667862 -1.311567 3.195553

H -4.056314 -0.509428 2.433005

H -5.065521 4.116795 0.369301

H -3.452854 3.799856 -0.289955

H -3.811561 3.351431 1.360583

H -8.028868 -0.152036 -1.825457

4a'-c14 , delta G = 1.2927 kcal/mol, population = 5.36 %

C -3.478739 -1.528328 -0.728002

C -2.442128 -1.665972 0.393426

C -2.157105 -0.461252 1.286306

C -2.576855 0.877519 0.644700

C -3.899567 0.871435 0.002436

C -4.722021 2.025423 0.008336

C -6.001733 1.934384 -0.518453

C -6.475337 0.739763 -1.056194

C -5.662861 -0.390398 -1.086691

C -4.387171 -0.327249 -0.553572

C -2.717723 -1.397917 -2.063784

C -2.972451 -0.643171 2.578161

C -4.282924 3.340145 0.593070

C 0.137561 -0.360493 0.702308

C 1.482359 -0.279191 1.252195

C 2.564667 -0.229170 0.448367

C 3.917728 -0.161667 0.910655

C 4.978702 -0.125878 0.071902

C 6.351847 -0.071220 0.499205

C 7.391779 -0.044632 -0.349157

C 8.830568 0.004949 0.060304

C 9.492086 1.348283 -0.330819

C 9.561515 1.640464 -1.829567

C 9.595005 -1.200104 -0.510102

O -0.792982 -0.432024 1.695136

O -0.176324 -0.382985 -0.473270

O -1.917203 -2.736327 0.595252

O -4.277673 -2.708389 -0.737608

O -1.879058 1.861277 0.797337

H -6.656610 2.795754 -0.508232

O -7.741736 0.736537 -1.544930

H 9.524750 -1.239778 -1.598666

H 10.136608 0.882586 -2.364285

H 10.045293 2.603208 -2.005346

H 8.949989 2.154744 0.170500

H 10.504589 1.349036 0.084191

H 8.869156 -0.056794 1.152008

H 7.189824 -0.067617 -1.417763

H 8.568350 1.686747 -2.280649

H 10.652267 -1.142722 -0.241563

H 9.192194 -2.134682 -0.116367

H 6.537259 -0.055650 1.570404

H 4.797957 -0.143379 -1.000166

H 4.084657 -0.145168 1.983836

H 2.404936 -0.245705 -0.626123

H 1.575772 -0.266980 2.330230

H -6.018706 -1.312488 -1.525514

H -3.668162 -3.456658 -0.664099

H -2.064936 -2.261035 -2.198787

H -2.110022 -0.496567 -2.088472

H -3.452256 -1.367571 -2.868077

H -4.035901 -0.717475 2.359185

H -2.803031 0.212523 3.230911

H -2.643159 -1.552856 3.077541

H -5.127336 4.026438 0.641729

H -3.497524 3.794868 -0.011021

H -3.871811 3.217031 1.595346

H -7.967569 -0.138860 -1.886259

4a'-c44 , delta G = 1.3887 kcal/mol, population = 4.56 %

C -3.501619 -1.708740 -0.241687

C -2.432896 -1.507343 0.839837

C -2.151877 -0.095533 1.347458

C -2.622010 1.000932 0.369139

C -3.960294 0.793787 -0.202806

C -4.806773 1.889614 -0.494159

C -6.097178 1.634632 -0.939609

C -6.553616 0.330066 -1.114863

C -5.717175 -0.750568 -0.848374

C -4.433968 -0.521714 -0.388281

C -2.778758 -1.951928 -1.582949

C -2.924828 0.081817 2.665233

C -4.385808 3.322318 -0.311344

C 0.118663 -0.115733 0.672801

C 1.474280 0.176017 1.113135

C 2.523195 0.037952 0.275679

C 3.884997 0.313314 0.619022

C 4.903559 0.161330 -0.258636

C 6.285148 0.435469 0.037257

C 7.268731 0.271826 -0.862229

C 8.733796 0.515040 -0.660123

C 9.507389 -0.796856 -0.928117

C 11.022962 -0.622660 -1.004975

C 9.081240 1.129742 0.694707

O -0.777318 0.071846 1.682392

O -0.230714 -0.491031 -0.431087

O -1.879370 -2.469124 1.320180

O -4.271854 -2.855039 0.109949

O -1.944338 1.998617 0.215287

H -6.762644 2.464531 -1.146629

O -7.808147 0.051386 -1.552102

H 10.142811 1.368743 0.749792

H 11.292231 0.120438 -1.760197

H 11.504845 -1.564074 -1.276045

H 9.252280 -1.516191 -0.143428

H 9.148718 -1.224649 -1.868562

H 9.040735 1.220911 -1.444817

H 6.997322 -0.092357 -1.851189

H 11.445628 -0.299969 -0.052299

H 8.526087 2.053670 0.865432

H 8.851286 0.439524 1.510447

H 6.510706 0.792181 1.036756

H 4.676517 -0.188511 -1.262905

H 4.093985 0.660649 1.626734

H 2.325632 -0.303982 -0.736528

H 1.602221 0.511767 2.133873

H -6.073628 -1.756988 -1.008700

H -3.642471 -3.540817 0.375269

H -2.105113 -2.804154 -1.486780

H -2.197537 -1.083544 -1.883732

H -3.534427 -2.167805 -2.337744

H -2.562307 -0.647357 3.387953

H -3.992349 -0.064152 2.511683

H -2.755041 1.088131 3.046657

H -3.628268 3.603939 -1.043110

H -3.945123 3.489883 0.671689

H -5.244996 3.981730 -0.427356

H -8.296122 0.868526 -1.719917

4a'-c33 , delta G = 1.6252 kcal/mol, population = 3.06 %

C -3.480362 -1.530971 -0.736702

C -2.441376 -1.672528 0.381904

C -2.155098 -0.471191 1.278923

C -2.575168 0.870155 0.643090

C -3.898964 0.866430 0.003284

C -4.719403 2.019212 0.015693

C -6.003359 1.929218 -0.506385

C -6.480186 0.735276 -1.043001

C -5.667308 -0.394553 -1.079970

C -4.390600 -0.332201 -0.554707

C -2.721002 -1.390452 -2.072653

C -2.969730 -0.657908 2.570633

C -4.279087 3.333264 0.600879

C 0.139630 -0.368828 0.694446

C 1.484619 -0.293348 1.244824

C 2.567016 -0.237150 0.441543

C 3.920047 -0.175522 0.904777

C 4.981352 -0.130966 0.066876

C 6.354321 -0.082350 0.495491

C 7.394748 -0.044108 -0.351786

C 8.833338 0.000129 0.059059

C 9.490927 1.354042 -0.301225

C 9.552406 1.684076 -1.792467

C 9.601155 -1.189334 -0.538680

O -0.790784 -0.444245 1.687120

O -0.174309 -0.384038 -0.481192

O -1.914878 -2.743156 0.578368

O -4.275380 -2.713190 -0.752449

O -1.877035 1.853287 0.798902

H -6.648280 2.799933 -0.485525

O -7.732265 0.616571 -1.553653

H 10.658162 -1.135433 -0.268512

H 8.556708 1.742489 -2.236519

H 10.123788 0.939507 -2.349415

H 8.949823 2.146625 0.222774

H 10.505399 1.346078 0.108884

H 8.872152 -0.086099 1.149093

H 7.193797 -0.051765 -1.420794

H 10.036090 2.650578 -1.946519

H 9.200716 -2.133865 -0.166848

H 9.531311 -1.204146 -1.627937

H 6.539187 -0.081452 1.566907

H 4.801082 -0.135966 -1.005406

H 4.086668 -0.170976 1.978131

H 2.407433 -0.243609 -0.633082

H 1.578124 -0.290503 2.322924

H -6.037274 -1.307959 -1.520931

H -3.663312 -3.459404 -0.679010

H -3.456930 -1.357035 -2.875537

H -2.066247 -2.251107 -2.213750

H -2.115511 -0.487443 -2.092782

H -4.033391 -0.730649 2.352119

H -2.799260 0.195015 3.226733

H -2.640681 -1.569843 3.066055

H -3.861646 3.208211 1.600222

H -5.123821 4.018701 0.656617

H -3.497980 3.790460 -0.006896

H -8.206383 1.455468 -1.480867

4a'-c45 , delta G = 1.7815 kcal/mol, population = 2.35 %

C 3.423156 1.729383 -0.019586

C 2.362952 1.376130 1.031786

C 2.099336 -0.093596 1.349016

C 2.573248 -1.044121 0.230278

C 3.908724 -0.750770 -0.310278

C 4.766120 -1.789649 -0.742945

C 6.053145 -1.464469 -1.151358

C 6.496393 -0.143717 -1.150176

C 5.648938 0.883740 -0.744358

C 4.368598 0.582609 -0.320081

C 2.690730 2.134161 -1.315824

C 2.884012 -0.436503 2.626689

C 4.361144 -3.238496 -0.748829

C -0.177439 -0.010934 0.698959

C -1.524491 -0.382228 1.104381

C -2.585495 -0.138297 0.307262

C -3.938321 -0.481077 0.624471

C -4.974893 -0.213683 -0.203048

C -6.346425 -0.549775 0.075291

C -7.353228 -0.266132 -0.767223

C -8.807455 -0.566641 -0.562361

C -9.641730 0.734729 -0.503840

C -9.568727 1.605524 -1.756441

C -9.095888 -1.441935 0.656666

O 0.729666 -0.320864 1.667530

O 0.156640 0.517614 -0.345185

O 1.804270 2.260270 1.638622

O 4.180458 2.831242 0.473095

O 1.901468 -2.015638 -0.057569

H 6.726034 -2.252663 -1.468935

O 7.748837 0.202650 -1.542484

H -10.160141 -1.677675 0.704408

H -8.569574 2.015572 -1.913024

H -10.259050 2.447657 -1.679178

H -10.681931 0.447659 -0.326618

H -9.324007 1.311391 0.370553

H -9.141459 -1.108068 -1.458232

H -7.104715 0.241525 -1.695343

H -9.839738 1.033891 -2.647973

H -8.543543 -2.382402 0.617182

H -8.827519 -0.930512 1.584244

H -6.543834 -1.057581 1.013612

H -4.772500 0.286676 -1.147048

H -4.125620 -0.978777 1.571615

H -2.406254 0.350903 -0.646139

H -1.636789 -0.864004 2.066824

H 5.995188 1.906048 -0.768824

H 3.543766 3.462834 0.837262

H 3.440490 2.449400 -2.040951

H 2.012082 2.962499 -1.109350

H 2.114346 1.306261 -1.721485

H 2.516372 0.182656 3.443287

H 3.948145 -0.255408 2.488788

H 2.730794 -1.487633 2.868936

H 3.913268 -3.535033 0.199795

H 5.229653 -3.868003 -0.938429

H 3.614618 -3.433321 -1.519292

H 8.246776 -0.581617 -1.808461

4a'-c58 , delta G = 1.8574 kcal/mol, population = 2.07 %

C -3.887611 -1.568512 0.200451

C -2.420682 -1.560596 0.649534

C -1.646492 -0.245562 0.622965

C -2.279926 0.807070 -0.309146

C -3.739667 0.940655 -0.197223

C -4.373899 2.193745 -0.370329

C -5.743745 2.282054 -0.159138

C -6.489713 1.162321 0.202696

C -5.872608 -0.076320 0.354642

C -4.507886 -0.185353 0.164742

C -3.946787 -2.185952 -1.212135

C -1.655666 0.320623 2.052951

C -3.626769 3.446648 -0.738521

C -0.014710 -1.030548 -0.903031

C 1.402231 -1.134030 -1.208947

C 2.390067 -0.665306 -0.416994

C 3.785353 -0.767511 -0.722076

C 4.750337 -0.286188 0.094996

C 6.162082 -0.372305 -0.172085

C 7.094182 0.114537 0.662951

C 8.581075 0.090920 0.474953

C 9.121017 1.539368 0.524912

C 10.643931 1.635771 0.591752

C 9.038568 -0.657988 -0.775496

O -0.275210 -0.456791 0.303491

O -0.910083 -1.424383 -1.629349

O -1.920993 -2.574742 1.078438

O -4.623270 -2.376415 1.115398

O -1.562400 1.525966 -0.977494

H -6.238725 3.239930 -0.269618

O -7.829279 1.219017 0.414893

H 8.651274 -1.678117 -0.792044

H 11.033551 1.068811 1.441320

H 10.958465 2.674442 0.710774

H 8.749813 2.078878 -0.352288

H 8.692979 2.038477 1.398873

H 8.995968 -0.423896 1.353224

H 6.755559 0.598346 1.577036

H 11.117707 1.251318 -0.312672

H 8.699852 -0.151287 -1.682767

H 10.125732 -0.718238 -0.813277

H 6.456411 -0.859835 -1.095401

H 4.453675 0.200382 1.021116

H 4.066087 -1.255577 -1.650997

H 2.123000 -0.177106 0.514570

H 1.622543 -1.622678 -2.149668

H -6.463879 -0.941825 0.613515

H -4.101042 -3.178734 1.258212

H -4.993934 -2.256972 -1.504928

H -3.504052 -3.182432 -1.194683

H -3.405087 -1.578819 -1.933456

H -1.151621 -0.382833 2.713555

H -2.674692 0.474870 2.402557

H -1.127864 1.273666 2.064251

H -4.280526 4.312758 -0.643884

H -3.259355 3.396643 -1.763695

H -2.753726 3.595108 -0.102605

H -8.154319 2.118248 0.275439

# Table S81. The experimental ECD curve of **5**


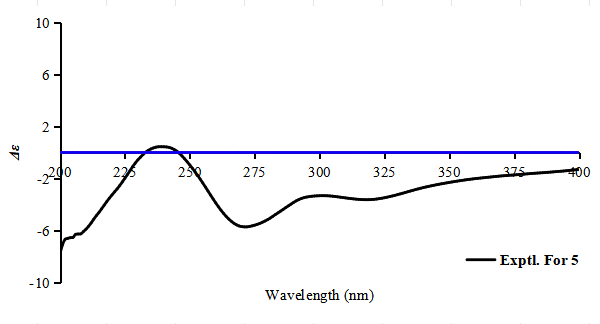


# Table S82. Geometry data of conformers of compound 6

6-c1, delta G = 0.0000 kcal/mol, population = 100.00 %

C 3.147384 0.129803 -0.000846

C 3.103806 -1.257404 0.075793

C 1.884246 -1.913957 0.118569

C 0.693829 -1.193800 0.091427

C 0.737794 0.212785 -0.005163

C 1.982737 0.889286 -0.052580

C -0.605111 -1.958609 0.190418

C -1.835267 -1.178127 -0.228385

C -1.815200 0.233223 0.362705

C -0.535300 0.949348 -0.090752

O -0.640787 2.100936 -0.489244

C 2.107894 2.385917 -0.141509

C -1.878621 0.230590 1.893775

O -2.935102 0.932466 -0.169181

O -1.856838 -1.102199 -1.652524

H -2.737232 -1.687381 0.130395

H 4.104789 0.635093 -0.026515

H 4.025842 -1.824860 0.101139

H 1.849542 -2.995183 0.173387

H -0.542811 -2.862971 -0.416791

H -0.736555 -2.293062 1.223280

H 1.498429 2.890372 0.608564

H 3.148726 2.678027 -0.002915

H 1.767599 2.754017 -1.109813

H -1.005974 -0.244762 2.340977

H -1.934451 1.257898 2.254471

H -2.775337 -0.299230 2.217661

H -2.598294 1.812891 -0.412164

H -2.531603 -0.444805 -1.874799

# Table S83. Geometry data of conformers of compound 7

7-c1, delta G = 0.0000 kcal/mol, population = 100.00 %

C 3.229038 -0.198848 0.062589

C 3.015100 -1.572863 0.050397

C 1.726304 -2.077068 -0.017337

C 0.633289 -1.216107 -0.064414

C 0.852034 0.179000 -0.074360

C 2.169414 0.699484 -0.009235

C -0.750632 -1.820314 -0.086909

C -1.820556 -0.855765 -0.554299

C -1.688432 0.489154 0.169048

C -0.317134 1.070696 -0.186112

O -0.267313 2.237088 -0.553106

C 2.476000 2.172552 0.005649

C -1.817056 0.382096 1.692668

O -2.705081 1.352623 -0.327399

O -3.093896 -1.450878 -0.330763

H -1.693721 -0.654873 -1.625438

H 4.239464 0.185031 0.125837

H 3.859185 -2.249701 0.095345

H 1.560199 -3.147119 -0.028931

H -0.752657 -2.707170 -0.721942

H -1.008522 -2.160331 0.920621

H 2.263003 2.630317 -0.960989

H 1.871431 2.705509 0.739781

H 3.529083 2.327431 0.239142

H -1.774885 1.381708 2.125635

H -2.777391 -0.068309 1.940878

H -1.021874 -0.218064 2.133630

H -2.247564 2.170508 -0.589462

H -3.759644 -0.817690 -0.630067

# **Table S84.** The ITS sequences of *Penicillium expansum* DWS880

| CTTGGTCACATTTGAGGAAGTAAAAGTCGTAACAAGGTTTCCGTAGGTGAACCTGCGGAAGGATCATTACTGAGTGAGGGCCCTCTGGGTCCAACCTCCCACCCGTGTTTATTGTACCTTGTTGCTTCGGTGCGCCCGCCTCACGGCCGCCGGGGGGCTTCTGCCCCCGGGTCCGCGCGCACCGGAGACACTATTGAACTCTGTCTGAAGATTGCAGTCTGAGCATAAACTAAATAAGTTAAAACTTTCAACAACGGATCTCTTGGTTCCGGCATCGATGAAGAACGCAGCGAAATGCGATAACTAATGTGAATTGCAGAATTCAGTGAATCATCGAGTCTTTGAACGCACATTGCGCCCCCTGGTATTCCGGGGGGCATGCCTGTCCGAGCGTCATTGCTGCCCTCAAGCACGGCTTGTGTGTTGGGCTCCGTCCCCCCGGGGACGGGTCCGAAAGGCAGCGGCGGCACCGAGTCCGGTCCTCGAGCGTATGGGGCTTTGTCACCCGCTCTGTAGGCCCGGCCGGCGCCAGCCGACAACCAATCATCCTTTTTTCAGGTTGACCTCGGATCAGGTAGGGATACCCGCTGAACTTAAGCATA |
| --- |


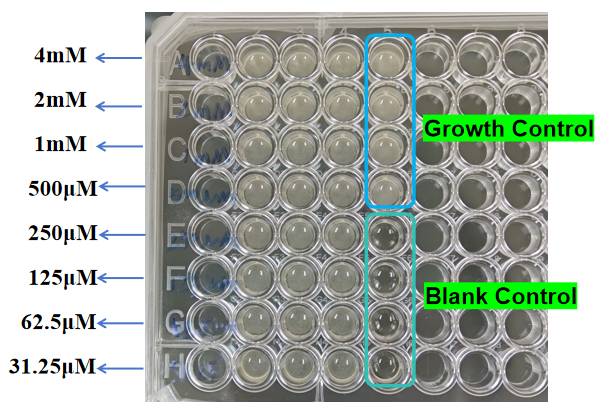


# Fig. S85. **Initial evaluation of fluconazole resistance in *Candida albicans.*** The tested *C. albicans* strain showed no visual growth inhibition across a fluconazole concentration range from 31.25 μM to 4 mM. BC, blank control (medium only); GC, growth control (medium and inoculum).


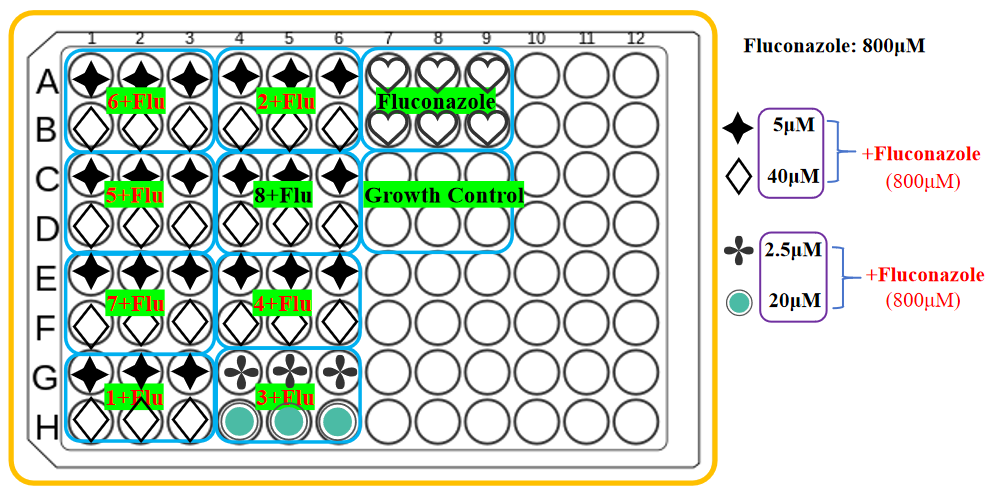


# Fig. S86. **Screening for fluconazole potentiators against resistant *Candida albicans*.** The assay identified compounds that synergize with fluconazole:

• Test Compounds: **1**, **2**, **4**-**8** at 5 & 40 μM; **3** at 2.5 & 20 μM.

• Fluconazole: Fixed concentration of 800 μM (ineffective alone).

• Controls: Blank (medium), Growth control (inoculum), Fluconazole control (800 μM).

• Result Interpretation: The absence of turbidity in combination wells signifies potentiation and complete growth inhibition.


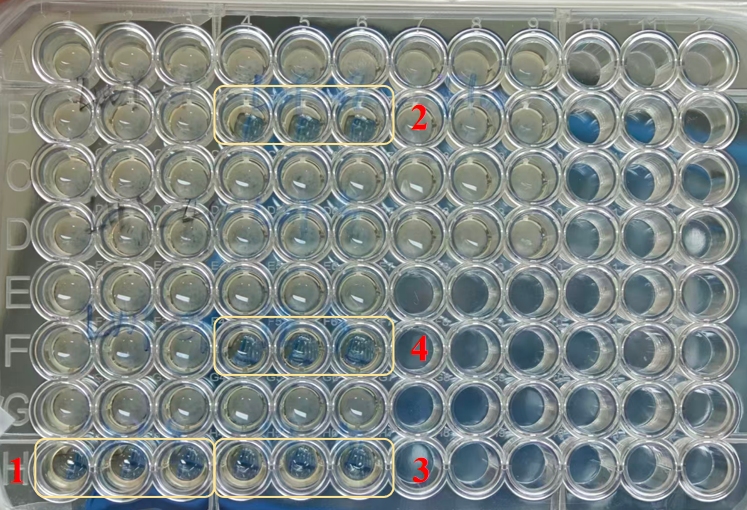


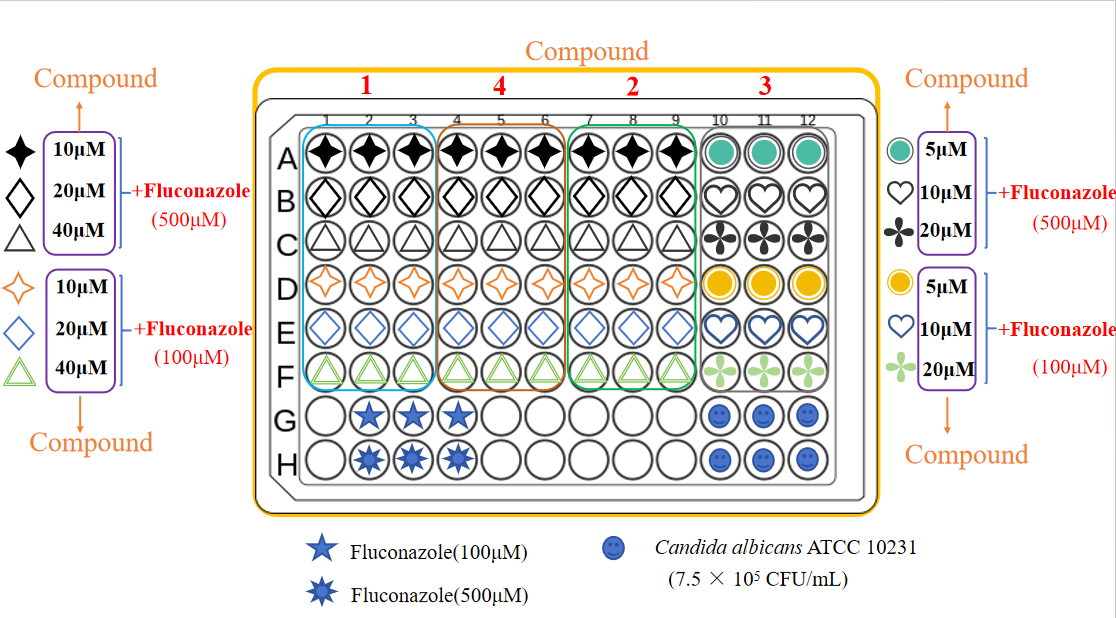


Fig. S87. Synergy screening using fixed-ratio combinations. Assessment of the synergistic potential of compounds **1**–**4** in combination with fluconazole against *C. albicans*. Compounds **1**, **2**, and **4** were tested at 10, 20, and 40 μM, while compound **3** was evaluated at lower concentrations (5, 10, and 20 μM) due to its intrinsic antifungal activity (MIC=50 μM). All compounds were combined with fixed concentrations of fluconazole (100 and 500 μM). Fungal growth was assessed by visual evaluation of culture turbidity.


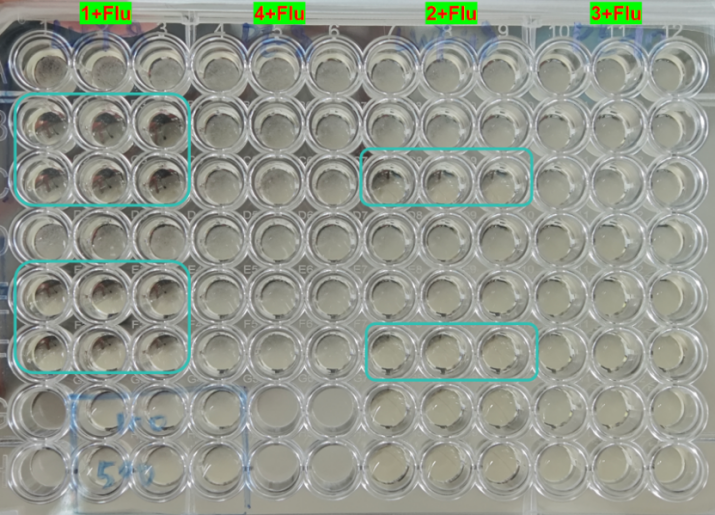


Top view of the 96-well plate


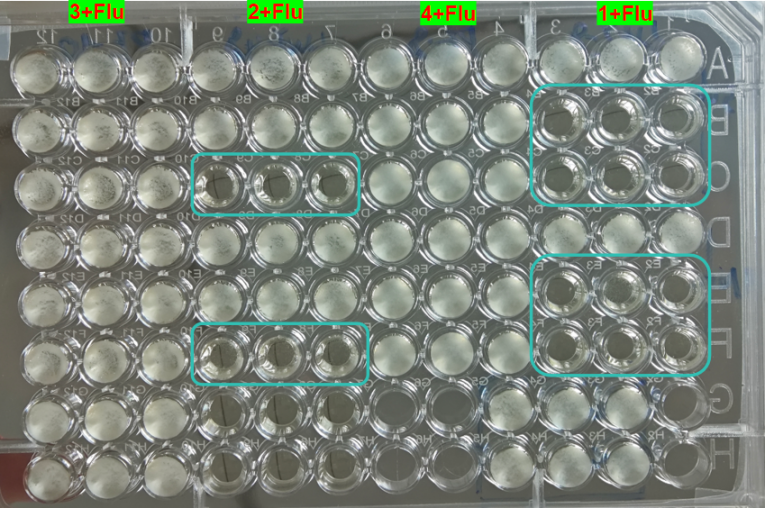


Bottom view of the 96-well plate for visual turbidity assessment


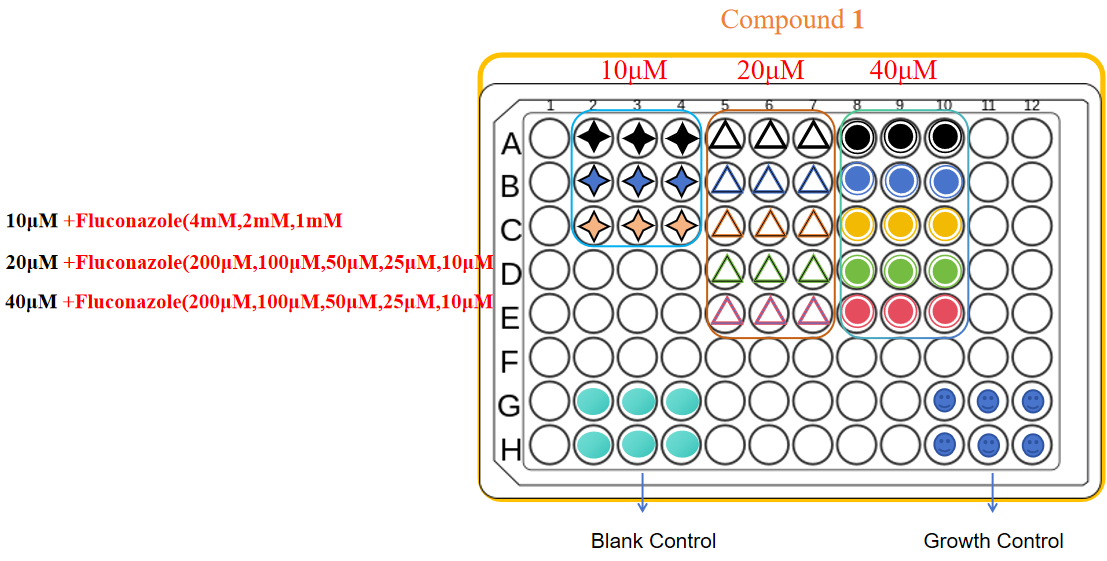


# Fig. S88. **Detailed characterization of potentiation efficacy for Compound 1.**

Follow-up assays to precisely determine the extent of fluconazole MIC reduction by the most promising synergists (**1** and **2**). Concentration ranges were optimized from the primary screen data.


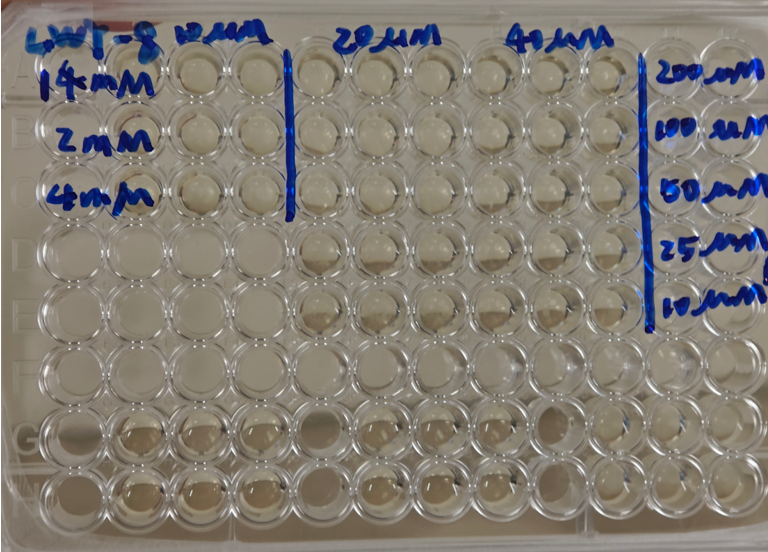


Top view of the 96-well plate


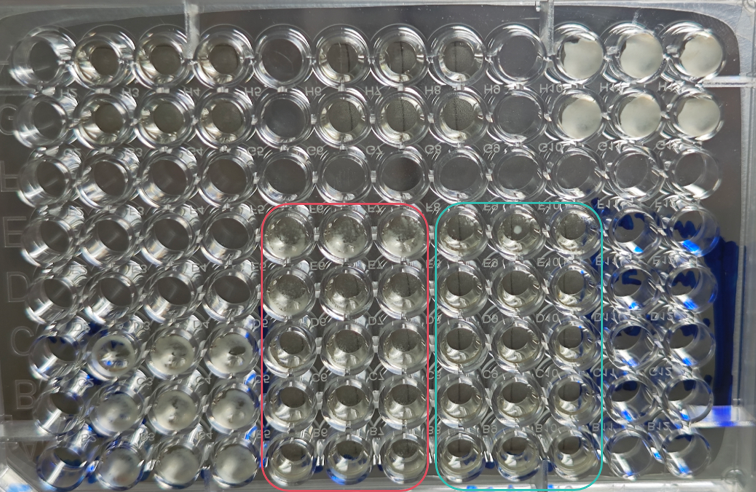


Bottom view of the 96-well plate for visual turbidity assessment


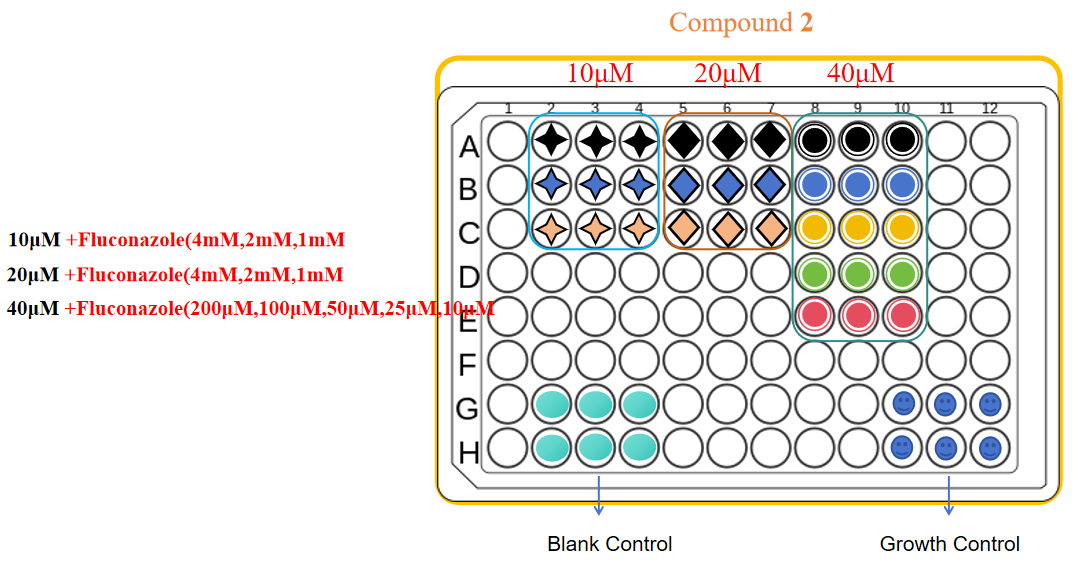


# Fig. S89. Detailed characterization of potentiation efficacy for Compound **2**


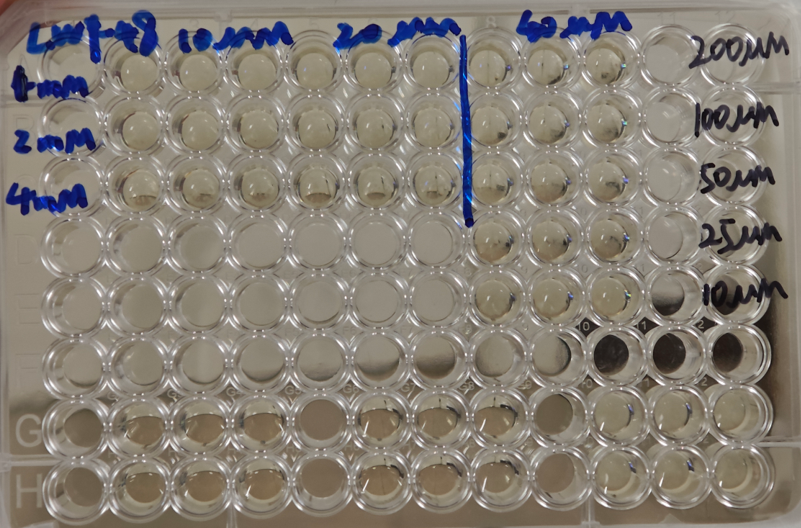


Top view of the 96-well plate


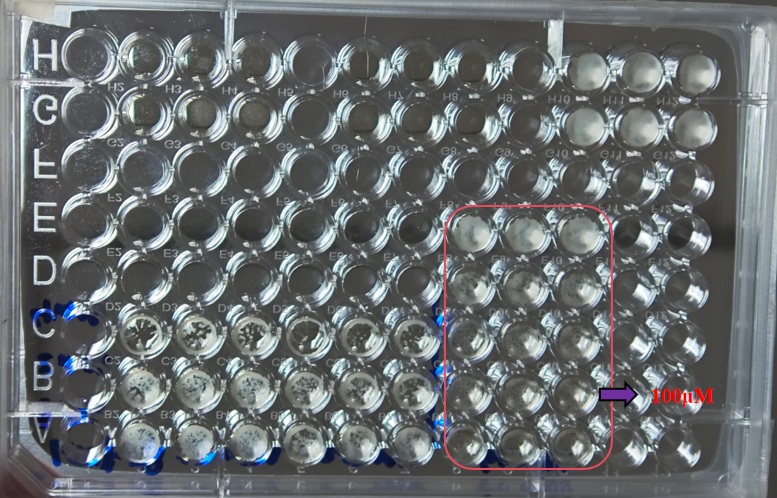


Bottom view of the 96-well plate for visual turbidity assessment


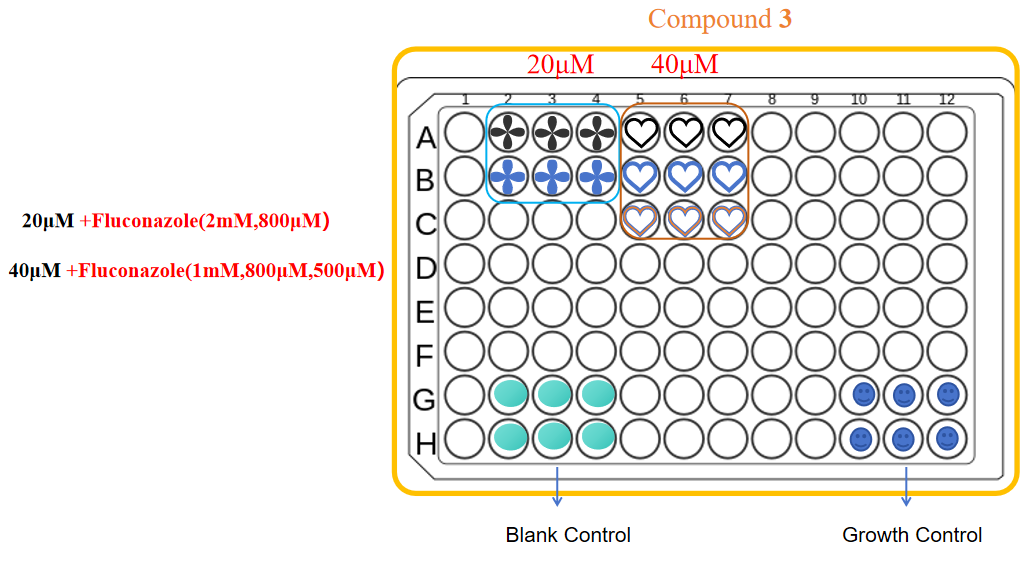


# Fig. S90. Detailed characterization of potentiation efficacy for Compound **3**


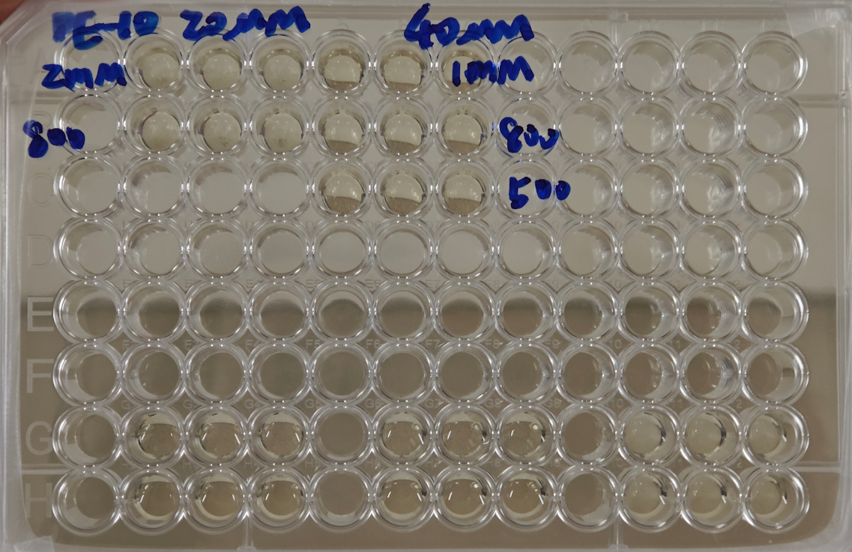


Top view of the 96-well plate


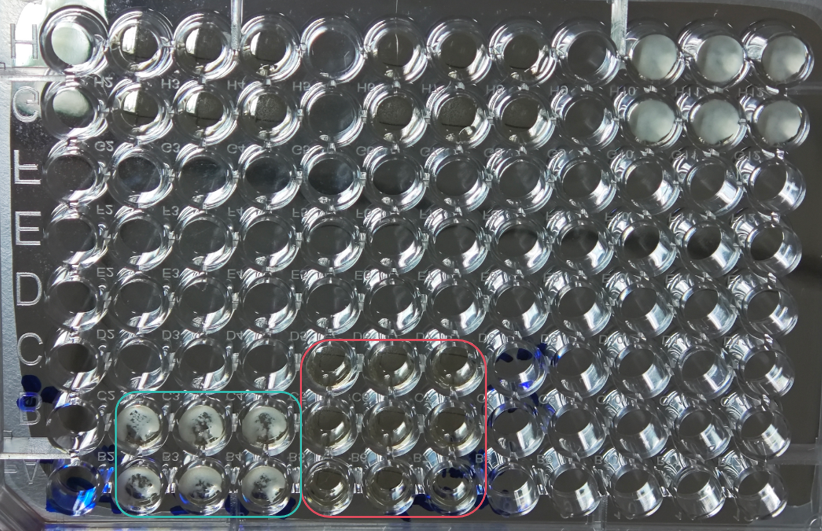


Bottom view of the 96-well plate for visual turbidity assessment
